# Tracing Foodborne Botulism Events Caused by Clostridium botulinum in Xinjiang Province, China, Using a Core Genome Sequence Typing Scheme

**DOI:** 10.1128/spectrum.01164-22

**Published:** 2022-11-15

**Authors:** Xin Ma, Kexin Li, Fang Li, Jing Su, Weiwei Meng, Yanming Sun, Hui Sun, Jiazheng Sun, Yonghe Yuan, Yujia Lin, Songnian Hu, Xuefang Xu, Zilong He

**Affiliations:** a Center for Disease Control and Prevention of Xinjiang Uygur Autonomous Region, Urumqi, China; b State Key Laboratory of Microbial Resources, Institute of Microbiology, Chinese Academy of Sciences, Beijing, China; c School of Engineering Medicine, Beihang University, Beijing, China; d Beijing Advanced Innovation Center for Big Data-Based Precision Medicine, Interdisciplinary Innovation Institute of Medicine and Engineering, Beihang University, Beijing, China; e State Key Laboratory for Infectious Disease Prevention and Control, Chinese Center for Disease Control and Prevention, Beijing, China; f National Institute for Communicable Diseases Control and Prevention, Chinese Center for Disease Control and Prevention, Beijing, China; g Criminal Investigation School, People's Public Security University of China, Beijing, China; University of California, Davis

**Keywords:** foodborne botulism, *Clostridium botulinum*, phylogenetic tree, core genome markers, whole-genome sequencing

## Abstract

Foodborne botulism is a rare but life-threatening illness resulting from the action of a potent toxin mainly produced by Clostridium botulinum. It grows in an oxygen-deficient environment and is extremely viable in meat and soy products, making it one of the most virulent bacteria. How to track foodborne botulism events quickly and accurately has become a key issue. Here, we investigated two foodborne botulism events that occurred in Xinjiang in 2019 based on whole-genome sequencing and also successfully traced the relationship between clinical and food C. botulinum isolates using whole-genome core gene markers. All 59 isolates were classified as group I strains. Of the strains isolated in this study, 44 were found to be botulinum toxin A(B), and 15 isolates contained only the toxin B locus. Both the toxin A and B gene segments were located on the chromosome and organized in an ha cluster. Antibiotic resistance and virulence factors were also investigated. A set of 329 universal core gene markers were established using C. botulinum strains from a public database. These core gene markers were applied to the published C. botulinum genomes, and three outbreaks were identified. This work demonstrates that universal core gene markers can be used to trace foodborne botulism events, and we hope that our work will facilitate this effort in future.

**IMPORTANCE** In this study, we analyzed 59 foodborne botulism (FB)-related strains isolated in Xinjiang Province, China. Our findings not only reveal the group classification, neurotoxin locus organization, antibiotic resistance and virulence factors of these strains but also establish a set of core gene markers for tracing foodborne botulism events, which was verified using published genomes. These findings indicate that these gene markers might be used as a potential tracing tool for FB events caused by C. botulinum group I strains, which have relatively stable genomic components.

## INTRODUCTION

Botulism is a life-threatening acute neuroparalysis caused by botulinum toxins (BoNTs). BoNTs are mainly produced by Clostridium botulinum (1). C. botulinum strains are divided into four different groups (I to IV), which vary in their physiological and phylogenetic features. Group I strains are proteolytic and include BoNT types A, B, and F. Members of group II are nonproteolytic and consist of BoNT types B, E, and F.

Group I and II strains are responsible for most botulism cases in humans (2–5). Group III strains produce BoNT types C and D, causing botulism in animals (6, 7). Finally, members of group IV produce BoNT G, which is the least studied (8). These BoNTs are grouped into seven serotypes, A, B, C, D, E, F, and G, based on antigenic properties, target sites, differences in structure, and toxicity (9). Usually, one strain consists of 1 BoNT type only. Some strains can produce 2 toxins, with the lower amount of toxin indicated by a lowercase letter (10).

There are 6 forms of botulism, according to the route of intoxication, including foodborne botulism (FB), wound botulism, infant botulism, adult intestinal colonization, iatrogenic botulism, and inhalational botulism (11). In the United States, FB is mainly associated with consuming home-canned, preserved, or fermented vegetables, meat, or fish (11–13). In Europe, FB is frequently caused by homemade, canned fish, ham, pork, or other meat products (14–18). In China, FB is usually related to homemade bean products, sausage, cereal, pork, or other meat products (19–22). Outbreaks of foodborne botulism are related to improperly canned or bottled foods and to the failure to keep refrigerated products at an appropriate temperature (23). FB is a serious and fatal poisoning caused by eating food containing as little as 50 ng botulinum neurotoxin (24).

In 1897, following an outbreak of FB associated with salted ham in Belgium, the first isolation of a strain of *C. botulism* was reported (25). Nowadays, FB is still the main type of botulism. Food traceability is critically important in FB. However, few studies have been conducted on tracing the source of FB events using whole-genome sequencing data. A C. botulinum type B strain collected in May 2015 was investigated to trace potential sources of transmission using high-throughput sequencing (21). In 2017, a large FB outbreak in California was caused by commercial nacho cheese sauce dispensed at a gas station market. Epidemiological and multilocus sequence typing (MLST) scheme evidence confirmed that the cheese sauce was the outbreak source (26). In 2021, an outbreak of 4 patients consuming vacuum-packaged salted fish and ham with botulinum types A, B, and E was reported (27). Thus, how to trace the source of FB events quickly and accurately still needs further study.

Here, we investigated two FB outbreaks in 2019 in Xinjiang Province, China. We inferred that these two outbreaks were correlated with stinky tofu using core genome MLST. Meanwhile, we established a set of universal core gene markers for botulism outbreaks, which was verified by tracing three possible outbreaks from published C. botulinum genomes. Our goal is to make it convenient for researchers to trace the source of FB outbreaks.

## RESULTS

### Botulinum neurotoxin types and MLST determination.

In total, 59 clinical and environmental strains of C. botulinum were isolated and sequenced in this study. Information about these strains is provided in Table 1. In addition, the assembly quality and annotation completeness of the newly sequenced genomes and published genomes were checked (see Tables S1 and S2 in the supplemental material). The average contig *N*_50_ value was 1,407,239 bp, and the average number of contigs was 107. There were 44 C. botulinum isolates confirmed as botulinum toxin A(B) and 15 isolates confirmed as toxin B. Eight C. botulinum strains were isolated from events A and B. In event A, strains XJFE01 and XJFD23 were isolated from feces and food, respectively. In event B, five strains (XJFE02, XJFE03, XJFE04, XJFE05, and XJFE06) were isolated from food and one strain (XJFD26) from stool. All the strains in these two outbreak events were botulinum toxin A(B). The other 51 environmental strains were nonclinical isolates recovered from soil and food (air dried meat, fermented bean curd, honey, horseflesh, soybean paste, and stinky tofu). In all, 54 strains were confirmed as MLST sequence type 16 (ST16); the others, XJSL24, XJSL25, XJFD17, XJFD20, and XJSL26, had a new MLST type [*aroE*(13), *mdh*(5), *aceK*(17), *oppB*(5), *rpoB*(9), *recA*(7), and *hsp*(6)]. The genomic features of the isolates and public strains are shown in Table S3. The average number of open reading frames (ORFs) in the environmental strains in this study was 3,654, which is higher than that in isolates from the foodborne botulism outbreak event (average, 3,615), and the average number of ORFs in the 593 publicly available C. botulinum genomes was 3,877. In addition, the average genome size of the environmental isolates (about 3,885,750 bp) was larger than that in the botulism outbreak events (about 3,855,638 bp). All strains in this study were classified into group I on the basis of whole-genomic single nucleotide polymorphisms (SNPs) and formed a single phylogenetic unit with the public group I strains (Fig. S1).

**TABLE 1:** Basic information on the isolates in this study

Isolate	Environmental source	Source	Botulinum toxin	Total length (bp)	*N*_50_ (bp)	*N*_90_ (bp)	Max contig size (bp)	No. of contigs	No. of SNPs
XJFE01	Feces	Feces	A(B)	3,825,915	955,342	296,787	1,061,969	89	3
XJFE02	Feces	Feces	A(B)	3,827,365	956,012	296,787	1,061,969	114	4
XJFE03	Feces	Feces	A(B)	3,828,361	955,342	296,497	1,061,969	104	3
XJFE04	Feces	Feces	A(B)	3,827,763	955,342	296,787	1,061,969	89	5
XJFE05	Feces	Feces	A(B)	3,828,797	2,179,046	420,481	2,179,046	75	6
XJFE06	Feces	Feces	A(B)	3,829,643	816,599	146,162	956,627	84	5
XJFD01	Horseflesh	Food	A(B)	3,884,325	2,792,238	414,835	2,792,238	121	9
XJFD02	Horseflesh	Food	A(B)	3,828,936	956,642	296,788	1,063,985	80	11
XJFD03	Horseflesh	Food	A(B)	3,895,533	2,810,310	415,071	2,810,310	125	7
XJFD04	Horseflesh	Food	A(B)	4,142,761	3,234,046	218,207	3,234,046	125	11
XJFD05	Horseflesh	Food	A(B)	3,833,009	956,573	296,788	1,063,812	121	14
XJFD06	Horseflesh	Food	A(B)	3,894,149	3,215,118	415,381	3,215,118	189	8
XJFD07	Air dried meat	Food	A(B)	4,045,546	815,982	217,210	1,063,813	99	11
XJFD08	Horseflesh	Food	A(B)	3,901,112	2,087,846	415,070	2,087,846	128	8
XJFD09	Horseflesh	Food	A(B)	3,830,383	2,179,678	420,369	2,179,678	90	13
XJFD10	Horseflesh	Food	A(B)	3,883,146	956,642	296,787	1,063,813	246	8
XJFD11	Horseflesh	Food	A(B)	4,098,077	222,904	28,925	420,354	299	10
XJFD12	Horseflesh	Food	A(B)	3,901,752	2,087,864	414,833	2,087,864	104	11
XJFD13	Horseflesh	Food	A(B)	3,900,191	2,087,864	414,835	2,087,864	103	13
XJFD14	Horseflesh	Food	A(B)	3,854,163	799,272	140,528	956,857	100	14
XJFD15	Horseflesh	Food	A(B)	3,851,096	799,272	140,528	956,857	109	11
XJFD16	Stinky tofu	Food	A(B)	4,113,611	900,068	140,499	1,968,362	221	12
XJFD17	Stinky tofu	Food	B	3,850,417	799,272	140,528	956,866	101	11
XJFD18	Stinky tofu	Food	A(B)	4,315,655	3,233,729	407,450	3,233,729	109	9
XJFD19	Soybean paste	Food	A(B)	3,851,551	799,272	140,528	956,857	91	4
XJFD20	Honey	Food	B	3,904,121	3,233,472	414,878	3,233,472	102	7
XJFD21	Stinky tofu	Food	A(B)	3,826,428	955,342	296,787	1,061,969	94	14
XJFD22	Stinky tofu	Food	A(B)	3,824,926	955,654	296,787	1,062,141	109	8
XJFD23	Stinky tofu	Food	A(B)	3,839,955	956,643	296,788	1,063,985	97	8
XJFD24	Fermented bean curd	Food	A(B)	3,828,834	956,303	296,788	1,063,248	83	8
XJFD25	Fermented bean curd	Food	A(B)	3,849,585	1,957,964	140,710	1,957,964	168	7
XJFD26	Stinky tofu	Food	A(B)	3,850,329	1,957,964	140,422	1,957,964	103	6
XJFD27	Stinky tofu	Food	A(B)	3,827,407	499,330	296,787	1,061,561	100	6
XJSL01	Soil	Soil	A(B)	3,825,238	955,342	295,510	1,061,969	118	11
XJSL02	Soil	Soil	B	3,826,427	955,342	296,787	1,061,969	85	12
XJSL03	Soil	Soil	A(B)	3,825,232	955,342	296,787	1,061,969	90	11
XJSL04	Soil	Soil	B	3,824,657	955,342	296,081	1,061,969	106	13
XJSL05	Soil	Soil	B	3,822,412	955,342	296,787	1,061,969	76	9
XJSL06	Soil	Soil	A(B)	3,822,853	955,342	296,787	1,062,141	82	8
XJSL07	Soil	Soil	B	3,828,534	956,591	296,788	1,063,814	95	9
XJSL08	Soil	Soil	A(B)	3,808,495	2,084,406	330,456	2,084,406	113	6
XJSL09	Soil	Soil	B	3,826,541	816,600	146,162	956,627	82	8
XJSL10	Soil	Soil	A(B)	3,822,393	955,342	296,788	1,061,969	78	10
XJSL11	Soil	Soil	A(B)	3,810,519	2,084,406	330,456	2,084,406	91	9
XJSL12	Soil	Soil	A(B)	3,824,661	955,342	235,169	1,062,141	91	6
XJSL13	Soil	Soil	B	3,822,627	955,342	296,787	1,061,969	89	8
XJSL14	Soil	Soil	B	3,853,316	955,677	295,511	1,066,496	91	8
XJSL15	Soil	Soil	A(B)	3,825,444	955,149	296,788	1,063,853	126	9
XJSL16	Soil	Soil	A(B)	3,827,011	956,591	296,788	1,063,814	90	10
XJSL17	Soil	Soil	B	3,827,434	956,591	296,788	1,063,814	100	9
XJSL18	Soil	Soil	A(B)	3,843,751	1,958,015	140,415	1,958,015	99	13
XJSL19	Soil	Soil	B	3,850,252	2,204,596	420,419	2,204,596	97	7
XJSL20	Soil	Soil	A(B)	3,849,868	2,204,596	420,319	2,204,596	75	11
XJSL21	Soil	Soil	B	3,849,341	2,204,696	420,102	2,204,696	74	10
XJSL22	Soil	Soil	A(B)	3,850,401	2,204,576	420,419	2,204,576	81	9
XJSL23	Soil	Soil	A(B)	3,824,788	2,179,624	420,278	2,179,624	85	8
XJSL24	Soil	Soil	B	3,845,461	955,677	296,788	1,066,668	51	7
XJSL25	Soil	Soil	B	3,844,141	523,931	135,440	772,039	64	8
XJSL26	Soil	Soil	B	3,914,753	202,356	53,918	397,771	97	9

### Pangenomic analysis and genetic population structure.

**FIG 1:**
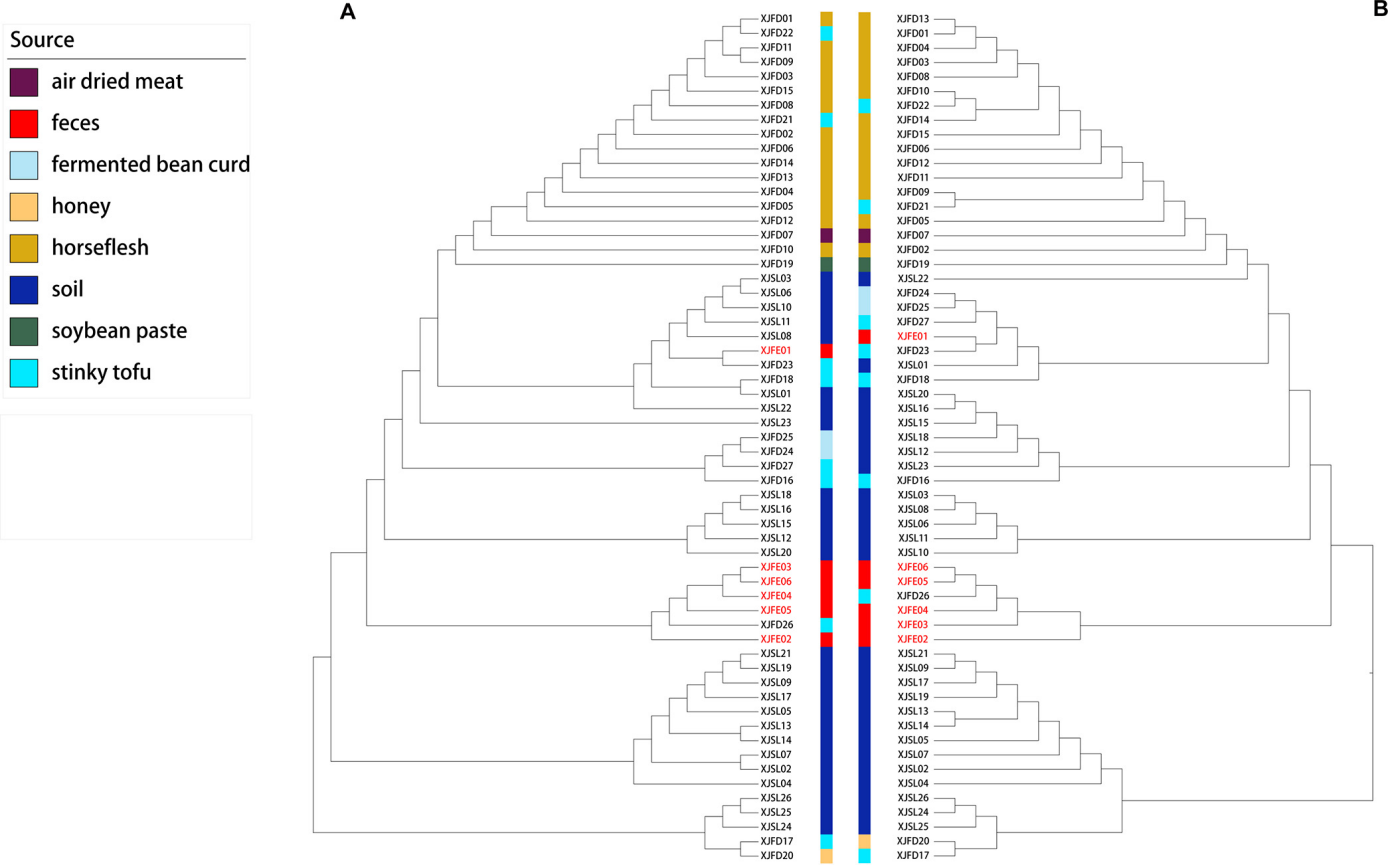
Phylogenetic trees showing the relationships between C. botulinum genomes. Isolates in red text represent clinical strains from botulism outbreaks. The neighbor joining (NJ) method was used to construct phylogenetic trees using (A) 2,933 core genes shared by the 59 C. botulinum isolates in this study and (B) 329 core genes shared by public C. botulinum genome sequences and our 59 isolates.

Pangenomic analysis of all the genomes was conducted in the current study. The core genes of the isolates in this study are listed in Table S4. These 59 C. botulinum genomes shared 2,933 core genes (present in ≥99% of isolates and with 95% identity) and 3,171 accessory genes. As shown by functional annotations using KEGG, the main pathways of the 2,933 core genes were ABC transporters, two-component systems, ribosomes, and so on (Fig. S2A). A phylogenetic tree was built based on a core genome alignment, showing the lineage designation of the isolates (Fig. 1A). In the first outbreak, event A, in Ili Kazak Autonomous Prefecture, the clinical isolate (XJFE01) and the stinky tofu isolate (XJFD23) formed a monophyletic branch of the tree, which is not clustered with other C. botulinum type A(B) isolates from the same district. In the second outbreak, event B, five isolates from infected patients (XJFE02, XJFE03, XJFE04, XJFE05, and XJFE06) and the food isolate (XJFD26) also clustered in the same branch of the phylogenetic tree. Based on the above results, the human-derived C. botulinum strains in two food-human transmissions clustered with their food samples in the phylogeny tree, categorizing the tofu as the presumed exposure site. Quantitative analysis of SNPs was performed using only the highest quality SNPs. The public BoNT/A-producing strain ATCC 3502 was chosen as the reference to conduct a quantitative analysis of SNPs. The results showed that the clinical isolates from two events had up to 6 SNP differences compared to the reference genome, while some diversity (up to 14 SNPs) was observed among the environmental isolates (Table 1).

### Universal gene markers for tracing FB events caused by C. botulinum.

From the 59 isolates in this study and 593 published C. botulinum genomes, 329 genes were defined as universal core gene markers (Table S5). The most enriched pathways of the 329 genes involved ABC transporters, purine metabolism, and others (Fig. S2B). To evaluate the validity of these markers, a contrasting tree of the 59 isolates was constructed using the markers in this study (Fig. 1B). The three main clades of the soil isolates showed in the phylogenetic tree, and the two transmission events were traced again. Briefly, the environmental isolate (XJFD23) had a close phylogenetic relationship with an isolate from a patient (XJFE01) in event A. In the second outbreak event, an isolate from domestic stinky tofu and 5 isolates from the infected patients were aggregated. In other words, we demonstrated the validity of the universal gene markers based on public data resolution.

Moreover, in order to verify that the universal gene markers could also be applied to published C. botulinum genomes, some C. botulinum group I strains and isolates from UK and Irish cases of FB were used to characterize the outbreak events (Table S6) (28–34). Separated from other isolates, most strains in three possible botulism incidents (found under SRA accession numbers SRR8527709 and SRR8527710 [collected in 2006]; SRR8527763, SRR8527761, SRR8527762, and SRR8527766 [collected in 2011]; and SRR8527656 and SRR8527653 [collected in 2012]) formed a distinct lineage (Fig. S3).

### Gene cluster of neurotoxins, antibiotic resistance genes, and virulence factors carried by C. botulinum isolates.

**FIG 2:**
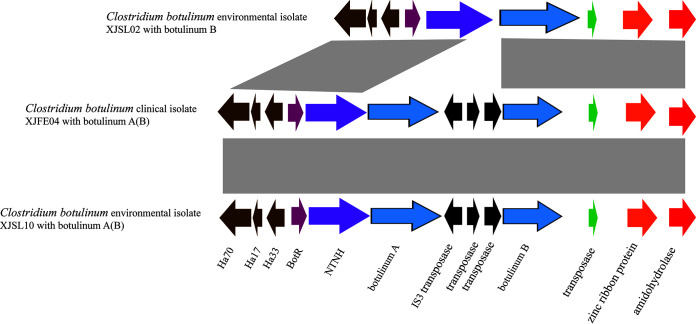
Botulinum neurotoxin-producing gene clusters of strains XJSL02, XJFE04, and XJSL10.

Botulinum neurotoxin-producing gene clusters were investigated in 59 isolates. We found that all the neurotoxins were carried on the chromosomes. Three representative strains (two isolates, one clinical and one environmental, carrying neurotoxin A(B) and one environmental isolate carrying neurotoxin B) were selected to display the gene clusters (Fig. 2). We found that the structure carrying neurotoxin A(B) was very conserved in the clinical and environmental isolates. They all had a classic ha cluster. Even the isolate carrying neurotoxin B was similar in cluster organization, except for three missing transposases.

**FIG 3:**
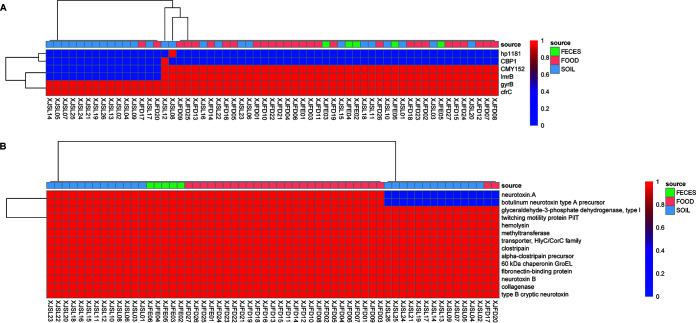
Heat maps showing the distribution of antibiotic resistance (A) and virulence-related (B) genes.

The antibiotic resistance genes and virulence factors of these isolates were explored, as well (Fig. 3). In this study, we found that the antibiotic resistance genes *gyrB* and *cfrC* were present in all isolates. In addition, over 74% of the isolates included multidrug resistance (MDR) genes such as CMY-152 and *lmrB*. The antibiotic resistance genes CBP1 and *hp1181* only existed in XJSL12 and XJSL08, respectively. We also found that these strains, isolated from soil in the same area (Ili Kazak Autonomous Prefecture) in 2018, formed an independent branch in the phylogenetic trees. All strains had the virulence-related genes *colA*, *fbp*, *groEL*, *cloSI*, CLK_2301, CLI_2965, CBO1880, CBO1450, *pilT*, and the sequence found under GenBank accession number ABM73977. Strains XJSL14, XJSL05, XJSL07, XJSL25, XJSL24, XJSL21, XJSL19, XJSL26, XJSL13, XJSL02, XJSL04, XJSL09, XJFD17, XJSL17, and XJFD20 with neurotoxin B displayed a unique gene pattern, with the absence of several MDR genes, *hp1181*, CBP1, CMY152, and *lmrB*.

## DISCUSSION

Epidemiological investigation and genomic analysis of isolates are necessary for tracing FB events. Here, we investigated two outbreaks, isolated 59 related strains, and established a tracing method using universal gene markers in the high-risk area Xinjiang Uygur Autonomous Region, China, for the first time. It has been reported that fermented tofu and soy products are the highest risk foods for botulism (22, 35). In our study, we found that the isolates of human infection were clustered together with the strains isolated from stinky tofu. FB is usually found above latitude 30°N in northern China (22). Two outbreaks in this study both occurred above latitude 42°N. There might be three causes for the frequent outbreaks in the north of China. The first reason might be due to dietary habits over the last century, as no good preserving methods for food were available (36). Meat was usually buried in the soil, generating C. botulinum reproduction under anaerobic and high protein conditions. The second reason might be related to dead animals in pasturing areas in China, which also boosts the spread of C. botulinum in the soil (37, 38). The last reason might related to the well-known low oxygen content at high latitudes (39). Hence, people should pay more attention to the processing of fermented soybean products to reduce the risk of FB.

Three types of phylogenetic markers are commonly used to construct evolutionary trees: MLST, core genome MLST (cgMLST), and genome-wide SNPs. Many C. botulinum studies have constructed evolutionary trees using these three types of phylogenetic markers (40–42). MLST has the advantage of fewer data requirements and faster tree construction. It allows rapid establishment of bacterial linkage with a large amount of phenotypic information. However, as the cost of next-generation genome sequencing decreases, more informative loci on the bacterial genome are revealed, which implies that evolutionary trees can be classified in detail. The disadvantages of the insufficient resolution of MLST and conflicting information of some phenotypes are exposed. In addition, for next-generation genome sequencing, several incomplete genome sequences make it difficult to ensure the integrity of MLST regions. Compared with MLST, cgMLST and genome-wide SNP markers are more highly recommended. The difference is that cgMLST is based on the core gene marker construction for several strains, while genome-wide SNPs are constructed using a single bacterial genome as a reference template. Theoretically, the genome-wide SNP approach contains more locus information than the MLST approach; thus, evolutionary trees constructed using the genome-wide SNP approach are more accurate. However, the prerequisite for genome-wide SNP tree construction is that the strains have no large structural variations. In contrast, cgMLST uses only the core genes of the strains for alignment, which makes the method relatively more universal. However, the number of core genes in cgMLST depends on the number of strains included, so the number of core genes used for the same species may vary depending on the strains which are included. In this research, we tried to construct an evolutionary tree using the sequenced strains in our study while integrating the publicly available strains, with the aim of making it efficient for researchers to trace C. botulinum FB events. We studied the validity of the universal gene marker-based public data resolution with the 59 isolates in this study. We also tracked three outbreaks using published genomes in a public database, using the universal gene markers we established. However, the isolation source information of the strains we tracked was missing from the published database. We can only speculate that the isolates may have come from the same outbreak based on when and where they were isolated. From the present results, the universal cgMLST results likely replicate the genealogical relationships of the evolutionary tree constructed using the strains in this study and possibly using the strains in the published database, which were restricted by limited metadata. Moreover, the phylogenetic trees constructed using newly sequenced genomes and published genomes were also used to characterize the outbreak events (see Fig. S4 in the supplemental material). Most of the strains in these two botulism incidents clustered. The results indicate that the cgMLST molecular markers we identified are highly universal and will potentially be useful for other C. botulinum evolutionary tree studies in the future.

All 59 isolates belonged to group I. Group I strains are the major cause of FB in humans (28). This classification according to whole-genomic SNPs implied that these 59 isolates share some similarity with other group I strains, which might provide a basis for core gene markers. This similarity was also found in strain ATCC 3502, which shared 63% of its coding sequences with other group I strains (43). We also screened antibiotic-resistant genes as well as virulence factors in the 59 C. botulinum isolates. We found that resistance genes were rare. There are few studies on antibiotic resistance in C. botulinum, except for one case report of a penicillin- and metronidazole-resistant strain isolated from an infant (44). This suggests that although C. botulinum originates mainly from soil and soil is the gene pool for resistance genes, C. botulinum is not selected by antibiotics. The drug resistance of the C. botulinum isolates in this study was mainly dominated by beta-lactamases, which are a common type of antibiotic commonly used in clinical treatment. Few plasmid-mediated resistance genes were detected in the resistance profile of C. botulinum, suggesting that the spread of resistance genes in C. botulinum is limited. From the point of view of virulence genes, those of C. botulinum are still mainly related to botulinum toxin, which is a neurotoxin containing polymeric protein produced by C. botulinum. It is the most toxic biotoxin known among natural toxins and synthetic agents; it mainly inhibits the release of acetylcholine from nerve endings and causes muscle relaxation paralysis, especially respiratory muscle paralysis. In this study, neurotoxins A and B were both located on the chromosome and organized in classic ha clusters, indicating that the strains are relatively conserved and stable, as found by Carter et al. (37–39). In addition, C. botulinum contains chaperonin as well as hemolysin, and most of the virulence genes are present in all strains.

No soil source was traced for the food contamination in this study. One reason might be the genetic variation during the process of long-term food fermentation, in which the spores recover and toxin is produced. The mutations may cause changes in the topology of the phylogenetic tree. Another reason might be due to inaccurate sampling of the soil. This might be resolved by a more precise food-tracing system and random soil sampling in the future.

### Conclusion.

Our study shows that these C. botulinum strains isolated in Xinjiang possessed a single type A(B) or B neurotoxin gene. Through comparative genomic analysis of 59 C. botulinum strains, foodborne botulism events caused by C. botulinum were tracked using core genes. C. botulinum strains isolated from humans and tofu in two food-human transmission events were shown in a phylogeny tree. Moreover, universal core gene markers, constructed by combining our 59 isolates and 598 public C. botulinum genomes, were successfully used to trace the three possible FB events from the published genomes.

## MATERIALS AND METHODS

### Epidemiological investigation.

Two FB events (A and B) were investigated in Xinjiang Province, China. Event A occurred in Ili Kazak Autonomous Prefecture, Xinjiang Province, in mid-May 2019. After being informed by the clinic, the local Center for Disease Control (CDC) started an epidemiological investigation by interviewing patients. According to the description, 0.5 kg of fresh tofu was placed directly into a plastic jar, without special treatment such as boiling, and fermented for 1 month at room temperature. Within 8 to 9 h of eating 20 to 30 g fermented tofu, the patient (strain XJFE01) experienced blurred vision, bucking, dizziness, dysphagia, and generalized weakness. After receiving equine double anti-A and B antitoxin, the patient improved significantly and recovered.

On 1 December 2019, five workers (strains XJFE02, XJFE03, XJFE04, XJFE05, and XJFE06) in Xinjiang’s Bayingol Mongolian Autonomous Prefecture suffered botulinum toxin poisoning (event B) after consuming homemade fermented tofu from a construction site canteen. According to the investigation, one patient purchased the tofu at a local market and made it into stinky tofu to share for consumption. The five patients recovered from botulinum toxin poisoning after treatment with botulinum toxin antiserum A + B.

### Environmental and laboratory investigation.

In events A and B, the remaining stinky tofu eaten by the patients, as well as stool samples, were collected for laboratory analysis. As soil is the main natural reservoir for C. botulinum spores, and the soybeans used to make tofu are also grown in soil, we hypothesized that the soil in Xinjiang may also be a source of contamination. Soil samples were collected not only from the outbreak area but also from other areas of Xinjiang (Table 1). In total, 59 clinical and environmental strains of C. botulinum were included in this study. The strains were recovered from a wide geographical area in Xinjiang Uygur Autonomous Region (Ili Kazak Autonomous Prefecture, Urumqi, Aksu Prefecture, Bayingol Mongolian Autonomous Prefecture, and Bortala Mongol Autonomous Prefecture) between 2018 and 2019. Food and stool samples were tested using the mouse bioassay (MBA) for neurotoxin. All samples were enriched in cooked meat and TPGY (thioglycollate-peptone-glucose-yeast extract) medium for C. botulinum isolation. The isolated strains were identified by quantitative PCR (qPCR) and confirmed by Gram staining and matrix-assisted laser desorption ionization–time of flight mass spectrometry (MALDI-TOF MS) (21).

### Library construction and whole-genome sequencing.

Genomic DNA was extracted from pure cultures using lysozyme, mutanolysin, and RNase A in Tris-EDTA (TE) buffer at 37°C for 60 min, followed by lysis in proteinase K, SDS, and NaCl for 60 min at 55°C. The DNA purity was evaluated using the NanoDrop spectrophotometer, and the DNA concentration was determined using a compact fluorimeter (Qubit 3.0; Thermo Fisher Scientific). The genomic DNA was then diluted to 0.2 ng/μL, and libraries were prepared using a Nextera XT DNA library preparation kit (Illumina, Inc., Cambridge, UK). A Qubit double-stranded DNA (dsDNA) high-sensitivity (HS) assay kit (Invitrogen) was used to determine the concentration of the sample libraries, and the libraries were pooled and sequenced using the Illumina NovaSeq 6000 instrument in 250-bp paired-end mode.

### Data preprocessing, genome assembly, gene prediction, annotation, and type determination.

Trimmomatic v0.36 was used to obtain high-quality reads. When the average Phred score fell below 20, a 4-nucleotide sliding window was used to remove nucleotides from the 3′ end. The reads were assembled using SPAdes v3.14.0 with the “–careful” option (45). QUAST v4.6 was used to conduct a quality check of the genomes (46). BUSCO v5 was used to infer the annotation completeness (47). The genome assemblies were predicted and annotated using Prokka v1.13 (28). Multilocus sequence typing (MLST) schemes were determined using the PubMLST C. botulinum database (http://pubmlst.org/cbotulinum/). Unless otherwise specified, default parameters were used for all software.

### Bioinformatics analyses.

The pangenome analysis and core genome MLST of C. botulinum were performed using Roary v3.13 with default parameters (29). In order to trace C. botulinum food-human transmission events, we established universal core gene markers using 593 public C. botulinum genomes, combined with the 59 isolates in this study. Phylogenetic trees were generated using core genome single nucleotide polymorphisms (SNPs) based on the isolates in this project combined with public strains. Phylogenetic trees were constructed using RAxML v8 (30) using the fitting model (parameter PROTGAMMAIJTTF) with default settings and visualized using iTOL (https://itol.embl.de). Snippy software was used to conduct the quantitative SNP analysis (https://github.com/tseemann/snippy). The KEGG database (http://www.kegg.jp) was used to study the core gene biological functions. The gene cluster locations of the botulinum neurotoxins were checked in this study using Platon (48). Drug resistance genes and virulence factors were identified using BLASTp (31) against the CARD (32) and VFDB (33) databases, respectively. R scripts using the packages ggplot2 and vegan in R v3.6.1 were used for statistical analysis and visualization.

### Data availability.

The sequencing data were deposited at NCBI under BioProject accession number PRJNA858251 and SRA accession numbers SRR20215563 to SRR20215621.
