# Supplementary material for: Tracing Foodborne Botulism Events Caused by Clostridium botulinum in Xinjiang Province, China, Using a Core Genome Sequence Typing Scheme
Source: Microbiol Spectr. 2022 Nov 15;10(6):e01164-22. doi: 10.1128/spectrum.01164-22 (PMC9769928; doi:10.1128/spectrum.01164-22)
Supplement: Supplemental file 1 — Fig. S1 to S4 and Tables S1 to S6. Download spectrum.01164-22-s0001.pdf, PDF file, 2.9 MB [file spectrum.01164-22-s0001.pdf]

Supplemental Material

Figure S1: Phylogenetic tree of strains in this study and public strains belonging to *C. botulinum* Groups I-III based on whole genomic SNPs. The blue circle represented Group I, the orange circle represented Group II, the pink circle represented Group III. The red type represented isolates in this study.

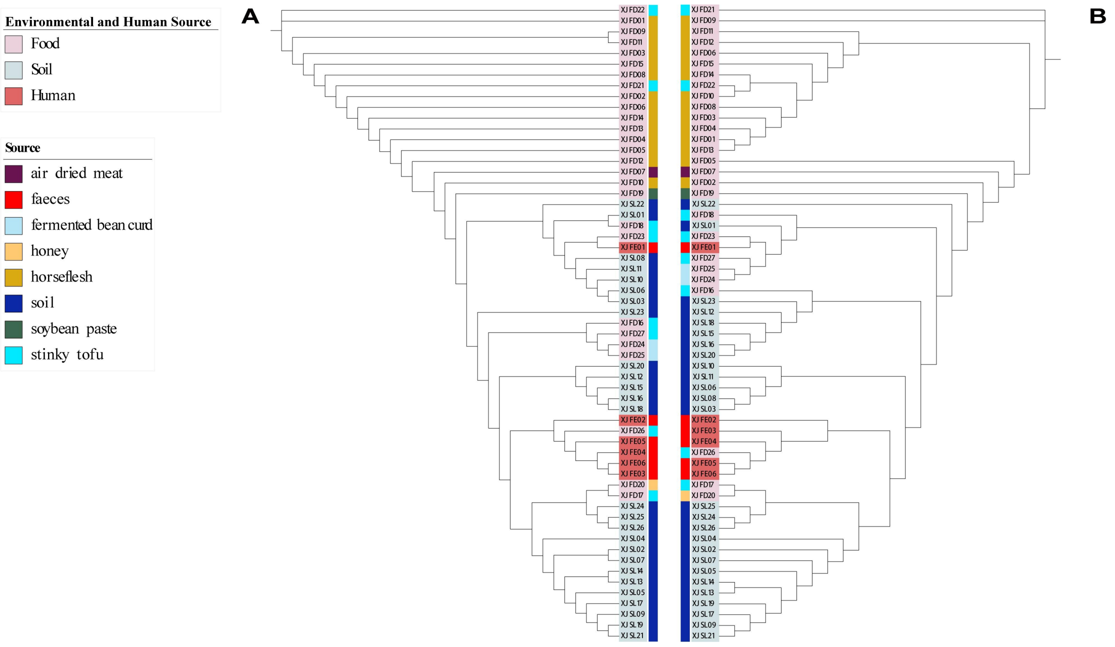

Figure S2: Functional distributions in the core gene set. (A) Function annotation of 2933 core genes. (B) Function annotation of 329 core genes.

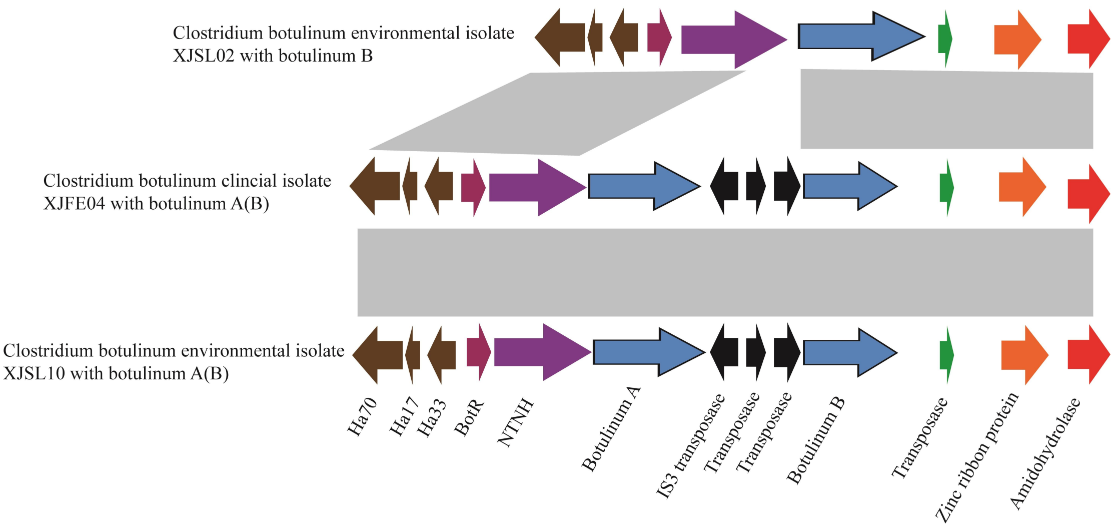

Figure S3: Phylogenetic tree of published *C. botulinum* genomes to track the

foodborne botulism outbreak events. Isolates with red label represent genomes from the outbreaks. (A). Phylogenetic tree with public isolates from the foodborne botulism outbreak event in 2006. (B). Phylogenetic tree with public isolates from the foodborne botulism outbreak event in 2011. (C). Phylogenetic tree with public isolates from the foodborne botulism outbreak event in 2012.

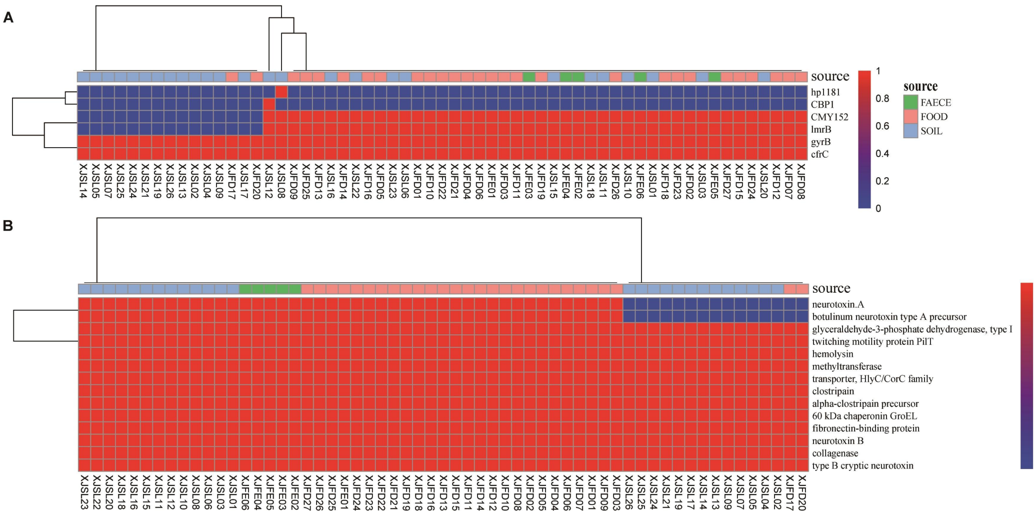

Figure S4: Phylogenetic tree with newly sequenced genomes and published genomes to track the foodborne botulism outbreak events. Isolates with red label represent clinical strains and isolates with blue label represent corresponding environmental samples of botulism outbreak in newly sequenced genomes. Isolates with brown label represent botulism outbreak strains in published genomes.

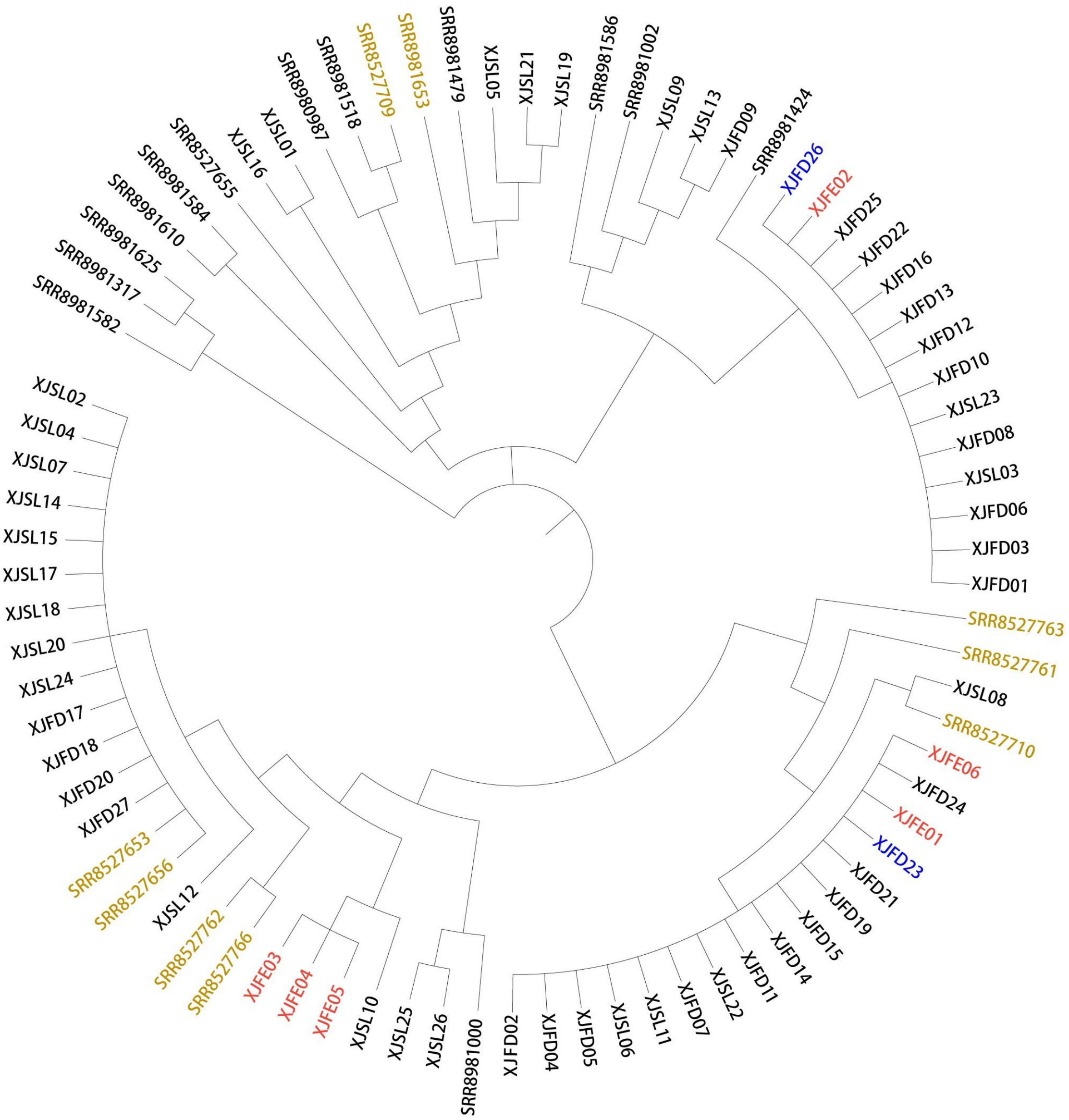
SRR8981582
SRR8981317
SRR8981625
SRR8981610
SRR8981584
SRR8527655
XJSL16
XJSL01
SRR8980987
SRR8981518
SRR8527709
SRR8981653
SRR8981479
XJSL05
XJSL21
XJSL19
SRR8981586
SRR8981002
XJSL09
XJSL13
XJFD09
SRR8981424
XJFD26
XJFE02
XJFD25
XJFD22
XJFD16
XJFD13
XJFD12
XJFD10
XJSL23
XJFD08
XJSL03
XJFD06
XJFD03
XJFD01
SRR8527763
SRR8527761
XJSL08
SRR8527710
XJFE06
XJFD24
XJFE01
XJFD23
XJFD21
XJFD19
XJFD15
XJFD14
XJFD11
XJSL22
XJFD07
XJSL11
XJSL06
XJFD05
XJFD04
XJFD02
SRR8981000
XJSL26
XJSL25
XJSL10
XJFE05
XJFE04
XJFE03
SRR8527766
SRR8527762
XJSL12
SRR8527656
SRR8527653
XJFD27
XJFD20
XJFD18
XJFD17
XJSL24
XJSL20
XJSL18
XJSL17
XJSL15
XJSL14
XJSL07
XJSL04
XJSL02

Table S1 Basic information of assembly quality based on all isolates

Assembly	# contigs	Largest contig	Total length	GC (%)	N50	N90	auN	L50	L90	# N's per 100 kbp	# contigs (>= 0 bp)	# contigs (>= 1000 bp)	# contigs (>= 5000 bp)	# contigs (>= 10000 bp)	# contigs (>= 25000 bp)	# contigs (>= 50000 bp)	Total length (>= 0 bp)	Total length (>= 1000 bp)	Total length (>= 5000 bp)	Total length (>= 10000 bp)	Total length (>= 25000 bp)	Total length (>= 50000 bp)
XJFE01	89	1061969	3825915	28	955342	296787	783028.5	2	5	4.86	89	20	10	10	7	7	3841028	3821377	3802233	3802233	3742920	3742920
XJFE02	114	1061969	3827365	28.01	956012	296787	783067.4	2	5	5.04	114	21	10	10	7	7	3848298	3824206	3802903	3802903	3743590	3743590
XJFE03	104	1061969	3828361	28.02	955342	296497	782484.8	2	5	7.65	104	19	10	10	7	7	3846033	3820746	3801943	3801943	3742630	3742630
XJFE04	8	10	38	2	9	2	78	2	5	5.07	89	19	10	10	7	7	3841	38212	38022	38022	37429	37429

	9	61	27	8	5	9	26										890	01	33	33	20	20
		96	76	.	5	6	52															
		9	3	0	3	7	.4															
				2	4	8																
					2	7																
					2																	
					1	4																
				2	7	2	15															
		21	38	8	9	0	30															
		79	28	.	0	4	86															
	7	04	79	0	4	8	7.										3841	38264	38060	38060	37467	37467
XJFE05	5	6	7	1	6	1	7	1	3	7.5	75	18	8	8	5	5	686	10	28	28	15	15
					8	1																
					1	4																
			38		6	6	71															
		95	29		5	1	37															
	8	66	64	2	9	6	88										3843	38252	38052	38052	37458	37458
XJFE06	4	27	3	8	9	2	.1	3	6	2.53	84	22	11	11	8	8	144	35	08	08	95	95
					2																	
					7	4																
				2	9	1	21															
		27	38	8	2	4	02															
	1	92	84	.	2	8	92															
	2	23	32	0	3	3	2.										3905	38770	38611	38611	38116	37685
XJFD01	1	8	5	4	8	5	9	1	3	7.52	121	16	8	8	5	4	110	90	63	63	77	92
XJFD02	8	10	38	2	9	2	78	2	5	2.56	80	21	10	10	7	7	3841	38254	38050	38050	37457	37457

	0	63	28	8	5	9	40										202	83	81	81	68	68
		98	93		6	6	97															
		5	6		6	7	.6															
					4	8																
					2	8																
					2																	
					8	4																
				2	1	1	21															
		28	38	8	0	5	23															
	1	10	95	.	3	0	14															
	2	31	53	0	1	7	9.										3916	38859	38679	38679	38312	37881
XJFD03	5	0	3	5	0	1	4	1	3	4.8	125	17	7	7	5	4	909	16	36	36	62	77
					3																	
					2	2																
				2	3	1	25															
		32	41	7	4	8	83															
	1	34	42	.	0	2	21															
	2	04	76	8	4	0	7.										4163	41331	41202	41202	40506	40075
XJFD04	5	6	1	5	6	7	7	1	3	4.76	125	15	9	9	5	4	848	77	43	43	72	87
					9	2																
				2	5	9																
		10	38	8	6	6	78															
	1	63	33	.	5	7	32															
	2	81	00	0	7	8	19										3853	38223	38051	38051	37459	37459
XJFD05	1	2	9	1	3	8	.5	2	5	0	121	19	10	10	7	7	722	72	48	48	09	09
XJFD06	1	32	38	2	3	4	27	1	2	9.94	189	18	7	7	4	3	3929	38838	38635	38635	38140	37709

	8	15	94	8	2	1	04										934	01	69	69	83	98
	9	11	14	.	1	5	57															
		8	9	0	5	3	8.															
				5	1	8	4															
					1	1																
					8																	
					8	2																
				2	1	1																
		10	40	7	5	7	75															
		63	45	.	9	2	34															
	9	81	54	8	8	1	35										4063	40403	40214	40214	39621	39621
XJFD07	9	3	6	6	2	0	.7	3	6	0.25	99	21	11	11	8	8	576	29	85	85	72	72
					2																	
					0	4																
				2	8	1	13															
		20	39	8	7	5	46															
	1	87	01	.	8	0	44															
	2	84	11	0	4	7	3.										3925	38975	38794	38794	38299	37868
XJFD08	8	6	2	3	6	0	3	1	4	4.92	128	18	9	9	6	5	198	41	02	02	16	31
					2																	
					1	4																
					7	2																
		21	38		9	0	15															
		79	30		6	3	31															
	9	67	38	2	7	6	54			10.3							3845	38239	38074	38074	37481	37481
XJFD09	0	8	3	8	8	9	6	1	3	1	90	16	8	8	5	5	521	55	53	53	40	40

XJFD10	246	1063813	3883146	28.03	956642	296787	773125.2	2	5	0.52	246	39	10	10	7	7	3934143	3857967	3805110	3805110	3745797	3745797
XJFD11	299	420354	4098077	27.96	222904	28925	222481.9	7	23	17.08	299	109	44	33	23	20	4142217	4070319	3940608	3870323	3691328	3586816
XJFD12	104	2087864	3901752	28.03	2087864	414833	1502729.2	1	3	7.3	104	18	8	8	5	4	3919940	3899439	3880107	3880107	3830621	3787536
XJFD13	103	2087864	3900191	28.01	2087864	414835	1503489.2	1	3	7.26	103	16	8	8	5	4	3918954	3897169	3880379	3880379	3830893	3787808

XJFD14	100	956857	3854163	28	799272	140528	714290.3	3	6	5.03	100	22	12	12	9	8	3871074	3848627	3828154	3828154	3768841	3728593
XJFD15	109	956857	3851096	27.96	799272	140528	714943.4	3	6	5.09	109	19	12	12	9	8	3870366	3843129	3828553	3828553	3769240	3728992
XJFD16	221	1968362	4113611	27.9	900068	140499	1212773.9	2	5	6.9	221	56	26	14	7	5	4148578	4097803	4026684	3941086	3831459	3750081
XJFD17	101	956866	3850417	27.96	799272	140528	715023.8	3	6	7.4	101	20	12	12	9	8	3868820	3843661	3828118	3828118	3768805	3728557
XJFD18	10	3233	4315	27	32	40	2506	1	3	6.72	109	16	8	8	5	4	4334075	4306132	4289084	4289084	4239598	4196513

	9	72	65	.	3	7	59															
		9	5	9	3	4	7.															
				7	7	5	8															
					2	0																
					9																	
					7	1																
				2	9	4																
			38	7	9	0	71															
		95	51	.	2	5	48															
	9	68	55	9	7	2	50										3865	38452	38282	38282	37689	37287
XJFD19	1	57	1	8	2	8	.5	3	6	7.61	91	21	12	12	9	8	968	70	99	99	86	38
					3																	
					2	4																
				2	3	1	27															
		32	39	8	3	4	27															
	1	33	04	.	4	8	87															
	0	47	12	0	7	7	7.										3921	38990	38814	38814	38319	37888
XJFD20	2	2	1	4	2	8	4	1	2	7.35	102	16	7	7	4	3	804	95	20	20	34	49
					9	2																
					5	9																
		10	38		5	6	78															
		61	26		3	7	28															
	9	96	42	2	4	8	76										3842	38208	38020	38020	37427	37427
XJFD21	4	9	8	8	2	7	.8	2	5	5.04	94	19	10	10	7	7	052	77	16	16	03	03
	1	10	38	2	9	2	78										3844	38183	38027	38027	37434	37434
XJFD22	0	62	24	7	5	9	34	2	5	7.4	109	18	10	10	7	7	999	37	17	17	04	04

	9	14	92	.	5	6	81															
		1	6	9	6	7	.1															
				9	5	8																
					4	7																
					9	2																
				2	5	9																
		10	38	8	6	6																
		63	39	.	6	7	78															
	9	98	95	0	4	8	19										3856	38344	38119	38050	37462	37462
XJFD23	7	5	5	2	3	8	39	2	5	7.55	97	23	11	10	7	7	812	12	66	46	39	39
					9	2																
					5	9																
		10	38		6	6	78															
		63	28		3	7	35															
	8	24	83	2	0	8	77										3841	38240	38041	38041	37448	37448
XJFD24	3	8	4	8	3	8	.3	2	5	2.59	83	20	10	10	7	7	945	56	94	94	81	81
					1																	
					9	1																
				2	5	4	11															
		19	38	8	7	0	90															
	1	57	49	.	9	7	60															
	6	96	58	0	6	1	2.										3877	38403	38194	38194	37610	36668
XJFD25	8	4	5	5	4	0	9	1	5	4.96	168	24	13	13	10	7	834	94	89	89	98	09
	1	19	38	2	1	1	12															
	0	57	50	8	9	4	15										3864	38450	38213	38213	37629	36686
XJFD26	3	96	32	.	5	0	72	1	5	0	103	25	12	12	9	6	962	08	41	41	50	61

		4	9	0	7	4	5.															
				6	9	2	3															
					6	2																
					4																	
					4	2																
				2	9	9																
		10	38	8	9	6	63															
	1	61	27	.	3	7	39															
	0	56	40	0	3	8	94			10.1							3843	38223	38076	38023	37430	37430
XJFD27	0	1	7	1	0	7	.1	3	6	1	100	20	13	12	9	9	429	38	81	70	57	57
					9	2																
				2	5	9																
		10	38	8	5	5	78															
	1	61	25	.	3	5	29															
	1	96	23	0	4	1	93										3845	38175	38011	38011	37418	37418
XJSL01	8	9	8	2	2	0	.4	2	5	7.42	118	18	10	10	7	7	000	64	30	30	17	17
					9	2																
				2	5	9																
		10	38	8	5	6	78															
		61	26	.	3	7	29															
	8	96	42	0	4	8	22										3839	38210	38022	38022	37429	37429
XJSL02	5	9	7	2	2	7	.5	2	5	5.07	85	21	10	10	7	7	378	70	33	33	20	20
		10	38	2	9	2	78															
		61	25	8	5	9	31															
	9	96	23	.	5	6	18										3839	38188	38020	38020	37427	37427
XJSL03	0	9	2	0	3	7	.2	2	5	5.1	90	20	10	10	7	7	860	48	16	16	03	03

XJSL04	106	1061969	3824657	128.01	42955342	87296081	783175	2	5	7.87	106	20	10	10	7	7	3843465	3818924	3801527	3801527	3742214	3742214
XJSL05	76	1061969	3822412	28	955342	296787	783743.7	2	5	5.15	76	19	10	10	7	7	3834261	3818554	3802233	3802233	3742920	3742920
XJSL06	82	1062141	3822853	28	955342	296787	783747.8	2	5	7.38	82	18	10	10	7	7	3836788	3816484	3802404	3802404	3743091	3743091
XJSL07	95	1063814	3828534	28.01	956591	296788	784134.4	2	5	2.53	95	20	10	10	7	7	3845875	3825311	3805221	3805221	3745908	3745908

XJSL08	113	2084406	3808495	28.03	2084406	330456	1334980.8	1	4	0	113	18	9	9	7	6	3827709	3802938	3784788	3784788	3748013	3714207
XJSL09	82	956627	3826541	28	816600	146162	714342	3	6	0	82	20	11	11	8	8	3839477	3820757	3805117	3805117	3745804	3745804
XJSL10	78	1061969	3822393	28	955342	296788	783747.4	2	5	5.15	78	20	10	10	7	7	3834773	3819140	3802234	3802234	3742921	3742921
XJSL11	91	2084406	3810519	28.04	2084406	330456	1335436.5	1	4	0	91	18	9	9	7	6	3823433	3803539	3788164	3788164	3751389	3717583

XJSL12	91	1062141	3824661	28.01	955342	235169	711934.1	2	6	7.37	91	20	11	11	8	8	3839220	3817664	3802595	3802595	3743282	3743282
XJSL13	89	1061969	3822627	27.99	955342	296787	783699.4	2	5	4.92	89	19	10	10	7	7	3838100	3818547	3802232	3802232	3742919	3742919
XJSL14	91	1066496	3853316	28	955677	295511	789457.7	2	5	5.09	91	21	10	10	7	7	3867957	3846731	3827857	3827857	3768499	3768499
XJSL15	126	1063853	3825444	27.99	955149	296788	784063.5	2	5	5.05	126	18	10	10	7	7	3848879	3817256	3803809	3803809	3744496	3744496
XJSL16	90	1063	3827	27	95	29	7843	2	5	2.59	90	18	10	10	7	7	3840711	3818872	3805004	3805004	3745691	3745691

		81	01	.	6	6	93															
		4	1	9	5	7	.5															
				9	9	8																
					1	8																
					9	2																
				2	5	9																
		10	38	7	6	6	78															
	1	63	27	.	5	7	43															
	0	81	43	9	9	8	07										3843	38192	38050	38050	37456	37456
XJSL17	0	4	4	9	1	8	.1	2	5	2.56	100	18	10	10	7	7	989	00	04	04	91	91
					1																	
					9	1																
				2	5	4	11															
		19	38	8	8	0	92															
		58	43	.	0	4	49															
	9	01	75	0	1	1	7.										3856	38369	38217	38217	37484	36537
XJSL18	9	5	1	3	5	5	5	1	5	0	99	23	14	14	10	7	563	00	16	16	19	79
					2																	
					2	4																
				2	0	2																
		22	38	7	4	0	15															
		04	50	.	5	4	51															
	9	59	25	9	9	1	56										3864	38451	38317	38317	37724	37724
XJSL19	7	6	2	6	6	9	6	1	3	9.74	97	16	8	8	5	5	916	92	73	73	15	15
	7	22	38	2	2	4	15										3860	38450	38316	38316	37723	37723
XJSL20	5	04	49	7	2	2	51	1	3	7.61	75	16	8	8	5	5	099	94	73	73	15	15

		596	868	.96	04596	0319	698.8															
XJSL21	74	2204696	3849341	27.95	2204696	420102	1551971.4	1	3	7.53	74	16	8	8	5	5	3858947	3845456	3831456	3831456	3772098	3772098
XJSL22	81	2204576	3850401	27.97	2204576	420419	1551483.2	1	3	9.92	81	16	8	8	5	5	3863192	3845407	3831753	3831753	3772395	3772395
XJSL23	85	2179624	3824788	27.97	2179624	420278	1533682.4	1	3	5.12	85	16	8	8	5	5	3836876	3821304	3807422	3807422	3748109	3748109

XJSL24	51	1066668	3845461	27.95	955677	296788	791924.9	2	5	4.99	51	17	10	10	7	7	3852304	3842735	3830498	3830498	3771140	3771140
XJSL25	64	772039	3844141	27.95	523931	135440	503019.9	3	8	2.58	64	24	18	16	12	10	3851808	3840199	3829045	3814380	3739982	3649931
XJSL26	97	397771	3914753	28.04	202356	53918	200820.4	7	23	0	97	48	39	37	28	24	3924988	3909525	3892748	3882349	3742757	3595477
SRR8527655	89	361553	3911345	28.02	230906	47787	196749.4	7	25	0	89	47	41	38	31	20	3920387	3906450	3895041	3877080	3742022	3294310
SRR8527656	88	3976	3914	28	14	62	1804	8	25	0	88	52	38	35	31	26	3923121	3913153	3884012	3862851	3801508	3621072

		27	24	.	0	5	20															
			9	0	4	7	.5															
				3	6	6																
					1																	
					3																	
				2	3	8																
			39	7	7	2	34															
		69	32	.	0	0	96															
	6	69	20	9	6	2	70		1								3940	39277	39111	39051	38682	38341
SRR8527709	6	71	3	8	3	6	.6	5	3	0.05	66	29	21	20	18	17	617	84	74	21	27	16
					4	1																
				2	8	0																
			39	7	0	2	43															
		69	34	.	5	5	22															
	6	79	47	9	2	9	63										3941	39296	39160	39100	38731	38390
SRR8527710	0	97	4	9	9	6	.9	4	9	0	60	25	17	16	14	13	788	76	92	39	45	34
					2																	
				2	4	8																
			40	7	4	6	26															
		52	23	.	3	6	61															
	8	71	46	8	8	0	59		1								4035	40196	40031	40031	39003	38325
SRR8527718	7	70	7	8	3	7	.8	6	7	0	87	36	27	27	21	19	934	84	14	14	37	77
			39	2	9	2	99															
	1	22	88	7	3	1	10															
	2	09	34	.	9	9	9.	1	4								3998	39844	39456	38595	35360	31339
SRR8527719	9	31	9	1	1	6	4	5	5	0	129	87	72	61	42	31	749	81	07	85	55	77

SRR8527720	249	220329	3991762	927.28	635913	710178	56646.7	30	109	0	249	192	143	109	51	13	4004548	3986562	3848840	3602155	2638145	1268455
SRR8527761	189	267613	3986236	28.17	73112	13155	103578.9	15	57	0.03	189	121	92	73	44	23	3999954	3978122	3906067	3773760	3355084	2567308
SRR8527762	104	473008	3983098	28.09	154223	48507	207781	8	25	0	104	51	40	38	31	23	3994015	3976255	3955909	3943121	3835811	3502174
SRR8527763	153	287047	4005216	28.18	73910	18318	89635.3	18	53	0	153	105	83	72	48	31	4014681	3995419	3945913	3864807	3508084	2830379
SRR8527764	131	341156	3994245	28.1	1223	3814	150248.5	10	33	0	131	71	52	45	38	26	4007119	3986763	3938933	3894895	3773113	3306311

				3	7	8																
SRR8527766	88	410776	3983951	28.08	1212200	67575	235490.3	7	18	0	88	41	31	28	24	19	3993592	3977334	3957079	3935803	3861262	3670523
SRR8980982	152	251900	3790851	28.16	55407	17756	95692	15	62	0	152	121	92	78	45	17	3798195	3787216	3709262	3618700	3069663	2025877
SRR8980983	61	1048055	4220381	27.95	711044	90850	607010.1	3	9	0	61	25	19	18	17	14	4228699	4219422	4207588	4202038	4186316	4105580
SRR8980987	183	242981	3814075	28.14	88863	19172	97399.8	14	52	0	183	97	81	71	39	25	3831739	3810317	3769233	3703930	3153806	2632494
SRR8981000	15	4523	3909	27	73	15	1233	14	59	0	150	112	89	77	43	20	3917408	3904677	3843300	3753033	3220932	2359176

	0	58	62	.	6	7	17															
			4	9	4	9	.2															
				7	4	4																
				2	9	1																
			38	8	2	9	10															
	1	23	65	.	2	5	92															
	3	08	03	0	3	3	10	1	5								3871	38589	38035	37552	32173	25944
SRR8981002	0	41	8	4	6	0	.3	2	1	0	130	98	76	69	39	22	357	55	86	47	78	34
					1																	
				2	8	3																
			41	7	6	8	27															
		73	46	.	5	0	22															
	8	58	43	8	0	1	68		2								4153	41434	41140	40892	39709	35963
SRR8981005	7	27	1	8	1	6	.1	6	5	0	87	56	43	39	32	21	954	23	73	31	33	69
				2	4	1																
			42	7	7	2	93															
	1	44	01	.	2	8	76															
	9	67	49	9	4	3	2.	2	8								4209	41989	40955	39360	33022	20904
SRR8981278	6	34	2	9	7	6	3	3	4	0	196	159	119	97	57	22	744	82	78	86	30	67
					2																	
				2	3	3																
			38	8	1	9	26															
		65	77	.	1	9	78															
	9	18	16	0	2	4	18		2								3885	38734	38428	38009	36546	32734
SRR8981312	2	99	3	7	9	6	.8	6	5	0	92	59	46	39	30	20	208	58	35	27	90	21
SRR8981317	1	28	38	2	7	2	10	1	5	0	143	98	73	69	49	27	3896	38830	38125	37888	34732	27217

	43	6883	87016	8.01	6016	3426	1944.4	5	1								380	91	25	81	60	60
SRR8981412	81	594386	4318857	27.78	313525	63613	311357.7	6	17	0	81	38	28	25	24	21	4329125	4315490	4294470	4271789	4256881	4169132
SRR8981424	102	579617	3976158	28.15	146161	32789	218852.4	7	26	0	102	57	45	43	30	21	3984781	3972611	3946396	3933942	3698249	3385673
SRR8981479	109	511479	3974318	28.11	201438	25702	225503.8	6	31	0	109	68	55	48	32	19	3982288	3970642	3938757	3888362	3618012	3183225
SRR8981518	59	807026	4137793	27.8	7361	1400	523347.1	3	10	0	59	23	17	16	14	12	4146478	4134898	4119857	4111168	4074967	4016613

SRR8981519	98	608537	4140302	827.89	72260327	1243713	283037.7	5	22	0	98	54	42	37	27	20	4150122	4137190	4109678	4068453	3896793	3647965
SRR8981561	1394	902827	4748163	28.11	356243	8292	458816.4	4	22	0	1394	161	26	19	16	14	5072054	4543146	4307234	4252228	4197808	4129444
SRR8981570	129	307242	3890189	28	101627	22921	126856.3	11	44	0	129	83	66	62	41	23	3899521	3885337	3842420	3817864	3449833	2790762
SRR8981574	232	199381	4192196	28.01	41513	11615	58723.3	30	103	0	232	186	137	112	58	20	4202100	4190012	4057287	3879853	3036153	1671892
SRR8981582	1	29	39	2	7	2	10	1	5	0	151	95	79	68	47	25	3924	39067	38636	37824	34358	26534

	5	26	13	8	4	1	38	5	1								827	08	46	49	26	29
	1	12	76	.	4	6	57															
			0	0	8	8	.8															
				1	0	5																
				2	5	1																
			38	8	6	3	72															
	2	21	87	.	9	7	14															
	1	47	14	0	7	2	0.	2	7								3902	38805	37804	36555	31505	22482
SRR8981584	9	52	1	2	6	8	3	0	1	0	219	140	101	84	52	25	427	46	20	84	61	82
				2	6	1																
			39	8	2	5	67															
	1	14	62	.	1	9	60															
	6	41	60	0	4	8	2.	2	6								3969	39547	38556	37801	32515	22420
SRR8981586	5	51	8	2	5	6	4	1	9	0	165	130	96	85	53	25	039	16	65	11	72	89
					3																	
				2	5	7																
			40	7	6	7	31															
		53	62	.	2	9	91															
	7	72	24	9	4	4	87		1								4071	40604	40378	40236	39752	39173
SRR8981599	7	20	0	2	3	5	.1	5	3	0	77	34	24	22	19	17	878	05	09	10	42	94
					4	1																
				2	0	4																
			38	8	9	0	44															
		91	94	.	7	3	47															
	9	82	51	0	4	5	70		1								3909	38910	38663	38510	38005	37031
SRR8981610	9	90	6	2	5	5	.8	4	0	0	99	32	20	18	15	12	862	93	69	64	41	78

SRR8981625	136	272939	4066515	28.06	107008	16184	112129.1	13	51	0	136	113	82	67	39	24	4071839	4063011	3971773	3869360	3431766	2889061
SRR8981642	157	433471	4211203	27.98	79886	16252	125087.7	14	60	0	157	116	92	78	49	22	4219133	4207702	4140603	4032023	3584595	2600763
SRR8981648	73	639567	3974098	28.1	284610	64353	314355.7	5	17	0	73	39	29	27	22	19	3981384	3972249	3950935	3935646	3854808	3745989
SRR8981653	228	257573	3959573	28.14	78160	12852	88781.8	16	64	0	228	133	96	79	42	24	3976969	3952837	3860173	3739150	3184543	2539995
SRR8981656	108	361847	3975672	28.1	1296	2732	155230.8	9	34	0.05	108	74	57	52	36	27	3982274	3972538	3930274	3902486	3652513	3350997

GCA_000017025.1_ASM1702v1_genomic	1	3863450	3863450	28.21	593863450	3 3863450	3863450	1	1	0	1	1	1	1	1	1	3863450	3863450	3863450	3863450	3863450	3863450
GCA_000017045.1_ASM1704v1_genomic	1	3760560	3760560	28.18	3760560	3760560	3760560	1	1	0	1	1	1	1	1	1	3760560	3760560	3760560	3760560	3760560	3760560
GCA_000017065.1_ASM1706v1_genomic	2	3995387	4012918	28.3	3995387	3995387	3978009.2	1	1	0.25	2	2	2	2	1	1	4012918	4012918	4012918	4012918	3995387	3995387
GCA_000019305.1_ASM1930v1_genomic	2	395823	410701	28.	395	395	382023	1	1	0	2	2	2	2	2	2	4107013	4107013	4107013	4107013	4107013	4107013

		3	3	23	8233	8233	2.4															
GCA_000019545.1_ASM1954v1_genomic	2	3992906	4259691	28.14	3992906	3992906	3759538.6	1	1	0	2	2	2	2	2	2	4259691	4259691	4259691	4259691	4259691	4259691
GCA_000020345.1_ASM2034v1_genomic	3	3977794	4257769	28.04	3977794	3977794	3733376.8	1	1	0	3	3	3	2	2	2	4257769	4257769	4257769	4247816	4247816	4247816
GCA_000022765.1_ASM2276v1_genomic	1	4155278	4155278	28.21	4155278	4155278	4155278	1	1	0	1	1	1	1	1	1	4155278	4155278	4155278	4155278	4155278	4155278
GCA_0000635	2	38	39	2	3	3	38	1	1	0	2	2	2	2	1	1	3903	39032	39032	39032	38869	38869

85.1_ASM6358		86	03	8	8	8	70										260	60	60	60	16	16
v1_genomic		91	26	.	8	8	70															
		6	0	2	6	6	8.															
				4	9	9	9															
					1	1																
					6	6																
					3	3																
					9	9																
				2	9	9	39															
		39	40	8	3	3	75															
GCA_0000923		93	10	.	0	0	70															
45.1_ASM9234		08	61	2	8	8	5.										4010	40106	40106	40106	39930	39930
v1_genomic	2	3	4	9	3	3	3	1	1	0.27	2	2	2	2	1	1	614	14	14	14	83	83
					4	2																
				2	3	0																
		14	40	8	3	2	71															
GCA_0001710		31	30	.	5	5	24															
55.1_ASM1710	4	30	94	4	0	5	91										4031	40258	39883	39813	39200	38803
5v1_genomic	8	7	9	7	1	4	.8	3	8	0	49	42	15	14	10	9	357	97	53	98	95	55
					1																	
				2	7	3																
			42	8	0	9	17															
GCA_0001710		32	17	.	3	2	28															
75.1_ASM1710	7	78	75	2	1	1	58		2								4217	42140	41918	41520	39437	36857
7v1_genomic	0	05	4	3	5	5	.1	9	7	0	70	66	52	47	31	24	754	95	78	78	92	18
GCA_0002531	1	39	39	2	3	3	39	1	1	0	1	1	1	1	1	1	3919	39197	39197	39197	39197	39197

95.1_ASM25319v1_genomic		19740	19740	8.21	919740	919740	19740										740	40	40	40	40	40
GCA_000307635.1_ASM30763v1_genomic	1740	44330	4075366	28.14	4028	918	6053.8	271	1133	0	1743	1062	189	49	3	0	4076805	3600283	1671736	720635	98878	0
GCA_000307655.2_CFSAN0001628_2.0_genomic	246	199101	4013630	27.98	36032	7524	49099.4	32	132	0	246	239	158	109	45	15	4013630	4008542	3783625	3415619	2428916	1305664
GCA_000439615.1_CFSAN002367_1.0_genomic	161	176269	3872496	27.99	41523	13061	59389.2	28	91	0	164	157	122	106	52	15	3873962	3869681	3771535	3662796	2782324	1385718
GCA_000439635.1_CFSAN002368_1.0_genomic	234	121302	3848300	27.95	31642	7641	39269.8	37	133	0	238	228	166	110	47	14	3850187	3843658	3671598	3266558	2212872	1066091

GCA_000439655.1_CFSAN002369_1.0_genomic	129	161786	3645064	27.96	52024	15175	58737	23	72	0	130	124	103	84	54	26	3645546	3641193	3586450	3451938	2961811	1978896
GCA_000439815.2_ASM43981v2_genomic	36	2040448	4083414	27.92	946263	140081	1286655.6	2	6	0	43	19	12	10	9	8	4086083	4071535	4049933	4035199	4011530	3982605
GCA_000503815.1_ASM50381v1_genomic	3	4004744	4408349	27.54	4004744	4004744	3656690.5	1	1	639.15	3	3	3	3	3	3	4408349	4408349	4408349	4408349	4408349	4408349
GCA_000582375.1_CDC54075.1_genomic	540	61472	4255473	27.89	13282	3741	16142.3	97	324	1.36	542	511	273	138	24	1	4256146	4232572	3609509	2611636	802682	61472
GCA_000582415.1_CDC54088.1_genomic	469	112584	386810	28.	129	375	16875.	95	310	1.58	469	466	253	133	18	1	3868107	3865505	3234922	2374171	628875	112584

GCA_000582435.1_CDC54085.1_genomic	345	101478	74095763	1928.02	4719805	05544	427775.3	57	204	0.98	345	338	214	132	42	10	4095763	4090304	3739315	3137914	1727164	684262
GCA_000582455.1_CDC54091.1_genomic	721	68031	4079046	28.11	84782	2547	11070.2	145	485	1.86	721	702	280	107	7	1	4079046	4062439	2931896	1697798	242151	68031
GCA_000710975.1_B2_331_genomic	10	2732590	3809103	27.97	7325902	424576	2066140.2	1	3	0	10	10	6	5	5	5	3809103	3809103	3801025	3792025	3792025	3792025
GCA_000710985.1_B2_275_genomic	13	2203691	3978188	28.02	203691	141597	1403295.6	1	5	0	13	13	9	8	8	8	3978188	3978188	3969866	3960759	3960759	3960759

GCA_000710995.1_A2B3_87_genomic	13	2767020	4168550	27.86	2767020	232419	1940332.6	1	4	0	13	13	9	8	8	6	4168550	4168550	4161107	4152139	4152139	4078334
GCA_000711005.1_A2B7_92_genomic	14	1849744	4057812	27.93	698075	178792	1072280	2	6	0	14	14	10	9	9	8	4057812	4057812	4050317	4041404	4041404	4012834
GCA_000711055.1_A2_117_genomic	11	2752809	3808262	28.01	2752809	299394	2074858.9	1	3	0.03	11	11	7	6	6	5	3808262	3808262	3800868	3791717	3791717	3763994
GCA_000711065.1_B2_128_genomic	10	2786970	3844467	27.98	278697	427487	2126055	1	3	0	10	10	6	5	5	4	3844467	3844467	3835967	3826666	3826666	3798124

GCA_000711095.1_B2_267_genomic	12	2837736	3903580	27.96	02837736	420552	2161554.6	1	3	0	12	12	8	7	5	4	3903580	3903580	3895086	3885710	3853238	3824698
GCA_000711105.1_F_357_genomic	15	1045218	3832122	28.03	957457	129790	741989.3	2	6	0.03	15	15	11	10	9	8	3832122	3832122	3824403	3815223	3797755	3768953
GCA_000711115.1_B2_433_genomic	12	2803320	4124526	27.85	2803320	266695	2017898.1	1	4	0	12	12	8	7	6	5	4124526	4124526	4116026	4106754	4095161	4066619
GCA_000730705.1_ASM73070v1_genomic	388	81742	3900773	28.18	18419	4470	24172	61	223	0.36	389	367	206	133	35	9	3901135	3884208	3435338	2920188	1411601	552608

GCA_000730715.1_ASM73071v1_genomic	304	123950	3975522	28.13	26132	6144	33243	44	167	0.78	310	291	188	123	45	10	3977994	3966385	3696838	3231460	2017014	735358
GCA_000730725.1_ASM73072v1_genomic	337	98730	3964859	28.05	22262	5453	28773.1	51	180	0.53	341	313	190	128	44	8	3966737	3945647	3622641	3175614	1831906	529064
GCA_000730735.1_ASM73073v1_genomic	867	46145	3683802	28.54	6353	1911	8090.6	172	592	0.54	869	822	232	75	3	0	3684733	3647968	2183170	1069711	97135	0
GCA_000730785.1_ASM73078v1_genomic	418	78034	3960455	28.21	16985	4397	21825.7	70	241	0.43	418	394	218	133	33	4	3960455	3941613	3458202	2848389	1256813	267460
GCA_000730795.1_ASM73079v1_genomic	399	100640	3890459	28.19	16863	4566	24198	63	233	0.36	399	385	215	125	31	7	3890459	3879577	3417081	2772694	1292298	483242
GCA_0007308	5	62	38	2	1	3	15	9	3	0.52	524	505	249	120	20	4	3868	38548	31653	22393	71488	22040

05.1_ASM7308	2	80	68	8	1	3	99	3	2								980	14	28	00	0	5
0v1_genomic	3	4	56	.	9	8	5.		8													
			1	2	8	3	4															
				3	4																	
				2																		
	1		38	8	5	1																
GCA_0007308	0	28	22	.	6	6	70	2	6													
35.1_ASM7308	6	28	98	5	1	6	02	0	9								3833	36924	21150	95488		
3v1_genomic	2	0	5	4	5	1	.7	6	7	1.02	1086	885	244	71	3	0	726	37	07	4	82795	0
				2																		
	1		34	8	4	1																
GCA_0007308	0	18	43	.	1	5	50	2	7													
65.1_ASM7308	9	45	79	9	1	4	93	6	9								3444	33829	13451	36911		
6v1_genomic	1	8	3	2	5	3	.7	0	5	2.24	1093	1017	176	29	0	0	700	64	89	1	0	0
				2	1																	
			39	8	9	4	22															
GCA_0007308	3	67	64	.	0	5	65		2													
75.1_ASM7308	9	27	65	1	8	3	6.	6	2								3965	39465	35029	29529	14570	34343
7v1_genomic	9	9	1	3	6	9	1	3	4	0.53	400	376	210	133	39	6	132	10	11	55	23	5
				2																		
			36	8	6	1																
GCA_0007308	8	38	95	.	1	9	82	1	5													
85.1_ASM7308	6	19	07	5	9	0	98	7	9								3699	36496	21589	11420	15175	
8v1_genomic	9	4	3	7	6	3	.9	0	3	1.08	880	814	225	79	5	0	887	41	19	13	4	0
GCA_0007309	4	78	38	2	1	4	18	8	2								3898	38819	33024	26114	10018	24508
25.1_ASM7309	5	15	97	8	4	0	45	1	7	0.46	456	433	231	135	29	4	725	26	72	26	79	6

2v1_genomic	4	5	77	.	5	0	6.		7													
			8	2	4	9	7															
				1	9																	
				2																		
	1		35	8	4	1																
GCA_0007309	0	26	55	.	4	6	54	2	7													
45.1_ASM7309	7	45	26	9	3	4	67	5	8								3557	34998	14708	44758		
4v1_genomic	3	8	4	2	4	7	.8	7	0	2.22	1079	1003	191	33	1	0	862	63	57	1	26458	0
				2																		
	1		37	8	5	1																
GCA_0007309	0	28	28	.	0	6	67	2	7													
55.1_ASM7309	3	32	84	7	1	6	99	1	1								3731	36382	18719	79748		
5v1_genomic	9	3	1	6	8	7	.3	3	2	1.13	1046	923	214	56	2	0	781	44	85	9	54415	0
				2																		
			38	8	9	2	12															
GCA_0007309	6	45	88	.	3	6	21	1	4													
65.1_ASM7309	7	96	70	2	7	4	3.	2	1								3893	38456	29006	18760	37162	
6v1_genomic	3	7	5	2	4	0	6	0	5	0.33	684	618	257	112	11	0	295	97	23	91	5	0
				2	5	2																
GCA_0007694			40	7	9	0	68															
95.1_CFSAN02	1	18	05	.	8	0	28															
4410_01.0_ge	3	71	12	9	0	5	4.	2	6								4005	40017	39315	38689	33519	24836
nomic	1	09	8	2	4	6	5	2	8	0	131	126	95	86	56	31	128	64	74	95	47	31
GCA_0008169		38	38	2	3	3	38															
45.1_ASM8169		92	92	8	8	8	92										3892	38920	38920	38920	38920	38920
4v1_genomic	1	02	02	.	9	9	02	1	1	0	1	1	1	1	1	1	029	29	29	29	29	29

		9	9	03	2029	2029	9															
GCA_000816965.1_ASM81696v1_genomic	2	4077214	4334551	27.88	4077214	4077214	3850432.6	1	1	2.31	2	2	2	2	2	2	4334551	4334551	4334551	4334551	4334551	4334551
GCA_000817935.1_ASM81793v1_genomic	2	4089683	4365669	28	4089683	4089683	3848591.2	1	1	0	2	2	2	2	2	2	4365669	4365669	4365669	4365669	4365669	4365669
GCA_000829015.1_ASM82901v1_genomic	1	3901300	3901300	28.27	3901300	3901300	3901300	1	1	0	1	1	1	1	1	1	3901300	3901300	3901300	3901300	3901300	3901300
GCA_0008307	7	33	39	2	1	3	14	1	3	19.9	90	67	48	45	35	26	3938	39252	38820	38596	36910	33378

55.1_ASM8307	8	51	32	7	3	9	50	0	1	3							115	32	43	74	05	42
5v1_genomic		01	96	.	3	2	22															
			0	9	1	0	.1															
				5	3	7																
					5																	
					2																	
					9	4																
				2	1	3	20															
		29	43	7	6	5	99															
GCA_0008764		16	20	.	9	5	07															
95.2_ASM8764	1	97	66	7	7	0	5.										4320	43206	43148	43059	43059	42772
9v2_genomic	0	2	9	5	2	7	9	1	3	0	10	10	7	6	6	5	669	69	44	71	71	80
				2	3																	
			42	7	1	8	47															
GCA_0009652	2	14	84	.	3	0	67		1													
95.1_ASM9652	6	26	14	7	9	1	2.	3	3								4291	42757	40354	37465	25221	13111
9v1_genomic	5	14	0	8	5	5	4	6	2	0	287	253	159	119	49	14	470	28	91	90	62	22
				2																		
			38	8	7	1																
GCA_0009653	8	38	36	.	4	9	92	1	5													
25.1_ASM9653	8	37	47	2	4	9	38	5	6								3864	37441	24072	14050	12905	
2v1_genomic	5	6	6	5	9	3	.1	2	2	0	962	762	234	92	4	0	878	82	78	62	0	0
	1		37	2	3	1																
GCA_0009653	4	26	23	8	8	1	52	2	9													
45.1_ASM9653	4	29	94	.	0	5	54	8	8								3810	34586	13723	46917		
4v1_genomic	1	6	8	6	0	3	.6	1	1	0	1680	1080	168	33	2	0	828	12	76	3	51305	0

GCA_000965375.1_ASM965377v1_genomic	178	188959	3883299	428.02	42026	11782	52354.5	29	94	0	189	168	126	104	54	22	3886945	3875640	3763771	3608695	2787848	1656838
GCA_000965385.1_ASM965388v1_genomic	189	137732	3885155	28	50992	10559	52844.8	26	96	0	198	181	125	99	49	28	3888335	3879682	3723826	3538250	2757055	2060889
GCA_000965445.1_ASM965447v1_genomic	237	112275	3788682	28.07	30380	7790	40392.2	35	126	0	248	225	151	110	47	14	3792223	3780120	3565091	3273649	2240026	1097311
GCA_000965465.1_ASM965466v1_genomic	784	47145	4182051	28.04	9380	2442	11441.9	135	486	0	856	704	271	117	9	0	4207455	4119771	3016336	1916664	298827	0
GCA_000965505.1_ASM965511v1_genomic	511	90027	4232622	27.85	19719	3974	25930.9	63	251	0	570	431	214	127	32	9	4252259	4173777	3647225	3035483	1446909	621564

GCA_000965515.1_ASM96551v1_genomic	731	72565	4058094	28.1	9229	2539	12995.5	127	449	0	798	658	264	110	13	2	4081847	4004126	2977805	1872395	476634	127683
GCA_000986925.1_CBOT001-SEQ-1-ASM-1_genomic	21	1292763	3976482	28.1	835326	145896	801580.2	2	6	0	27	17	10	10	9	8	3978537	3973568	3958265	3958265	3940607	3910549
GCA_001273165.1_CBOT002-SEQ-1-ASM-1_genomic	20	2083105	3905320	28.02	2083105	143181	1335673	1	5	0	24	18	8	8	8	7	3906754	3903895	3877054	3877054	3877054	3848236
GCA_001273255.1_CBOT006-SEQ-1-ASM-1_genomic	45	500640	3903644	28.01	227875	50288	259440.3	6	18	0	56	39	30	29	25	18	3907623	3899516	3879462	3874231	3805466	3548923
GCA_001273275.1_CBOT007-SEQ-1-ASM-	26	978749	390591	28.	612	138	558122	3	9	0	35	24	14	14	14	12	3909427	3904710	3877732	3877732	3877732	3809556

1_genomic			1	0	7	2																
				2	9	3																
					2	5																
					7	1																
				2	2	4																
GCA_0012769			38	7	0	0																
85.1_CBOT009		93	87	.	4	3	61															
-SEQ-1-ASM-	2	55	69	9	2	8	45										3889	38847	38654	38654	38551	38204
1_genomic	4	45	0	8	3	0	27	3	7	0	28	20	12	12	11	10	092	88	00	00	79	06
					1																	
				2	4	3																
			39	7	2	5	19															
GCA_0015731		45	59	.	9	1	54															
75.1_10148_g	7	79	49	9	8	7	79		3								3959	39566	39175	38821	36716	33026
enomic	1	87	5	3	5	0	.6	8	0	0	71	67	50	45	33	23	495	29	61	27	44	04
					1																	
					1	2																
			38		6	6																
GCA_0015732		40	98		1	1	15															
35.1_ASM1573	7	91	54	2	1	9	22	1	3								3898	38970	38452	38090	35868	30315
23v1_genomic	9	53	6	8	5	6	24	0	6	0	79	77	58	53	39	23	546	74	00	10	64	93
				2	1	3																
			42	7	1	8																
GCA_0015732		37	01	.	7	1	15															
55.1_ASM1573	7	11	46	9	4	1	52	1	3								4201	41978	41627	41544	39575	35843
25v1_genomic	0	27	0	1	4	8	96	1	3	0	70	65	51	50	38	28	460	66	26	72	33	43

GCA_001573295.1_ASM157329v1_genomic	80	184098	3864510	27.98	877273	31064	93862	16	43	0	80	76	63	60	47	34	3864510	3861880	3827618	3804733	3610557	3153893
GCA_001573315.1_ASM157331v1_genomic	65	444784	3777532	27.99	137365	35127	159807	10	31	0	65	63	50	48	33	23	3777532	3776183	3744429	3728989	3478123	3104462
GCA_001573325.1_ASM157332v1_genomic	99	217785	4002127	27.98	78163	24042	94169.6	16	53	0	99	95	80	71	50	28	4002127	3999379	3964442	3899108	3546421	2752386
GCA_001573335.1_ASM157333v1_genomic	86	296419	4025192	27.94	97924	24367	127362.3	11	44	0	86	83	69	59	43	25	4025192	4023185	3990072	3912655	3622491	2994650
GCA_001573375.1_ASM157337v1_genomic	84	358807	3971034	27.9	1016	2405	133975.3	11	42	0	84	83	69	58	41	26	3971034	3970504	3935834	3844492	3573162	3056737

				8	6	2																
GCA_001573385.1_ASM157338v1_genomic	55	467882	3891395	28.03	4199059	47716	213932.8	7	25	0	55	55	40	37	30	24	3891395	3891395	3854094	3832830	3718458	3486000
GCA_001573395.1_ASM157339v1_genomic	55	483576	3909258	27.97	191646	37872	213682	7	24	0	55	53	41	38	29	21	3909258	3907907	3880964	3861325	3699340	3430768
GCA_001573435.1_ASM157343v1_genomic	74	343034	3942683	27.97	116912	33758	150269.5	10	35	0	74	69	55	52	39	25	3942683	3938738	3896972	3876447	3667592	3161781
GCA_001573455.1_ASM157345v1_genomic	42	717550	3880168	27.96	252959	50276	302720	5	20	0	42	41	31	31	27	20	3880168	3879446	3854886	3854886	3781898	3534385

GCA_001573465.1_ASM157346v1_genomic	34	843203	3791338	28.01	344362	65746	366461.1	4	15	0	34	34	24	24	23	17	3791338	3791338	3767140	3767140	3745201	3530481
GCA_001573485.1_ASM157348v1_genomic	64	277872	3812159	27.9	175053	36310	152419.2	9	28	0	65	59	47	43	31	23	3812410	3808085	3779388	3752803	3549804	3267832
GCA_001573515.1_ASM157351v1_genomic	48	536409	3856611	28.02	219996	52695	239616.7	6	22	0	48	46	34	34	30	22	3856611	3855235	3824249	3824249	3759837	3491890
GCA_001573535.1_ASM157353v1_genomic	98	235902	3896775	27.99	72415	20392	98098.2	15	50	0	98	96	75	69	46	28	3896775	3895598	3844663	3802982	3438733	2775245
GCA_001573555.1_ASM157355v1_genomic	97	226419	392028	27.	967	240	102889	13	50	0	97	93	74	68	47	21	3920281	3917385	3871865	3824722	3466542	2534113

			1	95	84	74	.8														
GCA_001573665.1_ASM157366v1_genomic	65	288740	4008651	28.07	119519	35806	141308.5	10	35	0	65	63	54	50	41	25	4008651	4007056	3986365	3958820	3798517	3226089
GCA_001573725.1_ASM157372v1_genomic	34	866701	3900045	27.99	410269	78862	479352.5	3	12	0	34	31	20	20	19	15	3900045	3898199	3872070	3872070	3861899	3718204
GCA_001573825.1_ASM157382v1_genomic	84	259440	3869437	28.03	100079	35938	107824.9	13	39	0	84	77	59	56	44	26	3869437	3865562	3822876	3800526	3626234	2914511
GCA_001573885.1_ASM157388v1_genomic	85	1034246	4285737	27.94	214161	48919	395669.3	5	20	0	85	67	32	29	26	19	4285737	4272453	4195741	4177462	4121637	3844167

GCA_001573905.1_ASM157390v1_genomic	66	301217	4267307	27.88	146794	61862	157442.2	11	27	0	66	56	39	39	32	28	4267307	4261506	4224990	4224990	4099609	3950579
GCA_001573935.1_ASM157393v1_genomic	38	689166	3873577	28.1	357116	91369	357157.4	4	13	0	38	33	20	19	18	16	3873577	3870165	3838857	3833841	3812916	3740969
GCA_001573955.1_ASM157395v1_genomic	50	587178	4230750	27.87	274519	76307	282869.7	6	17	0	50	44	25	25	23	19	4230750	4226274	4188567	4188567	4154801	3991960
GCA_001573965.1_ASM157396v1_genomic	61	658338	4274979	27.88	215880	62985	340998.1	5	18	0	61	50	28	28	26	18	4274979	4267501	4223430	4223430	4197208	3902790
GCA_001573985.1_ASM1573	67	9469	4010	28	23	62	3975	4	17	0	67	44	26	25	24	20	4010957	3996648	3960841	3954647	3939188	3810683

98v1_genomic		46	95	.	8	9	29															
			7	0	5	8	.7															
				5	2	5																
					8																	
					1																	
				2	5	1																
			43	8	0	1	24															
GCA_0015740	2	60	05	.	8	0	44															
15.1_ASM1574	8	94	63	0	5	1	85		3								4305	42169	40145	39090	37216	35041
01v1_genomic	0	91	3	3	0	2	.1	7	8	0	280	150	56	41	27	21	633	45	63	52	44	00
					5																	
				2	2	9																
		15	39	8	9	4	73															
GCA_0015740		07	90	.	9	0	29															
35.1_ASM1574	3	07	63	0	4	8	62		1								3990	39876	39582	39516	39362	38694
03v1_genomic	8	6	2	4	2	0	.2	2	0	0	38	33	18	17	16	14	632	34	00	71	12	99
					2																	
				2	2	5																
			43	7	2	2	28															
GCA_0015740	1	52	06	.	9	6	22															
55.1_ASM1574	4	99	76	8	0	7	92		1								4306	42652	41716	41537	40712	39090
05v1_genomic	1	62	0	7	1	4	.6	6	9	0	141	79	32	29	24	19	760	46	33	62	84	26
		18	41		7	7	97															
GCA_0015740	1	07	75		2	8	20															
65.1_ASM1574	7	05	40	2	3	9	57										4175	40999	40256	40256	39919	38817
06v1_genomic	4	7	4	8	2	3	.1	2	9	0	174	60	16	16	14	11	404	73	53	53	27	90

GCA_001574095.1_ASM157409v1_genomic	223	836975	4476519	27.92	57277699	127817	348008.5	5	19	0	223	141	40	26	19	16	4476519	4416618	4223302	4134586	4041684	3952545
GCA_001574115.1_ASM157411v1_genomic	99	941389	4311895	27.88	274519	85640	367737.9	5	17	0	99	51	24	23	22	19	4311895	4279120	4231424	4224715	4205952	4084112
GCA_001574125.1_ASM157412v1_genomic	66	941177	4281988	27.87	274519	96878	422555.8	4	14	0	66	43	21	21	20	17	4281988	4266377	4226999	4226999	4211540	4101748
GCA_001574135.1_ASM157413v1_genomic	139	679127	4368910	27.9	424584	52678	374125.7	4	17	0	139	86	27	25	23	17	4368910	4331571	4209253	4194939	4156649	3941851

GCA_001574175.1_ASM157417v1_genomic	204	506477	4495542	27.88	197058	45723	216843	8	23	0	204	118	32	31	28	22	4495542	4433808	4278758	4273642	4223234	4018133
GCA_001574195.1_ASM157419v1_genomic	61	581756	4055427	28	429106	91248	356364.3	4	13	0	61	50	21	20	18	16	4055427	4047935	3985287	3975553	3949576	3877433
GCA_001574205.1_ASM157420v1_genomic	46	1142320	3929155	28.04	460210	92591	542973.1	3	11	0	46	38	17	16	15	14	3929155	3924226	3884896	3878072	3862613	3834073
GCA_001574225.1_ASM157422v1_genomic	272	307541	4534970	28.07	140479	13641	144444.3	11	43	0	272	161	61	54	37	26	4534970	4455506	4273493	4226188	3981764	3603275
GCA_001574255.1_ASM1574	12	3908	4210	28	11	29	1557	10	38	0	124	94	56	52	41	25	4210622	4189229	4106999	4079198	3883248	3302251

25v1_genomic	4	20	62	.	7	8	14															
			2	3	7	1	.2															
				3	9	3																
					2																	
					2																	
				2	4	6																
			40	8	9	2	25															
GCA_0015742		58	91	.	8	9	41															
65.1_ASM1574	6	17	98	0	5	8	78		2								4091	40852	40310	40054	39794	37889
26v1_genomic	8	56	8	1	2	5	.2	6	0	0	68	58	31	28	26	21	988	28	61	23	46	25
					2																	
				2	7	5																
			41	8	4	5	32															
GCA_0015742		62	36	.	5	0	68															
85.1_ASM1574	6	70	38	0	1	3	21		1								4136	41305	40836	40674	39956	37668
28v1_genomic	1	51	4	4	9	5	.9	5	7	0	61	53	29	27	23	17	384	43	41	93	03	25
					3																	
				2	7	9																
			42	7	2	2	36															
GCA_0015743		74	96	.	3	4	84															
15.1_ASM1574	5	77	97	8	8	0	68		1								4296	42924	42464	42331	42176	41891
31v1_genomic	1	76	7	6	5	2	.8	5	4	0	51	44	21	19	18	17	977	17	06	00	41	01
			38	2	2	5	24															
GCA_0015743		49	82	7	0	5	84															
35.1_ASM1574	4	91	85	.	1	7	04		1								3882	38816	38577	38577	37580	35758
33v1_genomic	3	33	2	9	0	6	.2	6	9	0	43	41	30	30	25	20	852	94	51	51	98	32

GCA_001574355.1_ASM157435v1_genomic	72	519288	3929760	527.93	43270392	963596	269531.2	6	16	0	72	43	26	25	23	17	3929760	3912129	3876759	3871695	3825896	3624298
GCA_001574365.1_ASM157436v1_genomic	137	287793	3945914	27.98	64297	15544	97294.5	16	66	0	137	120	97	82	46	22	3945914	3935255	3874171	3755750	3172998	2316680
GCA_001879605.1_ASM187960v1_genomic	1	4089027	4089027	28.14	4089027	4089027	4089027	1	1	0	1	1	1	1	1	1	4089027	4089027	4089027	4089027	4089027	4089027
GCA_001879625.1_ASM187962v1_genomic	3	3954777	4020063	28.2	395477	395477	3891436.5	1	1	0	3	3	3	2	2	2	4020063	4020063	4020063	4014137	4014137	4014137

GCA_001889345.1_ASM188934v1_genomic	1	3867627	3867627	28.24	73867627	73867627	3867627	1	1	0	1	1	1	1	1	1	3867627	3867627	3867627	3867627	3867627	3867627
GCA_001889365.1_ASM188936v1_genomic	2	4235583	4432564	28.1	4235583	4235583	4056109.5	1	1	0	2	2	2	2	2	2	4432564	4432564	4432564	4432564	4432564	4432564
GCA_001921905.1_ASM192190v1_genomic	2	3957628	3967717	28.19	3957628	3957628	3947590.3	1	1	0	2	2	2	2	1	1	3967717	3967717	3967717	3967717	3957628	3957628
GCA_001921925.1_ASM192192v1_genomic	2	4217365	4414346	28.1	4217	4217	4037963.	1	1	0	2	2	2	2	2	2	4414346	4414346	4414346	4414346	4414346	4414346

GCA_001921945.1_ASM192194v1_genomic	2	3928293	4194523	28.01	3653928293	3653928293	73695858.7	1	1	0	2	2	2	2	2	2	4194523	4194523	4194523	4194523	4194523	4194523
GCA_001921965.1_ASM192196v1_genomic	2	3994274	4011842	28.19	3994274	3994274	3976859.9	1	1	0	2	2	2	2	1	1	4011842	4011842	4011842	4011842	3994274	3994274
GCA_001921985.1_ASM192198v1_genomic	4	3841378	4303351	27.89	3841378	244784	3452787.3	1	2	0	4	4	4	4	3	3	4303351	4303351	4303351	4303351	4291880	4291880
GCA_001951135.2_ASM1951	26	3982	4252	27	39	39	3731	1	1	0.56	26	26	18	12	2	1	4252578	4252578	4227107	4186039	4019222	3982791

13v2_genomic		791	578	.95	827915	827911	209.5															
GCA_002024365.1_ASM202436v1_genomic	146	237153	3995150	28	0355	7917	65567.6	24	72	0	160	130	99	90	61	25	4000805	3983932	3911866	3851594	3369586	2058859
GCA_002024375.1_ASM202437v1_genomic	135	191042	4000619	28.02	53118	23176	67426.2	22	66	0	148	121	92	82	62	25	4005673	3990339	3924280	3855323	3511911	2176221
GCA_002024385.1_ASM202438v1_genomic	22	1014528	3897060	27.95	956560	121134	647666.6	2	7	54.43	22	21	15	15	11	10	3897060	3896323	3882040	3882040	3804491	3754833
GCA_002024395.1_ASM202439v1_genomic	122	271192	3874100	27.87	60822	18193	77650	20	66	0	126	117	94	83	54	23	3875827	3870590	3811434	3722970	3237288	2163193

GCA_002024445.1_ASM202444v1_genomic	42	820215	3798005	28.04	238565	47246	339993.6	5	17	76.3	43	40	30	29	23	15	3798317	3796985	3769835	3760462	3640450	3324385
GCA_002024465.1_ASM202446v1_genomic	100	291050	4021142	27.9	75722	29493	114109.4	14	44	0	106	84	68	62	45	30	4023802	4011197	3974737	3935763	3646742	3079310
GCA_002103795.1_ASM210379v1_genomic	43	731916	4227590	27.84	407073	140764	469358	4	10	0	54	32	16	16	15	13	4230844	4219897	4185507	4185507	4163536	4095158
GCA_002103805.1_ASM210380v1_genomic	29	1123821	4040160	28	744494	282763	695317.6	3	6	0	39	22	14	13	10	9	4043191	4035566	4019424	4012258	3957292	3922396
GCA_002103825.1_ASM210382v1_genomic	29	754790	407761	27.	301	126	365996	5	13	0	35	26	18	18	17	16	4079517	4075101	4057630	4057630	4032730	4004104

			2	8	3	8	.1															
				6	2	1																
					3	9																
					5	3																
				2	8	0																
			39	7	3	0																
GCA_0021038		82	71	.	1	2	55															
55.1_ASM2103	2	17	70	9	9	2	79										3972	39689	39390	39390	39173	38885
85v1_genomic	9	22	9	8	1	8	24	3	7	0	32	25	11	11	10	9	674	96	78	78	71	58
					7	2																
				2	1	8																
		15	39	7	6	4	90															
GCA_0021038		47	04	.	7	9	70															
75.1_ASM2103	1	03	50	9	5	1	85										3906	39031	38841	38841	38841	38495
87v1_genomic	9	2	5	7	3	5	.9	2	5	0	26	17	9	9	9	8	590	50	22	22	22	13
					7	1																
				2	1	4																
		15	41	7	9	8	83															
GCA_0021038		45	11	.	2	9	15															
85.1_ASM2103	3	04	62	9	8	1	21										4113	41054	40866	40804	40532	40098
88v1_genomic	2	3	1	7	2	5	.4	2	7	0	37	23	14	13	11	10	388	02	10	99	82	96
				2	2																	
			38	7	8	8	35															
GCA_0021039	2	10	77	.	8	9	48		1													
75.1_ASM2103	4	20	78	9	9	0	5.	4	3								3888	38618	36947	33877	21656	11045
97v1_genomic	9	77	4	8	7	0	6	0	5	0	276	226	165	124	48	17	462	78	21	58	43	78

GCA_002103985.1_ASM210398v1_genomic	76	327954	3938211	28.02	123365	25752	152338.6	9	33	0	87	73	57	49	34	23	3941623	3935804	3892499	3833338	3575770	3173892
GCA_002104015.1_ASM210401v1_genomic	36	860537	3949014	28	593817	113195	518221.9	3	10	0	87	28	17	17	15	12	3963867	3943391	3920107	3920107	3880007	3769920
GCA_002260585.1_C.botulinum_2.0_genomic	275	57688	2904173	28.36	12831	5283	16001.2	72	213	0.07	275	275	227	104	15	1	2904173	2904173	2688900	1830056	506583	57688
GCA_002865745.1_ASM286574v1_genomic	1	3945134	3945134	28.24	3945134	3945134	3945134	1	1	0	1	1	1	1	1	1	3945134	3945134	3945134	3945134	3945134	3945134
GCA_002865765.1_ASM2865	1	3917	3917	28	39	39	3917	1	1	0	1	1	1	1	1	1	3917597	3917597	3917597	3917597	3917597	3917597

76v1_genomic		59	59	.	1	1	59															
		7	7	2	7	7	7															
				6	5	5																
					9	9																
					7	7																
					3	3																
					9	9																
				2	0	0	38															
		39	39	8	4	4	94															
GCA_0028658		04	14	.	4	4	39															
05.1_ASM2865		45	56	1	5	5	5.										3914	39145	39145	39145	39044	39044
80v1_genomic	2	4	5	6	4	4	2	1	1	0	2	2	2	2	1	1	565	65	65	65	54	54
					3	3																
					9	9																
					2	2	37															
		39	41		2	2	13															
GCA_0028658		22	54		0	0	54															
25.1_ASM2865		09	94	2	9	9	0.										4154	41549	41549	41549	41375	41375
82v1_genomic	3	9	9	8	9	9	2	1	1	0	3	3	3	3	2	2	949	49	49	49	19	19
					4	4																
					1	1																
				2	1	1	40															
		41	41	8	6	6	74															
GCA_0028658		16	59	.	8	8	95															
45.1_ASM2865		85	64	2	5	5	2.										4159	41596	41596	41596	41596	41168
84v1_genomic	2	6	0	3	6	6	1	1	1	0	2	2	2	2	2	1	640	40	40	40	40	56

GCA_002865885.1_ASM286588v1_genomic	3	4051146	4084533	28.2	4051146	4051146	4018189.6	1	1	0	3	3	3	3	1	1	4084533	4084533	4084533	4084533	4051146	4051146
GCA_002866045.1_ASM286604v1_genomic	2	3928430	4169506	28.16	3928430	3928430	3715231.5	1	1	0	2	2	2	2	2	2	4169506	4169506	4169506	4169506	4169506	4169506
GCA_002866125.1_ASM286612v1_genomic	4	3959509	4050477	28.15	3959509	3959509	3871561.8	1	1	0	4	4	4	4	2	2	4050477	4050477	4050477	4050477	4017094	4017094
GCA_002866225.1_ASM286622v1_genomic	3	4029015	4109153	28.19	40290	40290	3951358.3	1	1	0	3	3	3	3	2	2	4109153	4109153	4109153	4109153	4085859	4085859

GCA_003014955.1_ASM301495v1_genomic	155	186179	3877613	28.07	1545877	1514595	54593.9	27	86	0	155	145	119	99	54	22	3877613	3870361	3806271	3658944	2893958	1703622
GCA_003017145.1_ASM301714v1_genomic	1	3988693	3988693	27.85	3988693	3988693	3988693	1	1	0	1	1	1	1	1	1	3988693	3988693	3988693	3988693	3988693	3988693
GCA_003017195.1_ASM301719v1_genomic	1	4004635	4004635	27.92	4004635	4004635	4004635	1	1	0	1	1	1	1	1	1	4004635	4004635	4004635	4004635	4004635	4004635
GCA_003017225.1_ASM301722v1_genomic	1	3998747	3998747	27.79	39987	39987	3998747	1	1	0	1	1	1	1	1	1	3998747	3998747	3998747	3998747	3998747	3998747

GCA_003017335.1_ASM301733v1_genomic	1	3978960	3978960	28.08	473978960	473978960	3978960	1	1	0	1	1	1	1	1	1	3978960	3978960	3978960	3978960	3978960	3978960
GCA_003058345.1_ASM305834v1_genomic	1	3858511	3858511	28.22	3858511	3858511	3858511	1	1	0	1	1	1	1	1	1	3858511	3858511	3858511	3858511	3858511	3858511
GCA_003058445.1_ASM305844v1_genomic	2	3920316	3930410	28.19	3920316	3920316	3910273.8	1	1	0	2	2	2	2	1	1	3930410	3930410	3930410	3930410	3920316	3920316
GCA_003345315.1_ASM334531v1_genomic	3	395490	401850	28.	395	395	389314	1	1	0	3	3	3	2	2	2	4018503	4018503	4018503	4012577	4012577	4012577

		1	3	2	490139	490139	2.2															
GCA_003345335.1_ASM334533v1_genomic	3	3922194	4202171	28.07	22194	22194	3678245.8	1	1	0	3	3	3	2	2	2	4202171	4202171	4202171	4192218	4192218	4192218
GCA_003412385.1_ASM341238v1_genomic	220	233500	4149224	27.96	34270	8743	50606.3	36	123	0	220	214	164	118	54	16	4149224	4144779	4019833	3690633	2613466	1300616
GCA_003412395.1_ASM341239v1_genomic	485	59414	4050896	27.92	12720	1440	17145.1	91	302	0	485	460	261	131	20	3	4050896	4030672	3459239	2481149	764356	1694944
GCA_003412435.1_ASM341243v1_genomic	93	405272	4196137	27.9	16128	3627	176446.8	9	31	0	93	74	52	46	34	26	4196137	4183828	4139027	4099440	3896780	3590299

GCA_003515665.1_ASM351566v1_genomic	615	107208	4162303	27.93	312639	3403	19479.5	92	331	0	615	494	254	136	22	5	4162303	4084818	3433080	2577443	910669	354012
GCA_003610755.1_ASM361075v1_genomic	23	1288732	3822134	27.99	850668	308838	800412.8	2	5	0	51	18	11	11	9	7	3830015	3818706	3802847	3802847	3766071	3708389
GCA_003994935.1_ASM399493v1_genomic	5	3648522	3896768	28.18	3648522	3648522	3430586.1	1	1	0	5	5	3	2	2	2	3896768	3896768	3891344	3886099	3886099	3886099
GCA_003996845.1_ASM399684v1_genomic	228	117147	4035670	28.01	32017	8280	38850.3	38	129	0	228	218	169	114	55	18	4035670	4028857	3894196	3501391	2514327	1226207
GCA_003996885.1_ASM3996	46	7820	4024	28	25	69	3435	5	15	0	46	34	26	26	21	16	4024675	4017564	4001277	4001277	3911664	3742543

88v1_genomic		67	675	.06	53281	650	36.1															
GCA_003996895.1_ASM399689v1_genomic	71	305408	4019721	28.08	30857	33235	147552.6	10	32	0	71	62	51	50	36	25	4019721	4014038	3989031	3983705	3744027	3346264
GCA_003996925.1_ASM399692v1_genomic	457	63587	4050546	28.22	14089	4222	16874.7	91	288	0	457	445	257	146	25	2	4050546	4040962	3506356	2690404	828128	117246
GCA_003996935.1_ASM399693v1_genomic	71	518778	3905902	28.12	384775	95569	338395.2	5	12	0	71	45	22	21	16	13	3905902	3886524	3841440	3835710	3751272	3647074
GCA_004120535.1_ASM412053v1_genomic	47	560790	4015174	28.05	27770	93329	315492.9	5	15	0	47	32	24	23	20	19	4015174	4005899	3990188	3983711	3947798	3913004

GCA_009733885.1_ASM973388v1_genomic	2	3886767	3903111	28.24	93886767	3886767	3870559.9	1	1	0	2	2	2	2	1	1	3903111	3903111	3903111	3903111	3886767	3886767
GCA_009938935.1_ASM993893v1_genomic	87	437390	4317030	27.75	118884	25058	156108.9	11	40	0	99	85	67	57	40	27	4320953	4315747	4268016	4190308	3904009	3463214
GCA_009939325.1_ASM993932v1_genomic	108	260350	4309445	27.76	87657	19761	102244.3	15	56	0	121	105	85	75	48	28	4313565	4307486	4250480	4177848	3705386	2981084
GCA_009939345.1_ASM993934v1_genomic	65	362589	4319503	27.75	175917	34207	174361.3	9	30	0	79	64	50	46	33	25	4323822	4318894	4283922	4259458	4012630	3734869
GCA_0100783	7	40	43	2	1	2	15	1	3	0	85	73	63	57	40	25	4323	43193	42943	42425	39324	33449

15.1_ASM1007831v1_genomic	4	3612	19929	7.75	152422	8406	5307.8	0	9								559	20	87	70	58	89
GCA_011009485.1_ASM1100948v1_genomic	56	579507	3971297	28.11	184213	41489	244921.7	6	24	0	65	53	41	39	28	20	3973868	3969579	3936527	3923779	3742528	3437033
GCA_011009795.1_ASM1100979v1_genomic	35	656569	3863409	28.01	658713	81471	377997.7	4	12	0	49	33	25	23	18	13	3867730	3861932	3844204	3833840	3748677	3554113
GCA_011009805.1_ASM1100980v1_genomic	35	484638	3855398	28.02	25341	98463	291364.3	5	13	0	54	32	22	19	18	16	3861023	3853205	3830515	3808280	3787387	3710687
GCA_011009855.1_ASM1100985v1_genomi	49	399466	402384	27.	225	668	214439	7	20	0	61	44	35	33	26	23	4027438	4020274	3999136	3985210	3883974	3801746

c			9	8	9	7	.1															
				8	1	7																
					3																	
				2	5	1																
GCA_0110098			37	8	8	3	79															
75.1_ASM1100	1	25	91	.	7	6	53															
987v1_genomi	3	27	95	0	1	5	1.	1	6								3798	37878	37213	35664	30338	22399
c	0	37	4	2	6	4	7	9	9	0	154	125	102	81	48	25	868	21	02	98	82	37
				2	8	1																
GCA_0110098			34	8	7	5	95															
85.1_ASM1100	1	23	67	.	7	8	35															
988v1_genomi	0	28	30	0	0	0	5.	1	4								3473	34632	33990	32834	28962	24962
c	2	26	5	9	8	8	7	3	8	0	123	97	75	59	36	25	206	47	18	59	30	02
					2																	
				2	9	6																
GCA_0110099			38	8	6	1	38															
15.1_ASM1100		83	74	.	2	4	72															
991v1_genomi	4	30	98	0	2	9	87		1								3880	38694	38477	38477	37701	36042
c	1	35	2	5	8	6	.7	4	4	0	61	34	24	24	20	15	866	25	82	82	31	29
					4																	
				2	4	9																
GCA_0110099			42	7	1	6	45															
25.1_ASM1100		74	68	.	2	5	65															
992v1_genomi	4	48	39	8	6	3	68		1								4274	42605	42231	42231	41709	40988
c	8	12	0	8	1	7	.7	4	1	0	70	37	19	19	16	14	843	44	39	39	87	28
GCA_0110099	7	32	37	2	1	4	13	1	3	0	83	63	49	47	38	29	3798	37896	37532	37405	35832	32435

55.1_ASM1100995v1_genomic	0	5259	94182	8.03	028444	1603	2372.7	2	3								113	00	03	55	68	55
GCA_011009975.1_ASM1100997v1_genomic	30	701218	3887594	27.99	005711	92509	419312.7	4	11	0	41	28	17	17	17	16	3891053	3886540	3863127	3863127	3863127	3818034
GCA_011009985.1_ASM1100998v1_genomic	79	396172	3844066	27.98	145658	245911	158239.5	10	35	0	97	77	59	50	34	23	3849420	3843004	3797768	3737725	3447206	3050251
GCA_011010005.1_ASM1101000v1_genomic	102	176527	3976706	27.1	4046	9177	85393	17	52	0	114	97	80	69	47	31	3980847	3973606	3933713	3848141	3484242	2896919
GCA_011010035.1_ASM1101003v1_genomic	58	418189	3852700	28.0	1384	4643	190096.7	8	26	0	69	57	48	40	28	22	3856330	3852091	3827846	3767305	3562616	3316457

GCA_011010055.1_ASM1101005v1_genomic	95	305499	3851952	328.03	2381619	318371	103307.6	14	49	0	112	92	79	70	42	28	3857002	3849744	3820074	3764341	3332714	2790225
GCA_011010065.1_ASM1101006v1_genomic	50	336598	3854144	28.03	212300	35381	201177.2	7	21	0	73	49	37	32	28	19	3860944	3853535	3825536	3787104	3710915	3404623
GCA_011010085.1_ASM1101008v1_genomic	50	349378	3859362	28.02	131596	45136	184867.1	8	25	0	61	49	39	39	32	22	3862813	3858753	3834252	3834252	3714791	3362177
GCA_011010105.1_ASM1101010v1_genomic	86	534885	3852440	28.03	90864	24681	160447.2	11	39	0	99	82	68	57	38	25	3856483	3849316	3812307	3730493	3449556	2909899
GCA_011010135.1_ASM1101	31	6115	3864	28	31	98	3155	5	13	0	41	30	21	20	18	16	3867578	3863724	3841121	3835719	3791323	3729963

013v1_genomi		93	33	.	2	4	54															
c			3	0	4	6	.4															
				1	2	3																
					1																	
				2	3																	
GCA_0110101			38	8	1	7																
55.1_ASM1101	2	13	24	.	8	8	44		1													
015v1_genomi	2	46	28	0	9	0	33	3	2								3828	38182	36201	33088	22393	12905
c	6	68	1	9	1	4	8	2	3	0	240	218	152	108	43	16	753	44	10	39	79	06
					1																	
				2	5	3																
GCA_0110101			38	8	3	9	18															
65.1_ASM1101		34	55	.	0	8	58															
016v1_genomi	5	97	10	0	9	1	67		2								3859	38544	38293	38043	35937	33720
c	4	90	7	2	6	8	.1	8	5	0	67	53	44	40	28	22	200	98	56	89	27	86
					1																	
				2	4	4																
GCA_0110101			38	8	0	3	17															
75.1_ASM1101		41	54	.	4	4	96															
017v1_genomi	6	07	86	0	7	2	13		2								3859	38542	37997	37748	35546	33591
c	7	30	4	3	9	9	.6	9	7	0	83	66	46	42	29	24	559	55	41	48	73	18
				2	1	3																
GCA_0110102			38	8	1	3	15															
05.1_ASM1101		36	54	.	5	9	71															
020v1_genomi	6	83	15	0	4	8	24		3								3859	38527	38217	37634	35810	31954
c	7	57	7	3	4	2	.8	9	1	0	86	65	54	46	34	24	700	11	60	35	94	11

GCA_011010235.1_ASM1101023v1_genomic	125	234423	4172991	27.93	677461	19415	91547.1	17	58	0	138	118	87	75	48	32	4176930	4168128	4094334	3998520	3549034	3015057
GCA_011010245.1_ASM1101024v1_genomic	109	231814	3851739	28.04	69977	18741	84662.5	18	58	0	127	107	85	76	46	29	3857022	3850206	3789099	3722820	3205600	2629825
GCA_011010275.1_ASM1101027v1_genomic	103	215744	4132035	27.9	98626	27190	102130.4	15	48	0	114	92	74	64	50	27	4134956	4125111	4078139	4008977	3783102	2977901
GCA_011010285.1_ASM1101028v1_genomic	31	451214	3862824	28.01	312421	82592	286902.5	6	14	0	42	30	23	22	21	17	3866454	3862215	3846515	3841072	3820031	3661861
GCA_011010295.1_ASM1101029v1_genomic	44	454861	3862071	28.0	2597	4161	245254.8	6	20	0	58	43	35	33	27	18	3866446	3861462	3841348	3827666	3712668	3394680

GCA_011010335.1_ASM1101033v1_genomic	118	254239	4110959	227.88	0166455	916263	98016.7	16	58	0	133	114	92	74	48	26	4115608	4108033	4049336	3915656	3500259	2697572
GCA_011010345.1_ASM1101034v1_genomic	133	232754	3904815	28.01	62150	15533	86135.7	17	65	0	146	124	97	79	48	24	3908835	3898489	3830855	3694062	3184868	2373425
GCA_011010355.1_ASM1101035v1_genomic	56	333260	3909619	28	157181	60191	165404.4	9	24	0	66	49	37	36	31	25	3912899	3905012	3879433	3872007	3789573	3577570
GCA_011010375.1_ASM1101037v1_genomic	53	459688	3905917	28	239332	50179	224504.9	7	20	0	64	45	33	32	27	20	3909273	3900799	3873453	3867531	3774448	3539455
GCA_011010415.1_ASM1101	11	2386	3991	28	68	20	8804	17	54	0	134	113	84	69	49	28	3996158	3987061	3911570	3802850	3494630	2678498

041v1_genomi	9	69	26	.	2	0	7.															
c			7	0	6	4	6															
				8	8	2																
				2	8	1																
GCA_0110104			39	8	1	7	11															
35.1_ASM1101	1	33	62	.	9	9	28															
043v1_genomi	1	71	44	0	9	1	03	1	5								3967	39562	38811	38032	32912	26960
c	5	33	8	8	1	1	.6	4	3	0	134	107	81	70	39	24	861	70	74	13	84	83
					1																	
				2	0	2																
GCA_0110104			39	8	4	5	14															
45.1_ASM1101		42	81	.	3	6	13															
044v1_genomi	9	10	08	0	3	2	95	1	4								3985	39760	39267	38510	35888	29309
c	4	93	2	9	0	3	.2	1	1	0	109	87	68	57	41	23	895	88	52	85	91	48
					1																	
				2	4	3																
GCA_0110104			39	8	2	0	13															
55.1_ASM1101		29	81	.	8	5	50															
045v1_genomi	8	33	40	0	0	0	24	1	3								3985	39764	39294	38696	37107	31302
c	5	93	5	5	2	8	.7	0	6	0	97	78	58	49	40	24	038	05	52	43	14	79
					1																	
				2	4	4																
GCA_0110104			39	8	5	1	16															
85.1_ASM1101		31	46	.	0	4	49															
048v1_genomi	6	47	84	0	6	5	52		2								3951	39428	39074	38729	36832	34209
c	8	40	6	3	7	1	.3	9	8	0	83	62	48	43	32	25	200	57	50	99	22	51

GCA_011010505.1_ASM1101050v1_genomic	83	472356	3944985	28.03	108928	26476	150204.5	11	39	0	99	76	61	56	41	26	3949710	3940342	3904332	3871748	3626454	3121440
GCA_011010535.1_ASM1101053v1_genomic	75	276958	3946356	28.04	134910	28811	132511.1	11	36	0	92	69	58	53	36	27	3951549	3942367	3913907	3874876	3571825	3240605
GCA_011010545.1_ASM1101054v1_genomic	78	335539	3946157	28.03	145067	22738	155486	9	34	0	93	72	59	52	31	25	3950691	3942168	3911661	3861972	3481929	3252719
GCA_011010575.1_ASM1101057v1_genomic	144	297754	3922917	28.06	66182	13366	86709.6	18	68	0	168	136	100	77	47	22	3929568	3917354	3820298	3645683	3176613	2244558
GCA_011010615.1_ASM1101061v1_genomi	137	222625	394001	28.	774	138	78275.	18	66	0	152	130	98	80	45	26	3944562	3934708	3842180	3711241	3131643	2474449

c			2	0	9	1	7															
				6	7	6																
				2	8	1																
GCA_0110106			41	8	0	4	89															
35.1_ASM1101	1	22	61	.	9	5	73															
063v1_genomi	4	08	76	0	3	1	6.	1	6								4168	41524	40653	38964	33074	28619
c	7	48	6	7	5	1	5	7	4	0	167	134	99	76	41	28	159	77	87	51	77	45
					1																	
				2	1	2																
GCA_0110106			42	7	7	2	14															
45.1_ASM1101	1	43	88	.	4	3	37															
064v1_genomi	1	26	40	9	6	0	81	1	4								4295	42783	42097	41413	37962	32647
c	9	74	2	1	0	7	.8	1	5	0	142	104	73	63	42	26	290	30	35	68	98	20
					2																	
				2	2	3																
GCA_0110106			41	8	2	4	20															
55.1_ASM1101		44	37	.	1	8	11															
065v1_genomi	6	29	71	0	4	4	16		2								4141	41357	41036	40501	38974	34934
c	7	74	0	6	1	5	.9	8	7	0	80	64	49	42	32	21	644	00	51	00	22	19
					4	1																
GCA_0110106			37	2	4	1	61															
85.1_ASM1101	1	17	13	8	1	1	44															
068v1_genomi	7	31	95	.	5	2	0.	2	8								3719	37065	36077	34160	26571	16004
c	4	92	2	3	4	7	6	3	8	0	193	164	122	95	47	17	910	77	10	04	75	49
GCA_0110107	1	59	41	2	9	2	19		3								4167	41535	40969	40021	38311	34327
15.1_ASM1101	0	57	62	8	9	8	56	9	6	0	115	88	63	49	39	27	027	81	45	09	08	59

071v1_genomi	0	87	43	.	0	6	14															
c			6	0	7	4	.8															
				7	4	0																
					1																	
				2	2	3																
GCA_0110107			41	8	1	0	15															
35.1_ASM1101		39	63	.	0	9	33															
073v1_genomi	8	03	56	0	7	8	58	1	3								4168	41554	41015	40674	38729	35628
c	6	43	5	6	8	4	.5	1	1	0	102	75	51	46	35	26	441	76	80	29	52	90
				2	4	1																
GCA_0110107			39	8	8	3	62															
45.1_ASM1101	1	15	43	.	1	3	11															
074v1_genomi	5	69	04	0	7	7	5.	2	8								3948	39383	38325	37108	28761	19642
c	9	79	1	2	9	8	1	4	5	0	177	152	116	98	48	23	662	35	25	36	08	80
					1																	
				2	0	3																
GCA_0110107			37	8	1	3	12															
75.1_ASM1101		30	93	.	0	8	98															
077v1_genomi	7	41	87	0	6	7	37	1	3								3798	37911	37538	37103	35269	29866
c	5	26	3	2	2	0	.1	1	7	0	89	71	56	50	40	25	028	32	87	45	91	04
					1																	
					5	3																
GCA_0110107			39		0	6	15															
85.1_ASM1101		30	48		2	3	97															
078v1_genomi	6	94	24	2	4	3	44		3								3952	39455	39223	38886	37378	33484
c	5	38	2	8	6	7	.5	9	1	0	80	61	51	46	36	25	848	84	63	91	04	00

GCA_011010835.1_ASM1101083v1_genomic	157	265178	4217871	27.89	67578	15542	84215.4	19	70	0	176	141	104	82	51	25	4223375	4206673	4109350	3954016	3418924	2468043
GCA_011010865.1_ASM1101086v1_genomic	57	427071	4224536	27.87	236704	55498	218186.3	7	22	0	72	51	35	33	29	23	4228906	4220802	4188065	4175179	4103401	3894559
GCA_011010895.1_ASM1101089v1_genomic	106	357418	4222791	27.88	90981	26609	135074.4	13	48	0	126	100	74	66	49	26	4228724	4218831	4156272	4100170	3827494	2994848
GCA_011010905.1_ASM1101090v1_genomic	50	538039	4224079	27.87	292033	78221	276339.9	6	17	0	65	43	29	26	24	20	4228449	4219465	4191330	4174011	4142044	3984204
GCA_011010935.1_ASM1101093v1_genomic	84	291199	3761753	27.9	8052	2357	114864.1	12	42	0	96	83	68	60	38	28	3765488	3761144	3715326	3667415	3291083	2908253

GCA_011010945.1_ASM1101094v1_genomic	67	368067	3792699	928.02	3104920	531975	148313.2	10	34	0	77	63	50	48	39	23	3795956	3790263	3761280	3750044	3579513	2988507
GCA_011010965.1_ASM1101096v1_genomic	73	440189	4086599	28.02	116047	39905	166857.9	10	32	0	88	69	51	49	37	28	4090957	4083980	4045946	4031018	3820352	3507166
GCA_011010975.1_ASM1101097v1_genomic	66	461851	4087032	28.02	278117	53408	241548.5	6	23	0	82	59	43	38	27	23	4091619	4082418	4051825	4019020	3843276	3714596
GCA_011011015.1_ASM1101101v1_genomic	63	482173	4087224	28.02	209473	52479	223010.3	7	25	0	77	55	37	36	30	25	4091150	4082397	4045732	4039485	3921212	3721515
GCA_0110110	7	42	40	2	1	3	17	9	2	0	95	69	47	40	35	25	4091	40824	40379	39950	39191	35862

35.1_ASM1101	5	09	86	8	4	4	50		8								843	56	89	26	95	70
103v1_genomi		64	23	.	0	5	42															
c			8	0	4	6	.8															
				2	0	9																
					1																	
					1																	
				2	7	5																
GCA_0110110			40	8	6	5	21															
45.1_ASM1101		53	87	.	4	3	48															
104v1_genomi	6	37	12	0	6	7	13		2								4091	40833	40491	40355	39111	37172
c	3	02	2	2	6	6	.2	7	4	0	78	57	40	38	30	24	492	88	85	92	41	28
				2	8	1																
GCA_0110110			40	8	4	6	11															
75.1_ASM1101	1	33	73	.	4	6	75															
107v1_genomi	2	60	73	0	2	9	68	1	5								4078	40673	39866	38138	34065	28923
c	9	38	2	4	6	1	.3	4	5	0	147	120	89	65	42	27	951	81	34	39	57	06
					1																	
				2	2	4																
GCA_0110110			40	8	2	8	21															
95.1_ASM1101		58	84	.	4	2	57															
109v1_genomi	7	91	02	0	0	9	52		2								4088	40790	40308	39942	38055	36387
c	3	96	0	3	4	1	.1	8	7	0	90	66	46	41	30	26	873	85	08	11	40	50
GCA_0110111			40	2	1	3	13															
05.1_ASM1101		36	84	8	0	1	91															
110v1_genomi	9	28	09	.	8	6	71	1	3								4089	40774	40171	39817	37570	33627
c	1	37	6	0	2	7	.8	2	8	0	108	82	59	54	40	29	245	51	90	73	96	73

GCA_011011135.1_ASM1101113v1_genomic	83	461219	4085081	328.03	38106292	635889	163574.7	10	35	0	100	77	57	47	40	28	4089887	4081235	4040312	3964880	3844392	3417282
GCA_011011145.1_ASM1101114v1_genomic	149	260757	4075139	28.05	57202	15476	84150.7	19	74	0	167	143	103	90	51	23	4080372	4071405	3967275	3877240	3240605	2280952
GCA_011011165.1_ASM1101116v1_genomic	101	277269	4082612	28.03	96552	23338	110182.7	14	45	0	116	96	71	60	44	28	4086921	4079244	4012494	3926941	3651491	3095023
GCA_011011185.1_ASM1101118v1_genomic	85	413351	4082437	28.03	173077	30383	182484.2	8	31	0	103	76	54	46	35	21	4087594	4076807	4026351	3967747	3789701	3297793
GCA_011011215.1_ASM1101	10	4611	4084	28	98	23	1375	12	46	0	120	97	70	63	44	27	4088527	4079196	4006034	3955596	3641072	3038292

121v1_genomi	5	93	35	.	2	7	80															
c			4	0	1	0	.3															
				3	4	7																
				2	8	2																
GCA_0110112			39	7	9	4	11															
35.1_ASM1101		29	29	.	8	5	65															
123v1_genomi	8	57	30	9	5	0	32	1	4								3931	39281	38814	38331	35153	30009
c	5	67	4	9	7	3	.7	3	1	0	90	83	65	58	40	27	029	64	10	45	61	74
				2	9	2																
GCA_0110112			39	7	8	6	11															
45.1_ASM1101		22	30	.	9	3	69															
124v1_genomi	8	90	92	9	2	1	13	1	4								3933	39292	38855	38244	36049	31088
c	5	62	3	9	9	4	.9	2	0	0	91	82	64	56	42	28	063	72	36	30	05	04
					4																	
				2	8	9																
GCA_0110112			40	8	3	1	43															
55.1_ASM1101		74	84	.	1	0	95															
125v1_genomi	4	09	77	0	2	0	74		1								4090	40815	40378	40378	40101	39083
c	6	08	9	2	7	2	.2	4	1	0	65	41	19	19	17	14	337	59	44	44	09	05
					1																	
				2	0	2																
GCA_0110112			39	8	3	1	11															
95.1_ASM1101		22	72	.	2	6	08															
129v1_genomi	9	49	08	0	3	1	04	1	4								3975	39680	39056	38393	35259	29050
c	8	69	2	5	1	5	.7	3	4	0	107	91	70	60	41	25	101	58	04	80	68	82
GCA_0110113	5	32	39	2	1	5	17	9	2	0	65	52	41	37	30	25	3978	39724	39425	39145	37903	36205

15.1_ASM1101	9	46	76	8	7	9	07		5								619	70	33	15	25	89
131v1_genomi		08	49	.	8	9	58															
c			4	0	6	6	.1															
				4	7	2																
					8																	
					3																	
				2	0	6																
GCA_0110113			39	8	8	0	35															
35.1_ASM1101		66	76	.	1	9	78															
133v1_genomi	4	34	32	0	9	5	61		1								3978	39723	39509	39402	39219	38109
c	2	36	5	4	0	0	.2	4	6	0	48	35	26	24	23	20	450	01	09	82	68	91
					1																	
				2	0	2																
GCA_0110113			39	8	1	8	13															
45.1_ASM1101		29	13	.	1	8	12															
134v1_genomi	8	97	61	0	6	2	20	1	4								3916	39098	38647	38069	35788	30888
c	4	70	1	5	3	9	.6	1	1	0	94	78	62	55	42	28	426	56	32	82	72	57
				2	5	1																
GCA_0110113			39	8	5	9	74															
75.1_ASM1101	1	26	94	.	3	0	81															
137v1_genomi	4	83	94	0	9	6	7.	2	7								3998	39869	39195	37933	32249	21469
c	1	08	8	9	0	3	1	2	2	0	153	130	104	86	54	24	935	80	98	76	42	28
GCA_0110113			39	2	1	5	19															
85.1_ASM1101		39	31	7	8	8	79															
138v1_genomi	5	53	50	.	5	5	08		2								3934	39303	39018	38755	38048	35990
c	0	95	1	9	1	7	.7	8	2	0	59	48	36	32	28	23	183	61	94	92	58	61

GCA_011011415.1_ASM1101141v1_genomic	31	1078187	4000424	828.08	05296954	494088	470673	4	13	0	41	27	20	19	19	16	4003589	3997990	3982593	3977282	3977282	3877502
GCA_011011435.1_ASM1101143v1_genomic	99	318135	3995213	28.1	96816	20179	128104.5	11	44	0	112	91	73	61	41	26	3999383	3989546	3944164	3856268	3538568	3014673
GCA_011011445.1_ASM1101144v1_genomic	181	239287	4082421	28.04	48488	11343	62770	26	90	0	197	169	124	97	52	25	4087213	4074308	3959920	3760860	3020171	2029252
GCA_011011465.1_ASM1101146v1_genomic	98	493490	4105858	28.02	116058	43374	187552.2	9	33	0	118	78	52	47	36	28	4111607	4091189	4035055	4001551	3822842	3506324
GCA_011011495.1_ASM1101	13	2153	3789	28	60	13	7160	20	69	0	148	129	100	83	47	26	3793988	3786528	3706432	3578945	3005180	2256288

149v1_genomi	4	81	93	.	9	8	1.															
c			6	0	4	3	5															
				4	9	3																
					2																	
				2	1	5																
GCA_0110115			37	8	5	6	22															
15.1_ASM1101		40	96	.	8	2	16															
151v1_genomi	4	38	01	0	9	5	17		2								3798	37935	37695	37639	36881	34564
c	7	78	4	2	5	3	.4	7	0	0	56	43	32	31	27	20	774	06	08	89	00	11
				2	4	1																
GCA_0110115			31	8	2	3	50															
35.1_ASM1101	1	13	53	.	4	6	25															
153v1_genomi	3	46	94	2	4	9	5.	2	7								3155	31517	30573	29513	22758	13365
c	2	33	9	1	4	8	2	4	2	0	137	128	95	81	44	18	674	29	61	06	43	78
					9	2																
GCA_0110115			39		9	5	11															
45.1_ASM1101		25	55		5	7	09															
154v1_genomi	8	60	51	2	8	4	93	1	4								3958	39539	39222	38980	35977	30406
c	0	95	1	8	4	6	.8	3	1	0	89	77	63	59	42	28	357	26	45	31	07	14
				2	7	1																
GCA_0110115			38	8	3	8	99															
75.1_ASM1101	1	34	07	.	2	4	10															
157v1_genomi	1	70	33	0	0	1	7.	1	5								3810	38038	37254	36278	32190	25564
c	8	42	1	1	9	7	8	6	4	0	131	113	83	70	44	26	990	57	24	26	91	61
GCA_0110115	4	37	38	2	2	5	22		2								3871	38667	38438	38213	37543	34833
85.1_ASM1101	8	35	68	7	7	1	61	6	1	0	56	45	34	31	28	21	259	67	24	55	00	62

158v1_genomi		87	34	.	1	4	56															
c			6	9	7	8	.5															
				8	6	0																
					9																	
					2																	
				2	9	6																
GCA_0110116			39	7	5	3	30															
15.1_ASM1101		63	32	.	3	3	45															
161v1_genomi	4	35	66	9	2	0	07		1								3934	39305	39071	38821	38240	36764
c	4	27	9	8	8	8	.4	5	8	0	48	41	32	28	24	20	160	99	07	74	52	10
					4	1																
				2	2	8																
GCA_0110116		10	38	7	0	3																
35.1_ASM1101		63	48	.	1	4	62															
163v1_genomi	2	32	80	9	9	4	11										3852	38477	38315	38261	38050	38050
c	3	3	7	7	0	2	90	3	7	0	33	21	13	12	11	11	127	45	89	98	70	70
				2	8	1																
GCA_0110116			38	8	0	7	12															
45.1_ASM1101	1	33	21	.	1	5	28															
164v1_genomi	0	68	85	0	5	5	14	1	4								3826	38206	37445	36706	31989	27097
c	6	13	0	4	9	4	.9	2	9	0	120	104	75	65	37	23	272	56	47	37	06	40
				2	1	3																
GCA_0110116			37	8	0	1	13															
65.1_ASM1101		30	91	.	1	1	84															
166v1_genomi	7	71	95	0	0	8	20	1	3								3795	37894	37579	37521	34938	29947
c	1	20	7	3	4	7	.5	0	6	0	83	67	55	54	38	25	683	69	23	44	57	24

GCA_011011695.1_ASM1101169v1_genomic	35	513015	3795626	28.02	6300430	90756	287021.1	5	14	0	46	32	23	22	21	17	3799096	3793757	3773692	3767971	3744635	3595611
GCA_011011715.1_ASM1101171v1_genomic	65	304781	3872874	28	126624	38206	141620.4	10	33	0	77	61	48	47	41	24	3876634	3870300	3838955	3833189	3739428	3118289
GCA_011011725.1_ASM1101172v1_genomic	56	370544	3872115	28	186760	41835	175984.1	8	26	0	71	51	38	37	34	24	3876531	3869008	3837790	3832656	3777514	3427010
GCA_011011735.1_ASM1101173v1_genomic	137	177221	3800979	28.07	53416	13795	65178.8	22	72	0	151	133	100	87	51	24	3805074	3798439	3696880	3595796	3011832	2053588
GCA_011011755.1_ASM1101	87	2804	3774	28	75	25	1173	12	47	0	99	83	68	63	47	22	3778049	3771949	3734803	3697953	3406065	2515312

175v1_genomic		08	452	.042	5975	1971	55															
GCA_011011765.1_ASM1101176v1_genomic	125	216122	3790637	8.042	26539	56482	76411.8	19	67	0	137	122	98	80	51	21	3794376	3788768	3723261	3580612	3116587	2005922
GCA_011011815.1_ASM1101181v1_genomic	96	241655	3765116	7.992	309214	15335	97906.2	14	48	0	103	93	74	67	43	23	3767669	3763247	3709178	3662298	3277520	2541399
GCA_011011835.1_ASM1101183v1_genomic	55	323329	3863668	8.012	28464	56601	166499.3	9	25	0	70	52	40	38	33	25	3868071	3861799	3835977	3824513	3741036	3478966
GCA_011011845.1_ASM1101184v1_genomic	154	172311	3807223	8.07	7887	3562	62263.4	23	82	0	166	147	114	92	51	19	3810758	3802299	3719943	3559182	2842319	1738913
GCA_011011865.1_ASM1101	35	5631	3796	28	30	93	3079	5	13	0	48	31	21	20	19	16	3800178	3793611	3771907	3766317	3742981	3632253

186v1_genomi		99	21	.	0	0	89														
c			9	0	4	9	.8														
				2	3	2															
					0																
				2	8	3															
GCA_0110118			37	8	3	0	10														
85.1_ASM1101		35	93	.	9	1	78														
188v1_genomi	8	72	01	0	5	3	47	1	4								3796	37890	37442	37001	34750	29624
c	9	31	8	2	4	4	.6	5	2	0	102	83	65	58	44	30	818	39	84	86	69	42
					1																
				2	1	3															
GCA_0110118			37	8	8	8	12														
95.1_ASM1101		23	93	.	9	3	30														
189v1_genomi	6	71	00	0	8	3	80	1	3								3796	37904	37635	37352	35909	32877
c	6	47	5	2	5	7	.6	2	3	0	79	62	50	46	38	29	672	70	50	71	81	99
					1																
				2	7	4															
GCA_0110119			37	8	1	2	20														
25.1_ASM1101		45	95	.	3	4	58														
192v1_genomi	5	71	37	0	0	5	95		2								3799	37911	37564	37422	36842	32264
c	7	41	5	2	9	7	.3	7	6	0	70	51	38	36	33	21	286	45	93	47	92	03
				2	6	1															
GCA_0110119			37	8	5	7	88														
55.1_ASM1101	1	26	87	.	5	3	05														
195v1_genomi	2	85	27	0	3	4	1.	1	5								3791	37813	36975	35930	31744	23243
c	4	40	0	5	3	3	1	6	8	0	141	116	86	72	46	23	805	92	93	46	65	87

GCA_011011965.1_ASM1101196v1_genomic	44	378178	3861541	28.02	232319	59399	200235.6	7	21	0	56	43	36	33	27	22	3865412	3860932	3846048	3827366	3732935	3552689
GCA_011011995.1_ASM1101199v1_genomic	69	372959	3795195	28.02	112888	37448	150924.5	10	33	0	82	65	50	47	37	25	3799106	3792663	3756937	3734306	3564236	3109507
GCA_011012005.1_ASM1101200v1_genomic	128	284604	4329931	27.95	75878	19835	89131.2	18	64	0	154	117	95	83	56	29	4337561	4322491	4264053	4171003	3726864	2795430
GCA_011012015.1_ASM1101201v1_genomic	59	301935	3864960	27.98	133509	42016	137420.3	11	30	0	66	56	45	44	36	27	3867484	3863271	3839964	3834792	3690093	3361388
GCA_011012055.1_ASM1101205v1_genomi	61	373308	397670	28.	156	429	178930	8	27	0	76	55	42	39	33	24	3981193	3972363	3942810	3922844	3800417	3466320

c			7	0	4	6	.5															
				5	6	6																
					6																	
					1																	
				2	4	2																
GCA_0110120			41	8	0	9	21															
75.1_ASM1101		64	63	.	1	9	66															
207v1_genomi	8	06	06	0	0	9	71		3								4168	41542	40994	40519	38003	35915
c	9	63	6	6	0	4	.4	8	1	0	107	77	53	46	32	26	420	11	94	24	60	67
					1																	
				2	7	3																
GCA_0110120			41	8	3	5																
95.1_ASM1101		58	64	.	0	4	25															
209v1_genomi	8	59	57	0	6	3	10		2								4170	41545	40881	40383	38879	36460
c	8	13	8	6	9	6	12	6	5	0	107	74	45	38	29	22	318	60	72	09	63	18
					1																	
				2	3	3																
GCA_0110121			39	8	7	7	18															
05.1_ASM1101		44	70	.	1	0	62															
210v1_genomi	7	96	41	0	1	1	78		2								3972	39663	39273	38555	37778	34026
c	1	46	2	5	4	1	.4	8	9	0	78	64	51	40	35	24	945	88	73	25	32	61
				2	1	3																
GCA_0110121			40	8	4	0	20															
25.1_ASM1101		54	86	.	0	0	59															
212v1_genomi	8	68	37	0	4	3	82		2								4092	40807	40366	39600	37501	34855
c	4	52	4	2	0	1	.6	8	8	0	105	75	53	42	30	23	235	39	22	71	99	16

GCA_011012155.1_ASM1101215v1_genomic	113	296954	3994632	28.11	279623	17833	122392.2	12	58	0	120	109	90	76	44	18	3997069	3991936	3936152	3842356	3311714	2431266
GCA_011012165.1_ASM1101216v1_genomic	54	585606	4000134	28.08	192554	52205	233605	7	25	0	65	50	39	37	32	25	4003701	3997406	3970823	3958994	3877045	3645208
GCA_011012195.1_ASM1101219v1_genomic	127	234764	3946198	28.01	61324	16463	79849	19	63	0	138	122	92	78	50	25	3949924	3943085	3857147	3756862	3299089	2344663
GCA_011012215.1_ASM1101221v1_genomic	55	331701	3795398	28.02	137249	42457	164650.5	9	25	0	68	51	39	37	31	24	3799360	3792802	3766659	3754104	3641256	3393560
GCA_011012225.1_ASM1101222v1_genomi	126	204903	378665	28.	545	146	78495.	18	66	0	136	122	98	82	50	22	3789818	3784051	3717849	3608528	3090546	2112713

c			1	04	243	34	6															
GCA_011012355.1_ASM1101235v1_genomic	51	654991	3991807	27.99	405591	86621	347796.8	5	13	0	74	46	27	21	17	17	3998487	3988472	3938465	3898032	3837798	3837798
GCA_011012365.1_ASM1101236v1_genomic	67	351067	3904088	27.99	418762	36564	166418.2	8	30	0	83	64	54	45	32	26	3908841	3902144	3880924	3814059	3591252	3372506
GCA_011012375.1_ASM1101237v1_genomic	35	842773	3899272	27.99	337353	89161	371721	5	15	0	54	34	26	24	21	17	3905010	3898663	3879867	3868922	3812746	3656990
GCA_011012435.1_ASM1101243v1_genomic	40	609100	3889896	28.02	15616	69015	313773.9	5	15	0	59	33	26	26	22	17	3895678	3885125	3870050	3870050	3798609	3626336

GCA_011012455.1_ASM1101245v1_genomic	61	456745	3845485	28.05	199405	40526	202919.5	7	27	0	78	59	50	44	29	24	3850217	3844109	3823247	3779053	3523529	3336850
GCA_011012465.1_ASM1101246v1_genomic	27	809098	3996408	28.02	503308	99416	470247.6	3	11	0	39	26	17	17	16	14	4000037	3995851	3977964	3977964	3959803	3881487
GCA_011012695.1_ASM1101269v1_genomic	159	223754	4007306	28.1	43995	12868	66149.4	25	86	0	170	151	120	99	53	20	4010699	4000532	3909367	3758346	3008842	1793637
GCA_011012725.1_ASM1101272v1_genomic	55	370871	4060046	28.04	160958	43805	182949	9	25	0	64	52	43	41	30	23	4062902	4058145	4036258	4022151	3843135	3577241
GCA_011012735.1_ASM1101273v1_genomi	110	247394	384686	28.	569	190	88777.	17	58	0	128	108	90	72	52	22	3851997	3845589	3795211	3673931	3351078	2245494

c			9	0	4	9	2															
				6	9	1																
				2	3	1																
GCA_0110127			38	8	9	2	52															
55.1_ASM1101	1	18	56	.	7	2	78															
275v1_genomi	6	80	19	0	5	7	2.	2	9								3861	38541	37649	36108	26861	14351
c	6	11	3	4	8	9	6	9	6	0	184	163	130	108	52	18	614	25	30	01	71	75
				2	9	2																
GCA_0110127			38	8	9	9																
95.1_ASM1101		44	88	.	0	9	14															
279v1_genomi	7	15	05	0	1	5	92	1	3								3894	38859	38511	38057	35538	30558
c	6	10	5	1	6	6	41	1	9	0	98	73	61	55	40	27	468	48	12	32	38	10
					1																	
					1	3																
GCA_0110128			38		1	7	20															
15.1_ASM1101		61	91		0	6	13															
281v1_genomi	7	04	74	2	0	3	30		3								3910	38879	38595	38329	36030	31507
c	3	24	5	8	3	9	.9	8	4	0	145	67	54	50	36	25	017	53	49	68	27	83
					1																	
				2	0	1																
GCA_0110128			38	8	3	9	13															
25.1_ASM1101		29	64	.	5	9	06															
282v1_genomi	9	47	65	0	7	5	46	1	4								3871	38627	38218	37447	33814	29471
c	1	67	4	3	6	7	.5	1	2	0	116	88	70	60	37	24	748	87	78	19	93	66
GCA_0110128	3	57	40	2	2	8	31		1								4068	40648	40494	40380	39423	38473
35.1_ASM1101	6	80	65	8	9	4	37	5	5	0	45	35	28	26	21	18	260	27	45	09	77	93

283v1_genomi<br>c		76	43<br>5	.<br>0<br>3	2<br>3<br>8<br>7<br>1	5<br>7<br>2	03<br>.9															
GCA_0110128<br>75.1_ASM1101<br>287v1_genomi<br>c	6<br>4	42<br>04<br>94	41<br>79<br>39<br>0	2<br>7<br>.<br>9<br>8	6<br>9<br>4<br>7<br>5<br>1	4<br>3<br>2<br>0<br>7	17<br>99<br>38<br>.6	8	3<br>0	0	80	62	50	44	37	27	4183<br>819	41782<br>54	41550<br>36	41178<br>89	39999<br>64	36398<br>24
GCA_0110128<br>85.1_ASM1101<br>288v1_genomi<br>c	6<br>6	39<br>36<br>72	38<br>67<br>83<br>1	2<br>8<br>.<br>0<br>2	0<br>6<br>9<br>0<br>6<br>3	3<br>8<br>1<br>6<br>1<br>1	13<br>70<br>44<br>.1	1<br>2	3<br>3	0	85	65	54	49	37	29	3873<br>600	38672<br>22	38414<br>10	38129<br>30	36104<br>14	33376<br>51
GCA_0110130<br>55.1_ASM1101<br>305v1_genomi<br>c	4<br>3	73<br>48<br>76	39<br>99<br>66<br>8	2<br>8<br>.<br>0<br>5	2<br>8<br>1<br>6<br>9	3<br>1<br>2<br>5<br>3	45<br>23<br>20<br>.2	4	1<br>0	0	60	33	16	15	14	13	4005<br>020	39933<br>22	39582<br>22	39514<br>46	39358<br>87	39072<br>47
GCA_0110131<br>75.1_ASM1101<br>317v1_genomi<br>c	1<br>1<br>5	21<br>91<br>82	35<br>75<br>95<br>3	2<br>8<br>.<br>1	5<br>7<br>6<br>1	1<br>6<br>5<br>6	72<br>53<br>0.<br>4	1<br>9	6<br>5	0	135	109	94	84	48	22	3581<br>508	35721<br>24	35353<br>36	34593<br>88	28885<br>19	19707<br>94

				9	3	1																
					7																	
				2	3	8																
GCA_0110132		13	39	8	8	2	69															
35.1_ASM1101		53	94	.	1	1	19															
323v1_genomi	4	48	27	0	3	9	73		1								4001	39884	39477	39477	38955	37829
c	9	8	4	4	3	3	.5	2	1	0	74	40	19	19	16	13	618	50	27	27	75	74
				2	5	1																
GCA_0110132			38	8	5	1	10															
55.1_ASM1101	1	38	97	.	7	7	25															
325v1_genomi	4	78	41	0	8	4	61	1	7								3902	38943	37910	36244	30320	21012
c	7	43	7	1	4	8	.6	7	4	0	162	143	106	84	46	19	144	68	11	59	87	13
				2	6	1																
GCA_0110132			39	8	9	3																
65.1_ASM1101	1	25	92	.	8	5	97															
326v1_genomi	4	23	06	0	3	7	01	1	6								3997	39853	38990	37510	31703	25938
c	6	04	7	7	3	0	1	5	5	0	165	135	99	78	42	25	729	40	73	73	66	86
				2	7	1																
GCA_0110133			39	8	8	4	93															
15.1_ASM1101	1	22	99	.	0	1	58															
331v1_genomi	3	94	74	0	0	7	2.	1	5								4005	39925	39009	37647	32765	27176
c	7	80	8	9	8	4	6	6	9	0	154	126	90	73	42	27	196	95	58	73	03	47
GCA_0110133			40	2	1	1	12															
35.1_ASM1101	1	34	56	8	0	8	03															
333v1_genomi	2	98	10	.	3	4	46	1	5								4063	40491	39689	38721	34180	29067
c	2	21	8	0	6	4	.6	3	1	0	144	111	79	67	40	25	501	12	19	35	57	70

GCA_011013435.1_ASM1101343v1_genomic	111	244752	4062875	828.04	1683458	918549	106150.5	14	55	0	117	107	85	71	42	24	4065017	4059896	3998437	3901929	3379452	2729204
GCA_011013545.1_ASM1101354v1_genomic	149	234228	3992106	28.07	61916	13203	80192.9	19	72	0	163	139	106	85	46	24	3996558	3985523	3903405	3753562	3110118	2300777
GCA_011013585.1_ASM1101358v1_genomic	147	363478	3996100	28.06	87511	13458	117305.9	13	66	0	162	137	101	79	40	20	4000766	3989950	3911448	3747942	3131575	2474786
GCA_011013615.1_ASM1101361v1_genomic	100	229539	3904326	27.99	86026	19618	90292.9	16	52	0	115	99	79	70	45	29	3908999	3903717	3853474	3787973	3372183	2789367
GCA_011013635.1_ASM1101363v1_genomic	121	397810	3890002	28.0	5774	1506	102656.7	16	65	0	135	120	100	79	49	21	3894475	3889393	3836082	3672507	3205889	2239417

				2	4	9																
GCA_011013675.1_ASM1101367v1_genomic	67	467660	3898138	27.99	131014	28343	172791.6	9	31	0	82	66	53	48	33	26	3902882	3897531	3864942	3831960	3574544	3327838
GCA_011013685.1_ASM1101368v1_genomic	153	210518	3994757	28.05	53566	13136	65480.8	24	82	0	160	151	119	92	48	28	3996921	3993162	3913383	3714952	2962821	2221297
GCA_011013735.1_ASM1101373v1_genomic	43	495671	3889068	27.99	298129	63027	263273.7	6	17	0	55	39	30	28	23	19	3892642	3886366	3863232	3852090	3775822	3615770
GCA_011013765.1_ASM1101376v1_genomic	78	279137	3828431	27.97	110997	27261	116433.2	13	37	0	88	75	58	52	40	29	3831714	3826501	3784562	3744826	3531624	3148706
GCA_011013785.1_ASM1101	93	2823	3749	28	87	24	1168	12	47	0	101	88	72	64	44	20	3751711	3745291	3709367	3648909	3308856	2472452

378v1_genomi		49	05	.	4	1	18															
c			7	0	8	5	.3															
				1	4	5																
					1																	
				2	3	3																
GCA_0110138			38	8	0	1	14															
15.1_ASM1101		27	93	.	2	8	91															
381v1_genomi	6	87	77	0	7	7	85		3								3905	38896	38694	38516	36286	32243
c	7	56	3	1	5	6	.2	9	3	0	110	61	52	50	37	26	020	90	85	42	60	95
					1																	
				2	1	2																
GCA_0110138			38	8	5	0	13															
35.1_ASM1101		29	64	.	7	9	34															
383v1_genomi	8	42	05	0	2	3	91	1	3								3867	38604	38125	37648	34074	30778
c	7	03	7	1	9	4	.2	1	7	0	99	82	64	57	33	24	632	04	91	04	71	50
					1																	
				2	4	4																
GCA_0110138			39	7	2	3	19															
65.1_ASM1101		52	01	.	7	0	15															
386v1_genomi	5	11	01	9	5	7	34		2								3906	39004	38702	38520	37230	34682
c	6	74	2	9	9	3	.9	8	5	0	72	55	42	39	31	24	223	05	46	16	48	06
					7	1																
GCA_0110138			38		4	9																
85.1_ASM1101		24	95		3	3	98															
388v1_genomi	9	78	80	2	0	1	49	1	5								3900	38942	38393	37701	33660	28393
c	8	00	5	8	7	6	5	5	0	0	113	96	78	69	43	29	642	87	02	77	20	28

GCA_011013905.1_ASM1101390v1_genomic	38	693025	3904096	27.98	293372	56130	308143.5	5	17	0	50	36	28	26	23	21	3907747	3902814	3883690	3872343	3808725	3741531
GCA_011013935.1_ASM1101393v1_genomic	31	716393	4218512	27.94	305722	62929	368169.6	5	14	0	43	30	23	23	21	16	4222245	4217975	4203205	4203205	4165533	3970350
GCA_011013955.1_ASM1101395v1_genomic	130	311783	4003693	28.1	75106	13997	93759.3	17	65	0	139	128	105	75	47	23	4006568	4002299	3939922	3728672	3261566	2420455
GCA_011013965.1_ASM1101396v1_genomic	120	247497	3881761	28.02	67496	13831	93238.2	16	59	0	136	119	91	74	46	27	3886673	3881154	3805062	3683991	3261501	2576916
GCA_011013975.1_ASM1101397v1_genomic	226	152247	3877438	28.1	3337	8092	43234.8	34	127	0	238	223	156	106	50	16	3881202	3875126	3683798	3304798	2404021	1203631

GCA_011014015.1_ASM1101401v1_genomic	129	240455	3916745	828.05	260408	15817	74074.5	21	70	0	173	119	102	89	51	25	3928118	3909910	3864985	3775578	3177669	2230515
GCA_011014035.1_ASM1101403v1_genomic	114	249862	3888365	27.98	93721	17486	102180.2	14	55	0	134	108	82	75	38	25	3894198	3883918	3815882	3769425	3161519	2746251
GCA_011014055.1_ASM1101405v1_genomic	134	241545	3892993	28.01	53943	13588	77598.3	20	72	0	147	131	108	86	50	23	3897028	3890678	3823464	3668169	3085214	2120882
GCA_011014085.1_ASM1101408v1_genomic	153	184359	3887115	28.02	49502	11833	59145.6	25	85	0	168	148	122	96	55	24	3891786	3883470	3813417	3620435	2980262	1928202
GCA_011014095.1_ASM1101409v1_genomic	120	213050	3837067	28.09	57058	15749	72270.7	20	70	0	140	119	102	86	50	24	3843117	3836460	3781315	3672879	3075833	2162955

GCA_011014135.1_ASM1101413v1_genomic	118	233786	3882437	28.03	72883	18497	88958.2	16	61	0	134	115	89	75	48	23	3887587	3880219	3806805	3707393	3212072	2366318
GCA_011014155.1_ASM1101415v1_genomic	139	245846	3786350	28.16	55531	11560	81875.1	18	76	0	158	138	111	81	43	20	3791625	3785741	3692255	3462454	2856299	2015878
GCA_011014175.1_ASM1101417v1_genomic	53	637431	3896926	28	180675	43779	227692.2	7	24	0	92	49	39	38	29	23	3906975	3894342	3873881	3868484	3695878	3480474
GCA_011014195.1_ASM1101419v1_genomic	107	338841	3842034	28.07	59625	17414	99829.1	16	62	0	127	106	93	82	44	23	3847854	3841425	3805498	3729427	3089553	2336646
GCA_011014215.1_ASM1101421v1_genomic	151	158413	3813957	28.13	46617	13846	57785.7	25	86	0	169	149	117	97	51	22	3819146	3812675	3716062	3570324	2786070	1770028

GCA_011014225.1_ASM1101422v1_genomic	33	665013	3904128	27.98	379901	71011	347258.1	5	14	0	50	32	24	23	19	18	3909237	3903519	3885636	3880244	3816529	3774889
GCA_011014255.1_ASM1101425v1_genomic	122	195907	3851680	28.06	62021	16257	69647.4	21	70	0	143	121	100	87	49	28	3857788	3851071	3787726	3692497	3067791	2332368
GCA_011014265.1_ASM1101426v1_genomic	58	598787	3893034	28	166803	45547	201334.9	8	26	0	82	56	45	41	30	24	3900190	3891772	3860808	3831306	3650264	3414236
GCA_011014295.1_ASM1101429v1_genomic	111	243785	3879447	28.03	79908	16349	93463.1	15	56	0	128	110	89	73	41	27	3884428	3878838	3821608	3704348	3187200	2675104
GCA_011014305.1_ASM1101430v1_genomic	178	154968	3772540	28.1	3499	1089	48695.5	32	107	0	193	177	141	114	47	14	3777092	3771931	3662955	3469868	2350473	1197556

				8	3	0																
					1																	
				2	0	2																
GCA_0110143			38	8	7	3	14															
35.1_ASM1101		43	67	.	8	7	30															
433v1_genomi	9	06	65	0	6	6	39	1	4								3873	38647	38235	37302	33908	29210
c	3	43	3	7	9	0	.3	1	1	0	113	89	72	57	37	24	413	12	40	78	98	36
				2	4	1																
GCA_0110143			37	8	7	2	62															
45.1_ASM1101	1	19	03	.	4	8	11															
434v1_genomi	4	37	14	1	6	3	8.	2	8								3706	36992	36290	34779	27755	18420
c	3	35	1	1	3	6	2	3	1	0	154	138	113	93	50	22	291	82	55	92	12	66
					1																	
				2	5	3																
GCA_0110143			38	8	2	9	16															
75.1_ASM1101		29	18	.	1	6	80															
437v1_genomi	6	55	01	0	9	8	43		2								3820	38132	37837	37707	36192	32852
c	2	32	6	3	9	7	.9	8	8	0	72	56	43	41	33	24	899	69	02	86	60	03
					1																	
					1	2																
GCA_0110143			38		5	6	14															
95.1_ASM1101		34	79		7	9	38															
439v1_genomi	7	24	71	2	1	2	13	1	3								3883	38778	38437	37927	36077	29277
c	7	47	7	8	9	5	.4	0	8	0	88	74	62	54	42	22	047	67	34	54	94	86
GCA_0110144	5	36	38	2	1	4	16		2								3886	38828	38596	38322	36519	33506
15.1_ASM1101	9	94	84	8	3	1	50	9	9	0	68	57	48	44	33	25	862	43	84	42	31	01

441v1_genomi		59	15		6	3	24															
c			3		5	3	.5															
					3	5																
					2																	
				2	8	2																
GCA_0110144			41	7	0	3																
25.1_ASM1101	1	58	98	.	0	7	15															
442v1_genomi	0	81	91	9	0	8	79	1	5								4202	41976	41379	40432	37419	30147
c	5	43	0	7	5	0	96	3	0	0	116	103	81	67	48	27	341	82	53	98	36	37
				2	8	2																
GCA_0110144			37	8	8	1	10															
45.1_ASM1101		28	71	.	9	6	52															
444v1_genomi	8	19	65	0	4	1	90	1	4								3775	37709	37348	36888	33666	26981
c	4	38	1	6	0	6	.1	4	4	0	97	83	69	62	42	25	133	50	68	10	30	20
					1																	
				2	2	4																
GCA_0110144			38	7	2	1																
75.1_ASM1101		34	87	.	2	9	14															
447v1_genomi	6	32	79	9	8	4	26	1	3								3891	38849	38536	38485	36380	34236
c	3	21	6	9	7	5	75	1	0	0	75	59	47	46	34	28	373	65	68	32	68	83
					3																	
				2	8	9																
GCA_0110145		18	40	7	9	4	95															
45.1_ASM1101		28	06	.	5	6	23															
454v1_genomi	3	33	58	8	8	0	23		1								4010	40054	39737	39737	39429	38050
c	2	7	1	7	8	8	.3	2	0	0	45	30	18	18	16	12	473	55	02	02	53	89

GCA_011014565.1_ASM1101456v1_genomic	26	1308132	4006958	27.87	696731	142292	724897.4	2	7	0	36	26	16	14	13	9	4009954	4006958	3985307	3974883	3956089	3839277
GCA_011014595.1_ASM1101459v1_genomic	55	745602	3832786	28.03	131181	41630	252167.5	7	25	0	72	49	41	36	31	22	3837899	3828154	3810835	3778420	3681251	3349112
GCA_011014735.1_ASM1101473v1_genomic	40	1237787	4216150	27.87	484749	91264	634507.5	3	10	0	57	34	17	17	15	13	4221895	4211915	4178957	4178957	4145148	4073480
GCA_011014745.1_ASM1101474v1_genomic	58	512771	3899701	28.03	174024	38142	240725.3	6	25	0	67	57	44	37	30	22	3902622	3899092	3860915	3810116	3700316	3404041
GCA_011014775.1_ASM1101	52	4294	3900	28	21	42	2241	7	21	0	60	51	37	34	27	19	3903471	3900149	3860746	3841394	3739792	3435627

477v1_genomi		90	75	.	3	3	31															
c			8	0	0	1	.4															
				3	1	8																
					2																	
					3																	
				2	0	4																
GCA_0110148			38	8	0	0	27															
25.1_ASM1101		56	19	.	3	7	80															
482v1_genomi	4	62	74	0	9	9	20		1								3824	38166	37955	37807	36533	34117
c	4	31	3	3	5	4	.5	5	7	0	62	40	32	30	23	16	218	65	78	25	45	64
					2																	
					0	1																
				2	8	4	13															
GCA_0110149		20	39	8	5	3	35															
25.1_ASM1101		85	05	.	0	1	30															
492v1_genomi	2	08	15	0	8	8	1.										3907	39045	38783	38729	38729	38442
c	0	0	2	1	0	8	6	1	5	0	26	19	9	8	8	7	211	43	46	59	59	30
					7	1																
				2	0	4																
GCA_0110149		14	39	8	1	3	81															
55.1_ASM1101		19	04	.	6	1	51															
495v1_genomi	2	11	48	0	6	8	30										3906	39038	38776	38723	38538	37818
c	4	7	0	2	1	8	.9	2	6	0	30	23	13	12	11	9	539	71	74	04	49	01
GCA_0110149	1	19	38	2	6	1	77															
65.1_ASM1101	2	40	96	8	0	5	65	1	6								3898	38959	38309	37374	31133	22727
496v1_genomi	1	92	57	.	6	8	5	9	7	0	127	120	97	84	47	24	638	70	43	88	31	37

c			9	0	2	0																
				4	3	4																
				2	4	1																
GCA_0110149			36	8	4	2	53															
95.1_ASM1101	1	14	64	.	7	2	80															
499v1_genomi	5	84	15	1	1	5	7.	2	8								3668	36619	35853	34095	25432	15229
c	4	41	7	6	5	8	5	6	9	0	168	151	124	99	46	19	164	17	93	67	87	27
				2	4	1																
GCA_0110150			37	8	8	3	63															
15.1_ASM1101	1	18	83	.	5	7	49															
501v1_genomi	4	62	43	0	7	3	3.	2	8								3786	37786	37094	35202	27799	18120
c	8	94	1	4	0	9	8	2	0	0	160	142	114	90	47	20	638	54	40	78	44	22
					4																	
				2	1	8																
GCA_0110151			39	7	2	1	38															
95.1_ASM1101		67	83	.	6	7	06															
519v1_genomi	3	31	62	9	3	1	73		1								3987	39801	39592	39505	39077	36750
c	8	41	2	7	1	0	.2	4	2	0	51	33	23	22	20	13	584	42	12	99	44	91
					2																	
				2	2	7																
GCA_0110153			37	8	0	0	22															
05.1_ASM1101		41	99	.	2	4	11															
530v1_genomi	4	33	43	0	6	8	81		1								3803	37971	37774	37721	36919	36285
c	1	32	0	1	9	5	.8	7	8	0	54	38	29	28	23	21	211	82	55	05	52	16
GCA_0110153	1	14	37	2	4	1	54	2	9								3795	37860	36631	34286	26935	18007
15.1_ASM1101	7	57	91	8	5	0	24	5	2	0	186	165	124	93	47	22	661	51	74	45	60	97

531v1_genomi	3	42	92	.	4	1	4.															
c			7	0	2	1	7															
				3	3	3																
					4																	
				2	1	8																
GCA_0110153			39	7	2	1	43															
35.1_ASM1101		73	84	.	6	7	45															
533v1_genomi	3	48	54	9	3	1	76		1								3987	39818	39609	39471	38810	36912
c	7	17	1	7	4	0	.8	4	1	0	47	33	23	21	18	12	443	34	04	34	26	45
				2	7	2																
GCA_0110153			38	8	4	2	11															
95.1_ASM1101	1	28	54	.	2	7	17															
539v1_genomi	0	63	95	0	7	4	02	1	4								3859	38503	37865	37118	34155	26618
c	4	92	6	7	5	0	.4	4	9	0	119	98	74	63	46	25	679	24	79	16	87	45
					2																	
				2	7	6																
GCA_0110154			37	8	4	3	23															
15.1_ASM1101		40	96	.	0	0	62															
541v1_genomi	4	76	07	0	7	2	43		1								3800	37942	37741	37684	37340	35162
c	0	06	6	2	0	7	.7	6	8	0	54	37	28	27	25	19	184	07	42	26	42	87
					7	1																
				2	1	4																
GCA_0110154			42	7	1	1	57															
25.1_ASM1101		80	18	.	2	4	08															
542v1_genomi	2	54	40	9	4	6	15										4222	42178	42029	42029	41829	41201
c	2	64	4	5	0	0	.6	3	8	0	35	21	14	14	13	11	300	67	63	63	93	74

GCA_011015435.1_ASM1101543v1_genomic	39	484709	3907787	28.01	286544	60828	256765.4	6	17	0	49	37	27	26	23	18	3910953	3906725	3884875	3879532	3829880	3618448
GCA_011015475.1_ASM1101547v1_genomic	86	372118	3788508	28.04	101062	23336	125430.7	12	44	0	98	82	69	62	41	22	3792202	3785756	3750252	3703324	3341876	2632272
GCA_011015495.1_ASM1101549v1_genomic	62	304171	3794325	28.02	180987	41451	160583.7	8	29	0	74	58	45	41	34	23	3798005	3791796	3762640	3730712	3604575	3174027
GCA_011015505.1_ASM1101550v1_genomic	55	400018	3794755	28.02	215895	38257	199224.3	7	25	0	69	51	42	40	30	21	3798916	3792319	3772840	3759083	3588652	3269887
GCA_011015535.1_ASM1101	35	4076	3796	28	34	90	2836	5	14	0	49	32	23	22	20	17	3800227	3794254	3774189	3768599	3730255	3634336

553v1_genomi		03	12	.	8	7	38															
c			3	0	9	5	.1															
				2	7	4																
					4																	
					1																	
				2	1	3																
GCA_0110155			38	8	8	6	13															
45.1_ASM1101		30	50	.	9	0	75															
554v1_genomi	6	33	05	0	8	1	12	1	3								3853	38476	38217	37991	36067	32873
c	8	15	0	3	5	1	.6	1	3	0	81	64	52	49	37	28	953	08	38	33	62	51
					1																	
				2	4	3																
GCA_0110155			38	8	2	9	17															
55.1_ASM1101		33	61	.	2	6	07															
555v1_genomi	6	99	71	0	4	1	95		2								3866	38598	38254	38065	36306	33098
c	4	78	5	2	9	6	.2	8	8	0	82	61	47	44	33	24	946	46	28	40	28	34
					1																	
					3	4																
GCA_0110155			38		0	0	15															
95.1_ASM1101		32	71		8	0	63															
559v1_genomi	5	35	98	2	7	3	00		2								3876	38701	38492	38370	37321	33513
c	7	02	8	8	7	7	.5	9	8	0	72	54	43	41	36	25	485	19	35	83	64	78
GCA_0110156			38	2	7	1	85															
15.1_ASM1101	1	22	56	8	2	9	78															
561v1_genomi	1	74	21	.	6	3	7.	1	5								3860	38536	37829	36833	32856	27336
c	3	99	5	0	5	2	8	7	4	0	126	109	83	69	45	30	097	75	92	71	49	66

GCA_011015635.1_ASM1101563v1_genomic	25	947237	3900790	328.07	3419384	8130390	502209.5	3	9	0	38	22	15	14	13	12	3904478	3898923	3883522	3877559	3856539	3827741
GCA_011015655.1_ASM1101565v1_genomic	37	563200	3795627	28.02	285002	77522	293544.6	5	15	0	53	34	25	24	22	17	3800274	3793758	3773693	3768096	3720102	3542217
GCA_011015665.1_ASM1101566v1_genomic	57	304108	3794456	28.02	131934	38108	154099.9	9	29	0	74	53	43	42	34	24	3799286	3791902	3768154	3762559	3597611	3238066
GCA_011015685.1_ASM1101568v1_genomic	67	400018	3793011	28.02	121144	39526	151456.9	10	29	0	79	63	47	40	34	28	3796730	3790575	3748185	3693813	3593143	3396612
GCA_0110157	7	40	37	2	1	2	13	1	4	0	90	73	57	54	42	23	3797	37903	37465	37256	34886	28153

15.1_ASM1101	8	00	93	8	0	8	66	1	0								447	37	37	53	35	70
571v1_genomi		18	64	.	1	3	49															
c			0	0	0	3	.1															
				2	6	2																
					2																	
					2																	
				2	4	6																
GCA_0110157			37	8	6	3																
35.1_ASM1101		40	96	.	8	0	25															
573v1_genomi	4	01	37	0	6	4	49		1								3800	37945	37744	37624	37035	35352
c	0	55	6	2	4	7	20	6	7	0	52	37	28	26	23	18	099	07	42	37	65	10
				2	9	1																
GCA_0110157			37	8	0	9	12															
95.1_ASM1101		33	59	.	4	7	22															
579v1_genomi	9	68	54	0	6	0	88	1	4								3762	37576	37112	36611	33131	27656
c	5	49	5	1	8	1	.4	2	6	0	106	92	73	65	42	26	997	76	20	33	03	89
				2	3	1																
GCA_0110160			38	8	3	0	46															
45.1_ASM1101	1	14	59	.	7	5	98		1													
604v1_genomi	9	24	03	0	7	9	0.	3	0								3863	38537	37130	35317	26949	12600
c	1	54	9	5	9	2	8	2	4	0	204	184	133	109	58	15	045	35	83	57	55	61
				2	5	1																
GCA_0110160			38	8	2	2	62															
95.1_ASM1101	1	17	95	.	7	4	93															
609v1_genomi	4	87	40	0	1	9	1.	2	7								3899	38932	38043	36137	30663	20006
c	7	15	2	1	4	4	1	3	9	0	161	144	112	88	55	24	867	70	72	98	26	25

GCA_011017095.1_ASM1101709v1_genomic	48	735826	4142457	27.88	247126	56256	287568.7	6	19	0	67	45	36	31	26	20	4148498	4140538	4120992	4086964	3998547	3793214
GCA_011017125.1_ASM1101712v1_genomic	44	634239	3931843	28.12	235813	60834	278766.2	5	21	0	63	41	32	31	28	23	3937539	3929703	3908167	3903086	3858900	3667430
GCA_011017145.1_ASM1101714v1_genomic	88	311835	3905698	27.97	98097	21385	122036.1	12	45	0	100	85	72	64	43	24	3909499	3903712	3869072	3813400	3483804	2781542
GCA_011017175.1_ASM1101717v1_genomic	85	331115	3859490	28.03	93184	22935	124826.6	12	42	0	92	78	66	59	38	23	3862069	3853907	3826237	3777904	3378576	2781034
GCA_011017235.1_ASM1101723v1_genomic	163	165079	4058825	28.0	6545	1132	62116.1	23	88	0	172	158	128	95	46	27	4061684	4054937	3980435	3737982	2970064	2251276

GCA_011017245.1_ASM1101724v1_genomic	90	264841	3925636	528.01	199072	423887	101895.3	14	46	0	102	88	71	63	45	27	3929760	3924338	3885668	3825570	3528420	2859841
GCA_011017265.1_ASM1101726v1_genomic	115	261137	3912590	28.03	56725	18089	83650.6	18	66	0	129	113	93	83	49	22	3917155	3911291	3859909	3785474	3159738	2181877
GCA_011017275.1_ASM1101727v1_genomic	78	260074	3858288	28	99063	30615	106088.6	14	39	0	90	76	59	56	42	30	3862412	3856990	3819626	3799192	3574817	3104011
GCA_011017315.1_ASM1101731v1_genomic	64	378148	3858567	28	118253	47131	143117.2	11	30	0	82	60	44	42	34	28	3864363	3855872	3819290	3805624	3655820	3408770
GCA_011017375.1_ASM1101737v1_genomic	31	829676	3972825	28.1	5419	1458	435481.5	3	11	0	41	28	21	19	16	14	3975595	3971107	3956235	3941404	3899074	3821216

GCA_011017455.1_ASM1101745v1_genomic	46	778103	3794947	28.01	58262043	9668691	305404.1	5	17	0	61	40	29	28	21	20	3799270	3790516	3765593	3759817	3630192	3586913
GCA_011017465.1_ASM1101746v1_genomic	73	361875	3912119	27.99	138535	37645	143053.1	10	33	0	80	69	56	48	37	26	3914449	3909263	3877385	3821385	3645662	3229934
GCA_011017495.1_ASM1101749v1_genomic	102	263453	3880835	28.02	80208	20306	98762.4	15	52	0	115	100	79	69	45	26	3884615	3879525	3824663	3755250	3342175	2626476
GCA_011017505.1_ASM1101750v1_genomic	181	263601	3879349	28.06	41757	10625	62833.3	27	95	0	186	176	126	97	51	21	3881310	3876333	3724367	3513807	2751473	1711279
GCA_011017535.1_ASM1101	10	3589	3959	28	81	22	1128	14	50	0	108	99	77	65	48	24	3961828	3958008	3896689	3816114	3530275	2666846

753v1_genomi	2	89	68	.	0	3	29															
c			8	0	8	9	.8															
				1	0	2																
					1																	
				2	4	2																
GCA_0110175			38	8	1	6	15															
45.1_ASM1101		35	21	.	2	0	02															
754v1_genomi	7	01	83	0	8	4	22		3								3825	38171	37870	37574	34659	31050
c	3	67	0	3	6	6	.4	9	4	0	87	67	55	51	35	25	875	42	69	35	85	96
				2	7	1																
GCA_0110175			40	7	4	3	96															
75.1_ASM1101	1	30	21	.	7	5	99															
757v1_genomi	3	96	92	9	3	8	5.	1	6								4025	40195	39491	37811	31902	23566
c	3	70	3	4	0	3	6	5	8	0	145	129	103	81	44	21	593	47	99	10	27	89
				2	8	1																
GCA_0110175			37	8	2	8	11															
85.1_ASM1101		27	38	.	5	1	51															
758v1_genomi	9	55	93	0	1	2	26	1	4								3743	37366	36876	36483	32845	27594
c	0	35	7	3	5	5	.2	3	3	0	105	87	69	64	39	25	766	71	36	22	33	16
					1																	
					4	3																
GCA_0110176			38		7	4	14															
15.1_ASM1101		26	85		3	7	13															
761v1_genomi	6	00	55	2	4	1	25	1	3								3888	38835	38574	38394	36181	31384
c	5	47	6	8	3	6	.6	0	2	0	74	62	52	49	36	23	393	07	61	22	45	81
GCA_0110176	1	24	38	2	5	1	73	2	7	0	150	138	108	86	47	26	3893	38880	38073	36452	30090	22338

35.1_ASM1101763v1_genomic	43	7964	91359	8.06	59483	2888	298.9	1	4								783	44	43	90	88	33
GCA_011017645.1_ASM1101764v1_genomic	41	682795	3973312	28.1	040702	65275	339866.2	4	18	0	51	38	31	28	24	21	3976162	3971594	3956722	3938807	3863653	3770781
GCA_011017655.1_ASM1101765v1_genomic	69	379391	3901270	28.02	020403	29904	188579.3	7	30	0	75	67	49	46	32	16	3903329	3899882	3851551	3833395	3597612	2982923
GCA_011017685.1_ASM1101768v1_genomic	38	627401	3997192	28.04	50388	55692	352281.6	4	15	0	46	37	27	25	20	17	3999847	3996584	3971498	3958529	3867447	3745585
GCA_011017715.1_ASM1101771v1_genomic	53	410446	3858262	27.9	1776	4151	213496.9	7	24	0	66	51	40	37	30	21	3861879	3856802	3830147	3806844	3681033	3373064

				8	0	1																
					9																	
					1																	
				2	0	1																
GCA_0110177			39	8	2	8																
35.1_ASM1101	1	39	74	.	9	1	12															
773v1_genomi	0	20	54	0	3	2	45	1	4								3977	39732	39048	38408	33937	28863
c	3	70	8	2	5	5	20	3	8	0	114	101	76	67	39	25	776	38	00	44	22	16
				2	6	1																
GCA_0110177			41	7	3	3	88															
55.1_ASM1101	1	28	94	.	9	3	77															
775v1_genomi	3	59	67	9	4	4	5.	1	7								4198	41935	41042	39843	33993	23906
c	9	12	4	9	3	3	9	9	2	0	153	137	107	90	52	24	842	14	82	72	09	85
				2	5	1																
GCA_0110177			38	8	0	2	73															
65.1_ASM1101	1	24	23	.	1	9	70															
776v1_genomi	4	04	64	0	0	8	1.	2	7								3826	38187	37283	35599	29306	20078
c	9	26	3	9	4	9	1	1	6	0	157	142	109	86	47	22	200	21	87	17	41	04
				2	5	1																
GCA_0110177			42	7	0	3	56															
75.1_ASM1101	1	14	59	.	7	2	16															
777v1_genomi	6	75	32	9	3	8	0.	2	9								4264	42574	41517	39668	32273	21702
c	9	27	1	7	8	8	3	8	0	0	185	166	126	101	58	28	670	18	85	18	17	38
GCA_0110177		75	39	2	4	7	41															
85.1_ASM1101	3	69	73	8	1	6	46		1								3976	39722	39573	39420	38571	37422
778v1_genomi	3	21	94	.	1	6	28	4	1	0	42	30	23	21	16	13	492	24	52	96	76	40

c			1	1	3	9	.4															
					1	2																
					7																	
					1																	
					8	5																
GCA_0110178			39	2	4	4	24															
35.1_ASM1101		66	74	8	5	4	58															
783v1_genomi	5	72	18	.	2	5	74		2								3976	39724	39481	39322	38460	36411
c	2	62	9	1	1	2	.2	7	3	0	61	49	38	35	30	24	740	72	93	53	43	94
				2	6	1																
GCA_0110178			39	8	6	2	10															
45.1_ASM1101	1	28	60	.	3	7	17															
784v1_genomi	3	01	29	1	4	4	47	1	6								3963	39571	38577	36872	32375	23013
c	7	98	0	4	1	8	.3	5	5	0	147	132	97	75	46	20	063	66	86	44	61	38
					2																	
					1	2																
					4	9	14															
GCA_0110178		21	39	2	6	1	03															
75.1_ASM1101		46	72	8	8	6	97															
787v1_genomi	2	81	28	.	1	9	1.										3975	39705	39556	39504	39138	38398
c	1	6	7	1	6	4	5	1	4	0	32	18	11	10	8	6	255	69	97	81	61	46
				2	8	2																
GCA_0110178			40	8	9	0	13															
85.1_ASM1101		37	37	.	0	2	43															
788v1_genomi	9	22	02	0	5	0	25	1	4								4040	40355	39810	39002	35211	30450
c	4	97	0	8	7	6	.5	3	6	0	104	92	74	63	40	27	375	89	22	77	48	72

GCA_011017895.1_ASM1101789v1_genomic	104	257507	3950423	28.14	94911	18434	104695.6	14	50	0	113	101	82	67	43	23	3953003	3948705	3896991	3788234	3415314	2675493
GCA_011017935.1_ASM1101793v1_genomic	191	168244	3977154	28.09	38443	9784	50365.1	30	109	0	195	189	143	107	52	17	3978554	3975762	3825825	3568094	2716094	1422659
GCA_011017955.1_ASM1101795v1_genomic	101	342607	4050795	28.06	117786	20074	133696.6	11	43	0	107	96	73	60	38	23	4052937	4047566	3983884	3891101	3537635	2976071
GCA_011017965.1_ASM1101796v1_genomic	73	320754	3882563	27.98	121541	31397	135755.2	11	32	0	81	69	55	48	35	26	3885041	3879818	3844215	3792124	3602804	3279204
GCA_011017975.1_ASM1101797v1_genomic	95	490668	4201766	27.9	8253	2569	147608.1	12	49	0	106	92	75	70	49	24	4205108	4199949	4151961	4117392	3795306	2889216

GCA_011017995.1_ASM1101799v1_genomic	249	155165	3827754	828.13	926653	86870	39131.1	40	143	0	266	243	170	120	44	11	3833113	3823096	3603648	3261151	2028321	906881
GCA_011018275.1_ASM1101827v1_genomic	33	725514	3888513	27.98	269624	91351	319320.7	5	15	0	40	30	22	22	20	18	3890949	3886409	3868138	3868138	3836301	3766211
GCA_011018285.1_ASM1101828v1_genomic	53	525698	3996495	28.03	172239	62585	220668.3	7	23	0	69	47	36	35	29	23	4000784	3992606	3965974	3960709	3873096	3649774
GCA_011018295.1_ASM1101829v1_genomic	131	263429	4318329	27.89	73974	16665	96099.6	18	61	0	148	118	92	82	49	34	4323914	4309996	4253471	4176637	3653382	3172210
GCA_011038385.1_ASM1101838v1_genomi	124	295714	396782	27.	646	169	89524.	19	61	0	131	115	94	76	54	24	3969952	3961626	3909866	3774442	3447034	2334558

c			2	9	1	0	6															
				6	5	4																
					1																	
				2	4	2																
GCA_0110183			40	8	0	0	13															
95.1_ASM1101		32	64	.	3	1	55															
839v1_genomi	8	17	37	0	3	6	35	1	3								4066	40629	40176	39417	35971	30863
c	6	91	8	4	8	4	.6	1	9	0	92	84	68	57	36	22	520	76	99	55	91	75
					4	1																
				2	5	1																
GCA_0110185			38	8	2	8	39															
25.1_ASM1101		74	86	.	2	9	68															
852v1_genomi	4	47	50	0	1	2	45		1								3890	38850	38478	38326	38145	37200
c	0	00	3	2	4	5	.2	4	1	0	54	38	19	17	16	13	475	55	76	30	55	61
					1																	
				2	8	4																
GCA_0110185			39	8	7	6	21															
75.1_ASM1101		52	18	.	1	4	92															
857v1_genomi	5	67	01	1	1	3	25		2								3920	39158	38829	38720	37601	34767
c	4	54	8	3	3	4	.5	7	5	0	62	51	39	37	30	23	613	81	61	61	71	78
				2	7	2																
GCA_0110185			40	8	6	1	10															
95.1_ASM1101		25	63	.	3	5	12															
859v1_genomi	9	76	68	0	8	3	13	1	4								4066	40624	40226	39440	35949	30003
c	7	65	5	3	9	1	.7	5	9	0	104	95	80	68	46	30	063	02	47	90	98	01
GCA_0110186	1	26	39	2	8	1	11	1	4	0	113	100	80	61	38	25	3905	38996	38445	36956	33214	28168

25.1_ASM1101	0	91	03	8	8	7	05	3	8								906	37	27	54	52	65
862v1_genomi	6	83	62	.	0	5	87															
c			9	0	1	3	.8															
				3	4	5																
					7	2																
GCA_0110186			38		5	3	11															
75.1_ASM1101		31	86		0	2	48															
867v1_genomi	9	02	22	2	2	4	37	1	4								3889	38832	38287	38000	34220	28130
c	3	17	6	8	8	5	.5	3	6	0	103	89	70	65	42	26	327	85	16	48	85	93
					4	1																
				2	0	0																
GCA_0110187			41	7	0	6	39															
25.1_ASM1101		73	35	.	2	0	03															
872v1_genomi	3	04	21	8	6	9	22		1								4141	41312	41172	41032	40671	39655
c	5	45	1	7	2	3	.3	4	2	0	57	29	22	20	18	15	632	85	84	48	19	90
					1																	
				2	0	2																
GCA_0110187			38	7	0	0	12															
95.1_ASM1101		33	90	.	7	2	79															
879v1_genomi	9	84	70	9	9	5	59	1	4								3896	38871	38320	37438	34526	29805
c	3	39	9	7	0	2	.5	1	2	0	114	88	70	58	39	25	849	45	93	12	91	49
				2	1	3																
GCA_0110188			40	7	4	1	16															
15.1_ASM1101		34	48	.	0	9	16															
881v1_genomi	7	30	27	8	6	3	05		3								4051	40439	40036	39592	36883	32831
c	8	79	2	8	5	4	.1	9	4	0	88	72	57	51	35	24	467	58	58	22	65	47

GCA_011018895.1_ASM1101889v1_genomic	76	445844	3858947	28.19	7118475	25618	147397.2	11	34	0	85	74	57	50	35	26	3861604	3857811	3800186	3749207	3504972	3199956
GCA_011018945.1_ASM1101894v1_genomic	84	370715	4064172	28.03	94743	22972	124305.2	13	42	0	91	82	72	61	39	29	4066542	4062781	4038786	3958884	3602633	3206843
GCA_011018995.1_ASM1101899v1_genomic	52	374034	3958258	27.98	182421	58923	193740.9	8	21	0	70	47	35	33	26	22	3963247	3954783	3923431	3913231	3798731	3657845
GCA_011019005.1_ASM1101900v1_genomic	67	336397	3824374	28.02	141486	34595	158332.4	9	29	0	77	62	47	42	34	25	3827299	3820515	3777829	3746711	3600912	3300616
GCA_011019055.1_ASM1101	78	5111	3943	28	11	26	1713	9	36	0	91	76	63	53	38	23	3946924	3941659	3910254	3834869	3602253	3095935

905v1_genomi		73	13	.	8	6	78															
c			5	1	7	9	.2															
				3	7	7																
					1																	
					8	2																
GCA_0110190			43	2	8	0	10															
65.1_ASM1101	1	26	17	7	3	1	52															
906v1_genomi	2	35	98	.	9	8	51	1	5								4324	43113	42363	41288	37541	31023
c	7	86	2	9	3	5	.6	5	3	0	148	117	86	71	47	29	852	23	08	77	67	46
					1																	
				2	9	3																
GCA_0110190			39	8	6	0	26															
95.1_ASM1101		61	71	.	6	0	94															
909v1_genomi	5	23	67	1	7	4	50		2								3973	39692	39415	39142	37361	33286
c	9	32	0	1	1	1	.8	6	5	0	67	55	45	41	30	18	995	60	14	47	66	57
					1																	
				2	1	5																
GCA_0110191			38	8	9	1	16															
25.1_ASM1101		30	58	.	3	6	25															
912v1_genomi	5	93	82	0	8	5	35		2								3862	38534	38361	38245	37225	34977
c	6	23	4	3	6	0	.6	9	7	0	69	49	41	39	33	27	767	83	95	64	60	49
				2	3	1																
GCA_0110191			39	7	1	2																
65.1_ASM1101		76	61	.	0	8	36															
916v1_genomi	3	76	56	9	6	8	16		1								3967	39574	39400	39347	38877	37983
c	5	53	2	8	6	3	26	5	2	0	57	29	21	20	17	14	417	78	07	25	13	74

GCA_011019215.1_ASM1101921v1_genomic	28	934089	4007591	27.87	2696096	8141766	620163.8	3	8	0	41	27	17	15	14	11	4011528	4006982	3985331	3974837	3956043	3867307
GCA_011019415.1_ASM1101941v1_genomic	111	254624	3996170	28.11	64277	17066	95344.2	17	63	0	120	110	95	82	42	25	3999022	3995562	3950760	3859019	3180835	2514364
GCA_011019425.1_ASM1101942v1_genomic	23	1953568	3890552	27.98	1953568	122316	1139949.1	1	6	0	32	20	12	12	10	9	3893254	3888740	3870400	3870400	3842008	3807413
GCA_011019455.1_ASM1101945v1_genomic	114	299436	3857541	28.04	75057	15536	99179	15	60	0	134	109	93	79	40	23	3863217	3854225	3812835	3714953	3081121	2442790
GCA_011019525.1_ASM1101	80	1904	3859	28	10	25	1021	14	41	0	99	76	62	59	42	29	3865210	3856883	3828373	3808919	3520158	3058861

952v1_genomic		84	326		31611	977	64.5															
GCA_011019625.1_ASM1101962v1_genomic	80	361069	3822552	28.02	459272	26709	149858.5	9	36	0	89	75	59	52	37	23	3825254	3818693	3771866	3721257	3468600	2987790
GCA_011019745.1_ASM1101974v1_genomic	49	519677	3864312	28.04	522116	432011	275878	5	20	0	72	43	35	32	26	17	3871282	3860241	3842950	3818851	3704235	3368457
GCA_011019775.1_ASM1101977v1_genomic	23	1302612	3903575	27.98	86151	20296	711520.1	2	9	0	39	22	15	14	13	12	3908743	3902966	3888398	3883001	3861708	3823404
GCA_011019895.1_ASM1101989v1_genomic	69	336181	3903930	27.9	1400	3731	167437.1	9	29	0	83	67	51	44	32	24	3908265	3902648	3863768	3812104	3620344	3321110

GCA_011019915.1_ASM1101991v1_genomic	75	467371	3900356	928	1098636	233633	150954.7	11	39	0	92	74	61	57	43	25	3905658	3899747	3871123	3849336	3631192	2964257
GCA_011019945.1_ASM1101994v1_genomic	55	524887	3896534	28.03	140299	41725	207064	8	25	0	63	52	42	40	31	23	3899182	3894625	3869078	3858707	3707878	3429170
GCA_011019975.1_ASM1101997v1_genomic	53	495809	3901745	27.99	157207	43208	188340.4	8	27	0	72	52	43	41	33	26	3907454	3901136	3880521	3867901	3737862	3507417
GCA_011020005.1_ASM1102000v1_genomic	39	575432	4065618	28.02	251905	97485	280043	6	17	0	51	38	31	29	21	18	4069384	4065010	4049628	4036968	3878609	3786121
GCA_0110200	1	28	40	2	7	2	89	1	5	0	115	103	79	70	48	32	4033	40287	39523	38844	34915	29210

15.1_ASM1102001v1_genomic	05	0114	30284	8.0772	94127	02201	056.5	8	4								683	94	67	91	90	15
GCA_011020075.1_ASM1102007v1_genomic	113	260611	3855555	8.042	239610	99202	100738.9	14	54	0	130	107	87	68	45	23	3861043	3851176	3801465	3662521	3284317	2500708
GCA_011020115.1_ASM1102011v1_genomic	87	423815	3828287	8.082	444744	0170	148789.6	10	40	0	97	86	68	58	34	25	3831692	3827757	3783769	3714076	3328104	3029121
GCA_011020175.1_ASM1102017v1_genomic	177	259936	3869734	8.062	950912	94672	71430.7	21	90	0	183	170	124	85	42	20	3871905	3864777	3731684	3440298	2685948	1902996
GCA_011020195.1_ASM1102019v1_genomic	70	432283	3887613	7.98	4506	6881	167887.4	9	31	0	78	67	56	50	32	25	3890199	3885401	3858463	3812505	3540144	3305496

GCA_011020255.1_ASM1102025v1_genomic	47	309451	3901669	27.99	233742	52764	187019	8	22	0	61	46	38	34	27	22	3906242	3901060	3881150	3856178	3729225	3515359
GCA_011020275.1_ASM1102027v1_genomic	51	588343	3888783	27.99	206315	45348	246507.9	6	22	0	59	49	38	35	29	19	3891347	3887473	3861129	3840882	3757222	3359963
GCA_011020285.1_ASM1102028v1_genomic	87	276914	3883670	27.99	87847	24985	114023.9	13	42	0	94	84	66	58	41	28	3886062	3881560	3832007	3780332	3489057	3009643
GCA_011020525.1_ASM1102052v1_genomic	88	248341	3890941	28.01	86575	20954	113542	12	47	0	102	87	75	68	41	24	3895460	3890334	3855133	3809028	3382176	2779451
GCA_011020535.1_ASM1102053v1_genomic	55	550711	4137433	27.8	2018	4672	224560.6	7	25	0	70	52	43	38	31	24	4142381	4135514	4112154	4073821	3959585	3691514

				8	4	3																
					9																	
					1																	
				2	3	2																
GCA_0110205			41	7	3	2	18															
95.1_ASM1102		54	31	.	6	7	27															
059v1_genomi	8	45	63	8	6	4	74		3								4136	41297	40855	39943	36872	34064
c	3	21	3	9	0	1	.6	9	6	0	96	80	64	52	34	26	150	14	96	65	67	04
					1																	
				2	5	3																
GCA_0110206			38	8	7	4	20															
55.1_ASM1102		60	71	.	1	6	60															
065v1_genomi	5	09	16	1	7	6	07		2								3873	38705	38419	37844	35689	34368
c	8	17	8	2	9	0	.4	8	6	0	65	57	47	39	28	24	546	60	36	88	66	96
					1																	
				2	3	2																
GCA_0110206			38	7	9	9	14															
65.1_ASM1102		29	33	.	3	6	41															
066v1_genomi	7	68	39	9	9	7	15	1	3								3836	38311	37837	37547	35529	32271
c	4	25	2	8	8	8	.7	0	3	0	85	70	53	49	36	26	926	47	37	53	94	69
					2	1																
GCA_0110207			34	2	9	0	41															
35.1_ASM1102	1	18	36	8	6	0	54		1													
073v1_genomi	8	73	28	.	5	0	4.	3	1								3440	34344	33176	30973	19845	68742
c	7	81	8	3	5	1	5	5	2	0	199	184	143	112	44	8	104	87	49	46	06	5
GCA_0110208	9	30	38	2	9	2	12	1	4	0	100	87	70	60	41	24	3860	38534	38116	37416	34185	28306

15.1_ASM1102	3	61	58	8	7	4	54	1	4								661	38	33	51	93	72
081v1_genomi		22	08	.	0	1	46															
c			2	0	9	5	.6															
				4	3	4																
					1																	
				2	1	2																
GCA_0110208			38	7	1	5	13															
75.1_ASM1102		31	00	.	0	8	77															
087v1_genomi	7	99	16	9	2	5	95	1	3								3804	37965	37727	37424	34566	32394
c	0	79	3	9	5	4	.2	0	3	0	84	65	54	50	34	27	147	99	35	94	33	48
					1																	
				2	8	4																
GCA_0110208			40	8	9	3	19															
95.1_ASM1102		46	62	.	5	7	18															
089v1_genomi	5	69	46	0	2	3	89		2								4065	40618	40295	39935	38099	35493
c	9	90	5	3	0	6	.8	8	7	0	67	58	46	41	31	24	156	57	66	16	54	39
				2	7	1																
GCA_0110209			37	8	3	9	87															
15.1_ASM1102	1	23	28	.	1	3	18															
091v1_genomi	1	49	52	1	7	1	5.	1	5								3735	37223	36598	35802	31921	24752
c	3	17	9	2	5	9	3	6	3	0	140	104	81	69	45	26	398	72	76	51	93	98
				2	8	2																
GCA_0110209			38	8	8	3	15															
65.1_ASM1102		41	74	.	1	1	00															
096v1_genomi	8	51	14	0	5	0	99	1	4								3877	38719	38422	37742	34041	29304
c	5	95	8	1	0	2	.6	0	2	0	97	82	71	61	38	25	729	93	48	23	66	49

GCA_011020995.1_ASM1102099v1_genomic	84	247488	3869824	28.02	105185	24130	111902.3	13	44	0	95	82	71	59	43	24	3873090	3868514	3841662	3749941	3482652	2855688
GCA_011021125.1_ASM1102112v1_genomic	82	404043	4318041	27.89	140422	43046	161740.8	10	32	0	100	72	48	44	39	29	4323927	4311521	4258684	4230395	4155407	3780602
GCA_011021215.1_ASM1102121v1_genomic	115	248435	3914870	28.03	56729	18946	80797.5	19	63	0	134	111	92	83	54	26	3920860	3911955	3868117	3807280	3340880	2374203
GCA_011021325.1_ASM1102132v1_genomic	91	544711	3956052	27.98	108227	26801	159749.9	12	38	0	110	85	67	53	38	26	3961475	3951968	3902258	3806976	3563798	3152729
GCA_011021335.1_ASM1102133v1_genomi	122	381697	395423	28.	730	162	101632	17	60	0	146	117	91	73	48	25	3960976	3950865	3865448	3735871	3324590	2481314

c			9	0	2	2	.4															
				1	1	1																
				2	9	1																
GCA_0110213			37	8	2	7	11															
75.1_ASM1102	1	29	87	.	8	9	60															
137v1_genomi	0	20	37	0	3	8	29	1	4								3790	37849	37124	36020	31839	26341
c	3	79	9	4	0	4	.8	2	7	0	112	100	75	60	36	22	007	98	40	37	36	90
					1																	
				2	0	3																
GCA_0110214			39	8	2	0	94															
15.1_ASM1102		17	21	.	6	9	60															
141v1_genomi	8	79	03	0	2	9	6.	1	4								3926	39197	38869	38425	36724	30287
c	2	70	1	2	8	7	1	5	3	0	99	80	65	59	48	30	334	32	86	05	69	96
					1																	
				2	5	3																
GCA_0110214			40	8	3	4	21															
35.1_ASM1102		58	56	.	7	5	93															
143v1_genomi	6	24	93	0	6	0	45		2								4059	40563	40215	39855	38148	35335
c	1	96	9	4	4	3	.6	8	6	0	69	60	47	42	31	23	534	31	48	68	78	79
					1																	
					7	2																
GCA_0110214			44	2	0	6	28															
95.1_ASM1102		77	46	8	6	3	43															
149v1_genomi	6	17	91	.	9	9	64		3								4449	44463	44280	43762	40526	37105
c	7	54	9	1	9	9	.8	6	0	0	74	66	58	51	31	22	297	11	42	49	80	19
GCA_0110215	4	41	38	2	2	5	22	7	1	0	58	43	33	32	24	19	3887	38830	38572	38500	37030	35310

15.1_ASM1102	5	24	84	7	3	5	58		9								780	30	77	18	16	42
151v1_genomi		03	40	.	2	4	77															
c			2	9	6	9	.2															
				9	9	5																
					7																	
					3	1																
				2	9	5																
GCA_0110215			39	7	8	2	40															
35.1_ASM1102		80	46	.	3	6	87															
153v1_genomi	3	30	71	9	1	7	09		1								3951	39432	39214	39214	38698	38124
c	2	64	0	7	5	9	.5	4	0	0	51	27	18	18	15	13	862	35	98	98	83	09
					2																	
				2	3	6																
GCA_0110215			43	7	5	2	29															
45.1_ASM1102		65	20	.	3	3	77															
154v1_genomi	6	67	19	8	2	6	55		1								4325	43131	42585	42397	41699	39094
c	8	97	6	9	9	6	.6	6	9	0	85	57	33	30	26	19	658	77	92	24	51	12
					1																	
				2	5	6																
GCA_0110215			38	7	8	6	17															
65.1_ASM1102		38	72	.	7	1	03															
156v1_genomi	4	21	87	9	4	9	86		2								3877	38708	38482	38398	37464	36704
c	8	02	5	9	0	4	.8	9	3	0	64	45	34	33	28	26	660	88	69	46	22	04
GCA_0110216		90	37	2	2	7	38															
15.1_ASM1102	3	22	88	8	8	2	24		1								3791	37882	37684	37633	37054	35670
161v1_genomi	5	11	99	.	2	5	72	4	5	0	43	34	25	24	21	17	445	94	84	99	73	42

c			5	0	1	6	.6															
				4	8	5																
					6																	
					4	1																
				2	8	4																
GCA_0110216			39	7	7	5	53															
55.1_ASM1102		90	60	.	0	8	83															
165v1_genomi	2	80	77	9	2	5	87										3966	39573	39393	39393	39074	38499
c	8	18	9	7	4	3	.6	3	9	0	49	23	15	15	13	11	395	05	68	68	98	92
					4	1																
				2	1	5																
GCA_0110217		12	38	7	4	0	64															
35.1_ASM1102		75	89	.	8	7	03															
173v1_genomi	2	30	04	9	6	1	27										3892	38883	38700	38700	38189	37410
c	4	5	5	8	3	6	.7	3	8	0	35	23	15	15	12	10	108	44	04	04	26	37
					3																	
				2	0	9																
GCA_0110217			39	8	6	5	34															
55.1_ASM1102		73	03	.	2	2	71															
175v1_genomi	3	73	66	0	7	2	07		1								3905	39030	38768	38680	38279	37246
c	3	04	1	2	4	5	.1	5	3	0	40	32	22	21	19	16	967	52	55	19	73	43
					1	3																
GCA_0110217			38		3	5	14															
65.1_ASM1102		39	96		0	7	48															
176v1_genomi	6	05	72	2	7	2	87	1	3								3900	38954	38556	38106	36548	33321
c	6	70	2	8	5	3	.8	1	1	0	79	64	50	44	35	26	573	40	26	18	07	58

GCA_011021895.1_ASM1102189v1_genomic	86	209167	3898767	28	2103795	25756	100714.6	14	43	0	105	82	66	60	44	29	3904612	3896144	3847697	3802711	3551664	3025847
GCA_011021905.1_ASM1102190v1_genomic	73	291469	3894256	28	113566	35011	127521.2	11	37	0	90	72	59	55	39	25	3899682	3893649	3858758	3835769	3581808	3018218
GCA_011021915.1_ASM1102191v1_genomic	82	334175	3901678	27.99	131288	24467	143708.1	10	37	0	99	81	64	52	36	25	3906988	3901069	3854775	3773352	3500100	3138891
GCA_011021955.1_ASM1102195v1_genomic	50	623652	3872300	28.05	175812	55407	234601.8	7	22	0	66	44	36	34	29	23	3877160	3867669	3850350	3839958	3768172	3545065
GCA_0110220	1	20	39	2	3	9	54	2	1	0	205	186	138	104	53	18	3969	39614	38258	35682	27544	15278

75.1_ASM1102	9	42	65	7	8	9	52	9	0								885	79	38	73	56	67
207v1_genomi	2	80	58	.	1	6	1.		5													
c			9	1	9	6	9															
				1	2																	
					1																	
				2	4	3																
GCA_0110220			38	8	0	8	14															
85.1_ASM1102		33	55	.	8	1	86															
208v1_genomi	5	65	18	0	1	8	32	1	2								3859	38545	38388	37932	36810	33728
c	5	42	2	3	3	7	.4	0	8	0	71	54	47	41	34	25	989	73	73	60	78	77
				2	9	2																
GCA_0113631			39	8	8	8	11															
75.1_ASM1136		27	12	.	3	6	64															
317v1_genomi	8	11	33	0	9	4	03	1	4								3918	39096	38744	38390	35963	30307
c	3	51	1	3	8	8	.6	2	2	0	102	79	65	60	44	28	049	72	98	80	58	07
				2	9	2																
GCA_0113894			39	8	6	1	12															
95.1_ASM1138	1	34	36	.	1	6	04															
949v1_genomi	0	96	27	0	7	5	90	1	5								3940	39325	38672	38093	33898	25779
c	8	12	4	8	3	5	.6	3	0	0	122	103	78	69	43	21	268	35	21	53	44	17
					3	3																
				2	9	9	39															
GCA_0140686		39	39	8	1	1	06															
15.1_ASM1406		12	18	.	2	2	45															
861v1_genomi		36	28	1	3	3	4.										3918	39182	39182	39123	39123	39123
c	2	3	9	8	6	6	9	1	1	0	2	2	2	1	1	1	289	89	89	63	63	63

GCA_014805065.1_ASM1480506v1_genomic	25	1430429	4064247	28.02	3808975	399206	788649.4	2	8	0	25	24	16	15	14	12	4064247	4063390	4046504	4039748	4019668	3942378
GCA_014805085.1_ASM1480508v1_genomic	21	2832195	4108059	27.8	2832195	206641	2036107.7	1	4	0	21	17	10	10	8	6	4108059	4105725	4089710	4089710	4064460	4006986
GCA_014805095.1_ASM1480509v1_genomic	22	914022	4064564	28.02	548706	219458	566892.3	3	7	0	22	21	14	14	13	11	4064564	4063814	4048933	4048933	4028853	3951364
GCA_014805105.1_ASM1480510v1_genomic	81	292112	4059968	28.01	95651	31677	117840.8	13	42	0	81	77	63	59	46	26	4059968	4056985	4024805	3996361	3768198	2979352
GCA_0148051	2	80	40	2	5	1	52	3	8	0	25	23	15	15	14	11	4067	40662	40510	40510	40307	39268

45.1_ASM1480	5	91	67	8	2	2	34										487	20	31	31	13	30
514v1_genomi		63	48	.	2	7	60															
c			7	0	3	9																
				2	6	5																
					9	5																
					6	1																
				2	9	1																
GCA_0148051			40	8	3	9	61															
65.1_ASM1480		96	63	.	2	8	13															
516v1_genomi	2	35	96	0	8	2	31										4063	40625	40411	40411	40210	38732
c	6	07	7	4	6	7	.5	3	7	0	26	24	14	14	13	9	967	99	48	48	68	61
					4	1																
					3	5																
GCA_0148051			40		2	6																
85.1_ASM1480		73	62		7	5	44															
518v1_genomi	2	97	36	2	8	9	55										4062	40609	40451	40316	39772	38999
c	9	31	5	8	1	5	29	4	9	0	29	27	19	17	14	12	365	89	10	27	99	02
					4																	
				2	3	9																
GCA_0148051		13	40	8	8	6	67															
95.1_ASM1480		58	62	.	7	4	39															
519v1_genomi	2	28	15	0	5	8	56		1								4062	40607	40455	40306	40096	39323
c	8	6	2	1	8	5	.3	3	0	0	28	26	18	16	15	13	152	77	84	66	07	15
GCA_0148052		80	41	2	5	2	53															
15.1_ASM1480	2	85	20	7	1	5	26										4120	41196	41056	41056	40697	39924
521v1_genomi	2	77	53	.	9	1	04	3	8	0	22	21	13	13	11	9	534	88	13	13	13	24

c			4	9	0	3	.8															
				4	2	6																
					2	9																
					2																	
					1	2																
				2	1	5	13															
GCA_0148052		21	41	7	5	1	46															
35.1_ASM1480		15	29	.	1	8	20															
523v1_genomi	1	10	11	9	0	0	1.										4129	41279	41134	41134	41134	40848
c	7	8	0	7	8	4	8	1	5	0	17	15	7	7	7	6	110	31	51	51	51	18
					5																	
				2	2	9																
GCA_0148052			40	8	2	6	52															
55.1_ASM1480		80	65	.	3	4	94															
525v1_genomi	2	91	17	0	6	8	71										4065	40644	40476	40476	40275	39236
c	4	64	8	1	9	5	.7	3	8	0	24	23	15	15	14	11	178	28	50	50	70	09
					6	1																
				2	4	4																
GCA_0148052			40	8	0	8	53															
75.1_ASM1480		80	75	.	3	8	62															
527v1_genomi	4	84	11	0	8	5	78										4075	40640	40488	40488	40129	39011
c	2	77	6	1	0	7	.1	3	8	0	42	24	15	15	13	10	116	72	55	55	55	45
GCA_0148053			41	2	3	1	59															
05.1_ASM1480	1	20	38	7	7	1	66		1													
530v1_genomi	9	13	59	.	5	3	7.	2	0								4138	41344	40051	37619	29051	16990
c	6	03	0	8	2	9	5	7	2	0	196	189	138	105	54	18	590	27	95	25	13	82

GCA_014872905.1_ASM1487290v1_genomic	77	293927	4155425	127.99	3135352	031474	139890	11	37	0	77	71	58	53	42	24	4155425	4150838	4123312	4083830	3901402	3266401
GCA_016085375.1_PDT000162924.3_genomic	40	465422	3816972	27.98	243674	85709	248097.2	6	16	0	44	33	28	26	20	17	3818549	3812016	3800800	3785266	3681565	3550665
GCA_016085455.1_PDT000162920.3_genomic	46	409398	3828635	27.98	228076	54954	217504.2	7	19	0	49	38	33	32	23	19	3829895	3822981	3811928	3806409	3641675	3497386
GCA_016085475.1_PDT000162923.3_genomic	35	465440	3829903	27.98	268768	91689	283811.1	6	14	0	37	29	24	24	18	17	3830725	3825620	3814567	3814567	3704533	3669189
GCA_0160854	3	46	38	2	3	1	29	5	1	0	33	24	19	19	16	15	3832	38262	38150	38150	37563	37217

95.1_PDT0001	1	53	31	7	0	4	92		2								024	69	71	71	91	65
62918.3_geno		14	22	.	4	0	59															
mic			5	9	2	2	.5															
				8	2	2																
					0	8																
				2	2																	
GCA_0160855			37	8	7	6	33															
15.1_PDT0001	2	97	73	.	4	1	75		1													
62922.3_geno	8	40	64	0	0	3	2.	4	5								3778	37615	35060	30636	19978	99266
mic	8	3	8	9	2	6	1	0	5	0	299	271	175	112	44	15	163	69	20	15	21	3
					2																	
				2	4	9																
GCA_0160855			38	7	9	6	26															
35.1_PDT0001		46	21	.	2	3	89															
62917.3_geno	3	54	08	9	5	8	21		1								3822	38161	38047	38047	37202	36772
mic	5	06	1	8	2	0	.1	6	4	0	39	28	23	23	18	17	658	25	64	64	53	42
					2																	
				2	4	7																
GCA_0160855			38	7	3	8	27															
55.1_PDT0001		45	21	.	5	1	57															
62921.3_geno	3	24	35	9	1	8	31		1								3825	38178	38077	37945	36794	36096
mic	7	45	7	6	0	5	.9	6	4	0	51	32	27	25	18	16	766	15	80	55	95	04
GCA_0160856			38	2	3	9	30															
25.1_PDT0001		46	27	7	0	1	01															
62916.3_geno	3	52	16	.	4	7	84		1								3828	38222	38108	38108	37210	36780
mic	3	97	8	9	1	7	.6	5	3	0	36	26	21	21	16	15	428	12	51	51	60	49

				7	382	8																
GCA_016085715.1_PDT000162915.2_genomic	39	464671	3880271	27.99	43674	63274	268014.7	6	16	0	43	33	28	28	20	18	3881885	3876139	3864778	3864778	3726415	3640609
GCA_016798345.1_ASM1679834v1_genomic	1	3815652	3815652	28.17	3815652	3815652	3815652	1	1	0	1	1	1	1	1	1	3815652	3815652	3815652	3815652	3815652	3815652
GCA_016838665.1_ASM1683866v1_genomic	1	4090796	4090796	28.15	4090796	4090796	4090796	1	1	0	1	1	1	1	1	1	4090796	4090796	4090796	4090796	4090796	4090796
GCA_016989565.1_ASM1698956v1_genomic	49	631623	3865776	27.9	2060	4220	293335.1	5	22	0	54	46	38	34	29	20	3867597	3863884	3845371	3815210	3725581	3409395

				6	3	0																
					6																	
					3	1																
				2	9	4																
GCA_0169896			42	7	0	0	44															
05.1_ASM1698		86	78	.	4	1	74															
960v1_genomi	4	00	57	8	8	4	96		1								4281	42730	42369	42369	42369	41643
c	1	60	9	5	6	4	.9	4	1	0	51	33	15	15	15	13	823	74	42	42	42	53
					7	1																
				2	1	4																
GCA_0169896		15	41	7	0	4	84															
35.1_ASM1698		65	53	.	7	9	74															
963v1_genomi	2	68	16	8	8	0	40										4154	41497	41230	41177	40945	40603
c	8	4	9	1	1	8	.5	2	7	0	33	23	13	12	11	10	801	27	43	59	62	13
					2	1																
				2	6	1																
GCA_0169897			43	7	8	9	31															
15.1_ASM1698		62	25	.	2	4	29															
971v1_genomi	5	86	21	8	3	1	36		1								4328	43167	42754	42754	41847	41559
c	7	51	5	9	3	0	.6	6	5	0	66	44	23	23	19	18	178	19	77	77	53	40
					4	1																
				2	4	1																
GCA_0169897			42	7	1	8	46															
55.1_ASM1698		77	76	.	4	0	22															
975v1_genomi	3	38	57	8	5	9	61		1								4280	42722	42437	42437	42437	42149
c	8	41	8	9	2	1	.8	4	2	0	50	31	16	16	16	15	074	52	48	48	48	64

GCA_016989775.1_ASM1698977v1_genomic	24	938145	3970129	27.98	416669	280980	500903.7	4	8	0	32	22	13	13	12	11	3972687	3968788	3946832	3946832	3929137	3894595
GCA_016989785.1_ASM1698978v1_genomic	204	624810	4694759	27.92	263430	8254	244937.8	7	45	0	218	192	72	38	27	17	4699392	4686142	4394274	4165368	3990322	3634372
GCA_016989825.1_ASM1698982v1_genomic	25	2275100	4350026	28.25	2275100	146439	1376405.7	1	6	0	34	21	10	10	10	9	4352993	4347714	4322818	4322818	4322818	4294004
GCA_016989855.1_ASM1698985v1_genomic	53	586750	4366812	27.91	215685	82548	246226.3	7	19	0	62	46	29	29	27	20	4369978	4362632	4323233	4323233	4275772	4006756
GCA_0169898	3	16	40	2	7	1	95	2	6	0	43	25	10	10	10	9	4098	40905	40591	40591	40591	40303

95.1_ASM1698	1	60	94	8	4	4	06										296	47	43	43	43	29
989v1_genomi		31	80	.	3	0	07															
c		4	9	0	4	7	.6															
				3	5	7																
					0	3																
					5	1																
				2	8	3																
GCA_0169899			42	7	9	3	53															
75.1_ASM1698		88	57	.	2	2	09															
997v1_genomi	4	24	73	8	0	5	75										4262	42512	42128	42128	42128	41177
c	5	57	2	9	9	9	.6	3	9	0	61	35	15	15	15	12	384	32	03	03	03	64
					8	3																
				2	1	1																
GCA_0169900		16	40	8	5	1	97															
15.1_ASM1699		10	13	.	9	1	41															
001v1_genomi	1	76	48	0	5	2	21										4016	40128	39964	39964	39964	39676
c	7	2	5	7	7	2	.7	2	5	0	27	16	8	8	8	7	700	45	26	26	26	12
					7	1																
					3	4	10															
GCA_0169900		19	42	2	8	6	81															
25.1_ASM1699		12	03	7	1	4	23															
002v1_genomi	3	78	88	.	3	2	1.										4207	41998	41732	41732	41732	41444
c	0	9	0	9	8	4	7	2	7	0	43	24	11	11	11	10	851	51	99	99	99	85
GCA_0169900		70	39	2	2	6	29															
55.1_ASM1699	4	58	44	7	3	6	71		1								3946	39425	39173	39114	38476	36970
005v1_genomi	0	06	68	.	5	7	18	6	6	0	46	37	26	25	22	18	920	18	37	86	02	68

c			6	9	2	0	.3															
				9	4	6																
					6																	
					5	1																
				2	2	3																
GCA_0169900			41	8	1	3	42															
65.1_ASM1699		59	01	.	8	2	39															
006v1_genomi	4	80	81	0	9	2	56		1								4105	40953	40583	40412	40156	39868
c	9	38	1	1	3	9	.7	4	0	0	62	39	19	16	14	13	532	90	73	78	83	99
					1	1																
				2	9	2																
GCA_0169901			41	8	5	8	32															
15.1_ASM1699		82	62	.	2	4	06															
011v1_genomi	4	41	81	0	3	0	12		1								4167	41588	41264	41264	41082	39137
c	6	82	7	4	2	5	.2	6	6	0	61	40	25	25	24	18	208	15	53	53	72	26
					3																	
				2	6	8																
GCA_0169901			39	7	2	7																
55.1_ASM1699		74	01	.	6	7	35															
015v1_genomi	3	34	96	9	8	7	62		1								3904	39012	38794	38794	38155	37366
c	2	18	5	5	1	3	78	4	4	0	38	31	21	21	18	16	337	41	13	13	59	37
				2	4	1																
GCA_0169901			42	7	1	3	47															
95.1_ASM1699		91	65	.	0	1	57															
019v1_genomi	4	36	83	9	0	3	29		1								4270	42590	42188	42188	42032	41416
c	7	87	9	3	2	3	.2	4	1	0	61	37	17	17	16	14	285	27	36	36	77	17

GCA_016990235.1_ASM1699023v1_genomic	42	912296	4268949	27.88	0723245	7119412	570708	3	11	0	49	35	17	17	16	14	4271365	4263500	4226833	4226833	4211374	4144528
GCA_016990255.1_ASM1699025v1_genomic	45	603193	4058207	28.05	460477	117791	370783.9	4	12	0	58	39	20	19	17	15	4062838	4053703	4014081	4007442	3981795	3914896
GCA_016990275.1_ASM1699027v1_genomic	40	773437	4270340	27.88	274519	96485	415037	4	14	0	48	36	22	21	20	17	4273021	4267862	4236068	4229393	4213934	4103818
GCA_016990285.1_ASM1699028v1_genomic	21	2058083	3831850	28.04	2058083	141852	1291157.1	1	15	0	32	18	9	9	9	8	3835422	3829733	3805493	3805493	3805493	3776539

GCA_016990315.1_ASM1699031v1_genomic	29	1620237	4035226	28.03	8162533	3001098	958083.2	2	5	0	40	25	9	9	9	8	4038662	4032326	3999699	3999699	3999699	3970885
GCA_016990335.1_ASM1699033v1_genomic	48	895833	4107042	28.02	3921196	1359000	469285	4	10	0	60	38	18	16	14	13	4110538	4100801	4063198	4052600	4027005	3998221
GCA_016990345.1_ASM1699034v1_genomic	21	1880053	3831482	28.04	7923433	1356112	1147715.8	2	6	0	31	20	10	10	10	9	3834757	3830842	3804697	3804697	3804697	3775742
GCA_016990375.1_ASM1699037v1_genomic	39	937138	4109132	28.02	5977510	1118080	546218.8	3	10	0	52	32	14	14	14	13	4112819	4104758	4070339	4070339	4070339	4041555
GCA_016990395.1_ASM1699	20	8100	3847	28	67	29	5837	3	6	0	27	19	11	10	10	9	3850232	3847185	3828021	3821282	3821282	3777779

039v1_genomi		10	82		3	3	34															
c			5		3	5	.9															
					2	9																
					5	1																
					5	1																
				2	8	3																
GCA_0169904			41	8	9	5	56															
15.1_ASM1699		93	50	.	1	9	88															
041v1_genomi	3	78	94	0	7	2	92										4154	41465	41191	41191	40730	39683
c	5	14	5	4	2	9	.3	3	8	0	48	29	15	15	13	10	648	11	49	49	75	47
					1																	
				2	4	4																
GCA_0173306			43	7	2	2	18															
65.1_ASM1733		43	32	.	0	3	24															
066v1_genomi	6	97	55	7	0	5	58		3								4332	43284	43044	42859	41211	36657
c	7	77	5	3	3	2	.6	9	0	0	67	61	49	46	36	24	555	38	48	73	60	36
				2	5	1																
GCA_0173306			38	7	3	9	71															
85.1_ASM1733	1	25	43	.	5	0	29															
068v1_genomi	2	36	06	9	8	1	5.	2	6								3843	38395	37794	37374	31388	20157
c	2	43	6	6	5	4	5	2	9	0	122	116	95	89	54	23	066	51	74	49	08	18
				2	2	4																
GCA_0173306			39	8	7	4	30															
95.1_ASM1733		68	78	.	7	8	01															
069v1_genomi	5	60	90	0	2	2	92		1								3978	39704	39532	39429	37632	35698
c	5	68	5	1	6	0	.9	5	8	0	55	42	35	33	23	17	905	26	85	94	94	97

GCA_017330715.1_ASM1733071v1_genomic	40	636062	4185706	27.65	4255806	81315	318545.8	5	17	0	40	35	28	28	23	20	4185706	4182002	4165944	4165944	4075314	3982070
GCA_017330725.1_ASM1733072v1_genomic	131	215257	4187706	27.71	65567	17526	78229.6	19	68	0	131	125	101	85	52	25	4187706	4183321	4122890	4009968	3442280	2439678
GCA_017330765.1_ASM1733076v1_genomic	256	148760	4162320	27.69	32556	8152	44739.4	35	131	0	256	237	163	117	46	17	4162320	4149458	3955569	3624305	2431111	1366954
GCA_017330785.1_ASM1733078v1_genomic	80	273668	4199797	27.71	114010	37059	129694.7	12	38	0	80	69	57	55	43	29	4199797	4192253	4164911	4149538	3938043	3383591
GCA_017330795.1_ASM1733079v1_genomi	86	587411	433152	27.	225	491	242305	7	23	0	86	66	43	33	28	22	4331526	4318456	4260016	4194943	4112810	3889361

c			6	7	9	2	.3															
				5	0	5																
					1																	
				2	2																	
GCA_0173308			41	7	6	8	33															
15.1_ASM1733	2	10	63	.	6	2	29		1													
081v1_genomi	5	49	37	6	8	2	4.	4	4								4163	41591	39948	36431	23431	95280
c	2	53	7	6	2	9	8	6	9	0	252	247	186	137	56	15	377	63	23	70	84	1
				2	9	2																
GCA_0173308			42	7	0	2																
45.1_ASM1733		21	03	.	5	0	99															
084v1_genomi	9	35	61	6	1	7	22	1	5								4203	41992	41669	41385	37521	29260
c	7	89	4	7	8	4	3	5	2	0	97	90	78	74	50	27	614	70	21	58	93	80
					1																	
				2	1	2																
GCA_0173308			41	7	1	7	14															
65.1_ASM1733		32	98	.	9	7	43															
086v1_genomi	8	21	11	7	4	3	01	1	3								4198	41899	41562	41143	38305	33151
c	7	99	7	1	9	7	.6	1	8	0	87	75	62	56	39	24	117	11	24	28	06	37
				2	4	1																
GCA_0173308			41	7	6	2	55															
85.1_ASM1733	1	17	41	.	1	1	81															
088v1_genomi	9	73	30	7	2	1	2.	2	9								4141	41315	40035	38351	29881	20061
c	3	84	6	5	7	0	5	8	8	0	193	180	131	107	54	26	306	64	33	66	72	18
GCA_0173309	5	54	42	2	1	5	23		2								4217	42153	41853	41416	40888	38198
05.1_ASM1733	9	08	17	7	9	5	36	7	4	0	59	55	41	35	32	24	736	49	21	47	81	64

090v1_genomi		02	73	.	4	2	90															
c			6	7	2	9	.9															
					1	4																
					4																	
				2	7	1																
GCA_0173309			41	7	5	8	12															
25.1_ASM1733	1	41	58	.	3	1	92															
092v1_genomi	1	68	06	6	3	3	92	1	5								4158	41517	40879	39599	35870	31089
c	6	04	7	5	5	1	.1	4	0	0	116	107	82	65	42	29	067	33	21	37	62	22
					3																	
					0	8																
GCA_0173309			41	2	1	1	28															
45.1_ASM1733		50	58	7	4	3	07															
094v1_genomi	4	15	61	.	6	1	05		1								4158	41519	41375	41375	41006	40351
c	1	09	9	7	6	4	.2	6	6	0	41	31	24	24	22	20	619	27	42	42	50	96
					1																	
				2	2	3																
GCA_0173309			41	7	8	3	12															
65.1_ASM1733		27	39	.	5	6	96															
096v1_genomi	7	89	90	6	8	5	17	1	3								4139	41353	40992	40552	38952	33661
c	9	89	9	8	7	5	.1	2	6	0	79	71	56	50	41	27	909	69	76	67	12	17
				2	2	9																
GCA_0173309			41	7	5	5	33															
85.1_ASM1733		65	55	.	5	6	52															
098v1_genomi	3	32	71	6	8	5	97		1								4155	41523	41405	41345	40981	40003
c	4	90	0	8	4	3	.3	5	4	0	34	29	23	22	20	17	710	33	32	25	81	83

GCA_017331005.1_ASM1733100v1_genomic	85	594976	4227167	27.71	2118018	34152	220956.1	8	31	0	85	68	49	43	37	24	4227167	4215090	4169491	4126735	4008762	3574585
GCA_017331025.1_ASM1733102v1_genomic	78	430766	4153245	27.7	133216	32553	168251.2	10	32	0	78	71	57	49	34	24	4153245	4148449	4116021	4062196	3818712	3463264
GCA_017331045.1_ASM1733104v1_genomic	64	594898	4150368	27.69	224881	46863	258952.9	6	23	0	64	54	43	35	28	22	4150368	4143871	4119326	4066966	3953783	3734244
GCA_017590175.1_ASM1759017v1_genomic	237	148029	3789533	28.06	46820	11494	51808.9	27	89	0	238	161	118	95	57	25	3790015	3741158	3633993	3478916	2880930	1827886
GCA_017591115.1_ASM1759	27	1283	4057	28	28	81	3572	42	14	0	274	232	179	128	52	17	4057568	4030990	3889817	3524099	2304329	1115533

111v1_genomi	4	21	56	.	7	0	5.		3													
c			8	0	1	7	4															
				5	5																	
				2	4	1																
GCA_0177732			41	8	8	2	69															
55.1_PDT0009	1	21	29	.	0	9	03															
85504.1_geno	6	86	97	1	3	0	3.	2	8								4131	41253	40083	38508	30749	19732
mic	7	45	0	3	1	0	3	4	8	0	171	161	121	99	53	22	227	56	17	28	23	39
					2																	
					7	2																
				2	5	9	20															
GCA_9023862		27	38	8	2	9	74															
35.1_UHGG_M		52	08	.	8	3	85															
GYG-HGUT-0	1	80	26	0	0	9	8.										3808	38082	38008	37917	37917	37639
2355_genomic	1	9	2	1	9	4	9	1	3	0.03	11	11	7	6	6	5	262	62	68	17	17	94

Table S2 Basic information of annotation completeness based on BUSCO

Strains	Complete	Complete and single copy	Complete and duplicated	Fragmented	Missing	Total BUSCO groups
XJFE01	C:99.7%	S:98.9%	D:0.8%	F:0.0%	M:0.3%	n:264
XJFE02	C:99.7%	S:98.9%	D:0.8%	F:0.0%	M:0.3%	n:264
XJFE03	C:99.7%	S:98.9%	D:0.8%	F:0.0%	M:0.3%	n:264
XJFE04	C:99.7%	S:98.9%	D:0.8%	F:0.0%	M:0.3%	n:264
XJFE05	C:99.7%	S:98.9%	D:0.8%	F:0.0%	M:0.3%	n:264
XJFE06	C:99.7%	S:98.9%	D:0.8%	F:0.0%	M:0.3%	n:264
XJFD01	C:99.7%	S:98.9%	D:0.8%	F:0.0%	M:0.3%	n:264
XJFD02	C:99.7%	S:98.9%	D:0.8%	F:0.0%	M:0.3%	n:264
XJFD03	C:99.7%	S:98.9%	D:0.8%	F:0.0%	M:0.3%	n:264
XJFD04	C:99.7%	S:98.9%	D:0.8%	F:0.0%	M:0.3%	n:264
XJFD05	C:99.7%	S:98.9%	D:0.8%	F:0.0%	M:0.3%	n:264
XJFD06	C:99.7%	S:98.9%	D:0.8%	F:0.0%	M:0.3%	n:264
XJFD07	C:99.7%	S:98.9%	D:0.8%	F:0.0%	M:0.3%	n:264
XJFD08	C:99.7%	S:98.9%	D:0.8%	F:0.0%	M:0.3%	n:264
XJFD09	C:99.7%	S:98.9%	D:0.8%	F:0.0%	M:0.3%	n:264
XJFD10	C:99.7%	S:98.9%	D:0.8%	F:0.0%	M:0.3%	n:264
XJFD11	C:99.7%	S:98.9%	D:0.8%	F:0.0%	M:0.3%	n:264
XJFD12	C:99.7%	S:98.9%	D:0.8%	F:0.0%	M:0.3%	n:264
XJFD13	C:99.7%	S:98.9%	D:0.8%	F:0.0%	M:0.3%	n:264
XJFD14	C:99.7%	S:98.9%	D:0.8%	F:0.0%	M:0.3%	n:264
XJFD15	C:99.7%	S:98.9%	D:0.8%	F:0.0%	M:0.3%	n:264
XJFD16	C:99.7%	S:98.9%	D:0.8%	F:0.0%	M:0.3%	n:264
XJFD17	C:99.7%	S:98.9%	D:0.8%	F:0.0%	M:0.3%	n:264

XJFD18	C:99.6%	S:98.1%	D:1.5%	F:0.0%	M:0.4%	n:264
XJFD19	C:99.7%	S:98.9%	D:0.8%	F:0.0%	M:0.3%	n:264
XJFD20	C:99.7%	S:98.9%	D:0.8%	F:0.0%	M:0.3%	n:264
XJFD21	C:99.7%	S:98.9%	D:0.8%	F:0.0%	M:0.3%	n:264
XJFD22	C:99.7%	S:98.9%	D:0.8%	F:0.0%	M:0.3%	n:264
XJFD23	C:99.7%	S:98.9%	D:0.8%	F:0.0%	M:0.3%	n:264
XJFD24	C:99.7%	S:98.9%	D:0.8%	F:0.0%	M:0.3%	n:264
XJFD25	C:99.7%	S:98.9%	D:0.8%	F:0.0%	M:0.3%	n:264
XJFD26	C:99.7%	S:98.9%	D:0.8%	F:0.0%	M:0.3%	n:264
XJFD27	C:99.7%	S:98.9%	D:0.8%	F:0.0%	M:0.3%	n:264
XJSL01	C:99.7%	S:98.9%	D:0.8%	F:0.0%	M:0.3%	n:264
XJSL02	C:99.7%	S:98.9%	D:0.8%	F:0.0%	M:0.3%	n:264
XJSL03	C:99.7%	S:98.9%	D:0.8%	F:0.0%	M:0.3%	n:264
XJSL04	C:99.7%	S:98.9%	D:0.8%	F:0.0%	M:0.3%	n:264
XJSL05	C:99.7%	S:98.9%	D:0.8%	F:0.0%	M:0.3%	n:264
XJSL06	C:99.7%	S:98.9%	D:0.8%	F:0.0%	M:0.3%	n:264
XJSL07	C:99.7%	S:98.9%	D:0.8%	F:0.0%	M:0.3%	n:264
XJSL08	C:99.7%	S:98.9%	D:0.8%	F:0.0%	M:0.3%	n:264
XJSL09	C:99.7%	S:98.9%	D:0.8%	F:0.0%	M:0.3%	n:264
XJSL10	C:99.7%	S:98.9%	D:0.8%	F:0.0%	M:0.3%	n:264
XJSL11	C:99.7%	S:98.9%	D:0.8%	F:0.0%	M:0.3%	n:264
XJSL12	C:99.7%	S:98.9%	D:0.8%	F:0.0%	M:0.3%	n:264
XJSL13	C:99.7%	S:98.9%	D:0.8%	F:0.0%	M:0.3%	n:264
XJSL14	C:99.7%	S:98.9%	D:0.8%	F:0.0%	M:0.3%	n:264
XJSL15	C:99.7%	S:98.9%	D:0.8%	F:0.0%	M:0.3%	n:264
XJSL16	C:99.7%	S:98.9%	D:0.8%	F:0.0%	M:0.3%	n:264

XJSL17	C:99.7%	S:98.9%	D:0.8%	F:0.0%	M:0.3%	n:264
XJSL18	C:99.7%	S:98.9%	D:0.8%	F:0.0%	M:0.3%	n:264
XJSL19	C:99.7%	S:98.9%	D:0.8%	F:0.0%	M:0.3%	n:264
XJSL20	C:99.7%	S:98.9%	D:0.8%	F:0.0%	M:0.3%	n:264
XJSL21	C:99.7%	S:98.9%	D:0.8%	F:0.0%	M:0.3%	n:264
XJSL22	C:99.7%	S:98.9%	D:0.8%	F:0.0%	M:0.3%	n:264
XJSL23	C:99.7%	S:98.9%	D:0.8%	F:0.0%	M:0.3%	n:264
XJSL24	C:99.7%	S:98.9%	D:0.8%	F:0.0%	M:0.3%	n:264
XJSL25	C:99.7%	S:98.9%	D:0.8%	F:0.0%	M:0.3%	n:264
XJSL26	C:99.6%	S:98.5%	D:1.1%	F:0.0%	M:0.4%	n:264
GCA_000017045.1_ASM1704v1_genomic	C:99.6%	S:98.5%	D:1.1%	F:0.0%	M:0.4%	n:264
GCA_000017065.1_ASM1706v1_genomic	C:99.6%	S:98.5%	D:1.1%	F:0.0%	M:0.4%	n:264
GCA_000019305.1_ASM1930v1_genomic	C:99.6%	S:98.5%	D:1.1%	F:0.0%	M:0.4%	n:264
GCA_000019545.1_ASM1954v1_genomic	C:99.6%	S:98.5%	D:1.1%	F:0.0%	M:0.4%	n:264
GCA_000020345.1_ASM2034v1_genomic	C:99.6%	S:98.5%	D:1.1%	F:0.0%	M:0.4%	n:264
GCA_000022765.1_ASM2276v1_genomic	C:99.6%	S:98.5%	D:1.1%	F:0.0%	M:0.4%	n:264
GCA_000063585.1_ASM6358v1_genomic	C:99.6%	S:98.5%	D:1.1%	F:0.0%	M:0.4%	n:264
GCA_000092345.1_ASM9234v1_genomic	C:94.7%	S:93.9%	D:0.8%	F:3.8%	M:1.5%	n:264
GCA_000171055.1_ASM17105v1_genomic	C:99.2%	S:97.7%	D:1.5%	F:0.0%	M:0.8%	n:264
GCA_000171075.1_ASM17107v1_genomic	C:98.1%	S:97.0%	D:1.1%	F:0.8%	M:1.1%	n:264
GCA_000253195.1_ASM25319v1_genomic	C:98.5%	S:97.7%	D:0.8%	F:0.8%	M:0.7%	n:264
GCA_000307635.1_ASM30763v1_genomic	C:80.3%	S:80.3%	D:0.0%	F:15.2%	M:4.5%	n:264
GCA_000307655.2_CFSAN0001628_2.0_genomic	C:89.1%	S:88.3%	D:0.8%	F:6.1%	M:4.8%	n:264
GCA_000439615.1_CFSAN002367_1.0_genomic	C:54.2%	S:54.2%	D:0.0%	F:35.2%	M:10.6 %	n:264
GCA_000439635.1_CFSAN002368_1.0_genomic	C:53.8%	S:53.8%	D:0.0%	F:33.7%	M:12.5	n:264

					%	
GCA_000439655.1_CFSAN002369_1.0_genomic	C:49.6%	S:49.6%	D:0.0%	F:36.0%	M:14.4 %	n:264
GCA_000439815.2_ASM43981v2_genomic	C:93.5%	S:92.4%	D:1.1%	F:0.8%	M:5.7%	n:264
GCA_000503815.1_ASM50381v1_genomic	C:98.8%	S:97.3%	D:1.5%	F:0.4%	M:0.8%	n:264
GCA_000582375.1_CDC54075.1_genomic	C:89.4%	S:88.6%	D:0.8%	F:9.1%	M:1.5%	n:264
GCA_000582415.1_CDC54088.1_genomic	C:92.4%	S:92.4%	D:0.0%	F:5.7%	M:1.9%	n:264
GCA_000582435.1_CDC54085.1_genomic	C:94.7%	S:93.9%	D:0.8%	F:4.5%	M:0.8%	n:264
GCA_000582455.1_CDC54091.1_genomic	C:56.5%	S:56.1%	D:0.4%	F:27.3%	M:16.2 %	n:264
GCA_000710975.1_B2_331_genomic	C:99.6%	S:98.5%	D:1.1%	F:0.0%	M:0.4%	n:264
GCA_000710985.1_B2_275_genomic	C:99.6%	S:98.5%	D:1.1%	F:0.0%	M:0.4%	n:264
GCA_000710995.1_A2B3_87_genomic	C:99.7%	S:98.9%	D:0.8%	F:0.0%	M:0.3%	n:264
GCA_000711005.1_A2B7_92_genomic	C:99.2%	S:98.1%	D:1.1%	F:0.4%	M:0.4%	n:264
GCA_000711055.1_A2_117_genomic	C:99.6%	S:98.5%	D:1.1%	F:0.0%	M:0.4%	n:264
GCA_000711065.1_B2_128_genomic	C:99.6%	S:98.5%	D:1.1%	F:0.0%	M:0.4%	n:264
GCA_000711095.1_B2_267_genomic	C:99.6%	S:98.5%	D:1.1%	F:0.0%	M:0.4%	n:264
GCA_000711105.1_F_357_genomic	C:98.8%	S:97.7%	D:1.1%	F:0.8%	M:0.4%	n:264
GCA_000711115.1_B2_433_genomic	C:99.6%	S:98.5%	D:1.1%	F:0.0%	M:0.4%	n:264
GCA_000730705.1_ASM73070v1_genomic	C:94.7%	S:93.9%	D:0.8%	F:4.5%	M:0.8%	n:264
GCA_000730715.1_ASM73071v1_genomic	C:94.4%	S:93.6%	D:0.8%	F:3.8%	M:1.8%	n:264
GCA_000730725.1_ASM73072v1_genomic	C:95.5%	S:94.7%	D:0.8%	F:3.4%	M:1.1%	n:264
GCA_000730735.1_ASM73073v1_genomic	C:91.3%	S:90.2%	D:1.1%	F:5.7%	M:3.0%	n:264
GCA_000730785.1_ASM73078v1_genomic	C:96.6%	S:95.8%	D:0.8%	F:2.3%	M:1.1%	n:264
GCA_000730795.1_ASM73079v1_genomic	C:95.5%	S:94.7%	D:0.8%	F:3.8%	M:0.7%	n:264
GCA_000730805.1_ASM73080v1_genomic	C:93.2%	S:92.4%	D:0.8%	F:5.7%	M:1.1%	n:264

GCA_000730835.1_ASM73083v1_genomic	C:89.8%	S:88.3%	D:1.5%	F:6.8%	M:3.4%	n:264
GCA_000730865.1_ASM73086v1_genomic	C:89.1%	S:88.3%	D:0.8%	F:6.8%	M:4.1%	n:264
GCA_000730875.1_ASM73087v1_genomic	C:93.2%	S:92.4%	D:0.8%	F:5.7%	M:1.1%	n:264
GCA_000730885.1_ASM73088v1_genomic	C:93.2%	S:92.4%	D:0.8%	F:4.5%	M:2.3%	n:264
GCA_000730925.1_ASM73092v1_genomic	C:94.0%	S:93.2%	D:0.8%	F:4.2%	M:1.8%	n:264
GCA_000730945.1_ASM73094v1_genomic	C:80.7%	S:79.9%	D:0.8%	F:13.6%	M:5.7%	n:264
GCA_000730955.1_ASM73095v1_genomic	C:88.6%	S:87.5%	D:1.1%	F:7.6%	M:3.8%	n:264
GCA_000730965.1_ASM73096v1_genomic	C:95.9%	S:95.5%	D:0.4%	F:3.0%	M:1.1%	n:264
GCA_000769495.1_CFSAN024410_01.0_genomic	C:99.6%	S:99.2%	D:0.4%	F:0.0%	M:0.4%	n:264
GCA_000816945.1_ASM81694v1_genomic	C:99.7%	S:98.9%	D:0.8%	F:0.0%	M:0.3%	n:264
GCA_000816965.1_ASM81696v1_genomic	C:99.6%	S:98.1%	D:1.5%	F:0.0%	M:0.4%	n:264
GCA_000817935.1_ASM81793v1_genomic	C:99.6%	S:98.5%	D:1.1%	F:0.0%	M:0.4%	n:264
GCA_000829015.1_ASM82901v1_genomic	C:99.6%	S:98.5%	D:1.1%	F:0.0%	M:0.4%	n:264
GCA_000830755.1_ASM83075v1_genomic	C:99.6%	S:99.2%	D:0.4%	F:0.0%	M:0.4%	n:264
GCA_000876495.2_ASM87649v2_genomic	C:99.6%	S:98.5%	D:1.1%	F:0.0%	M:0.4%	n:264
GCA_000965295.1_ASM96529v1_genomic	C:66.3%	S:65.5%	D:0.8%	F:26.9%	M:6.8%	n:264
GCA_000965325.1_ASM96532v1_genomic	C:37.1%	S:36.7%	D:0.4%	F:44.7%	M:18.2%	n:264
GCA_000965345.1_ASM96534v1_genomic	C:41.3%	S:40.9%	D:0.4%	F:36.7%	M:22.0%	n:264
GCA_000965375.1_ASM96537v1_genomic	C:62.5%	S:62.1%	D:0.4%	F:26.1%	M:11.4%	n:264
GCA_000965385.1_ASM96538v1_genomic	C:58.7%	S:58.7%	D:0.0%	F:27.7%	M:13.6%	n:264
GCA_000965445.1_ASM96544v1_genomic	C:61.8%	S:61.0%	D:0.8%	F:25.4%	M:12.8%	n:264

GCA_000965465.1_ASM96546v1_genomic	C:42.0%	S:42.0%	D:0.0%	F:35.2%	M:22.8%	n:264
GCA_000965505.1_ASM96550v1_genomic	C:59.8%	S:59.8%	D:0.0%	F:27.7%	M:12.5%	n:264
GCA_000965515.1_ASM96551v1_genomic	C:64.8%	S:64.0%	D:0.8%	F:24.6%	M:10.6%	n:264
GCA_000986925.1_CBOT001-SEQ-1-ASM-1_genomic	C:99.6%	S:98.5%	D:1.1%	F:0.0%	M:0.4%	n:264
GCA_001273165.1_CBOT002-SEQ-1-ASM-1_genomic	C:99.2%	S:98.1%	D:1.1%	F:0.4%	M:0.4%	n:264
GCA_001273255.1_CBOT006-SEQ-1-ASM-1_genomic	C:99.6%	S:98.5%	D:1.1%	F:0.0%	M:0.4%	n:264
GCA_001273275.1_CBOT007-SEQ-1-ASM-1_genomic	C:99.6%	S:98.5%	D:1.1%	F:0.0%	M:0.4%	n:264
GCA_001276985.1_CBOT009-SEQ-1-ASM-1_genomic	C:99.6%	S:98.5%	D:1.1%	F:0.0%	M:0.4%	n:264
GCA_001573175.1_10148_genomic	C:99.6%	S:98.5%	D:1.1%	F:0.0%	M:0.4%	n:264
GCA_001573235.1_ASM157323v1_genomic	C:99.3%	S:98.5%	D:0.8%	F:0.4%	M:0.3%	n:264
GCA_001573255.1_ASM157325v1_genomic	C:99.6%	S:98.5%	D:1.1%	F:0.0%	M:0.4%	n:264
GCA_001573295.1_ASM157329v1_genomic	C:99.6%	S:98.5%	D:1.1%	F:0.0%	M:0.4%	n:264
GCA_001573315.1_ASM157331v1_genomic	C:99.6%	S:98.1%	D:1.5%	F:0.0%	M:0.4%	n:264
GCA_001573325.1_ASM157332v1_genomic	C:99.6%	S:96.2%	D:3.4%	F:0.0%	M:0.4%	n:264
GCA_001573335.1_ASM157333v1_genomic	C:99.6%	S:98.5%	D:1.1%	F:0.0%	M:0.4%	n:264
GCA_001573375.1_ASM157337v1_genomic	C:99.6%	S:98.5%	D:1.1%	F:0.0%	M:0.4%	n:264
GCA_001573385.1_ASM157338v1_genomic	C:99.6%	S:98.5%	D:1.1%	F:0.0%	M:0.4%	n:264
GCA_001573395.1_ASM157339v1_genomic	C:99.6%	S:98.5%	D:1.1%	F:0.0%	M:0.4%	n:264
GCA_001573435.1_ASM157343v1_genomic	C:99.6%	S:98.5%	D:1.1%	F:0.0%	M:0.4%	n:264
GCA_001573455.1_ASM157345v1_genomic	C:99.6%	S:98.5%	D:1.1%	F:0.0%	M:0.4%	n:264
GCA_001573465.1_ASM157346v1_genomic	C:99.6%	S:98.5%	D:1.1%	F:0.0%	M:0.4%	n:264
GCA_001573485.1_ASM157348v1_genomic	C:99.6%	S:98.5%	D:1.1%	F:0.0%	M:0.4%	n:264
GCA_001573515.1_ASM157351v1_genomic	C:99.6%	S:98.5%	D:1.1%	F:0.0%	M:0.4%	n:264

GCA_001573535.1_ASM157353v1_genomic	C:99.7%	S:98.9%	D:0.8%	F:0.0%	M:0.3%	n:264
GCA_001573555.1_ASM157355v1_genomic	C:99.3%	S:98.5%	D:0.8%	F:0.4%	M:0.3%	n:264
GCA_001573665.1_ASM157366v1_genomic	C:99.6%	S:96.2%	D:3.4%	F:0.0%	M:0.4%	n:264
GCA_001573725.1_ASM157372v1_genomic	C:99.6%	S:98.5%	D:1.1%	F:0.0%	M:0.4%	n:264
GCA_001573825.1_ASM157382v1_genomic	C:99.6%	S:98.5%	D:1.1%	F:0.0%	M:0.4%	n:264
GCA_001573885.1_ASM157388v1_genomic	C:99.6%	S:98.5%	D:1.1%	F:0.0%	M:0.4%	n:264
GCA_001573905.1_ASM157390v1_genomic	C:99.6%	S:98.5%	D:1.1%	F:0.0%	M:0.4%	n:264
GCA_001573935.1_ASM157393v1_genomic	C:99.6%	S:98.5%	D:1.1%	F:0.0%	M:0.4%	n:264
GCA_001573955.1_ASM157395v1_genomic	C:99.6%	S:98.5%	D:1.1%	F:0.0%	M:0.4%	n:264
GCA_001573965.1_ASM157396v1_genomic	C:99.6%	S:98.5%	D:1.1%	F:0.0%	M:0.4%	n:264
GCA_001573985.1_ASM157398v1_genomic	C:99.6%	S:98.5%	D:1.1%	F:0.0%	M:0.4%	n:264
GCA_001574015.1_ASM157401v1_genomic	C:99.6%	S:98.5%	D:1.1%	F:0.0%	M:0.4%	n:264
GCA_001574035.1_ASM157403v1_genomic	C:99.6%	S:98.5%	D:1.1%	F:0.0%	M:0.4%	n:264
GCA_001574055.1_ASM157405v1_genomic	C:99.6%	S:98.5%	D:1.1%	F:0.0%	M:0.4%	n:264
GCA_001574065.1_ASM157406v1_genomic	C:99.6%	S:98.5%	D:1.1%	F:0.0%	M:0.4%	n:264
GCA_001574095.1_ASM157409v1_genomic	C:99.6%	S:98.5%	D:1.1%	F:0.0%	M:0.4%	n:264
GCA_001574115.1_ASM157411v1_genomic	C:99.6%	S:98.5%	D:1.1%	F:0.0%	M:0.4%	n:264
GCA_001574125.1_ASM157412v1_genomic	C:99.6%	S:98.5%	D:1.1%	F:0.0%	M:0.4%	n:264
GCA_001574135.1_ASM157413v1_genomic	C:99.6%	S:98.5%	D:1.1%	F:0.0%	M:0.4%	n:264
GCA_001574175.1_ASM157417v1_genomic	C:99.6%	S:98.5%	D:1.1%	F:0.0%	M:0.4%	n:264
GCA_001574195.1_ASM157419v1_genomic	C:99.6%	S:98.5%	D:1.1%	F:0.0%	M:0.4%	n:264
GCA_001574205.1_ASM157420v1_genomic	C:99.6%	S:98.5%	D:1.1%	F:0.0%	M:0.4%	n:264
GCA_001574225.1_ASM157422v1_genomic	C:99.6%	S:98.1%	D:1.5%	F:0.0%	M:0.4%	n:264
GCA_001574255.1_ASM157425v1_genomic	C:99.6%	S:98.5%	D:1.1%	F:0.0%	M:0.4%	n:264
GCA_001574265.1_ASM157426v1_genomic	C:99.6%	S:98.5%	D:1.1%	F:0.0%	M:0.4%	n:264
GCA_001574285.1_ASM157428v1_genomic	C:99.6%	S:98.5%	D:1.1%	F:0.0%	M:0.4%	n:264

GCA_001574315.1_ASM157431v1_genomic	C:99.6%	S:98.5%	D:1.1%	F:0.0%	M:0.4%	n:264
GCA_001574335.1_ASM157433v1_genomic	C:99.6%	S:98.5%	D:1.1%	F:0.0%	M:0.4%	n:264
GCA_001574355.1_ASM157435v1_genomic	C:99.6%	S:98.5%	D:1.1%	F:0.0%	M:0.4%	n:264
GCA_001574365.1_ASM157436v1_genomic	C:99.6%	S:98.5%	D:1.1%	F:0.0%	M:0.4%	n:264
GCA_001879605.1_ASM187960v1_genomic	C:99.6%	S:98.5%	D:1.1%	F:0.0%	M:0.4%	n:264
GCA_001879625.1_ASM187962v1_genomic	C:99.6%	S:98.5%	D:1.1%	F:0.0%	M:0.4%	n:264
GCA_001889345.1_ASM188934v1_genomic	C:99.6%	S:98.5%	D:1.1%	F:0.0%	M:0.4%	n:264
GCA_001889365.1_ASM188936v1_genomic	C:99.6%	S:98.5%	D:1.1%	F:0.0%	M:0.4%	n:264
GCA_001921905.1_ASM192190v1_genomic	C:99.6%	S:98.5%	D:1.1%	F:0.0%	M:0.4%	n:264
GCA_001921925.1_ASM192192v1_genomic	C:99.6%	S:98.5%	D:1.1%	F:0.0%	M:0.4%	n:264
GCA_001921945.1_ASM192194v1_genomic	C:99.6%	S:98.5%	D:1.1%	F:0.0%	M:0.4%	n:264
GCA_001921965.1_ASM192196v1_genomic	C:99.6%	S:98.5%	D:1.1%	F:0.0%	M:0.4%	n:264
GCA_001921985.1_ASM192198v1_genomic	C:99.2%	S:98.1%	D:1.1%	F:0.4%	M:0.4%	n:264
GCA_001951135.2_ASM195113v2_genomic	C:98.8%	S:97.7%	D:1.1%	F:0.8%	M:0.4%	n:264
GCA_002024365.1_ASM202436v1_genomic	C:99.7%	S:98.9%	D:0.8%	F:0.0%	M:0.3%	n:264
GCA_002024375.1_ASM202437v1_genomic	C:99.7%	S:98.9%	D:0.8%	F:0.0%	M:0.3%	n:264
GCA_002024385.1_ASM202438v1_genomic	C:99.7%	S:98.9%	D:0.8%	F:0.0%	M:0.3%	n:264
GCA_002024395.1_ASM202439v1_genomic	C:99.7%	S:98.9%	D:0.8%	F:0.0%	M:0.3%	n:264
GCA_002024445.1_ASM202444v1_genomic	C:99.7%	S:98.9%	D:0.8%	F:0.0%	M:0.3%	n:264
GCA_002024465.1_ASM202446v1_genomic	C:98.5%	S:98.1%	D:0.4%	F:0.4%	M:1.1%	n:264
GCA_002103795.1_ASM210379v1_genomic	C:99.6%	S:98.5%	D:1.1%	F:0.0%	M:0.4%	n:264
GCA_002103805.1_ASM210380v1_genomic	C:99.6%	S:98.5%	D:1.1%	F:0.0%	M:0.4%	n:264
GCA_002103825.1_ASM210382v1_genomic	C:99.6%	S:98.5%	D:1.1%	F:0.0%	M:0.4%	n:264
GCA_002103855.1_ASM210385v1_genomic	C:99.7%	S:98.9%	D:0.8%	F:0.0%	M:0.3%	n:264
GCA_002103875.1_ASM210387v1_genomic	C:99.6%	S:98.5%	D:1.1%	F:0.0%	M:0.4%	n:264
GCA_002103885.1_ASM210388v1_genomic	C:99.6%	S:98.5%	D:1.1%	F:0.0%	M:0.4%	n:264

GCA_002103975.1_ASM210397v1_genomic	C:96.6%	S:95.5%	D:1.1%	F:1.9%	M:1.5%	n:264
GCA_002103985.1_ASM210398v1_genomic	C:99.6%	S:88.6%	D:11.0%	F:0.0%	M:0.4%	n:264
GCA_002104015.1_ASM210401v1_genomic	C:99.6%	S:98.5%	D:1.1%	F:0.0%	M:0.4%	n:264
GCA_002260585.1_C.botulinum_2.0_genomic	C:61.8%	S:61.4%	D:0.4%	F:12.5%	M:25.7 %	n:264
GCA_002865745.1_ASM286574v1_genomic	C:99.6%	S:98.5%	D:1.1%	F:0.0%	M:0.4%	n:264
GCA_002865765.1_ASM286576v1_genomic	C:99.6%	S:98.5%	D:1.1%	F:0.0%	M:0.4%	n:264
GCA_002865805.1_ASM286580v1_genomic	C:99.6%	S:98.5%	D:1.1%	F:0.0%	M:0.4%	n:264
GCA_002865825.1_ASM286582v1_genomic	C:99.6%	S:98.5%	D:1.1%	F:0.0%	M:0.4%	n:264
GCA_002865845.1_ASM286584v1_genomic	C:99.6%	S:98.5%	D:1.1%	F:0.0%	M:0.4%	n:264
GCA_002865885.1_ASM286588v1_genomic	C:99.6%	S:98.5%	D:1.1%	F:0.0%	M:0.4%	n:264
GCA_002866045.1_ASM286604v1_genomic	C:99.6%	S:98.5%	D:1.1%	F:0.0%	M:0.4%	n:264
GCA_002866125.1_ASM286612v1_genomic	C:99.6%	S:98.5%	D:1.1%	F:0.0%	M:0.4%	n:264
GCA_002866225.1_ASM286622v1_genomic	C:99.6%	S:98.5%	D:1.1%	F:0.0%	M:0.4%	n:264
GCA_003014955.1_ASM301495v1_genomic	C:97.8%	S:97.0%	D:0.8%	F:1.9%	M:0.3%	n:264
GCA_003017145.1_ASM301714v1_genomic	C:99.7%	S:98.9%	D:0.8%	F:0.0%	M:0.3%	n:264
GCA_003017195.1_ASM301719v1_genomic	C:99.7%	S:98.9%	D:0.8%	F:0.0%	M:0.3%	n:264
GCA_003017225.1_ASM301722v1_genomic	C:99.6%	S:98.5%	D:1.1%	F:0.0%	M:0.4%	n:264
GCA_003017335.1_ASM301733v1_genomic	C:95.0%	S:93.9%	D:1.1%	F:0.0%	M:5.0%	n:264
GCA_003058345.1_ASM305834v1_genomic	C:99.6%	S:98.5%	D:1.1%	F:0.0%	M:0.4%	n:264
GCA_003058445.1_ASM305844v1_genomic	C:98.8%	S:97.7%	D:1.1%	F:0.8%	M:0.4%	n:264
GCA_003345315.1_ASM334531v1_genomic	C:99.6%	S:98.5%	D:1.1%	F:0.0%	M:0.4%	n:264
GCA_003345335.1_ASM334533v1_genomic	C:99.7%	S:98.9%	D:0.8%	F:0.0%	M:0.3%	n:264
GCA_003412385.1_ASM341238v1_genomic	C:42.4%	S:42.4%	D:0.0%	F:36.0%	M:21.6 %	n:264
GCA_003412395.1_ASM341239v1_genomic	C:42.8%	S:42.8%	D:0.0%	F:37.5%	M:19.7	n:264

					%	
GCA_003412435.1_ASM341243v1_genomic	C:46.2%	S:45.8%	D:0.4%	F:37.9%	M:15.9 %	n:264
GCA_003515665.1_ASM351566v1_genomic	C:87.5%	S:86.7%	D:0.8%	F:10.2%	M:2.3%	n:264
GCA_003610755.1_ASM361075v1_genomic	C:99.7%	S:98.9%	D:0.8%	F:0.0%	M:0.3%	n:264
GCA_003994935.1_ASM399493v1_genomic	C:99.6%	S:98.5%	D:1.1%	F:0.0%	M:0.4%	n:264
GCA_003996845.1_ASM399684v1_genomic	C:66.3%	S:65.9%	D:0.4%	F:26.1%	M:7.6%	n:264
GCA_003996885.1_ASM399688v1_genomic	C:74.3%	S:73.9%	D:0.4%	F:20.5%	M:5.2%	n:264
GCA_003996895.1_ASM399689v1_genomic	C:40.6%	S:40.2%	D:0.4%	F:38.6%	M:20.8 %	n:264
GCA_003996925.1_ASM399692v1_genomic	C:15.5%	S:15.5%	D:0.0%	F:35.6%	M:48.9 %	n:264
GCA_003996935.1_ASM399693v1_genomic	C:72.0%	S:71.2%	D:0.8%	F:24.2%	M:3.8%	n:264
GCA_004120535.1_ASM412053v1_genomic	C:99.6%	S:98.5%	D:1.1%	F:0.0%	M:0.4%	n:264
GCA_009733885.1_ASM973388v1_genomic	C:99.6%	S:98.5%	D:1.1%	F:0.0%	M:0.4%	n:264
GCA_009938935.1_ASM993893v1_genomic	C:99.6%	S:98.5%	D:1.1%	F:0.0%	M:0.4%	n:264
GCA_009939325.1_ASM993932v1_genomic	C:99.7%	S:98.9%	D:0.8%	F:0.0%	M:0.3%	n:264
GCA_009939345.1_ASM993934v1_genomic	C:99.6%	S:98.5%	D:1.1%	F:0.0%	M:0.4%	n:264
GCA_010078315.1_ASM1007831v1_genomic	C:99.6%	S:98.5%	D:1.1%	F:0.0%	M:0.4%	n:264
GCA_011009485.1_ASM1100948v1_genomic	C:99.6%	S:98.5%	D:1.1%	F:0.0%	M:0.4%	n:264
GCA_011009795.1_ASM1100979v1_genomic	C:99.6%	S:98.5%	D:1.1%	F:0.0%	M:0.4%	n:264
GCA_011009805.1_ASM1100980v1_genomic	C:99.6%	S:98.5%	D:1.1%	F:0.0%	M:0.4%	n:264
GCA_011009855.1_ASM1100985v1_genomic	C:99.6%	S:98.5%	D:1.1%	F:0.0%	M:0.4%	n:264
GCA_011009875.1_ASM1100987v1_genomic	C:99.2%	S:97.7%	D:1.5%	F:0.4%	M:0.4%	n:264
GCA_011009885.1_ASM1100988v1_genomic	C:93.9%	S:92.8%	D:1.1%	F:0.8%	M:5.3%	n:264
GCA_011009915.1_ASM1100991v1_genomic	C:99.7%	S:98.9%	D:0.8%	F:0.0%	M:0.3%	n:264

GCA_011009925.1_ASM1100992v1_genomic	C:99.7%	S:98.9%	D:0.8%	F:0.0%	M:0.3%	n:264
GCA_011009955.1_ASM1100995v1_genomic	C:99.6%	S:98.5%	D:1.1%	F:0.0%	M:0.4%	n:264
GCA_011009975.1_ASM1100997v1_genomic	C:99.2%	S:98.1%	D:1.1%	F:0.4%	M:0.4%	n:264
GCA_011009985.1_ASM1100998v1_genomic	C:99.2%	S:98.1%	D:1.1%	F:0.4%	M:0.4%	n:264
GCA_011010005.1_ASM1101000v1_genomic	C:99.6%	S:98.5%	D:1.1%	F:0.4%	M:0.0%	n:264
GCA_011010035.1_ASM1101003v1_genomic	C:99.6%	S:98.5%	D:1.1%	F:0.0%	M:0.4%	n:264
GCA_011010055.1_ASM1101005v1_genomic	C:99.2%	S:98.1%	D:1.1%	F:0.4%	M:0.4%	n:264
GCA_011010065.1_ASM1101006v1_genomic	C:99.6%	S:98.5%	D:1.1%	F:0.0%	M:0.4%	n:264
GCA_011010085.1_ASM1101008v1_genomic	C:99.2%	S:98.1%	D:1.1%	F:0.4%	M:0.4%	n:264
GCA_011010105.1_ASM1101010v1_genomic	C:99.7%	S:98.9%	D:0.8%	F:0.0%	M:0.3%	n:264
GCA_011010135.1_ASM1101013v1_genomic	C:99.6%	S:98.5%	D:1.1%	F:0.0%	M:0.4%	n:264
GCA_011010155.1_ASM1101015v1_genomic	C:98.8%	S:97.7%	D:1.1%	F:0.0%	M:1.2%	n:264
GCA_011010165.1_ASM1101016v1_genomic	C:99.6%	S:98.5%	D:1.1%	F:0.0%	M:0.4%	n:264
GCA_011010175.1_ASM1101017v1_genomic	C:99.6%	S:98.5%	D:1.1%	F:0.0%	M:0.4%	n:264
GCA_011010205.1_ASM1101020v1_genomic	C:99.6%	S:98.5%	D:1.1%	F:0.0%	M:0.4%	n:264
GCA_011010235.1_ASM1101023v1_genomic	C:99.6%	S:98.5%	D:1.1%	F:0.0%	M:0.4%	n:264
GCA_011010245.1_ASM1101024v1_genomic	C:99.6%	S:98.5%	D:1.1%	F:0.0%	M:0.4%	n:264
GCA_011010275.1_ASM1101027v1_genomic	C:99.6%	S:98.5%	D:1.1%	F:0.0%	M:0.4%	n:264
GCA_011010285.1_ASM1101028v1_genomic	C:99.6%	S:98.5%	D:1.1%	F:0.0%	M:0.4%	n:264
GCA_011010295.1_ASM1101029v1_genomic	C:99.6%	S:98.5%	D:1.1%	F:0.0%	M:0.4%	n:264
GCA_011010335.1_ASM1101033v1_genomic	C:99.6%	S:98.5%	D:1.1%	F:0.0%	M:0.4%	n:264
GCA_011010345.1_ASM1101034v1_genomic	C:99.6%	S:98.5%	D:1.1%	F:0.0%	M:0.4%	n:264
GCA_011010355.1_ASM1101035v1_genomic	C:99.6%	S:98.5%	D:1.1%	F:0.0%	M:0.4%	n:264
GCA_011010375.1_ASM1101037v1_genomic	C:99.6%	S:98.5%	D:1.1%	F:0.0%	M:0.4%	n:264
GCA_011010415.1_ASM1101041v1_genomic	C:99.6%	S:98.5%	D:1.1%	F:0.0%	M:0.4%	n:264
GCA_011010435.1_ASM1101043v1_genomic	C:99.2%	S:98.1%	D:1.1%	F:0.4%	M:0.4%	n:264

GCA_011010445.1_ASM1101044v1_genomic	C:99.6%	S:98.5%	D:1.1%	F:0.0%	M:0.4%	n:264
GCA_011010455.1_ASM1101045v1_genomic	C:99.6%	S:98.5%	D:1.1%	F:0.0%	M:0.4%	n:264
GCA_011010485.1_ASM1101048v1_genomic	C:99.7%	S:98.9%	D:0.8%	F:0.0%	M:0.3%	n:264
GCA_011010505.1_ASM1101050v1_genomic	C:99.7%	S:98.9%	D:0.8%	F:0.0%	M:0.3%	n:264
GCA_011010535.1_ASM1101053v1_genomic	C:99.7%	S:98.9%	D:0.8%	F:0.0%	M:0.3%	n:264
GCA_011010545.1_ASM1101054v1_genomic	C:99.7%	S:98.9%	D:0.8%	F:0.0%	M:0.3%	n:264
GCA_011010575.1_ASM1101057v1_genomic	C:98.8%	S:97.7%	D:1.1%	F:0.0%	M:1.2%	n:264
GCA_011010615.1_ASM1101061v1_genomic	C:99.6%	S:98.5%	D:1.1%	F:0.0%	M:0.4%	n:264
GCA_011010635.1_ASM1101063v1_genomic	C:99.6%	S:98.5%	D:1.1%	F:0.0%	M:0.4%	n:264
GCA_011010645.1_ASM1101064v1_genomic	C:99.6%	S:98.5%	D:1.1%	F:0.0%	M:0.4%	n:264
GCA_011010655.1_ASM1101065v1_genomic	C:99.6%	S:98.5%	D:1.1%	F:0.0%	M:0.4%	n:264
GCA_011010685.1_ASM1101068v1_genomic	C:91.6%	S:90.5%	D:1.1%	F:0.8%	M:7.6%	n:264
GCA_011010715.1_ASM1101071v1_genomic	C:99.2%	S:98.1%	D:1.1%	F:0.4%	M:0.4%	n:264
GCA_011010735.1_ASM1101073v1_genomic	C:99.6%	S:98.5%	D:1.1%	F:0.0%	M:0.4%	n:264
GCA_011010745.1_ASM1101074v1_genomic	C:99.6%	S:98.5%	D:1.1%	F:0.0%	M:0.4%	n:264
GCA_011010775.1_ASM1101077v1_genomic	C:99.7%	S:98.9%	D:0.8%	F:0.0%	M:0.3%	n:264
GCA_011010785.1_ASM1101078v1_genomic	C:99.6%	S:98.5%	D:1.1%	F:0.0%	M:0.4%	n:264
GCA_011010835.1_ASM1101083v1_genomic	C:99.6%	S:98.5%	D:1.1%	F:0.0%	M:0.4%	n:264
GCA_011010865.1_ASM1101086v1_genomic	C:99.6%	S:98.5%	D:1.1%	F:0.0%	M:0.4%	n:264
GCA_011010895.1_ASM1101089v1_genomic	C:99.6%	S:98.5%	D:1.1%	F:0.0%	M:0.4%	n:264
GCA_011010905.1_ASM1101090v1_genomic	C:99.6%	S:98.5%	D:1.1%	F:0.0%	M:0.4%	n:264
GCA_011010935.1_ASM1101093v1_genomic	C:99.7%	S:98.9%	D:0.8%	F:0.0%	M:0.3%	n:264
GCA_011010945.1_ASM1101094v1_genomic	C:99.7%	S:98.9%	D:0.8%	F:0.0%	M:0.3%	n:264
GCA_011010965.1_ASM1101096v1_genomic	C:99.6%	S:98.5%	D:1.1%	F:0.0%	M:0.4%	n:264
GCA_011010975.1_ASM1101097v1_genomic	C:99.6%	S:98.5%	D:1.1%	F:0.0%	M:0.4%	n:264
GCA_011011015.1_ASM1101101v1_genomic	C:99.6%	S:98.5%	D:1.1%	F:0.0%	M:0.4%	n:264

GCA_011011035.1_ASM1101103v1_genomic	C:99.6%	S:98.5%	D:1.1%	F:0.0%	M:0.4%	n:264
GCA_011011045.1_ASM1101104v1_genomic	C:99.6%	S:98.5%	D:1.1%	F:0.0%	M:0.4%	n:264
GCA_011011075.1_ASM1101107v1_genomic	C:99.6%	S:98.5%	D:1.1%	F:0.0%	M:0.4%	n:264
GCA_011011095.1_ASM1101109v1_genomic	C:99.6%	S:98.5%	D:1.1%	F:0.0%	M:0.4%	n:264
GCA_011011105.1_ASM1101110v1_genomic	C:99.6%	S:98.5%	D:1.1%	F:0.0%	M:0.4%	n:264
GCA_011011135.1_ASM1101113v1_genomic	C:99.6%	S:98.5%	D:1.1%	F:0.0%	M:0.4%	n:264
GCA_011011145.1_ASM1101114v1_genomic	C:99.6%	S:98.5%	D:1.1%	F:0.0%	M:0.4%	n:264
GCA_011011165.1_ASM1101116v1_genomic	C:99.6%	S:98.5%	D:1.1%	F:0.0%	M:0.4%	n:264
GCA_011011185.1_ASM1101118v1_genomic	C:99.6%	S:98.5%	D:1.1%	F:0.0%	M:0.4%	n:264
GCA_011011215.1_ASM1101121v1_genomic	C:99.6%	S:98.5%	D:1.1%	F:0.0%	M:0.4%	n:264
GCA_011011235.1_ASM1101123v1_genomic	C:99.2%	S:98.1%	D:1.1%	F:0.4%	M:0.4%	n:264
GCA_011011245.1_ASM1101124v1_genomic	C:99.6%	S:98.5%	D:1.1%	F:0.0%	M:0.4%	n:264
GCA_011011255.1_ASM1101125v1_genomic	C:99.6%	S:98.5%	D:1.1%	F:0.0%	M:0.4%	n:264
GCA_011011295.1_ASM1101129v1_genomic	C:99.7%	S:98.9%	D:0.8%	F:0.0%	M:0.3%	n:264
GCA_011011315.1_ASM1101131v1_genomic	C:99.6%	S:98.5%	D:1.1%	F:0.0%	M:0.4%	n:264
GCA_011011335.1_ASM1101133v1_genomic	C:99.6%	S:98.5%	D:1.1%	F:0.0%	M:0.4%	n:264
GCA_011011345.1_ASM1101134v1_genomic	C:99.6%	S:98.5%	D:1.1%	F:0.0%	M:0.4%	n:264
GCA_011011375.1_ASM1101137v1_genomic	C:99.6%	S:98.5%	D:1.1%	F:0.0%	M:0.4%	n:264
GCA_011011385.1_ASM1101138v1_genomic	C:99.6%	S:98.5%	D:1.1%	F:0.0%	M:0.4%	n:264
GCA_011011415.1_ASM1101141v1_genomic	C:99.6%	S:98.5%	D:1.1%	F:0.0%	M:0.4%	n:264
GCA_011011435.1_ASM1101143v1_genomic	C:99.6%	S:98.5%	D:1.1%	F:0.0%	M:0.4%	n:264
GCA_011011445.1_ASM1101144v1_genomic	C:99.2%	S:98.1%	D:1.1%	F:0.4%	M:0.4%	n:264
GCA_011011465.1_ASM1101146v1_genomic	C:99.6%	S:98.5%	D:1.1%	F:0.0%	M:0.4%	n:264
GCA_011011495.1_ASM1101149v1_genomic	C:99.6%	S:98.5%	D:1.1%	F:0.0%	M:0.4%	n:264
GCA_011011515.1_ASM1101151v1_genomic	C:99.6%	S:98.5%	D:1.1%	F:0.0%	M:0.4%	n:264
GCA_011011535.1_ASM1101153v1_genomic	C:86.0%	S:85.2%	D:0.8%	F:1.1%	M:12.9	n:264

					%	
GCA_011011545.1_ASM1101154v1_genomic	C:99.7%	S:98.9%	D:0.8%	F:0.0%	M:0.3%	n:264
GCA_011011575.1_ASM1101157v1_genomic	C:97.7%	S:96.6%	D:1.1%	F:1.5%	M:0.8%	n:264
GCA_011011585.1_ASM1101158v1_genomic	C:99.6%	S:98.5%	D:1.1%	F:0.0%	M:0.4%	n:264
GCA_011011615.1_ASM1101161v1_genomic	C:99.6%	S:98.5%	D:1.1%	F:0.0%	M:0.4%	n:264
GCA_011011635.1_ASM1101163v1_genomic	C:99.6%	S:98.5%	D:1.1%	F:0.0%	M:0.4%	n:264
GCA_011011645.1_ASM1101164v1_genomic	C:98.1%	S:97.0%	D:1.1%	F:0.8%	M:1.1%	n:264
GCA_011011665.1_ASM1101166v1_genomic	C:99.6%	S:98.5%	D:1.1%	F:0.0%	M:0.4%	n:264
GCA_011011695.1_ASM1101169v1_genomic	C:99.6%	S:98.5%	D:1.1%	F:0.0%	M:0.4%	n:264
GCA_011011715.1_ASM1101171v1_genomic	C:99.2%	S:98.1%	D:1.1%	F:0.0%	M:0.8%	n:264
GCA_011011725.1_ASM1101172v1_genomic	C:99.2%	S:98.1%	D:1.1%	F:0.0%	M:0.8%	n:264
GCA_011011735.1_ASM1101173v1_genomic	C:98.8%	S:97.7%	D:1.1%	F:0.0%	M:1.2%	n:264
GCA_011011755.1_ASM1101175v1_genomic	C:99.2%	S:98.1%	D:1.1%	F:0.0%	M:0.8%	n:264
GCA_011011765.1_ASM1101176v1_genomic	C:99.6%	S:98.5%	D:1.1%	F:0.0%	M:0.4%	n:264
GCA_011011815.1_ASM1101181v1_genomic	C:99.3%	S:98.5%	D:0.8%	F:0.0%	M:0.7%	n:264
GCA_011011835.1_ASM1101183v1_genomic	C:99.2%	S:98.1%	D:1.1%	F:0.0%	M:0.8%	n:264
GCA_011011845.1_ASM1101184v1_genomic	C:98.8%	S:97.7%	D:1.1%	F:0.8%	M:0.4%	n:264
GCA_011011865.1_ASM1101186v1_genomic	C:99.6%	S:98.5%	D:1.1%	F:0.0%	M:0.4%	n:264
GCA_011011885.1_ASM1101188v1_genomic	C:99.6%	S:98.5%	D:1.1%	F:0.0%	M:0.4%	n:264
GCA_011011895.1_ASM1101189v1_genomic	C:99.6%	S:98.5%	D:1.1%	F:0.0%	M:0.4%	n:264
GCA_011011925.1_ASM1101192v1_genomic	C:99.6%	S:98.5%	D:1.1%	F:0.0%	M:0.4%	n:264
GCA_011011955.1_ASM1101195v1_genomic	C:99.2%	S:98.1%	D:1.1%	F:0.4%	M:0.4%	n:264
GCA_011011965.1_ASM1101196v1_genomic	C:99.6%	S:98.5%	D:1.1%	F:0.0%	M:0.4%	n:264
GCA_011011995.1_ASM1101199v1_genomic	C:99.6%	S:98.5%	D:1.1%	F:0.0%	M:0.4%	n:264
GCA_011012005.1_ASM1101200v1_genomic	C:99.6%	S:97.3%	D:2.3%	F:0.0%	M:0.4%	n:264
GCA_011012015.1_ASM1101201v1_genomic	C:99.7%	S:98.9%	D:0.8%	F:0.0%	M:0.3%	n:264

GCA_011012055.1_ASM1101205v1_genomic	C:99.6%	S:98.5%	D:1.1%	F:0.0%	M:0.4%	n:264
GCA_011012075.1_ASM1101207v1_genomic	C:99.6%	S:98.5%	D:1.1%	F:0.0%	M:0.4%	n:264
GCA_011012095.1_ASM1101209v1_genomic	C:99.6%	S:98.5%	D:1.1%	F:0.0%	M:0.4%	n:264
GCA_011012105.1_ASM1101210v1_genomic	C:99.6%	S:98.5%	D:1.1%	F:0.0%	M:0.4%	n:264
GCA_011012125.1_ASM1101212v1_genomic	C:98.8%	S:97.7%	D:1.1%	F:0.8%	M:0.4%	n:264
GCA_011012155.1_ASM1101215v1_genomic	C:99.6%	S:98.5%	D:1.1%	F:0.0%	M:0.4%	n:264
GCA_011012165.1_ASM1101216v1_genomic	C:99.6%	S:98.5%	D:1.1%	F:0.0%	M:0.4%	n:264
GCA_011012195.1_ASM1101219v1_genomic	C:99.2%	S:98.1%	D:1.1%	F:0.4%	M:0.4%	n:264
GCA_011012215.1_ASM1101221v1_genomic	C:99.6%	S:98.5%	D:1.1%	F:0.0%	M:0.4%	n:264
GCA_011012225.1_ASM1101222v1_genomic	C:99.6%	S:98.5%	D:1.1%	F:0.0%	M:0.4%	n:264
GCA_011012355.1_ASM1101235v1_genomic	C:99.6%	S:96.2%	D:3.4%	F:0.0%	M:0.4%	n:264
GCA_011012365.1_ASM1101236v1_genomic	C:99.6%	S:98.1%	D:1.5%	F:0.0%	M:0.4%	n:264
GCA_011012375.1_ASM1101237v1_genomic	C:99.6%	S:98.1%	D:1.5%	F:0.0%	M:0.4%	n:264
GCA_011012435.1_ASM1101243v1_genomic	C:99.6%	S:98.5%	D:1.1%	F:0.0%	M:0.4%	n:264
GCA_011012455.1_ASM1101245v1_genomic	C:99.6%	S:98.5%	D:1.1%	F:0.0%	M:0.4%	n:264
GCA_011012465.1_ASM1101246v1_genomic	C:99.6%	S:98.5%	D:1.1%	F:0.0%	M:0.4%	n:264
GCA_011012695.1_ASM1101269v1_genomic	C:98.1%	S:97.0%	D:1.1%	F:0.8%	M:1.1%	n:264
GCA_011012725.1_ASM1101272v1_genomic	C:99.6%	S:98.5%	D:1.1%	F:0.0%	M:0.4%	n:264
GCA_011012735.1_ASM1101273v1_genomic	C:99.2%	S:97.7%	D:1.5%	F:0.0%	M:0.8%	n:264
GCA_011012755.1_ASM1101275v1_genomic	C:99.6%	S:98.1%	D:1.5%	F:0.0%	M:0.4%	n:264
GCA_011012795.1_ASM1101279v1_genomic	C:99.6%	S:98.1%	D:1.5%	F:0.0%	M:0.4%	n:264
GCA_011012815.1_ASM1101281v1_genomic	C:99.6%	S:98.1%	D:1.5%	F:0.0%	M:0.4%	n:264
GCA_011012825.1_ASM1101282v1_genomic	C:99.6%	S:98.1%	D:1.5%	F:0.0%	M:0.4%	n:264
GCA_011012835.1_ASM1101283v1_genomic	C:99.6%	S:98.5%	D:1.1%	F:0.0%	M:0.4%	n:264
GCA_011012875.1_ASM1101287v1_genomic	C:99.6%	S:98.5%	D:1.1%	F:0.0%	M:0.4%	n:264
GCA_011012885.1_ASM1101288v1_genomic	C:99.6%	S:98.1%	D:1.5%	F:0.0%	M:0.4%	n:264

GCA_011013055.1_ASM1101305v1_genomic	C:99.6%	S:98.5%	D:1.1%	F:0.0%	M:0.4%	n:264
GCA_011013175.1_ASM1101317v1_genomic	C:94.3%	S:93.2%	D:1.1%	F:1.1%	M:4.6%	n:264
GCA_011013235.1_ASM1101323v1_genomic	C:99.7%	S:98.9%	D:0.8%	F:0.0%	M:0.3%	n:264
GCA_011013255.1_ASM1101325v1_genomic	C:99.6%	S:98.1%	D:1.5%	F:0.0%	M:0.4%	n:264
GCA_011013265.1_ASM1101326v1_genomic	C:99.2%	S:98.1%	D:1.1%	F:0.4%	M:0.4%	n:264
GCA_011013315.1_ASM1101331v1_genomic	C:99.7%	S:98.9%	D:0.8%	F:0.0%	M:0.3%	n:264
GCA_011013335.1_ASM1101333v1_genomic	C:99.2%	S:98.1%	D:1.1%	F:0.4%	M:0.4%	n:264
GCA_011013435.1_ASM1101343v1_genomic	C:99.6%	S:98.5%	D:1.1%	F:0.0%	M:0.4%	n:264
GCA_011013545.1_ASM1101354v1_genomic	C:99.6%	S:98.5%	D:1.1%	F:0.0%	M:0.4%	n:264
GCA_011013585.1_ASM1101358v1_genomic	C:99.6%	S:98.5%	D:1.1%	F:0.0%	M:0.4%	n:264
GCA_011013615.1_ASM1101361v1_genomic	C:99.6%	S:98.1%	D:1.5%	F:0.0%	M:0.4%	n:264
GCA_011013635.1_ASM1101363v1_genomic	C:99.6%	S:98.1%	D:1.5%	F:0.0%	M:0.4%	n:264
GCA_011013675.1_ASM1101367v1_genomic	C:99.2%	S:97.7%	D:1.5%	F:0.4%	M:0.4%	n:264
GCA_011013685.1_ASM1101368v1_genomic	C:99.3%	S:98.5%	D:0.8%	F:0.4%	M:0.3%	n:264
GCA_011013735.1_ASM1101373v1_genomic	C:99.6%	S:98.5%	D:1.1%	F:0.0%	M:0.4%	n:264
GCA_011013765.1_ASM1101376v1_genomic	C:99.7%	S:98.9%	D:0.8%	F:0.0%	M:0.3%	n:264
GCA_011013785.1_ASM1101378v1_genomic	C:99.2%	S:98.1%	D:1.1%	F:0.4%	M:0.4%	n:264
GCA_011013815.1_ASM1101381v1_genomic	C:99.6%	S:98.1%	D:1.5%	F:0.0%	M:0.4%	n:264
GCA_011013835.1_ASM1101383v1_genomic	C:99.2%	S:98.1%	D:1.1%	F:0.4%	M:0.4%	n:264
GCA_011013865.1_ASM1101386v1_genomic	C:99.2%	S:97.7%	D:1.5%	F:0.4%	M:0.4%	n:264
GCA_011013885.1_ASM1101388v1_genomic	C:99.6%	S:97.7%	D:1.9%	F:0.0%	M:0.4%	n:264
GCA_011013905.1_ASM1101390v1_genomic	C:99.2%	S:97.7%	D:1.5%	F:0.4%	M:0.4%	n:264
GCA_011013935.1_ASM1101393v1_genomic	C:99.6%	S:98.5%	D:1.1%	F:0.0%	M:0.4%	n:264
GCA_011013955.1_ASM1101395v1_genomic	C:98.9%	S:98.1%	D:0.8%	F:0.4%	M:0.7%	n:264
GCA_011013965.1_ASM1101396v1_genomic	C:99.6%	S:98.1%	D:1.5%	F:0.0%	M:0.4%	n:264
GCA_011013975.1_ASM1101397v1_genomic	C:98.8%	S:97.7%	D:1.1%	F:0.4%	M:0.8%	n:264

GCA_011014015.1_ASM1101401v1_genomic	C:99.6%	S:98.5%	D:1.1%	F:0.0%	M:0.4%	n:264
GCA_011014035.1_ASM1101403v1_genomic	C:99.6%	S:98.5%	D:1.1%	F:0.0%	M:0.4%	n:264
GCA_011014055.1_ASM1101405v1_genomic	C:99.6%	S:98.1%	D:1.5%	F:0.0%	M:0.4%	n:264
GCA_011014085.1_ASM1101408v1_genomic	C:99.2%	S:97.7%	D:1.5%	F:0.4%	M:0.4%	n:264
GCA_011014095.1_ASM1101409v1_genomic	C:99.6%	S:98.1%	D:1.5%	F:0.0%	M:0.4%	n:264
GCA_011014135.1_ASM1101413v1_genomic	C:99.2%	S:97.7%	D:1.5%	F:0.4%	M:0.4%	n:264
GCA_011014155.1_ASM1101415v1_genomic	C:99.6%	S:98.1%	D:1.5%	F:0.0%	M:0.4%	n:264
GCA_011014175.1_ASM1101417v1_genomic	C:99.6%	S:98.1%	D:1.5%	F:0.0%	M:0.4%	n:264
GCA_011014195.1_ASM1101419v1_genomic	C:99.6%	S:98.1%	D:1.5%	F:0.0%	M:0.4%	n:264
GCA_011014215.1_ASM1101421v1_genomic	C:98.8%	S:97.3%	D:1.5%	F:0.4%	M:0.8%	n:264
GCA_011014225.1_ASM1101422v1_genomic	C:99.6%	S:98.1%	D:1.5%	F:0.0%	M:0.4%	n:264
GCA_011014255.1_ASM1101425v1_genomic	C:99.6%	S:98.1%	D:1.5%	F:0.0%	M:0.4%	n:264
GCA_011014265.1_ASM1101426v1_genomic	C:99.6%	S:98.1%	D:1.5%	F:0.0%	M:0.4%	n:264
GCA_011014295.1_ASM1101429v1_genomic	C:99.2%	S:97.7%	D:1.5%	F:0.4%	M:0.4%	n:264
GCA_011014305.1_ASM1101430v1_genomic	C:98.1%	S:96.6%	D:1.5%	F:0.4%	M:1.5%	n:264
GCA_011014335.1_ASM1101433v1_genomic	C:99.6%	S:98.5%	D:1.1%	F:0.0%	M:0.4%	n:264
GCA_011014345.1_ASM1101434v1_genomic	C:98.8%	S:97.7%	D:1.1%	F:0.4%	M:0.8%	n:264
GCA_011014375.1_ASM1101437v1_genomic	C:99.2%	S:98.1%	D:1.1%	F:0.4%	M:0.4%	n:264
GCA_011014395.1_ASM1101439v1_genomic	C:99.6%	S:98.5%	D:1.1%	F:0.0%	M:0.4%	n:264
GCA_011014415.1_ASM1101441v1_genomic	C:99.6%	S:98.5%	D:1.1%	F:0.0%	M:0.4%	n:264
GCA_011014425.1_ASM1101442v1_genomic	C:99.6%	S:98.5%	D:1.1%	F:0.0%	M:0.4%	n:264
GCA_011014445.1_ASM1101444v1_genomic	C:99.3%	S:98.5%	D:0.8%	F:0.4%	M:0.3%	n:264
GCA_011014475.1_ASM1101447v1_genomic	C:99.7%	S:98.9%	D:0.8%	F:0.0%	M:0.3%	n:264
GCA_011014545.1_ASM1101454v1_genomic	C:99.6%	S:98.5%	D:1.1%	F:0.0%	M:0.4%	n:264
GCA_011014565.1_ASM1101456v1_genomic	C:99.6%	S:98.5%	D:1.1%	F:0.0%	M:0.4%	n:264
GCA_011014595.1_ASM1101459v1_genomic	C:99.7%	S:98.9%	D:0.8%	F:0.0%	M:0.3%	n:264

GCA_011014735.1_ASM1101473v1_genomic	C:99.6%	S:98.5%	D:1.1%	F:0.0%	M:0.4%	n:264
GCA_011014745.1_ASM1101474v1_genomic	C:99.6%	S:98.5%	D:1.1%	F:0.0%	M:0.4%	n:264
GCA_011014775.1_ASM1101477v1_genomic	C:99.6%	S:98.5%	D:1.1%	F:0.0%	M:0.4%	n:264
GCA_011014825.1_ASM1101482v1_genomic	C:99.6%	S:98.5%	D:1.1%	F:0.0%	M:0.4%	n:264
GCA_011014925.1_ASM1101492v1_genomic	C:99.6%	S:98.5%	D:1.1%	F:0.0%	M:0.4%	n:264
GCA_011014955.1_ASM1101495v1_genomic	C:99.6%	S:98.5%	D:1.1%	F:0.0%	M:0.4%	n:264
GCA_011014965.1_ASM1101496v1_genomic	C:99.6%	S:98.5%	D:1.1%	F:0.0%	M:0.4%	n:264
GCA_011014995.1_ASM1101499v1_genomic	C:99.2%	S:98.1%	D:1.1%	F:0.0%	M:0.8%	n:264
GCA_011015015.1_ASM1101501v1_genomic	C:99.6%	S:98.5%	D:1.1%	F:0.0%	M:0.4%	n:264
GCA_011015195.1_ASM1101519v1_genomic	C:99.6%	S:98.5%	D:1.1%	F:0.0%	M:0.4%	n:264
GCA_011015305.1_ASM1101530v1_genomic	C:99.6%	S:98.5%	D:1.1%	F:0.0%	M:0.4%	n:264
GCA_011015315.1_ASM1101531v1_genomic	C:99.3%	S:98.5%	D:0.8%	F:0.4%	M:0.3%	n:264
GCA_011015335.1_ASM1101533v1_genomic	C:99.6%	S:98.5%	D:1.1%	F:0.0%	M:0.4%	n:264
GCA_011015395.1_ASM1101539v1_genomic	C:99.2%	S:98.1%	D:1.1%	F:0.4%	M:0.4%	n:264
GCA_011015415.1_ASM1101541v1_genomic	C:99.6%	S:98.5%	D:1.1%	F:0.0%	M:0.4%	n:264
GCA_011015425.1_ASM1101542v1_genomic	C:99.6%	S:98.5%	D:1.1%	F:0.0%	M:0.4%	n:264
GCA_011015435.1_ASM1101543v1_genomic	C:99.2%	S:98.1%	D:1.1%	F:0.4%	M:0.4%	n:264
GCA_011015475.1_ASM1101547v1_genomic	C:99.2%	S:98.1%	D:1.1%	F:0.4%	M:0.4%	n:264
GCA_011015495.1_ASM1101549v1_genomic	C:99.6%	S:98.5%	D:1.1%	F:0.0%	M:0.4%	n:264
GCA_011015505.1_ASM1101550v1_genomic	C:99.6%	S:98.5%	D:1.1%	F:0.0%	M:0.4%	n:264
GCA_011015535.1_ASM1101553v1_genomic	C:99.6%	S:98.5%	D:1.1%	F:0.0%	M:0.4%	n:264
GCA_011015545.1_ASM1101554v1_genomic	C:99.2%	S:98.1%	D:1.1%	F:0.0%	M:0.8%	n:264
GCA_011015555.1_ASM1101555v1_genomic	C:99.2%	S:98.1%	D:1.1%	F:0.0%	M:0.8%	n:264
GCA_011015595.1_ASM1101559v1_genomic	C:99.2%	S:98.1%	D:1.1%	F:0.0%	M:0.8%	n:264
GCA_011015615.1_ASM1101561v1_genomic	C:98.4%	S:97.3%	D:1.1%	F:0.8%	M:0.8%	n:264
GCA_011015635.1_ASM1101563v1_genomic	C:99.6%	S:98.5%	D:1.1%	F:0.0%	M:0.4%	n:264

GCA_011015655.1_ASM1101565v1_genomic	C:99.6%	S:98.5%	D:1.1%	F:0.0%	M:0.4%	n:264
GCA_011015665.1_ASM1101566v1_genomic	C:99.6%	S:98.5%	D:1.1%	F:0.0%	M:0.4%	n:264
GCA_011015685.1_ASM1101568v1_genomic	C:99.6%	S:98.5%	D:1.1%	F:0.0%	M:0.4%	n:264
GCA_011015715.1_ASM1101571v1_genomic	C:99.6%	S:98.5%	D:1.1%	F:0.0%	M:0.4%	n:264
GCA_011015735.1_ASM1101573v1_genomic	C:99.6%	S:98.5%	D:1.1%	F:0.0%	M:0.4%	n:264
GCA_011015795.1_ASM1101579v1_genomic	C:99.2%	S:98.1%	D:1.1%	F:0.0%	M:0.8%	n:264
GCA_011016045.1_ASM1101604v1_genomic	C:99.2%	S:98.1%	D:1.1%	F:0.0%	M:0.8%	n:264
GCA_011016095.1_ASM1101609v1_genomic	C:99.6%	S:98.1%	D:1.5%	F:0.0%	M:0.4%	n:264
GCA_011017095.1_ASM1101709v1_genomic	C:99.6%	S:98.1%	D:1.5%	F:0.0%	M:0.4%	n:264
GCA_011017125.1_ASM1101712v1_genomic	C:99.6%	S:98.5%	D:1.1%	F:0.0%	M:0.4%	n:264
GCA_011017145.1_ASM1101714v1_genomic	C:99.6%	S:98.5%	D:1.1%	F:0.0%	M:0.4%	n:264
GCA_011017175.1_ASM1101717v1_genomic	C:99.6%	S:98.5%	D:1.1%	F:0.0%	M:0.4%	n:264
GCA_011017235.1_ASM1101723v1_genomic	C:99.2%	S:98.1%	D:1.1%	F:0.4%	M:0.4%	n:264
GCA_011017245.1_ASM1101724v1_genomic	C:99.7%	S:98.9%	D:0.8%	F:0.0%	M:0.3%	n:264
GCA_011017265.1_ASM1101726v1_genomic	C:99.7%	S:98.9%	D:0.8%	F:0.0%	M:0.3%	n:264
GCA_011017275.1_ASM1101727v1_genomic	C:99.7%	S:98.9%	D:0.8%	F:0.0%	M:0.3%	n:264
GCA_011017315.1_ASM1101731v1_genomic	C:99.7%	S:98.9%	D:0.8%	F:0.0%	M:0.3%	n:264
GCA_011017375.1_ASM1101737v1_genomic	C:99.6%	S:98.5%	D:1.1%	F:0.0%	M:0.4%	n:264
GCA_011017455.1_ASM1101745v1_genomic	C:99.6%	S:98.5%	D:1.1%	F:0.0%	M:0.4%	n:264
GCA_011017465.1_ASM1101746v1_genomic	C:99.6%	S:98.5%	D:1.1%	F:0.0%	M:0.4%	n:264
GCA_011017495.1_ASM1101749v1_genomic	C:99.6%	S:98.5%	D:1.1%	F:0.0%	M:0.4%	n:264
GCA_011017505.1_ASM1101750v1_genomic	C:99.6%	S:98.5%	D:1.1%	F:0.0%	M:0.4%	n:264
GCA_011017535.1_ASM1101753v1_genomic	C:99.6%	S:98.5%	D:1.1%	F:0.0%	M:0.4%	n:264
GCA_011017545.1_ASM1101754v1_genomic	C:99.6%	S:98.5%	D:1.1%	F:0.0%	M:0.4%	n:264
GCA_011017575.1_ASM1101757v1_genomic	C:99.6%	S:98.5%	D:1.1%	F:0.0%	M:0.4%	n:264
GCA_011017585.1_ASM1101758v1_genomic	C:99.6%	S:98.5%	D:1.1%	F:0.0%	M:0.4%	n:264

GCA_011017615.1_ASM1101761v1_genomic	C:99.6%	S:98.5%	D:1.1%	F:0.0%	M:0.4%	n:264
GCA_011017635.1_ASM1101763v1_genomic	C:99.6%	S:98.5%	D:1.1%	F:0.0%	M:0.4%	n:264
GCA_011017645.1_ASM1101764v1_genomic	C:99.6%	S:98.5%	D:1.1%	F:0.0%	M:0.4%	n:264
GCA_011017655.1_ASM1101765v1_genomic	C:99.7%	S:98.9%	D:0.8%	F:0.0%	M:0.3%	n:264
GCA_011017685.1_ASM1101768v1_genomic	C:99.6%	S:98.5%	D:1.1%	F:0.0%	M:0.4%	n:264
GCA_011017715.1_ASM1101771v1_genomic	C:99.6%	S:98.5%	D:1.1%	F:0.0%	M:0.4%	n:264
GCA_011017735.1_ASM1101773v1_genomic	C:99.6%	S:98.5%	D:1.1%	F:0.0%	M:0.4%	n:264
GCA_011017755.1_ASM1101775v1_genomic	C:99.2%	S:98.1%	D:1.1%	F:0.0%	M:0.8%	n:264
GCA_011017765.1_ASM1101776v1_genomic	C:99.6%	S:98.5%	D:1.1%	F:0.0%	M:0.4%	n:264
GCA_011017775.1_ASM1101777v1_genomic	C:99.6%	S:98.5%	D:1.1%	F:0.0%	M:0.4%	n:264
GCA_011017785.1_ASM1101778v1_genomic	C:99.6%	S:98.5%	D:1.1%	F:0.0%	M:0.4%	n:264
GCA_011017835.1_ASM1101783v1_genomic	C:99.6%	S:98.5%	D:1.1%	F:0.0%	M:0.4%	n:264
GCA_011017845.1_ASM1101784v1_genomic	C:99.6%	S:98.5%	D:1.1%	F:0.0%	M:0.4%	n:264
GCA_011017875.1_ASM1101787v1_genomic	C:99.6%	S:98.5%	D:1.1%	F:0.0%	M:0.4%	n:264
GCA_011017885.1_ASM1101788v1_genomic	C:99.6%	S:98.5%	D:1.1%	F:0.0%	M:0.4%	n:264
GCA_011017895.1_ASM1101789v1_genomic	C:99.6%	S:98.5%	D:1.1%	F:0.0%	M:0.4%	n:264
GCA_011017935.1_ASM1101793v1_genomic	C:99.6%	S:98.5%	D:1.1%	F:0.0%	M:0.4%	n:264
GCA_011017955.1_ASM1101795v1_genomic	C:99.6%	S:98.5%	D:1.1%	F:0.0%	M:0.4%	n:264
GCA_011017965.1_ASM1101796v1_genomic	C:99.6%	S:98.5%	D:1.1%	F:0.0%	M:0.4%	n:264
GCA_011017975.1_ASM1101797v1_genomic	C:99.6%	S:98.5%	D:1.1%	F:0.0%	M:0.4%	n:264
GCA_011017995.1_ASM1101799v1_genomic	C:99.2%	S:97.7%	D:1.5%	F:0.4%	M:0.4%	n:264
GCA_011018275.1_ASM1101827v1_genomic	C:99.7%	S:98.9%	D:0.8%	F:0.0%	M:0.3%	n:264
GCA_011018285.1_ASM1101828v1_genomic	C:99.6%	S:98.5%	D:1.1%	F:0.0%	M:0.4%	n:264
GCA_011018295.1_ASM1101829v1_genomic	C:99.2%	S:98.1%	D:1.1%	F:0.4%	M:0.4%	n:264
GCA_011018385.1_ASM1101838v1_genomic	C:99.7%	S:98.9%	D:0.8%	F:0.0%	M:0.3%	n:264
GCA_011018395.1_ASM1101839v1_genomic	C:99.6%	S:98.5%	D:1.1%	F:0.0%	M:0.4%	n:264

GCA_011018525.1_ASM1101852v1_genomic	C:99.6%	S:98.5%	D:1.1%	F:0.0%	M:0.4%	n:264
GCA_011018575.1_ASM1101857v1_genomic	C:99.6%	S:98.5%	D:1.1%	F:0.0%	M:0.4%	n:264
GCA_011018595.1_ASM1101859v1_genomic	C:99.7%	S:98.9%	D:0.8%	F:0.0%	M:0.3%	n:264
GCA_011018625.1_ASM1101862v1_genomic	C:99.6%	S:98.5%	D:1.1%	F:0.0%	M:0.4%	n:264
GCA_011018675.1_ASM1101867v1_genomic	C:99.6%	S:98.5%	D:1.1%	F:0.0%	M:0.4%	n:264
GCA_011018725.1_ASM1101872v1_genomic	C:98.8%	S:97.3%	D:1.5%	F:0.8%	M:0.4%	n:264
GCA_011018795.1_ASM1101879v1_genomic	C:99.6%	S:98.5%	D:1.1%	F:0.0%	M:0.4%	n:264
GCA_011018815.1_ASM1101881v1_genomic	C:99.6%	S:98.5%	D:1.1%	F:0.0%	M:0.4%	n:264
GCA_011018895.1_ASM1101889v1_genomic	C:98.1%	S:97.0%	D:1.1%	F:0.8%	M:1.1%	n:264
GCA_011018945.1_ASM1101894v1_genomic	C:99.6%	S:98.5%	D:1.1%	F:0.0%	M:0.4%	n:264
GCA_011018995.1_ASM1101899v1_genomic	C:99.6%	S:98.5%	D:1.1%	F:0.0%	M:0.4%	n:264
GCA_011019005.1_ASM1101900v1_genomic	C:99.6%	S:98.5%	D:1.1%	F:0.0%	M:0.4%	n:264
GCA_011019055.1_ASM1101905v1_genomic	C:99.6%	S:98.5%	D:1.1%	F:0.0%	M:0.4%	n:264
GCA_011019065.1_ASM1101906v1_genomic	C:99.6%	S:98.5%	D:1.1%	F:0.0%	M:0.4%	n:264
GCA_011019095.1_ASM1101909v1_genomic	C:99.6%	S:98.5%	D:1.1%	F:0.0%	M:0.4%	n:264
GCA_011019125.1_ASM1101912v1_genomic	C:99.6%	S:98.5%	D:1.1%	F:0.0%	M:0.4%	n:264
GCA_011019165.1_ASM1101916v1_genomic	C:99.6%	S:98.5%	D:1.1%	F:0.0%	M:0.4%	n:264
GCA_011019215.1_ASM1101921v1_genomic	C:99.6%	S:98.5%	D:1.1%	F:0.0%	M:0.4%	n:264
GCA_011019415.1_ASM1101941v1_genomic	C:99.2%	S:98.1%	D:1.1%	F:0.4%	M:0.4%	n:264
GCA_011019425.1_ASM1101942v1_genomic	C:99.6%	S:98.5%	D:1.1%	F:0.0%	M:0.4%	n:264
GCA_011019455.1_ASM1101945v1_genomic	C:98.1%	S:97.0%	D:1.1%	F:0.8%	M:1.1%	n:264
GCA_011019525.1_ASM1101952v1_genomic	C:99.7%	S:98.9%	D:0.8%	F:0.0%	M:0.3%	n:264
GCA_011019625.1_ASM1101962v1_genomic	C:99.7%	S:98.9%	D:0.8%	F:0.0%	M:0.3%	n:264
GCA_011019745.1_ASM1101974v1_genomic	C:99.6%	S:98.5%	D:1.1%	F:0.0%	M:0.4%	n:264
GCA_011019775.1_ASM1101977v1_genomic	C:99.6%	S:98.1%	D:1.5%	F:0.0%	M:0.4%	n:264
GCA_011019895.1_ASM1101989v1_genomic	C:99.6%	S:98.1%	D:1.5%	F:0.0%	M:0.4%	n:264

GCA_011019915.1_ASM1101991v1_genomic	C:99.2%	S:97.7%	D:1.5%	F:0.4%	M:0.4%	n:264
GCA_011019945.1_ASM1101994v1_genomic	C:99.6%	S:98.5%	D:1.1%	F:0.0%	M:0.4%	n:264
GCA_011019975.1_ASM1101997v1_genomic	C:99.6%	S:98.1%	D:1.5%	F:0.0%	M:0.4%	n:264
GCA_011020005.1_ASM1102000v1_genomic	C:99.6%	S:98.5%	D:1.1%	F:0.0%	M:0.4%	n:264
GCA_011020015.1_ASM1102001v1_genomic	C:99.6%	S:98.5%	D:1.1%	F:0.0%	M:0.4%	n:264
GCA_011020075.1_ASM1102007v1_genomic	C:99.6%	S:98.5%	D:1.1%	F:0.0%	M:0.4%	n:264
GCA_011020115.1_ASM1102011v1_genomic	C:99.6%	S:98.5%	D:1.1%	F:0.0%	M:0.4%	n:264
GCA_011020175.1_ASM1102017v1_genomic	C:98.8%	S:97.7%	D:1.1%	F:0.8%	M:0.4%	n:264
GCA_011020195.1_ASM1102019v1_genomic	C:99.6%	S:98.5%	D:1.1%	F:0.0%	M:0.4%	n:264
GCA_011020255.1_ASM1102025v1_genomic	C:99.6%	S:98.1%	D:1.5%	F:0.0%	M:0.4%	n:264
GCA_011020275.1_ASM1102027v1_genomic	C:99.6%	S:98.5%	D:1.1%	F:0.0%	M:0.4%	n:264
GCA_011020285.1_ASM1102028v1_genomic	C:99.7%	S:98.9%	D:0.8%	F:0.0%	M:0.3%	n:264
GCA_011020525.1_ASM1102052v1_genomic	C:99.6%	S:98.1%	D:1.5%	F:0.0%	M:0.4%	n:264
GCA_011020535.1_ASM1102053v1_genomic	C:98.8%	S:97.3%	D:1.5%	F:0.8%	M:0.4%	n:264
GCA_011020595.1_ASM1102059v1_genomic	C:99.2%	S:97.7%	D:1.5%	F:0.4%	M:0.4%	n:264
GCA_011020655.1_ASM1102065v1_genomic	C:99.6%	S:98.1%	D:1.5%	F:0.0%	M:0.4%	n:264
GCA_011020665.1_ASM1102066v1_genomic	C:99.6%	S:98.5%	D:1.1%	F:0.0%	M:0.4%	n:264
GCA_011020735.1_ASM1102073v1_genomic	C:93.9%	S:92.8%	D:1.1%	F:0.8%	M:5.3%	n:264
GCA_011020815.1_ASM1102081v1_genomic	C:99.2%	S:98.1%	D:1.1%	F:0.4%	M:0.4%	n:264
GCA_011020875.1_ASM1102087v1_genomic	C:99.6%	S:98.5%	D:1.1%	F:0.0%	M:0.4%	n:264
GCA_011020895.1_ASM1102089v1_genomic	C:99.6%	S:98.5%	D:1.1%	F:0.0%	M:0.4%	n:264
GCA_011020915.1_ASM1102091v1_genomic	C:99.6%	S:98.5%	D:1.1%	F:0.0%	M:0.4%	n:264
GCA_011020965.1_ASM1102096v1_genomic	C:99.6%	S:98.5%	D:1.1%	F:0.0%	M:0.4%	n:264
GCA_011020995.1_ASM1102099v1_genomic	C:99.6%	S:98.5%	D:1.1%	F:0.0%	M:0.4%	n:264
GCA_011021125.1_ASM1102112v1_genomic	C:99.6%	S:98.5%	D:1.1%	F:0.0%	M:0.4%	n:264
GCA_011021215.1_ASM1102121v1_genomic	C:99.7%	S:98.9%	D:0.8%	F:0.0%	M:0.3%	n:264

GCA_011021325.1_ASM1102132v1_genomic	C:99.7%	S:98.9%	D:0.8%	F:0.0%	M:0.3%	n:264
GCA_011021335.1_ASM1102133v1_genomic	C:99.6%	S:98.5%	D:1.1%	F:0.0%	M:0.4%	n:264
GCA_011021375.1_ASM1102137v1_genomic	C:99.2%	S:98.1%	D:1.1%	F:0.4%	M:0.4%	n:264
GCA_011021415.1_ASM1102141v1_genomic	C:99.7%	S:98.9%	D:0.8%	F:0.0%	M:0.3%	n:264
GCA_011021435.1_ASM1102143v1_genomic	C:99.6%	S:98.5%	D:1.1%	F:0.0%	M:0.4%	n:264
GCA_011021495.1_ASM1102149v1_genomic	C:99.6%	S:98.5%	D:1.1%	F:0.0%	M:0.4%	n:264
GCA_011021515.1_ASM1102151v1_genomic	C:99.6%	S:98.5%	D:1.1%	F:0.0%	M:0.4%	n:264
GCA_011021535.1_ASM1102153v1_genomic	C:99.7%	S:98.9%	D:0.8%	F:0.0%	M:0.3%	n:264
GCA_011021545.1_ASM1102154v1_genomic	C:99.6%	S:98.5%	D:1.1%	F:0.0%	M:0.4%	n:264
GCA_011021565.1_ASM1102156v1_genomic	C:99.7%	S:98.9%	D:0.8%	F:0.0%	M:0.3%	n:264
GCA_011021615.1_ASM1102161v1_genomic	C:99.6%	S:98.5%	D:1.1%	F:0.0%	M:0.4%	n:264
GCA_011021655.1_ASM1102165v1_genomic	C:99.7%	S:98.9%	D:0.8%	F:0.0%	M:0.3%	n:264
GCA_011021735.1_ASM1102173v1_genomic	C:99.6%	S:98.5%	D:1.1%	F:0.0%	M:0.4%	n:264
GCA_011021755.1_ASM1102175v1_genomic	C:99.7%	S:98.9%	D:0.8%	F:0.0%	M:0.3%	n:264
GCA_011021765.1_ASM1102176v1_genomic	C:99.2%	S:97.7%	D:1.5%	F:0.4%	M:0.4%	n:264
GCA_011021895.1_ASM1102189v1_genomic	C:99.2%	S:97.7%	D:1.5%	F:0.4%	M:0.4%	n:264
GCA_011021905.1_ASM1102190v1_genomic	C:99.6%	S:98.1%	D:1.5%	F:0.0%	M:0.4%	n:264
GCA_011021915.1_ASM1102191v1_genomic	C:99.6%	S:98.1%	D:1.5%	F:0.0%	M:0.4%	n:264
GCA_011021955.1_ASM1102195v1_genomic	C:99.2%	S:98.1%	D:1.1%	F:0.0%	M:0.8%	n:264
GCA_011022075.1_ASM1102207v1_genomic	C:98.4%	S:97.3%	D:1.1%	F:1.5%	M:0.1%	n:264
GCA_011022085.1_ASM1102208v1_genomic	C:99.6%	S:98.5%	D:1.1%	F:0.0%	M:0.4%	n:264
GCA_011363175.1_ASM1136317v1_genomic	C:99.2%	S:98.1%	D:1.1%	F:0.0%	M:0.8%	n:264
GCA_011389495.1_ASM1138949v1_genomic	C:99.6%	S:98.5%	D:1.1%	F:0.0%	M:0.4%	n:264
GCA_014068615.1_ASM1406861v1_genomic	C:99.6%	S:98.5%	D:1.1%	F:0.0%	M:0.4%	n:264
GCA_014805065.1_ASM1480506v1_genomic	C:99.6%	S:98.5%	D:1.1%	F:0.0%	M:0.4%	n:264
GCA_014805085.1_ASM1480508v1_genomic	C:99.7%	S:98.9%	D:0.8%	F:0.0%	M:0.3%	n:264

GCA_014805095.1_ASM1480509v1_genomic	C:99.6%	S:98.5%	D:1.1%	F:0.0%	M:0.4%	n:264
GCA_014805105.1_ASM1480510v1_genomic	C:98.4%	S:97.3%	D:1.1%	F:1.1%	M:0.5%	n:264
GCA_014805145.1_ASM1480514v1_genomic	C:99.6%	S:98.5%	D:1.1%	F:0.0%	M:0.4%	n:264
GCA_014805165.1_ASM1480516v1_genomic	C:99.6%	S:98.5%	D:1.1%	F:0.0%	M:0.4%	n:264
GCA_014805185.1_ASM1480518v1_genomic	C:99.7%	S:98.9%	D:0.8%	F:0.0%	M:0.3%	n:264
GCA_014805195.1_ASM1480519v1_genomic	C:99.3%	S:98.5%	D:0.8%	F:0.4%	M:0.3%	n:264
GCA_014805215.1_ASM1480521v1_genomic	C:99.7%	S:98.9%	D:0.8%	F:0.0%	M:0.3%	n:264
GCA_014805235.1_ASM1480523v1_genomic	C:99.6%	S:98.5%	D:1.1%	F:0.0%	M:0.4%	n:264
GCA_014805255.1_ASM1480525v1_genomic	C:99.6%	S:98.5%	D:1.1%	F:0.0%	M:0.4%	n:264
GCA_014805275.1_ASM1480527v1_genomic	C:99.7%	S:98.9%	D:0.8%	F:0.0%	M:0.3%	n:264
GCA_014805305.1_ASM1480530v1_genomic	C:99.3%	S:98.5%	D:0.8%	F:0.4%	M:0.3%	n:264
GCA_014872905.1_ASM1487290v1_genomic	C:99.7%	S:98.9%	D:0.8%	F:0.0%	M:0.3%	n:264
GCA_016085375.1_PDT000162924.3_genomic	C:99.7%	S:98.9%	D:0.8%	F:0.0%	M:0.3%	n:264
GCA_016085455.1_PDT000162920.3_genomic	C:99.7%	S:98.9%	D:0.8%	F:0.0%	M:0.3%	n:264
GCA_016085475.1_PDT000162923.3_genomic	C:99.7%	S:98.9%	D:0.8%	F:0.0%	M:0.3%	n:264
GCA_016085495.1_PDT000162918.3_genomic	C:99.7%	S:98.9%	D:0.8%	F:0.0%	M:0.3%	n:264
GCA_016085515.1_PDT000162922.3_genomic	C:99.7%	S:98.9%	D:0.8%	F:0.0%	M:0.3%	n:264
GCA_016085535.1_PDT000162917.3_genomic	C:99.7%	S:98.9%	D:0.8%	F:0.0%	M:0.3%	n:264
GCA_016085555.1_PDT000162921.3_genomic	C:99.7%	S:98.9%	D:0.8%	F:0.0%	M:0.3%	n:264
GCA_016085625.1_PDT000162916.3_genomic	C:99.7%	S:98.9%	D:0.8%	F:0.0%	M:0.3%	n:264
GCA_016085715.1_PDT000162915.2_genomic	C:99.7%	S:98.9%	D:0.8%	F:0.0%	M:0.3%	n:264
GCA_016798345.1_ASM1679834v1_genomic	C:99.6%	S:98.5%	D:1.1%	F:0.0%	M:0.4%	n:264
GCA_016838665.1_ASM1683866v1_genomic	C:99.7%	S:98.9%	D:0.8%	F:0.0%	M:0.3%	n:264
GCA_016989565.1_ASM1698956v1_genomic	C:99.7%	S:98.9%	D:0.8%	F:0.0%	M:0.3%	n:264
GCA_016989605.1_ASM1698960v1_genomic	C:99.6%	S:98.5%	D:1.1%	F:0.0%	M:0.4%	n:264
GCA_016989635.1_ASM1698963v1_genomic	C:99.6%	S:98.5%	D:1.1%	F:0.0%	M:0.4%	n:264

GCA_016989715.1_ASM1698971v1_genomic	C:99.6%	S:98.5%	D:1.1%	F:0.0%	M:0.4%	n:264
GCA_016989755.1_ASM1698975v1_genomic	C:99.6%	S:98.5%	D:1.1%	F:0.0%	M:0.4%	n:264
GCA_016989775.1_ASM1698977v1_genomic	C:99.6%	S:98.5%	D:1.1%	F:0.0%	M:0.4%	n:264
GCA_016989785.1_ASM1698978v1_genomic	C:99.6%	S:98.5%	D:1.1%	F:0.0%	M:0.4%	n:264
GCA_016989825.1_ASM1698982v1_genomic	C:99.6%	S:98.5%	D:1.1%	F:0.0%	M:0.4%	n:264
GCA_016989855.1_ASM1698985v1_genomic	C:99.6%	S:98.5%	D:1.1%	F:0.0%	M:0.4%	n:264
GCA_016989895.1_ASM1698989v1_genomic	C:99.6%	S:98.5%	D:1.1%	F:0.0%	M:0.4%	n:264
GCA_016989975.1_ASM1698997v1_genomic	C:99.6%	S:98.5%	D:1.1%	F:0.0%	M:0.4%	n:264
GCA_016990015.1_ASM1699001v1_genomic	C:99.6%	S:98.5%	D:1.1%	F:0.0%	M:0.4%	n:264
GCA_016990025.1_ASM1699002v1_genomic	C:99.6%	S:98.5%	D:1.1%	F:0.0%	M:0.4%	n:264
GCA_016990055.1_ASM1699005v1_genomic	C:99.6%	S:98.5%	D:1.1%	F:0.0%	M:0.4%	n:264
GCA_016990065.1_ASM1699006v1_genomic	C:99.6%	S:98.5%	D:1.1%	F:0.0%	M:0.4%	n:264
GCA_016990115.1_ASM1699011v1_genomic	C:99.6%	S:98.5%	D:1.1%	F:0.0%	M:0.4%	n:264
GCA_016990155.1_ASM1699015v1_genomic	C:99.6%	S:98.5%	D:1.1%	F:0.0%	M:0.4%	n:264
GCA_016990195.1_ASM1699019v1_genomic	C:99.6%	S:98.5%	D:1.1%	F:0.0%	M:0.4%	n:264
GCA_016990235.1_ASM1699023v1_genomic	C:99.6%	S:98.5%	D:1.1%	F:0.0%	M:0.4%	n:264
GCA_016990255.1_ASM1699025v1_genomic	C:99.6%	S:98.5%	D:1.1%	F:0.0%	M:0.4%	n:264
GCA_016990275.1_ASM1699027v1_genomic	C:99.6%	S:98.5%	D:1.1%	F:0.0%	M:0.4%	n:264
GCA_016990285.1_ASM1699028v1_genomic	C:99.6%	S:98.5%	D:1.1%	F:0.0%	M:0.4%	n:264
GCA_016990315.1_ASM1699031v1_genomic	C:99.6%	S:98.5%	D:1.1%	F:0.0%	M:0.4%	n:264
GCA_016990335.1_ASM1699033v1_genomic	C:99.6%	S:98.5%	D:1.1%	F:0.0%	M:0.4%	n:264
GCA_016990345.1_ASM1699034v1_genomic	C:99.6%	S:98.5%	D:1.1%	F:0.0%	M:0.4%	n:264
GCA_016990375.1_ASM1699037v1_genomic	C:99.6%	S:98.5%	D:1.1%	F:0.0%	M:0.4%	n:264
GCA_016990395.1_ASM1699039v1_genomic	C:99.6%	S:98.5%	D:1.1%	F:0.0%	M:0.4%	n:264
GCA_016990415.1_ASM1699041v1_genomic	C:99.6%	S:98.5%	D:1.1%	F:0.0%	M:0.4%	n:264
GCA_017330665.1_ASM1733066v1_genomic	C:94.0%	S:93.2%	D:0.8%	F:4.5%	M:1.5%	n:264

GCA_017330685.1_ASM1733068v1_genomic	C:93.2%	S:92.8%	D:0.4%	F:4.5%	M:2.3%	n:264
GCA_017330695.1_ASM1733069v1_genomic	C:92.8%	S:92.0%	D:0.8%	F:6.8%	M:0.4%	n:264
GCA_017330715.1_ASM1733071v1_genomic	C:94.4%	S:93.6%	D:0.8%	F:4.5%	M:1.1%	n:264
GCA_017330725.1_ASM1733072v1_genomic	C:82.2%	S:81.8%	D:0.4%	F:12.9%	M:4.9%	n:264
GCA_017330765.1_ASM1733076v1_genomic	C:83.8%	S:81.1%	D:2.7%	F:12.1%	M:4.1%	n:264
GCA_017330785.1_ASM1733078v1_genomic	C:95.9%	S:95.1%	D:0.8%	F:2.7%	M:1.4%	n:264
GCA_017330795.1_ASM1733079v1_genomic	C:93.2%	S:92.4%	D:0.8%	F:6.4%	M:0.4%	n:264
GCA_017330815.1_ASM1733081v1_genomic	C:56.4%	S:56.4%	D:0.0%	F:32.2%	M:11.4 %	n:264
GCA_017330845.1_ASM1733084v1_genomic	C:56.1%	S:55.7%	D:0.4%	F:34.5%	M:9.4%	n:264
GCA_017330865.1_ASM1733086v1_genomic	C:97.0%	S:96.2%	D:0.8%	F:1.9%	M:1.1%	n:264
GCA_017330885.1_ASM1733088v1_genomic	C:86.8%	S:86.4%	D:0.4%	F:11.0%	M:2.2%	n:264
GCA_017330905.1_ASM1733090v1_genomic	C:57.2%	S:56.4%	D:0.8%	F:31.1%	M:11.7 %	n:264
GCA_017330925.1_ASM1733092v1_genomic	C:47.7%	S:47.3%	D:0.4%	F:38.3%	M:14.0 %	n:264
GCA_017330945.1_ASM1733094v1_genomic	C:95.9%	S:95.1%	D:0.8%	F:2.7%	M:1.4%	n:264
GCA_017330965.1_ASM1733096v1_genomic	C:92.1%	S:91.3%	D:0.8%	F:6.8%	M:1.1%	n:264
GCA_017330985.1_ASM1733098v1_genomic	C:91.3%	S:90.5%	D:0.8%	F:6.1%	M:2.6%	n:264
GCA_017331005.1_ASM1733100v1_genomic	C:93.9%	S:92.8%	D:1.1%	F:4.9%	M:1.2%	n:264
GCA_017331025.1_ASM1733102v1_genomic	C:93.2%	S:93.2%	D:0.0%	F:4.9%	M:1.9%	n:264
GCA_017331045.1_ASM1733104v1_genomic	C:95.1%	S:94.7%	D:0.4%	F:4.2%	M:0.7%	n:264
GCA_017590175.1_ASM1759017v1_genomic	C:96.3%	S:95.5%	D:0.8%	F:0.0%	M:3.7%	n:264
GCA_017591115.1_ASM1759111v1_genomic	C:98.9%	S:98.1%	D:0.8%	F:0.4%	M:0.7%	n:264
GCA_017773255.1_PDT000985504.1_genomic	C:99.2%	S:98.1%	D:1.1%	F:0.4%	M:0.4%	n:264
GCA_902386235.1_UHGG_MGYG-HGUT-02355_geno	C:99.6%	S:98.5%	D:1.1%	F:0.0%	M:0.4%	n:264

mic

SRR8527653.fasta	C:99.6%	S:98.5%	D:1.1%	F:0.0%	M:0.4%	n:264
SRR8527655.fasta	C:99.6%	S:98.5%	D:1.1%	F:0.0%	M:0.4%	n:264
SRR8527656.fasta	C:99.6%	S:98.5%	D:1.1%	F:0.0%	M:0.4%	n:264
SRR8527709.fasta	C:99.7%	S:98.9%	D:0.8%	F:0.0%	M:0.3%	n:264
SRR8527710.fasta	C:99.7%	S:98.9%	D:0.8%	F:0.0%	M:0.3%	n:264
SRR8527718.fasta	C:99.7%	S:98.9%	D:0.8%	F:0.0%	M:0.3%	n:264
SRR8527719.fasta	C:99.7%	S:98.9%	D:0.8%	F:0.4%	M:0.1%	n:264
SRR8527720.fasta	C:99.3%	S:98.5%	D:0.8%	F:0.8%	M:0.1%	n:264
SRR8527761.fasta	C:99.2%	S:98.1%	D:1.1%	F:0.4%	M:0.4%	n:264
SRR8527762.fasta	C:99.6%	S:98.5%	D:1.1%	F:0.0%	M:0.4%	n:264
SRR8527763.fasta	C:99.6%	S:98.5%	D:1.1%	F:0.0%	M:0.4%	n:264
SRR8527764.fasta	C:99.6%	S:98.5%	D:1.1%	F:0.0%	M:0.4%	n:264
SRR8527766.fasta	C:99.6%	S:98.5%	D:1.1%	F:0.0%	M:0.4%	n:264
SRR8980982.fasta	C:99.2%	S:98.1%	D:1.1%	F:0.4%	M:0.4%	n:264
SRR8980983.fasta	C:99.6%	S:98.5%	D:1.1%	F:0.0%	M:0.4%	n:264
SRR8980987.fasta	C:99.6%	S:98.5%	D:1.1%	F:0.0%	M:0.4%	n:264
SRR8981000.fasta	C:99.6%	S:98.5%	D:1.1%	F:0.0%	M:0.4%	n:264
SRR8981002.fasta	C:99.7%	S:98.9%	D:0.8%	F:0.0%	M:0.3%	n:264
SRR8981005.fasta	C:99.6%	S:98.5%	D:1.1%	F:0.0%	M:0.4%	n:264
SRR8981278.fasta	C:99.6%	S:98.5%	D:1.1%	F:0.0%	M:0.4%	n:264
SRR8981312.fasta	C:99.6%	S:98.5%	D:1.1%	F:0.0%	M:0.4%	n:264
SRR8981317.fasta	C:99.7%	S:98.9%	D:0.8%	F:0.0%	M:0.3%	n:264
SRR8981412.fasta	C:99.6%	S:98.5%	D:1.1%	F:0.0%	M:0.4%	n:264
SRR8981424.fasta	C:99.7%	S:98.9%	D:0.8%	F:0.0%	M:0.3%	n:264
SRR8981479.fasta	C:99.7%	S:98.9%	D:0.8%	F:0.0%	M:0.3%	n:264

SRR8981518.fasta	C:98.8%	S:97.3%	D:1.5%	F:0.8%	M:0.4%	n:264
SRR8981519.fasta	C:99.2%	S:97.7%	D:1.5%	F:0.4%	M:0.4%	n:264
SRR8981561.fasta	C:99.3%	S:79.2%	D:20.1%	F:0.4%	M:0.3%	n:264
SRR8981570.fasta	C:99.7%	S:98.9%	D:0.8%	F:0.0%	M:0.3%	n:264
SRR8981574.fasta	C:99.3%	S:98.5%	D:0.8%	F:0.0%	M:0.7%	n:264
SRR8981582.fasta	C:99.7%	S:98.9%	D:0.8%	F:0.0%	M:0.3%	n:264
SRR8981584.fasta	C:99.7%	S:98.9%	D:0.8%	F:0.0%	M:0.3%	n:264
SRR8981586.fasta	C:99.7%	S:98.9%	D:0.8%	F:0.0%	M:0.3%	n:264
SRR8981599.fasta	C:99.2%	S:97.7%	D:1.5%	F:0.4%	M:0.4%	n:264
SRR8981610.fasta	C:99.7%	S:98.9%	D:0.8%	F:0.0%	M:0.3%	n:264
SRR8981625.fasta	C:99.7%	S:98.9%	D:0.8%	F:0.0%	M:0.3%	n:264
SRR8981642.fasta	C:99.2%	S:98.1%	D:1.1%	F:0.4%	M:0.4%	n:264
SRR8981648.fasta	C:99.7%	S:98.9%	D:0.8%	F:0.0%	M:0.3%	n:264
SRR8981653.fasta	C:99.6%	S:98.5%	D:1.1%	F:0.0%	M:0.4%	n:264
SRR8981656.fasta	C:99.6%	S:98.5%	D:1.1%	F:0.0%	M:0.4%	n:264

Table S3 Genome features of isolates

Isolates	Source	Genome Size	GC content	ORF	rRNA	tRNA
XJFE01	FEACE	3848292	0.2811	3590	14	81
XJFE02	FAECE	3864134	0.2804	3628	0	82
XJFE03	FAECE	3859513	0.28	3622	0	80
XJFE04	FAECE	3858165	0.2799	3621	0	81
XJFE05	FAECE	3852112	0.2797	3618	14	80
XJFE06	FAECE	3851616	0.2797	3616	14	80
XJFD01	Environment	3843543	0.2809	3589	15	89
XJFD02	Environment	3837553	0.2806	3586	15	83
XJFD03	Environment	3844630	0.2811	3585	14	82
XJFD04	Environment	3842558	0.281	3586	14	79
XJFD05	Environment	3838417	0.2807	3584	13	80
XJFD06	Environment	3838211	0.2806	3589	16	81
XJFD07	Environment	3838577	0.2806	3582	13	81
XJFD08	Environment	3841234	0.2806	3583	15	81
XJFD09	Environment	3839472	0.2808	3581	17	85
XJFD10	Environment	3844228	0.281	3587	15	89
XJFD11	Environment	3839088	0.2808	3582	14	81
XJFD12	Environment	3839570	0.2807	3584	14	81
XJFD13	Environment	3843175	0.281	3583	13	81
XJFD14	Environment	3833971	0.2804	3583	14	81
XJFD15	Environment	3836596	0.2806	3585	15	81
XJFD16	Environment	3845585	0.2808	3589	15	81
XJFD17	Environment	3827318	0.281	3576	15	82
XJFD18	Environment	3839091	0.2805	3588	14	81

XJFD19	Environment	3834483	0.2804	3583	14	81
XJFD20	Environment	3822747	0.2809	3573	16	82
XJFD21	Environment	3838930	0.2807	3583	16	81
XJFD22	Environment	3837908	0.2806	3584	15	81
XJFD23	Environment	3837522	0.2805	3619	13	82
XJFD24	Environment	3848292	0.2804	3583	14	81
XJFD25	Environment	3840321	0.2804	3588	14	81
XJFD26	Environment	3843307	0.2802	3624	0	83
XJFD27	Environment	3862509	0.2802	3585	0	81
XJSL01	Environment	3836190	0.2805	3590	14	81
XJSL02	Environment	3839571	0.2811	3719	20	85
XJSL03	Environment	3900956	0.2805	3586	14	81
XJSL04	Environment	3837631	0.2811	3732	19	84
XJSL05	Environment	3912852	0.2793	4083	19	84
XJSL06	Environment	4159501	0.2807	3583	17	82
XJSL07	Environment	3850151	0.2814	3726	20	88
XJSL08	Environment	3925292	0.2791	3909	14	82
XJSL09	Environment	4060101	0.2808	3743	21	83
XJSL10	Environment	3920850	0.2807	3588	15	86
XJSL11	Environment	3842046	0.2808	3649	15	81
XJSL12	Environment	3930668	0.2803	3894	14	83
XJSL13	Environment	4138742	0.2809	3744	20	83
XJSL14	Environment	3915883	0.2806	3740	20	83
XJSL15	Environment	3914799	0.2806	3613	13	82
XJSL16	Environment	3867499	0.2802	3615	15	81
XJSL17	Environment	3866601	0.2802	4032	19	87

XJSL18	Environment	4144521	0.2802	3618	14	81
XJSL19	Environment	3865443	0.2801	4263	19	103
XJSL20	Environment	4330018	0.2803	3613	14	81
XJSL21	Environment	3862397	0.281	3749	19	83
XJSL22	Environment	3917747	0.2807	3594	13	81
XJSL23	Environment	3853435	0.2806	3587	16	81
XJSL24	Environment	3838472	0.2815	3622	17	85
XJSL25	Environment	3873383	0.2811	3621	14	82
XJSL26	Environment	3861194	0.2808	3619	14	82
GCA_000017025.1_ASM1702v1_genomic	Public Genome	3855885	0.2821	3609	24	81
GCA_000017045.1_ASM1704v1_genomic	Public Genome	3863450	0.2818	3486	24	81
GCA_000017065.1_ASM1706v1_genomic	Public Genome	3760560	0.272	3756	27	81
GCA_000019305.1_ASM1930v1_genomic	Public Genome	4012918	0.2685	3889	27	82
GCA_000019545.1_ASM1954v1_genomic	Public Genome	4107013	0.2697	4021	27	81
GCA_000020345.1_ASM2034v1_genomic	Public Genome	4259691	0.2597	4153	27	81
GCA_000022765.1_ASM2276v1_genomic	Public Genome	4257769	0.2821	3939	27	81
GCA_000063585.1_ASM6358v1_genomic	Public Genome	4155278	0.2752	3654	27	79
GCA_000092345.1_ASM9234v1_genomic	Public Genome	3903260	0.2719	3977	27	71
GCA_000171055.1_ASM17105v1_genomic	Public Genome	4010614	0.3774	3728	56	84
GCA_000171075.1_ASM17107v1_genomic	Public Genome	4031357	0.3087	4060	53	81
GCA_000253195.1_ASM25319v1_genomic	Public Genome	4217754	0.2821	3743	27	72
GCA_000307635.1_ASM30763v1_genomic	Public Genome	3919740	0.281	4416	1	44
GCA_000307655.2_CFSAN0001628_2.0_genomic	Public Genome	4076805	0.2784	4310	3	42
GCA_000439615.1_CFSAN002367_1.0_genomic	Public Genome	4013630	0.2791	5717	2	36
GCA_000439635.1_CFSAN002368_1.0_genomic	Public Genome	3873962	0.2752	5679	2	35
GCA_000439655.1_CFSAN002369_1.0_genomic	Public Genome	3850187	0.2829	5406	2	34

GCA_000439815.2_ASM43981v2_genomic	Public Genome	3645546	0.3158	4011	11	83
GCA_000503815.1_ASM50381v1_genomic	Public Genome	4086083	0.2575	4242	3	49
GCA_000582375.1_CDC54075.1_genomic	Public Genome	4408349	0.2836	4604	16	71
GCA_000582415.1_CDC54088.1_genomic	Public Genome	4256146	0.2795	4115	11	52
GCA_000582435.1_CDC54085.1_genomic	Public Genome	3868107	0.279	4113	13	72
GCA_000582455.1_CDC54091.1_genomic	Public Genome	4095763	0.2768	5648	8	61
GCA_000710975.1_B2_331_genomic	Public Genome	4079046	0.268	3603	4	67
GCA_000710985.1_B2_275_genomic	Public Genome	3809103	0.2699	3825	3	67
GCA_000710995.1_A2B3_87_genomic	Public Genome	3978188	0.2641	3980	2	61
GCA_000711005.1_A2B7_92_genomic	Public Genome	4168550	0.2654	3799	3	72
GCA_000711055.1_A2_117_genomic	Public Genome	4057812	0.2657	3552	2	67
GCA_000711065.1_B2_128_genomic	Public Genome	3808262	0.2669	3617	1	65
GCA_000711095.1_B2_267_genomic	Public Genome	3844467	0.2673	3714	1	76
GCA_000711105.1_F_357_genomic	Public Genome	3903580	0.2679	3631	3	66
GCA_000711115.1_B2_433_genomic	Public Genome	3832122	0.2658	4004	1	61
GCA_000730705.1_ASM73070v1_genomic	Public Genome	4124526	0.2803	3889	19	76
GCA_000730715.1_ASM73071v1_genomic	Public Genome	3901135	0.2787	4089	20	72
GCA_000730725.1_ASM73072v1_genomic	Public Genome	3977994	0.2773	3873	13	75
GCA_000730735.1_ASM73073v1_genomic	Public Genome	3966737	0.2831	3566	13	68
GCA_000730785.1_ASM73078v1_genomic	Public Genome	3684733	0.2827	3853	19	69
GCA_000730795.1_ASM73079v1_genomic	Public Genome	3960455	0.2818	3823	16	77
GCA_000730805.1_ASM73080v1_genomic	Public Genome	3890459	0.2843	3884	16	69
GCA_000730835.1_ASM73083v1_genomic	Public Genome	3868980	0.2926	3711	19	79
GCA_000730865.1_ASM73086v1_genomic	Public Genome	3833726	0.2912	3359	13	62
GCA_000730875.1_ASM73087v1_genomic	Public Genome	3444700	0.2801	3993	19	71
GCA_000730885.1_ASM73088v1_genomic	Public Genome	3965132	0.2838	3553	15	71

GCA_000730925.1_ASM73092v1_genomic	Public Genome	3699887	0.2847	3831	17	70
GCA_000730945.1_ASM73094v1_genomic	Public Genome	3898725	0.2924	3789	18	76
GCA_000730955.1_ASM73095v1_genomic	Public Genome	3557862	0.2911	3808	24	83
GCA_000730965.1_ASM73096v1_genomic	Public Genome	3731781	0.279	3735	11	61
GCA_000769495.1_CFSAN024410_01.0_genomic	Public Genome	3893295	0.2836	3904	6	66
GCA_000816945.1_ASM81694v1_genomic	Public Genome	4005128	0.2803	3727	14	52
GCA_000816965.1_ASM81696v1_genomic	Public Genome	3892029	0.2674	4151	15	75
GCA_000817935.1_ASM81793v1_genomic	Public Genome	4334551	0.2683	4313	24	70
GCA_000829015.1_ASM82901v1_genomic	Public Genome	4365669	0.2827	3610	31	83
GCA_000830755.1_ASM83075v1_genomic	Public Genome	3901300	0.3011	3848	5	65
GCA_000876495.2_ASM87649v2_genomic	Public Genome	3938115	0.2603	4132	3	69
GCA_000965295.1_ASM96529v1_genomic	Public Genome	4320669	0.2731	5661	4	62
GCA_000965325.1_ASM96532v1_genomic	Public Genome	4291470	0.2726	6147	3	57
GCA_000965345.1_ASM96534v1_genomic	Public Genome	3864878	0.2745	5821	5	47
GCA_000965375.1_ASM96537v1_genomic	Public Genome	3810828	0.2795	5367	8	67
GCA_000965385.1_ASM96538v1_genomic	Public Genome	3886945	0.2758	5526	3	56
GCA_000965445.1_ASM96544v1_genomic	Public Genome	3888335	0.2753	5008	4	62
GCA_000965465.1_ASM96546v1_genomic	Public Genome	3792223	0.2714	6777	8	65
GCA_000965505.1_ASM96550v1_genomic	Public Genome	4207455	0.2674	6121	4	52
GCA_000965515.1_ASM96551v1_genomic	Public Genome	4252259	0.2712	5476	4	47
GCA_000986925.1_CBOT001-SEQ-1-ASM-1_genomic	Public Genome	4081847	0.3432	3753	11	78
GCA_001273165.1_CBOT002-SEQ-1-ASM-1_genomic	Public Genome	3978537	0.3372	3686	11	78
GCA_001273255.1_CBOT006-SEQ-1-ASM-1_genomic	Public Genome	3906754	0.3186	3718	10	78
GCA_001273275.1_CBOT007-SEQ-1-ASM-1_genomic	Public Genome	3907623	0.3248	3690	10	77
GCA_001276985.1_CBOT009-SEQ-1-ASM-1_genomic	Public Genome	3909427	0.3248	3676	11	83
GCA_001573175.1_10148_genomic	Public Genome	3889092	0.2819	3748	9	64

GCA_001573235.1_ASM157323v1_genomic	Public Genome	3959495	0.2854	3683	7	48
GCA_001573255.1_ASM157325v1_genomic	Public Genome	3898546	0.2858	4137	5	56
GCA_001573295.1_ASM157329v1_genomic	Public Genome	4201460	0.2815	3643	6	41
GCA_001573315.1_ASM157331v1_genomic	Public Genome	3864510	0.2875	3535	5	52
GCA_001573325.1_ASM157332v1_genomic	Public Genome	3777532	0.2796	3829	5	51
GCA_001573335.1_ASM157333v1_genomic	Public Genome	4002127	0.278	3861	7	57
GCA_001573375.1_ASM157337v1_genomic	Public Genome	4025192	0.2764	3798	6	48
GCA_001573385.1_ASM157338v1_genomic	Public Genome	3971034	0.2889	3682	6	63
GCA_001573395.1_ASM157339v1_genomic	Public Genome	3891395	0.2792	3691	8	57
GCA_001573435.1_ASM157343v1_genomic	Public Genome	3909258	0.2916	3726	6	59
GCA_001573455.1_ASM157345v1_genomic	Public Genome	3942683	0.2908	3676	5	59
GCA_001573465.1_ASM157346v1_genomic	Public Genome	3880168	0.2929	3555	5	58
GCA_001573485.1_ASM157348v1_genomic	Public Genome	3791338	0.2854	3587	6	52
GCA_001573515.1_ASM157351v1_genomic	Public Genome	3812410	0.2926	3682	8	63
GCA_001573535.1_ASM157353v1_genomic	Public Genome	3856611	0.2891	3712	7	66
GCA_001573555.1_ASM157355v1_genomic	Public Genome	3896775	0.2839	3721	8	46
GCA_001573665.1_ASM157366v1_genomic	Public Genome	3920281	0.2872	3779	7	55
GCA_001573725.1_ASM157372v1_genomic	Public Genome	4008651	0.3022	3719	10	69
GCA_001573825.1_ASM157382v1_genomic	Public Genome	3900045	0.2891	3597	6	42
GCA_001573885.1_ASM157388v1_genomic	Public Genome	3869437	0.2935	4229	7	73
GCA_001573905.1_ASM157390v1_genomic	Public Genome	4285737	0.2794	4171	8	56
GCA_001573935.1_ASM157393v1_genomic	Public Genome	4267307	0.2951	3567	8	75
GCA_001573955.1_ASM157395v1_genomic	Public Genome	3873577	0.2871	4105	9	69
GCA_001573965.1_ASM157396v1_genomic	Public Genome	4230750	0.2913	4198	9	62
GCA_001573985.1_ASM157398v1_genomic	Public Genome	4274979	0.2878	3790	7	71
GCA_001574015.1_ASM157401v1_genomic	Public Genome	4010957	0.2822	4096	8	69

GCA_001574035.1_ASM157403v1_genomic	Public Genome	4305633	0.2975	3774	7	69
GCA_001574055.1_ASM157405v1_genomic	Public Genome	3990632	0.2858	4212	8	71
GCA_001574065.1_ASM157406v1_genomic	Public Genome	4306760	0.2726	4016	10	72
GCA_001574095.1_ASM157409v1_genomic	Public Genome	4175404	0.2765	4426	9	71
GCA_001574115.1_ASM157411v1_genomic	Public Genome	4476519	0.2868	4218	8	73
GCA_001574125.1_ASM157412v1_genomic	Public Genome	4311895	0.2871	4196	7	65
GCA_001574135.1_ASM157413v1_genomic	Public Genome	4281988	0.2873	4267	8	73
GCA_001574175.1_ASM157417v1_genomic	Public Genome	4368910	0.2774	4429	8	70
GCA_001574195.1_ASM157419v1_genomic	Public Genome	4495542	0.2859	3853	9	69
GCA_001574205.1_ASM157420v1_genomic	Public Genome	4055427	0.2918	3689	8	69
GCA_001574225.1_ASM157422v1_genomic	Public Genome	3929155	0.2974	4466	7	69
GCA_001574255.1_ASM157425v1_genomic	Public Genome	4534970	0.2835	4083	9	70
GCA_001574265.1_ASM157426v1_genomic	Public Genome	4210622	0.2844	3916	9	67
GCA_001574285.1_ASM157428v1_genomic	Public Genome	4091988	0.2911	4013	9	70
GCA_001574315.1_ASM157431v1_genomic	Public Genome	4136384	0.2877	4209	9	70
GCA_001574335.1_ASM157433v1_genomic	Public Genome	4296977	0.2945	3685	7	70
GCA_001574355.1_ASM157435v1_genomic	Public Genome	3882852	0.2854	3736	9	47
GCA_001574365.1_ASM157436v1_genomic	Public Genome	3929760	0.2822	3789	5	56
GCA_001879605.1_ASM187960v1_genomic	Public Genome	3945914	0.2814	3814	27	81
GCA_001879625.1_ASM187962v1_genomic	Public Genome	4089027	0.2753	3816	27	82
GCA_001889345.1_ASM188934v1_genomic	Public Genome	4020063	0.2824	3538	27	81
GCA_001889365.1_ASM188936v1_genomic	Public Genome	3867627	0.2667	4332	27	85
GCA_001921905.1_ASM192190v1_genomic	Public Genome	4432564	0.2734	3717	27	83
GCA_001921925.1_ASM192192v1_genomic	Public Genome	3967717	0.2667	4316	27	83
GCA_001921945.1_ASM192194v1_genomic	Public Genome	4414346	0.2687	3971	27	81
GCA_001921965.1_ASM192196v1_genomic	Public Genome	4194523	0.2713	3752	27	82

GCA_001921985.1_ASM192198v1_genomic	Public Genome	4011842	0.2569	4220	27	82
GCA_001951135.2_ASM195113v2_genomic	Public Genome	4303351	0.2532	4300	25	81
GCA_002024365.1_ASM202436v1_genomic	Public Genome	4252578	0.2815	3790	2	41
GCA_002024375.1_ASM202437v1_genomic	Public Genome	4000805	0.2818	3798	2	46
GCA_002024385.1_ASM202438v1_genomic	Public Genome	4005673	0.2911	3703	5	62
GCA_002024395.1_ASM202439v1_genomic	Public Genome	3897060	0.2832	3648	2	41
GCA_002024445.1_ASM202444v1_genomic	Public Genome	3875827	0.3005	3566	11	73
GCA_002024465.1_ASM202446v1_genomic	Public Genome	3798317	0.3274	3924	2	43
GCA_002103795.1_ASM210379v1_genomic	Public Genome	4023802	0.3084	4041	11	78
GCA_002103805.1_ASM210380v1_genomic	Public Genome	4230844	0.3292	3911	11	80
GCA_002103825.1_ASM210382v1_genomic	Public Genome	4043191	0.3331	3958	9	82
GCA_002103855.1_ASM210385v1_genomic	Public Genome	4079517	0.2968	3737	10	62
GCA_002103875.1_ASM210387v1_genomic	Public Genome	3972674	0.3507	3654	8	79
GCA_002103885.1_ASM210388v1_genomic	Public Genome	3906590	0.3134	3991	10	78
GCA_002103975.1_ASM210397v1_genomic	Public Genome	4113388	0.2879	3710	5	39
GCA_002103985.1_ASM210398v1_genomic	Public Genome	3888462	0.2965	3745	4	64
GCA_002104015.1_ASM210401v1_genomic	Public Genome	3941623	0.2829	3764	10	60
GCA_002260585.1_C.botulinum_2.0_genomic	Public Genome	3963867	0.2807	3264	1	27
GCA_002865745.1_ASM286574v1_genomic	Public Genome	2904173	0.2824	3703	27	81
GCA_002865765.1_ASM286576v1_genomic	Public Genome	3945134	0.2826	3601	27	81
GCA_002865805.1_ASM286580v1_genomic	Public Genome	3917597	0.2726	3664	24	80
GCA_002865825.1_ASM286582v1_genomic	Public Genome	3914565	0.2648	4016	27	82
GCA_002865845.1_ASM286584v1_genomic	Public Genome	4154949	0.281	3958	27	82
GCA_002865885.1_ASM286588v1_genomic	Public Genome	4159640	0.2668	3936	27	85
GCA_002866045.1_ASM286604v1_genomic	Public Genome	4084533	0.2694	4023	27	81
GCA_002866125.1_ASM286612v1_genomic	Public Genome	4169506	0.2729	3868	27	84

GCA_002866225.1_ASM286622v1_genomic	Public Genome	4050477	0.2748	3972	27	85
GCA_003014955.1_ASM301495v1_genomic	Public Genome	4109153	0.2771	3684	5	66
GCA_003017145.1_ASM301714v1_genomic	Public Genome	3877613	0.2785	3695	8	61
GCA_003017195.1_ASM301719v1_genomic	Public Genome	3988693	0.2792	3705	10	62
GCA_003017225.1_ASM301722v1_genomic	Public Genome	4004635	0.2779	3717	10	55
GCA_003017335.1_ASM301733v1_genomic	Public Genome	3998747	0.2808	3683	27	70
GCA_003058345.1_ASM305834v1_genomic	Public Genome	3978960	0.2822	3569	27	82
GCA_003058445.1_ASM305844v1_genomic	Public Genome	3858511	0.2732	3702	27	82
GCA_003345315.1_ASM334531v1_genomic	Public Genome	3930410	0.2756	3807	27	84
GCA_003345335.1_ASM334533v1_genomic	Public Genome	4018503	0.2597	4057	30	84
GCA_003412385.1_ASM341238v1_genomic	Public Genome	4202171	0.2798	6654	10	56
GCA_003412395.1_ASM341239v1_genomic	Public Genome	4149224	0.2717	6397	7	52
GCA_003412435.1_ASM341243v1_genomic	Public Genome	4050896	0.2869	6797	8	63
GCA_003515665.1_ASM351566v1_genomic	Public Genome	4196137	0.2798	4521	18	79
GCA_003610755.1_ASM361075v1_genomic	Public Genome	4162303	0.3739	3582	12	83
GCA_003994935.1_ASM399493v1_genomic	Public Genome	3830015	0.3083	3620	23	82
GCA_003996845.1_ASM399684v1_genomic	Public Genome	3896768	0.2794	5491	7	74
GCA_003996885.1_ASM399688v1_genomic	Public Genome	4035670	0.3335	5108	8	68
GCA_003996895.1_ASM399689v1_genomic	Public Genome	4024675	0.305	6626	9	79
GCA_003996925.1_ASM399692v1_genomic	Public Genome	4019721	0.2808	7797	8	51
GCA_003996935.1_ASM399693v1_genomic	Public Genome	4050546	0.2957	4928	7	73
GCA_004120535.1_ASM412053v1_genomic	Public Genome	3905902	0.3112	3786	11	77
GCA_009733885.1_ASM973388v1_genomic	Public Genome	4015174	0.2752	3663	27	77
GCA_009938935.1_ASM993893v1_genomic	Public Genome	3903111	0.2894	4322	10	78
GCA_009939325.1_ASM993932v1_genomic	Public Genome	4320953	0.289	4311	10	78
GCA_009939345.1_ASM993934v1_genomic	Public Genome	4313565	0.2978	4324	10	78

GCA_010078315.1_ASM1007831v1_genomic	Public Genome	4323822	0.2965	4325	10	78
GCA_011009485.1_ASM1100948v1_genomic	Public Genome	4323559	0.309	3748	10	77
GCA_011009795.1_ASM1100979v1_genomic	Public Genome	3973868	0.3123	3674	10	78
GCA_011009805.1_ASM1100980v1_genomic	Public Genome	3867730	0.3169	3659	10	80
GCA_011009855.1_ASM1100985v1_genomic	Public Genome	3861023	0.3144	3899	10	79
GCA_011009875.1_ASM1100987v1_genomic	Public Genome	4027438	0.2915	3561	10	85
GCA_011009885.1_ASM1100988v1_genomic	Public Genome	3798868	0.3011	3261	11	83
GCA_011009915.1_ASM1100991v1_genomic	Public Genome	3473206	0.3323	3598	9	76
GCA_011009925.1_ASM1100992v1_genomic	Public Genome	3880866	0.3395	4181	12	82
GCA_011009955.1_ASM1100995v1_genomic	Public Genome	4274843	0.2975	3554	11	79
GCA_011009975.1_ASM1100997v1_genomic	Public Genome	3798113	0.335	3690	11	79
GCA_011009985.1_ASM1100998v1_genomic	Public Genome	3891053	0.2939	3599	11	79
GCA_011010005.1_ASM1101000v1_genomic	Public Genome	3849420	0.288	3753	10	74
GCA_011010035.1_ASM1101003v1_genomic	Public Genome	3980847	0.3012	3658	10	79
GCA_011010055.1_ASM1101005v1_genomic	Public Genome	3856330	0.2934	3652	10	80
GCA_011010065.1_ASM1101006v1_genomic	Public Genome	3857002	0.3148	3661	11	80
GCA_011010085.1_ASM1101008v1_genomic	Public Genome	3860944	0.3085	3665	10	80
GCA_011010105.1_ASM1101010v1_genomic	Public Genome	3862813	0.2932	3651	10	80
GCA_011010135.1_ASM1101013v1_genomic	Public Genome	3856483	0.3224	3675	10	78
GCA_011010155.1_ASM1101015v1_genomic	Public Genome	3867578	0.2819	3620	10	79
GCA_011010165.1_ASM1101016v1_genomic	Public Genome	3828753	0.3047	3657	10	80
GCA_011010175.1_ASM1101017v1_genomic	Public Genome	3859200	0.3054	3660	11	80
GCA_011010205.1_ASM1101020v1_genomic	Public Genome	3859559	0.3031	3655	10	79
GCA_011010235.1_ASM1101023v1_genomic	Public Genome	3859700	0.2906	4103	10	79
GCA_011010245.1_ASM1101024v1_genomic	Public Genome	4176930	0.2902	3652	10	79
GCA_011010275.1_ASM1101027v1_genomic	Public Genome	3857022	0.2973	4026	11	80

GCA_011010285.1_ASM1101028v1_genomic	Public Genome	4134956	0.3214	3669	10	78
GCA_011010295.1_ASM1101029v1_genomic	Public Genome	3866454	0.3084	3668	10	78
GCA_011010335.1_ASM1101033v1_genomic	Public Genome	3866446	0.289	4004	10	79
GCA_011010345.1_ASM1101034v1_genomic	Public Genome	4115608	0.2899	3673	10	78
GCA_011010355.1_ASM1101035v1_genomic	Public Genome	3908835	0.3108	3686	10	78
GCA_011010375.1_ASM1101037v1_genomic	Public Genome	3912899	0.3114	3681	10	78
GCA_011010415.1_ASM1101041v1_genomic	Public Genome	3909273	0.2932	3807	14	80
GCA_011010435.1_ASM1101043v1_genomic	Public Genome	3996158	0.2964	3781	10	81
GCA_011010445.1_ASM1101044v1_genomic	Public Genome	3967861	0.2983	3800	14	78
GCA_011010455.1_ASM1101045v1_genomic	Public Genome	3985895	0.3004	3797	10	79
GCA_011010485.1_ASM1101048v1_genomic	Public Genome	3985038	0.3123	3740	12	85
GCA_011010505.1_ASM1101050v1_genomic	Public Genome	3951200	0.3016	3733	12	85
GCA_011010535.1_ASM1101053v1_genomic	Public Genome	3949710	0.3053	3739	12	85
GCA_011010545.1_ASM1101054v1_genomic	Public Genome	3951549	0.3053	3736	12	85
GCA_011010575.1_ASM1101057v1_genomic	Public Genome	3950691	0.2998	3709	14	89
GCA_011010615.1_ASM1101061v1_genomic	Public Genome	3929568	0.2897	3721	12	85
GCA_011010635.1_ASM1101063v1_genomic	Public Genome	3944562	0.2971	4056	12	78
GCA_011010645.1_ASM1101064v1_genomic	Public Genome	4168159	0.2961	4213	12	79
GCA_011010655.1_ASM1101065v1_genomic	Public Genome	4295290	0.3119	4012	12	78
GCA_011010685.1_ASM1101068v1_genomic	Public Genome	4141644	0.2963	3598	11	78
GCA_011010715.1_ASM1101071v1_genomic	Public Genome	3719910	0.3066	4056	12	75
GCA_011010735.1_ASM1101073v1_genomic	Public Genome	4167027	0.312	4068	11	77
GCA_011010745.1_ASM1101074v1_genomic	Public Genome	4168441	0.2874	3751	12	78
GCA_011010775.1_ASM1101077v1_genomic	Public Genome	3948662	0.3003	3557	11	79
GCA_011010785.1_ASM1101078v1_genomic	Public Genome	3798028	0.3126	3758	12	79
GCA_011010835.1_ASM1101083v1_genomic	Public Genome	3952848	0.286	4084	11	77

GCA_011010865.1_ASM1101086v1_genomic	Public Genome	4223375	0.3141	4090	12	78
GCA_011010895.1_ASM1101089v1_genomic	Public Genome	4228906	0.2955	4082	13	79
GCA_011010905.1_ASM1101090v1_genomic	Public Genome	4228724	0.3189	4092	12	78
GCA_011010935.1_ASM1101093v1_genomic	Public Genome	4228449	0.2929	3488	10	78
GCA_011010945.1_ASM1101094v1_genomic	Public Genome	3765488	0.2996	3558	11	79
GCA_011010965.1_ASM1101096v1_genomic	Public Genome	3795956	0.3085	3907	12	79
GCA_011010975.1_ASM1101097v1_genomic	Public Genome	4090957	0.3105	3907	12	78
GCA_011011015.1_ASM1101101v1_genomic	Public Genome	4091619	0.3127	3910	13	79
GCA_011011035.1_ASM1101103v1_genomic	Public Genome	4091150	0.3088	3907	13	80
GCA_011011045.1_ASM1101104v1_genomic	Public Genome	4091843	0.3121	3912	12	78
GCA_011011075.1_ASM1101107v1_genomic	Public Genome	4091492	0.2926	3885	12	78
GCA_011011095.1_ASM1101109v1_genomic	Public Genome	4078951	0.3063	3909	12	78
GCA_011011105.1_ASM1101110v1_genomic	Public Genome	4088873	0.3011	3909	12	78
GCA_011011135.1_ASM1101113v1_genomic	Public Genome	4089245	0.3035	3904	12	78
GCA_011011145.1_ASM1101114v1_genomic	Public Genome	4089887	0.2906	3899	12	78
GCA_011011165.1_ASM1101116v1_genomic	Public Genome	4080372	0.2978	3903	11	78
GCA_011011185.1_ASM1101118v1_genomic	Public Genome	4086921	0.3006	3901	12	79
GCA_011011215.1_ASM1101121v1_genomic	Public Genome	4087594	0.2962	3898	11	78
GCA_011011235.1_ASM1101123v1_genomic	Public Genome	4088527	0.2906	3748	9	76
GCA_011011245.1_ASM1101124v1_genomic	Public Genome	3931029	0.2897	3754	9	76
GCA_011011255.1_ASM1101125v1_genomic	Public Genome	3933063	0.3251	3912	12	80
GCA_011011295.1_ASM1101129v1_genomic	Public Genome	4090337	0.2979	3776	10	80
GCA_011011315.1_ASM1101131v1_genomic	Public Genome	3975101	0.3037	3781	10	75
GCA_011011335.1_ASM1101133v1_genomic	Public Genome	3978619	0.3142	3779	10	74
GCA_011011345.1_ASM1101134v1_genomic	Public Genome	3978450	0.3037	3701	10	79
GCA_011011375.1_ASM1101137v1_genomic	Public Genome	3916426	0.2906	3830	11	77

GCA_011011385.1_ASM1101138v1_genomic	Public Genome	3998935	0.3016	3750	9	76
GCA_011011415.1_ASM1101141v1_genomic	Public Genome	3934183	0.3228	3833	10	78
GCA_011011435.1_ASM1101143v1_genomic	Public Genome	4003589	0.2948	3817	11	77
GCA_011011445.1_ASM1101144v1_genomic	Public Genome	3999383	0.2871	3906	11	78
GCA_011011465.1_ASM1101146v1_genomic	Public Genome	4087213	0.3018	3936	12	80
GCA_011011495.1_ASM1101149v1_genomic	Public Genome	4111607	0.2891	3555	11	79
GCA_011011515.1_ASM1101151v1_genomic	Public Genome	3793988	0.3014	3561	11	75
GCA_011011535.1_ASM1101153v1_genomic	Public Genome	3798774	0.2875	3037	9	76
GCA_011011545.1_ASM1101154v1_genomic	Public Genome	3155674	0.3016	3752	11	78
GCA_011011575.1_ASM1101157v1_genomic	Public Genome	3958357	0.2897	3554	10	77
GCA_011011585.1_ASM1101158v1_genomic	Public Genome	3810990	0.3079	3666	11	79
GCA_011011615.1_ASM1101161v1_genomic	Public Genome	3871259	0.3022	3757	9	76
GCA_011011635.1_ASM1101163v1_genomic	Public Genome	3934160	0.3443	3612	11	81
GCA_011011645.1_ASM1101164v1_genomic	Public Genome	3852127	0.2916	3631	10	78
GCA_011011665.1_ASM1101166v1_genomic	Public Genome	3826272	0.3003	3557	11	79
GCA_011011695.1_ASM1101169v1_genomic	Public Genome	3795683	0.3195	3558	11	79
GCA_011011715.1_ASM1101171v1_genomic	Public Genome	3799096	0.3016	3635	11	79
GCA_011011725.1_ASM1101172v1_genomic	Public Genome	3876634	0.3085	3636	11	79
GCA_011011735.1_ASM1101173v1_genomic	Public Genome	3876531	0.2859	3568	11	79
GCA_011011755.1_ASM1101175v1_genomic	Public Genome	3805074	0.2977	3541	11	79
GCA_011011765.1_ASM1101176v1_genomic	Public Genome	3778049	0.2891	3552	11	79
GCA_011011815.1_ASM1101181v1_genomic	Public Genome	3794376	0.2888	3483	11	77
GCA_011011835.1_ASM1101183v1_genomic	Public Genome	3767669	0.3082	3637	11	79
GCA_011011845.1_ASM1101184v1_genomic	Public Genome	3868071	0.2871	3596	11	80
GCA_011011865.1_ASM1101186v1_genomic	Public Genome	3810758	0.3241	3561	11	79
GCA_011011885.1_ASM1101188v1_genomic	Public Genome	3800178	0.2902	3556	11	79

GCA_011011895.1_ASM1101189v1_genomic	Public Genome	3796818	0.3033	3555	11	79
GCA_011011925.1_ASM1101192v1_genomic	Public Genome	3796672	0.3053	3562	11	79
GCA_011011955.1_ASM1101195v1_genomic	Public Genome	3799286	0.2929	3554	11	79
GCA_011011965.1_ASM1101196v1_genomic	Public Genome	3791805	0.3067	3672	10	78
GCA_011011995.1_ASM1101199v1_genomic	Public Genome	3865412	0.303	3557	11	79
GCA_011012005.1_ASM1101200v1_genomic	Public Genome	3799106	0.2957	4256	11	80
GCA_011012015.1_ASM1101201v1_genomic	Public Genome	4337561	0.3007	3645	11	79
GCA_011012055.1_ASM1101205v1_genomic	Public Genome	3867484	0.3142	3799	11	79
GCA_011012075.1_ASM1101207v1_genomic	Public Genome	3981193	0.3096	4051	12	78
GCA_011012095.1_ASM1101209v1_genomic	Public Genome	4168420	0.3126	4060	13	79
GCA_011012105.1_ASM1101210v1_genomic	Public Genome	4170318	0.2958	3776	10	78
GCA_011012125.1_ASM1101212v1_genomic	Public Genome	3972945	0.3007	3911	12	78
GCA_011012155.1_ASM1101215v1_genomic	Public Genome	4092235	0.2854	3819	9	76
GCA_011012165.1_ASM1101216v1_genomic	Public Genome	3997069	0.3136	3830	11	77
GCA_011012195.1_ASM1101219v1_genomic	Public Genome	4003701	0.2869	3765	11	81
GCA_011012215.1_ASM1101221v1_genomic	Public Genome	3949924	0.3078	3559	11	79
GCA_011012225.1_ASM1101222v1_genomic	Public Genome	3799360	0.2884	3548	11	79
GCA_011012355.1_ASM1101235v1_genomic	Public Genome	3789818	0.3391	3670	24	118
GCA_011012365.1_ASM1101236v1_genomic	Public Genome	3998487	0.3051	3599	9	75
GCA_011012375.1_ASM1101237v1_genomic	Public Genome	3908841	0.3388	3589	10	78
GCA_011012435.1_ASM1101243v1_genomic	Public Genome	3905010	0.3305	3701	7	67
GCA_011012455.1_ASM1101245v1_genomic	Public Genome	3895678	0.3077	3617	10	77
GCA_011012465.1_ASM1101246v1_genomic	Public Genome	3850217	0.3411	3772	9	75
GCA_011012695.1_ASM1101269v1_genomic	Public Genome	4000037	0.2868	3843	8	79
GCA_011012725.1_ASM1101272v1_genomic	Public Genome	4010699	0.3081	3888	8	79
GCA_011012735.1_ASM1101273v1_genomic	Public Genome	4062902	0.3015	3540	10	79

GCA_011012755.1_ASM1101275v1_genomic	Public Genome	3851997	0.2922	3559	9	78
GCA_011012795.1_ASM1101279v1_genomic	Public Genome	3861614	0.3094	3579	10	77
GCA_011012815.1_ASM1101281v1_genomic	Public Genome	3894468	0.3912	3582	13	93
GCA_011012825.1_ASM1101282v1_genomic	Public Genome	3910017	0.3229	3569	10	77
GCA_011012835.1_ASM1101283v1_genomic	Public Genome	3871748	0.3212	3882	8	78
GCA_011012875.1_ASM1101287v1_genomic	Public Genome	4068260	0.3277	4028	9	79
GCA_011012885.1_ASM1101288v1_genomic	Public Genome	4183819	0.3172	3556	10	81
GCA_011013055.1_ASM1101305v1_genomic	Public Genome	3873600	0.3171	3778	12	76
GCA_011013175.1_ASM1101317v1_genomic	Public Genome	4005020	0.3108	3370	11	81
GCA_011013235.1_ASM1101323v1_genomic	Public Genome	3581508	0.3301	3770	14	77
GCA_011013255.1_ASM1101325v1_genomic	Public Genome	4001618	0.2934	3581	9	76
GCA_011013265.1_ASM1101326v1_genomic	Public Genome	3902144	0.2886	3764	11	77
GCA_011013315.1_ASM1101331v1_genomic	Public Genome	3997729	0.2992	3769	15	82
GCA_011013335.1_ASM1101333v1_genomic	Public Genome	4005196	0.2913	3865	11	75
GCA_011013435.1_ASM1101343v1_genomic	Public Genome	4063501	0.2837	3874	8	75
GCA_011013545.1_ASM1101354v1_genomic	Public Genome	4065017	0.29	3764	12	76
GCA_011013585.1_ASM1101358v1_genomic	Public Genome	3996558	0.2894	3763	12	75
GCA_011013615.1_ASM1101361v1_genomic	Public Genome	4000766	0.3004	3590	9	76
GCA_011013635.1_ASM1101363v1_genomic	Public Genome	3908999	0.2947	3576	9	75
GCA_011013675.1_ASM1101367v1_genomic	Public Genome	3894475	0.3112	3589	9	73
GCA_011013685.1_ASM1101368v1_genomic	Public Genome	3902882	0.2845	3760	9	75
GCA_011013735.1_ASM1101373v1_genomic	Public Genome	3996921	0.3184	3666	11	81
GCA_011013765.1_ASM1101376v1_genomic	Public Genome	3892642	0.2974	3597	9	73
GCA_011013785.1_ASM1101378v1_genomic	Public Genome	3831714	0.2901	3503	10	75
GCA_011013815.1_ASM1101381v1_genomic	Public Genome	3751711	0.3629	3587	10	82
GCA_011013835.1_ASM1101383v1_genomic	Public Genome	3905020	0.3022	3678	8	77

GCA_011013865.1_ASM1101386v1_genomic	Public Genome	3867632	0.3163	3586	10	76
GCA_011013885.1_ASM1101388v1_genomic	Public Genome	3906223	0.2992	3582	9	72
GCA_011013905.1_ASM1101390v1_genomic	Public Genome	3900642	0.3282	3597	9	72
GCA_011013935.1_ASM1101393v1_genomic	Public Genome	3907747	0.3315	4021	9	74
GCA_011013955.1_ASM1101395v1_genomic	Public Genome	4222245	0.288	3824	8	76
GCA_011013965.1_ASM1101396v1_genomic	Public Genome	4006568	0.2957	3569	9	78
GCA_011013975.1_ASM1101397v1_genomic	Public Genome	3886673	0.2823	3647	8	79
GCA_011014015.1_ASM1101401v1_genomic	Public Genome	3881202	0.3207	3705	12	81
GCA_011014035.1_ASM1101403v1_genomic	Public Genome	3928118	0.3026	3648	11	83
GCA_011014055.1_ASM1101405v1_genomic	Public Genome	3894198	0.2905	3583	9	75
GCA_011014085.1_ASM1101408v1_genomic	Public Genome	3897028	0.2894	3569	9	76
GCA_011014095.1_ASM1101409v1_genomic	Public Genome	3891786	0.3	3528	9	77
GCA_011014135.1_ASM1101413v1_genomic	Public Genome	3843117	0.2966	3569	10	76
GCA_011014155.1_ASM1101415v1_genomic	Public Genome	3887587	0.297	3477	9	78
GCA_011014175.1_ASM1101417v1_genomic	Public Genome	3791625	0.3705	3581	10	82
GCA_011014195.1_ASM1101419v1_genomic	Public Genome	3906975	0.3032	3531	10	80
GCA_011014215.1_ASM1101421v1_genomic	Public Genome	3847854	0.2966	3498	10	79
GCA_011014225.1_ASM1101422v1_genomic	Public Genome	3819146	0.3364	3594	10	73
GCA_011014255.1_ASM1101425v1_genomic	Public Genome	3909237	0.2997	3536	10	80
GCA_011014265.1_ASM1101426v1_genomic	Public Genome	3857788	0.3339	3587	10	80
GCA_011014295.1_ASM1101429v1_genomic	Public Genome	3900190	0.2987	3566	9	79
GCA_011014305.1_ASM1101430v1_genomic	Public Genome	3884428	0.2921	3470	9	76
GCA_011014335.1_ASM1101433v1_genomic	Public Genome	3777092	0.307	3598	9	78
GCA_011014345.1_ASM1101434v1_genomic	Public Genome	3873413	0.2902	3484	10	79
GCA_011014375.1_ASM1101437v1_genomic	Public Genome	3706291	0.3063	3589	10	79
GCA_011014395.1_ASM1101439v1_genomic	Public Genome	3820899	0.2981	3648	11	78

GCA_011014415.1_ASM1101441v1_genomic	Public Genome	3883047	0.3028	3656	11	78
GCA_011014425.1_ASM1101442v1_genomic	Public Genome	3886862	0.2905	3997	9	70
GCA_011014445.1_ASM1101444v1_genomic	Public Genome	4202341	0.3021	3527	12	86
GCA_011014475.1_ASM1101447v1_genomic	Public Genome	3775133	0.3041	3658	11	78
GCA_011014545.1_ASM1101454v1_genomic	Public Genome	3891373	0.3185	3770	10	76
GCA_011014565.1_ASM1101456v1_genomic	Public Genome	4010473	0.3128	3769	9	61
GCA_011014595.1_ASM1101459v1_genomic	Public Genome	4009954	0.3239	3538	9	73
GCA_011014735.1_ASM1101473v1_genomic	Public Genome	3837899	0.3137	4093	6	73
GCA_011014745.1_ASM1101474v1_genomic	Public Genome	4221895	0.3019	3677	9	78
GCA_011014775.1_ASM1101477v1_genomic	Public Genome	3902622	0.301	3682	9	77
GCA_011014825.1_ASM1101482v1_genomic	Public Genome	3903471	0.3263	3582	10	84
GCA_011014925.1_ASM1101492v1_genomic	Public Genome	3824218	0.3362	3689	9	77
GCA_011014955.1_ASM1101495v1_genomic	Public Genome	3907211	0.3288	3690	9	77
GCA_011014965.1_ASM1101496v1_genomic	Public Genome	3906539	0.2855	3680	9	77
GCA_011014995.1_ASM1101499v1_genomic	Public Genome	3898638	0.2938	3379	8	79
GCA_011015015.1_ASM1101501v1_genomic	Public Genome	3668164	0.2909	3517	8	78
GCA_011015195.1_ASM1101519v1_genomic	Public Genome	3786638	0.3437	3802	10	79
GCA_011015305.1_ASM1101530v1_genomic	Public Genome	3987584	0.3279	3524	8	79
GCA_011015315.1_ASM1101531v1_genomic	Public Genome	3803211	0.2851	3512	8	79
GCA_011015335.1_ASM1101533v1_genomic	Public Genome	3795661	0.3279	3806	10	78
GCA_011015395.1_ASM1101539v1_genomic	Public Genome	3987443	0.2996	3591	9	65
GCA_011015415.1_ASM1101541v1_genomic	Public Genome	3859679	0.3175	3563	11	79
GCA_011015425.1_ASM1101542v1_genomic	Public Genome	3800184	0.3547	4015	9	80
GCA_011015435.1_ASM1101543v1_genomic	Public Genome	4222300	0.3213	3743	11	83
GCA_011015475.1_ASM1101547v1_genomic	Public Genome	3910953	0.2941	3557	11	79
GCA_011015495.1_ASM1101549v1_genomic	Public Genome	3792202	0.3036	3559	11	79

GCA_011015505.1_ASM1101550v1_genomic	Public Genome	3798005	0.3112	3561	11	79
GCA_011015535.1_ASM1101553v1_genomic	Public Genome	3798916	0.3278	3562	11	79
GCA_011015545.1_ASM1101554v1_genomic	Public Genome	3800227	0.3029	3622	11	80
GCA_011015555.1_ASM1101555v1_genomic	Public Genome	3853953	0.3159	3628	11	84
GCA_011015595.1_ASM1101559v1_genomic	Public Genome	3866946	0.3051	3641	11	76
GCA_011015615.1_ASM1101561v1_genomic	Public Genome	3876485	0.2925	3622	11	79
GCA_011015635.1_ASM1101563v1_genomic	Public Genome	3860097	0.3454	3683	10	81
GCA_011015655.1_ASM1101565v1_genomic	Public Genome	3904478	0.3248	3560	11	80
GCA_011015665.1_ASM1101566v1_genomic	Public Genome	3800274	0.3145	3560	11	82
GCA_011015685.1_ASM1101568v1_genomic	Public Genome	3799286	0.299	3555	11	79
GCA_011015715.1_ASM1101571v1_genomic	Public Genome	3796730	0.2966	3560	11	79
GCA_011015735.1_ASM1101573v1_genomic	Public Genome	3797447	0.3228	3562	11	82
GCA_011015795.1_ASM1101579v1_genomic	Public Genome	3800099	0.2936	3484	11	80
GCA_011016045.1_ASM1101604v1_genomic	Public Genome	3762997	0.2828	3625	11	78
GCA_011016095.1_ASM1101609v1_genomic	Public Genome	3863045	0.29	3582	9	75
GCA_011017095.1_ASM1101709v1_genomic	Public Genome	3899867	0.3216	3912	10	79
GCA_011017125.1_ASM1101712v1_genomic	Public Genome	4148498	0.3337	3739	11	79
GCA_011017145.1_ASM1101714v1_genomic	Public Genome	3937539	0.2928	3684	10	78
GCA_011017175.1_ASM1101717v1_genomic	Public Genome	3909499	0.2928	3645	11	76
GCA_011017235.1_ASM1101723v1_genomic	Public Genome	3862069	0.2806	3880	8	76
GCA_011017245.1_ASM1101724v1_genomic	Public Genome	4061684	0.2995	3743	10	76
GCA_011017265.1_ASM1101726v1_genomic	Public Genome	3929760	0.2957	3734	10	77
GCA_011017275.1_ASM1101727v1_genomic	Public Genome	3917155	0.304	3659	10	71
GCA_011017315.1_ASM1101731v1_genomic	Public Genome	3862412	0.3244	3664	10	77
GCA_011017375.1_ASM1101737v1_genomic	Public Genome	3864363	0.3338	3755	10	78
GCA_011017455.1_ASM1101745v1_genomic	Public Genome	3975595	0.329	3573	10	78

GCA_011017465.1_ASM1101746v1_genomic	Public Genome	3799270	0.2913	3701	9	75
GCA_011017495.1_ASM1101749v1_genomic	Public Genome	3914449	0.2922	3673	11	78
GCA_011017505.1_ASM1101750v1_genomic	Public Genome	3884615	0.2791	3666	9	75
GCA_011017535.1_ASM1101753v1_genomic	Public Genome	3881310	0.286	3770	8	74
GCA_011017545.1_ASM1101754v1_genomic	Public Genome	3961828	0.3059	3597	10	79
GCA_011017575.1_ASM1101757v1_genomic	Public Genome	3825875	0.2919	3869	10	84
GCA_011017585.1_ASM1101758v1_genomic	Public Genome	4025593	0.2948	3493	10	78
GCA_011017615.1_ASM1101761v1_genomic	Public Genome	3743766	0.3022	3656	11	79
GCA_011017635.1_ASM1101763v1_genomic	Public Genome	3888393	0.2835	3688	10	78
GCA_011017645.1_ASM1101764v1_genomic	Public Genome	3893783	0.3245	3747	10	78
GCA_011017655.1_ASM1101765v1_genomic	Public Genome	3976162	0.2913	3684	9	74
GCA_011017685.1_ASM1101768v1_genomic	Public Genome	3903329	0.3166	3767	8	78
GCA_011017715.1_ASM1101771v1_genomic	Public Genome	3999847	0.3207	3630	9	76
GCA_011017735.1_ASM1101773v1_genomic	Public Genome	3861879	0.2901	3733	11	76
GCA_011017755.1_ASM1101775v1_genomic	Public Genome	3977776	0.2898	3983	8	77
GCA_011017765.1_ASM1101776v1_genomic	Public Genome	4198842	0.2836	3586	10	77
GCA_011017775.1_ASM1101777v1_genomic	Public Genome	3826200	0.2819	4122	10	77
GCA_011017785.1_ASM1101778v1_genomic	Public Genome	4264670	0.3322	3751	10	77
GCA_011017835.1_ASM1101783v1_genomic	Public Genome	3976492	0.3101	3747	10	77
GCA_011017845.1_ASM1101784v1_genomic	Public Genome	3976740	0.2889	3738	10	77
GCA_011017875.1_ASM1101787v1_genomic	Public Genome	3963063	0.361	3749	10	81
GCA_011017885.1_ASM1101788v1_genomic	Public Genome	3975255	0.292	3842	8	78
GCA_011017895.1_ASM1101789v1_genomic	Public Genome	4040375	0.2928	3715	10	77
GCA_011017935.1_ASM1101793v1_genomic	Public Genome	3953003	0.28	3751	8	65
GCA_011017955.1_ASM1101795v1_genomic	Public Genome	3978554	0.2838	3860	8	75
GCA_011017965.1_ASM1101796v1_genomic	Public Genome	4052937	0.2935	3670	11	78

GCA_011017975.1_ASM1101797v1_genomic	Public Genome	3885041	0.2926	4002	9	77
GCA_011017995.1_ASM1101799v1_genomic	Public Genome	4205108	0.2862	3508	10	79
GCA_011018275.1_ASM1101827v1_genomic	Public Genome	3833113	0.3156	3661	11	78
GCA_011018285.1_ASM1101828v1_genomic	Public Genome	3890949	0.323	3856	10	80
GCA_011018295.1_ASM1101829v1_genomic	Public Genome	4000784	0.2846	4182	10	74
GCA_011018385.1_ASM1101838v1_genomic	Public Genome	4323914	0.2846	3761	9	73
GCA_011018395.1_ASM1101839v1_genomic	Public Genome	3969952	0.2883	3881	8	75
GCA_011018525.1_ASM1101852v1_genomic	Public Genome	4066520	0.3142	3681	6	72
GCA_011018575.1_ASM1101857v1_genomic	Public Genome	3890475	0.3014	3689	8	76
GCA_011018595.1_ASM1101859v1_genomic	Public Genome	3920613	0.2889	3880	8	76
GCA_011018625.1_ASM1101862v1_genomic	Public Genome	4066063	0.2863	3687	9	77
GCA_011018675.1_ASM1101867v1_genomic	Public Genome	3905906	0.2952	3654	11	79
GCA_011018725.1_ASM1101872v1_genomic	Public Genome	3889327	0.3557	3921	11	76
GCA_011018795.1_ASM1101879v1_genomic	Public Genome	4141632	0.3072	3647	11	79
GCA_011018815.1_ASM1101881v1_genomic	Public Genome	3896849	0.3004	3840	11	78
GCA_011018895.1_ASM1101889v1_genomic	Public Genome	4051467	0.3008	3632	8	77
GCA_011018945.1_ASM1101894v1_genomic	Public Genome	3861604	0.2894	3881	8	75
GCA_011018995.1_ASM1101899v1_genomic	Public Genome	4066542	0.3277	3748	9	85
GCA_011019005.1_ASM1101900v1_genomic	Public Genome	3963247	0.3042	3597	10	79
GCA_011019055.1_ASM1101905v1_genomic	Public Genome	3827299	0.3014	3678	9	81
GCA_011019065.1_ASM1101906v1_genomic	Public Genome	3946924	0.2847	4177	11	78
GCA_011019095.1_ASM1101909v1_genomic	Public Genome	4324852	0.3061	3750	10	77
GCA_011019125.1_ASM1101912v1_genomic	Public Genome	3973995	0.3195	3650	11	76
GCA_011019165.1_ASM1101916v1_genomic	Public Genome	3862767	0.3534	3754	9	81
GCA_011019215.1_ASM1101921v1_genomic	Public Genome	3967417	0.3285	3768	9	66
GCA_011019415.1_ASM1101941v1_genomic	Public Genome	4011528	0.2903	3822	8	77

GCA_011019425.1_ASM1101942v1_genomic	Public Genome	3999022	0.3431	3664	11	79
GCA_011019455.1_ASM1101945v1_genomic	Public Genome	3893254	0.3071	3633	11	80
GCA_011019525.1_ASM1101952v1_genomic	Public Genome	3863217	0.3246	3662	10	82
GCA_011019625.1_ASM1101962v1_genomic	Public Genome	3865210	0.2986	3600	10	78
GCA_011019745.1_ASM1101974v1_genomic	Public Genome	3825254	0.3269	3670	10	77
GCA_011019775.1_ASM1101977v1_genomic	Public Genome	3871282	0.3556	3597	9	77
GCA_011019895.1_ASM1101989v1_genomic	Public Genome	3908743	0.3026	3587	9	75
GCA_011019915.1_ASM1101991v1_genomic	Public Genome	3908265	0.3088	3590	10	77
GCA_011019945.1_ASM1101994v1_genomic	Public Genome	3905658	0.3013	3704	10	78
GCA_011019975.1_ASM1101997v1_genomic	Public Genome	3899182	0.3231	3591	10	77
GCA_011020005.1_ASM1102000v1_genomic	Public Genome	3907454	0.3204	3885	8	77
GCA_011020015.1_ASM1102001v1_genomic	Public Genome	4069384	0.2909	3856	8	76
GCA_011020075.1_ASM1102007v1_genomic	Public Genome	4033683	0.2939	3599	9	77
GCA_011020115.1_ASM1102011v1_genomic	Public Genome	3861043	0.2925	3535	9	69
GCA_011020175.1_ASM1102017v1_genomic	Public Genome	3831692	0.2784	3671	10	73
GCA_011020195.1_ASM1102019v1_genomic	Public Genome	3871905	0.2929	3657	11	67
GCA_011020255.1_ASM1102025v1_genomic	Public Genome	3890199	0.3182	3587	9	75
GCA_011020275.1_ASM1102027v1_genomic	Public Genome	3906242	0.3021	3660	11	79
GCA_011020285.1_ASM1102028v1_genomic	Public Genome	3891347	0.2897	3659	11	71
GCA_011020525.1_ASM1102052v1_genomic	Public Genome	3886062	0.3007	3576	9	75
GCA_011020535.1_ASM1102053v1_genomic	Public Genome	3895460	0.3058	3927	9	75
GCA_011020595.1_ASM1102059v1_genomic	Public Genome	4142381	0.2972	3919	8	74
GCA_011020655.1_ASM1102065v1_genomic	Public Genome	4136150	0.2977	3627	8	75
GCA_011020665.1_ASM1102066v1_genomic	Public Genome	3873546	0.2947	3574	10	78
GCA_011020735.1_ASM1102073v1_genomic	Public Genome	3836926	0.2873	3175	10	78
GCA_011020815.1_ASM1102081v1_genomic	Public Genome	3440104	0.2904	3647	11	76

GCA_011020875.1_ASM1102087v1_genomic	Public Genome	3860661	0.3034	3558	10	83
GCA_011020895.1_ASM1102089v1_genomic	Public Genome	3804147	0.2997	3879	8	76
GCA_011020915.1_ASM1102091v1_genomic	Public Genome	4065156	0.3181	3492	11	81
GCA_011020965.1_ASM1102096v1_genomic	Public Genome	3735398	0.2959	3647	11	80
GCA_011020995.1_ASM1102099v1_genomic	Public Genome	3877729	0.2986	3634	11	79
GCA_011021125.1_ASM1102112v1_genomic	Public Genome	3873090	0.2935	4177	11	78
GCA_011021215.1_ASM1102121v1_genomic	Public Genome	4323927	0.305	3733	10	78
GCA_011021325.1_ASM1102132v1_genomic	Public Genome	3920860	0.3062	3748	9	77
GCA_011021335.1_ASM1102133v1_genomic	Public Genome	3961475	0.3014	3743	9	80
GCA_011021375.1_ASM1102137v1_genomic	Public Genome	3960976	0.2847	3535	11	77
GCA_011021415.1_ASM1102141v1_genomic	Public Genome	3790007	0.3082	3741	10	78
GCA_011021435.1_ASM1102143v1_genomic	Public Genome	3926334	0.2991	3878	8	76
GCA_011021495.1_ASM1102149v1_genomic	Public Genome	4059534	0.2951	4249	8	76
GCA_011021515.1_ASM1102151v1_genomic	Public Genome	4449297	0.3262	3653	12	75
GCA_011021535.1_ASM1102153v1_genomic	Public Genome	3887780	0.3454	3738	9	82
GCA_011021545.1_ASM1102154v1_genomic	Public Genome	3951862	0.2986	4181	11	79
GCA_011021565.1_ASM1102156v1_genomic	Public Genome	4325658	0.3348	3662	9	77
GCA_011021615.1_ASM1102161v1_genomic	Public Genome	3877660	0.3164	3548	11	86
GCA_011021655.1_ASM1102165v1_genomic	Public Genome	3791445	0.3515	3761	9	65
GCA_011021735.1_ASM1102173v1_genomic	Public Genome	3966395	0.3303	3661	11	75
GCA_011021755.1_ASM1102175v1_genomic	Public Genome	3892108	0.3163	3685	9	77
GCA_011021765.1_ASM1102176v1_genomic	Public Genome	3905967	0.3065	3579	10	77
GCA_011021895.1_ASM1102189v1_genomic	Public Genome	3900573	0.3066	3580	10	79
GCA_011021905.1_ASM1102190v1_genomic	Public Genome	3904612	0.3107	3585	10	77
GCA_011021915.1_ASM1102191v1_genomic	Public Genome	3899682	0.3084	3591	9	77
GCA_011021955.1_ASM1102195v1_genomic	Public Genome	3906988	0.3304	3599	9	76

GCA_011022075.1_ASM1102207v1_genomic	Public Genome	3877160	0.2776	3735	9	74
GCA_011022085.1_ASM1102208v1_genomic	Public Genome	3969885	0.3041	3661	10	80
GCA_011363175.1_ASM1136317v1_genomic	Public Genome	3859989	0.2969	3747	11	80
GCA_011389495.1_ASM1138949v1_genomic	Public Genome	3918049	0.2997	3693	10	68
GCA_014068615.1_ASM1406861v1_genomic	Public Genome	3940268	0.269	3647	27	80
GCA_014805065.1_ASM1480506v1_genomic	Public Genome	3918289	0.2937	3884	3	59
GCA_014805085.1_ASM1480508v1_genomic	Public Genome	4064247	0.3101	3967	8	76
GCA_014805095.1_ASM1480509v1_genomic	Public Genome	4108059	0.294	3878	4	59
GCA_014805105.1_ASM1480510v1_genomic	Public Genome	4064564	0.2885	3884	4	43
GCA_014805145.1_ASM1480514v1_genomic	Public Genome	4059968	0.3148	3883	3	57
GCA_014805165.1_ASM1480516v1_genomic	Public Genome	4067487	0.3012	3872	4	57
GCA_014805185.1_ASM1480518v1_genomic	Public Genome	4063967	0.3023	3884	4	50
GCA_014805195.1_ASM1480519v1_genomic	Public Genome	4062365	0.3027	3882	4	60
GCA_014805215.1_ASM1480521v1_genomic	Public Genome	4062152	0.3121	3937	4	58
GCA_014805235.1_ASM1480523v1_genomic	Public Genome	4120534	0.3506	3942	10	68
GCA_014805255.1_ASM1480525v1_genomic	Public Genome	4129110	0.2962	3877	4	51
GCA_014805275.1_ASM1480527v1_genomic	Public Genome	4065178	0.2833	3892	6	68
GCA_014805305.1_ASM1480530v1_genomic	Public Genome	4075116	0.2717	3912	9	76
GCA_014872905.1_ASM1487290v1_genomic	Public Genome	4138590	0.2907	4070	5	27
GCA_016085375.1_PDT000162924.3_genomic	Public Genome	4155425	0.3155	3561	8	60
GCA_016085455.1_PDT000162920.3_genomic	Public Genome	3818549	0.3093	3571	7	56
GCA_016085475.1_PDT000162923.3_genomic	Public Genome	3829895	0.3122	3572	7	62
GCA_016085495.1_PDT000162918.3_genomic	Public Genome	3830725	0.3196	3576	6	63
GCA_016085515.1_PDT000162922.3_genomic	Public Genome	3832024	0.2759	3512	7	60
GCA_016085535.1_PDT000162917.3_genomic	Public Genome	3778163	0.3211	3566	8	57
GCA_016085555.1_PDT000162921.3_genomic	Public Genome	3822658	0.3201	3564	5	52

GCA_016085625.1_PDT000162916.3_genomic	Public Genome	3825766	0.3214	3569	7	61
GCA_016085715.1_PDT000162915.2_genomic	Public Genome	3828428	0.3149	3656	7	61
GCA_016798345.1_ASM1679834v1_genomic	Public Genome	3881885	0.2817	3542	21	80
GCA_016838665.1_ASM1683866v1_genomic	Public Genome	3815652	0.2815	3974	27	80
GCA_016989565.1_ASM1698956v1_genomic	Public Genome	4090796	0.3007	3578	11	66
GCA_016989605.1_ASM1698960v1_genomic	Public Genome	3867597	0.3026	4181	10	71
GCA_016989635.1_ASM1698963v1_genomic	Public Genome	4281823	0.3307	3967	12	78
GCA_016989715.1_ASM1698971v1_genomic	Public Genome	4154801	0.2959	4229	10	79
GCA_016989755.1_ASM1698975v1_genomic	Public Genome	4328178	0.3235	4181	12	80
GCA_016989775.1_ASM1698977v1_genomic	Public Genome	4280074	0.3347	3748	9	81
GCA_016989785.1_ASM1698978v1_genomic	Public Genome	3972687	0.2881	4691	12	63
GCA_016989825.1_ASM1698982v1_genomic	Public Genome	4699392	0.3244	4297	10	79
GCA_016989855.1_ASM1698985v1_genomic	Public Genome	4352993	0.3077	4298	10	79
GCA_016989895.1_ASM1698989v1_genomic	Public Genome	4369978	0.3307	3916	11	79
GCA_016989975.1_ASM1698997v1_genomic	Public Genome	4098296	0.3246	4161	12	80
GCA_016990015.1_ASM1699001v1_genomic	Public Genome	4262384	0.37	3824	11	71
GCA_016990025.1_ASM1699002v1_genomic	Public Genome	4016700	0.3301	4063	11	78
GCA_016990055.1_ASM1699005v1_genomic	Public Genome	4207851	0.3145	3770	9	78
GCA_016990065.1_ASM1699006v1_genomic	Public Genome	3946920	0.3163	3939	11	79
GCA_016990115.1_ASM1699011v1_genomic	Public Genome	4105532	0.3077	4007	10	79
GCA_016990155.1_ASM1699015v1_genomic	Public Genome	4167208	0.307	3717	9	64
GCA_016990195.1_ASM1699019v1_genomic	Public Genome	3904337	0.3145	4188	12	80
GCA_016990235.1_ASM1699023v1_genomic	Public Genome	4270285	0.2884	4175	11	67
GCA_016990255.1_ASM1699025v1_genomic	Public Genome	4271365	0.2977	3883	10	74
GCA_016990275.1_ASM1699027v1_genomic	Public Genome	4062838	0.2936	4188	10	64
GCA_016990285.1_ASM1699028v1_genomic	Public Genome	4273021	0.3401	3551	9	70

GCA_016990315.1_ASM1699031v1_genomic	Public Genome	3835422	0.341	3813	15	71
GCA_016990335.1_ASM1699033v1_genomic	Public Genome	4038662	0.3163	3942	12	79
GCA_016990345.1_ASM1699034v1_genomic	Public Genome	4110538	0.3413	3551	9	67
GCA_016990375.1_ASM1699037v1_genomic	Public Genome	3834757	0.3248	3943	11	79
GCA_016990395.1_ASM1699039v1_genomic	Public Genome	4112819	0.3373	3610	9	77
GCA_016990415.1_ASM1699041v1_genomic	Public Genome	3850232	0.3247	4015	12	67
GCA_017330665.1_ASM1733066v1_genomic	Public Genome	4154648	0.2912	4402	8	54
GCA_017330685.1_ASM1733068v1_genomic	Public Genome	4332555	0.2885	4000	9	71
GCA_017330695.1_ASM1733069v1_genomic	Public Genome	3843066	0.3052	4023	10	71
GCA_017330715.1_ASM1733071v1_genomic	Public Genome	3978905	0.2981	4213	9	56
GCA_017330725.1_ASM1733072v1_genomic	Public Genome	4185706	0.2788	4753	7	68
GCA_017330765.1_ASM1733076v1_genomic	Public Genome	4187706	0.2733	4668	4	58
GCA_017330785.1_ASM1733078v1_genomic	Public Genome	4162320	0.3027	4224	9	71
GCA_017330795.1_ASM1733079v1_genomic	Public Genome	4199797	0.2912	4409	5	64
GCA_017330815.1_ASM1733081v1_genomic	Public Genome	4331526	0.2755	5962	7	54
GCA_017330845.1_ASM1733084v1_genomic	Public Genome	4163377	0.2891	6149	9	76
GCA_017330865.1_ASM1733086v1_genomic	Public Genome	4203614	0.3006	4090	9	68
GCA_017330885.1_ASM1733088v1_genomic	Public Genome	4198117	0.282	4595	9	66
GCA_017330905.1_ASM1733090v1_genomic	Public Genome	4141306	0.2869	5987	5	63
GCA_017330925.1_ASM1733092v1_genomic	Public Genome	4217736	0.285	6389	9	56
GCA_017330945.1_ASM1733094v1_genomic	Public Genome	4158067	0.3186	4060	9	80
GCA_017330965.1_ASM1733096v1_genomic	Public Genome	4158619	0.2807	4192	5	52
GCA_017330985.1_ASM1733098v1_genomic	Public Genome	4139909	0.3016	4218	9	53
GCA_017331005.1_ASM1733100v1_genomic	Public Genome	4155710	0.2915	4303	5	61
GCA_017331025.1_ASM1733102v1_genomic	Public Genome	4227167	0.2908	4253	9	64
GCA_017331045.1_ASM1733104v1_genomic	Public Genome	4153245	0.2905	4137	5	50

GCA_017590175.1_ASM1759017v1_genomic	Public Genome	4150368	0.2905	3609	17	73
GCA_017591115.1_ASM1759111v1_genomic	Public Genome	3790015	0.2794	3932	10	71
GCA_017773255.1_PDT000985504.1_genomic	Public Genome	4057568	0.2813	3828	5	59
GCA_902386235.1_UHGG_MGYG-HGUT-02355_genomic	Public Genome	4131227	0.2657	3552	2	67

Table S4. 2933 core genes of 59 isolates.
rpsG,spoVT,sigA,codY,remA,gtaB,ychF,rpmJ,abrB,rpsK,rplP,clpB,ispG,parE,leuS,pduA_1,group_1233,rpsU,group_66144,yoaH_2,group_3769,dus_1,endoI,dhaD_2,ctfB,atpD,ileS,rplA,guaA,cheY_2,ybiT_4,eno,rnmV,ftsH_3,sigG,der,pduA_3,sigK,pstB3,mnmE_1,sutR,sigH,nrdA,group_790,group_3774,cutC_1,dnaA_2,yxlF_5,group_896,hcp,group_2314,rpoB,group_5585,feoB_2,cobC,yacP,tdcB,rplC,rplD,rpsS,rplV,rpsC,rplN,rplE,rplF,rpsE,adk,map_1,rpoA,ptsI,cdaA,dadA,buk2_2,cysC,korA,korB,padE,glmM,gph,megL_2,ndhI,cshA,glsA,pyrR_2,glnQ,mleN_3,mcpB_2,pepT,artQ_2,ddl,braC,livH,rbsC,lptB,yjjP_1,lemA,group_2607,tktB,dxs_1,ftsE,group_271,uvrA_2,rodA,pbpA,yhaM_1,ycdX,murB,yvcJ,group_1423,whiA,dnaE,pfkA,licC,spoIIAA,spoVAD,yigZ,yebC,group_2861,ruvA,tgt,gloC,hemZ_2,tam,tal_1,ulaB,manR,group_3510,pduA_2,cutD,pduV,dnaK_1,ccmL,ccmK,adhE_3,group_128,proC,ssuC_1,tauB,hadI_2,oppA_1,oppB,dppC_1,oppD_1,hslO,ktrA,pheS,ydcP,mutS2_2,group_3745,speA_1,pglF,wbpA,group_1737,group_2385,group_1965,fabG_1,iap_1,nadE,ytrA_1,potB,potD,msbA_2,yugI,hup,mazG,spoVB_1,prsA1,mfd,phoR_5,srrA_4,prs,glmU,purR,glnA,dnaN,recF_2,group_8656,gyrB,mutS2_1,group_5081,ribZ,cynR_1,mcpB_1,gcvPB,fabH_1,mcpS,group_726,bcd_2,thlA,hbd,mcpB_7,pflB,rplY,engB,lon1,lon2_2,clpX,clpP,tig,pyrD,pyrK,pyrC,pyrB,hgdC_1,fldC_3,gatB,gatA,pcrA_1,group_641,pgi,fldH,carE_3,carD_3,carC_2,guaB,groL,groS,yheS,yehU,cstA_2,group_205,group_1751,prfC,lysC_2,ftsH4,pepV,polA,mreB_1,mreC,minC,minD,minE,mrdB_2,group_910,obg,group_3029,yvhJ,yabJ,selD,selA,selB,yqeN,rpsT,gpr,hrcA,grpE,dnaK_2,mtaB,group_4283,pgpH,dgkA,ccpN,ppdK,dgt,thiC,mnmA_1,dpaL,uacT_1,mtaD,purE_1,purC,group_1849,purM,purN,purH,pepD_2,mepA_4,hadI_1,rny_1,carE_1,carD_1,carC_1,hadC,hadB,kduD,natB_1,natA_2,bmrR_3,group_406,rny_2,rimO_1,spoIIIE,lysC_1,pnp,truB,nrnA,infB,polC,rasP,ffh,smc_3,acpP_1,pta_1,group_6234,prdA_1,cobQ_1,cobD_1,cobD_2,hemC,nasF,hemB,cbiD,cbiJ,pduX,hmuU_2,btuF_1,dhaK,smc_2,larA_3,group_2487,group_823,group_2600,aspC,feoA_1,feoB_3,feuB_2,yclQ,group_1030,lhgO_2,trxB_1,group_4302,kdsD,feoB_4,feoA_2,serB1_1,rnfC_4,udk_1,trmR,typA,rnjA_1,fur_1,yrrK,alaS,iscU,iscS_1,cymR_1,group_1843,group_765,purL,arcC1,glyA,group_1493,csoR_1,glpX,ntpA,ntpG,flgG_2,flhA,flhB_1,group_105,magA,pgdA_2,mepA_6,sph,pepIP,group_2138,murC_2,cobQ_2,group_935,group_1243,ftsH_2,group_2392,rsxA,panF,murAA,mnaA,tagO,group_8651,upp,cphB,macB_2,metG_2,norM,spmB,spmA,group_22507,darA,folT,dapB_1,rpsL,rpoC,nusG,cysS,mcsA,ctsR,rpsJ,rplW,rpmC,rplO,secY,rpsM,rpsD_2,rplQ,ecfT,iadA,trxA_1,group_2051,glmS,fucP,tcdA,group_3396,recR,group_3568,dnaX_2,serS_2,preA,group_173,chiA1,group_2069,fliY,group_2896,group_2089,sbcD,ziaR,cadA,psuK_1,cysK1,yxeN_1,iscS_2,sstT_1,group_2165,group_1425,uvrB,uvrC_1,pyk,tagA,spoIIAB,sigF,ponA,hflX,ruvB,chuR_2,recJ_1,relA,aspS,ulaC,ulaA,pduL,viaA,puuR_2,cutC_2,flr_3,queT,fpgS,group_2865,group_3032,ssbA_1,dapH_2,patA_2,dapA,pheT_2,zapA,hmo,group_278,cqsS_2,group_108,hflC,pepP,cstA_1,group_581,potA_1,rhaR_1,dctA,copA,xdhA_2,grdD,grdC,grdE,gapN,gsiC,oppC,sdhA,tyrS_2,greA,dus_2,coaX,fhs,spoIIE,spoVG,murC_1,yrpC,murAB,secA_2,group_380,yjjW,group_4043,group_837,thiH,aprX,cmpR,accD,accC,fabZ,fabF,fabG_2,group_693,dnaC_1,yqfL,solA,lon2_1,rplI,group_5500,rpsF,fldC_4,soj,noc,rsmG,mnmG,mnmE_2,group_2012,yidC2,group_2499,ltaS2,asnS,norV_1,group_1159,feuB_1,feuC_1,nifH1,gcvPA,pdhD,hgdC_2,carE_4,group_2160,group_1255,pyrE_2,pyrI,group_2870,gatC,sstT_2,group_575,fldC_1,fldB_2,fldA_2,cfiB,tsaD,gltD_2,group_1152,tsaB,tyrS_1,trpS,fieF_1,group_4008,group_1084,yqeY,cdd_2,era,cotI_1,group_487,hdc,yueB,group_795,isdE,argF',dapE_1,ribBA,rlmCD_1,yvoA,steT_1,fldA_1,pepD_1,group_1019,group_1838,aspC_3,pgsA,rbfA,pyrH,tsf_1,rpsB,rbgA,sipS,rplS,group_14445,rpsP,cbiM,cbiA,cbiC,cbiH,btuR,asrB,fecE_1,group_3767,group_1031,glpT,argS,nlpA,amaA,group_1269,prdA_2,prdA_3,prdA_4,recG,rpe,rsgA,prkC,rpoZ,gmk,sigE,ftsZ,group_4755,apeA,ntpD,ntpB,group_1647,group_716,flhF,fliP,group_2850,pomA_2,fliG,fliF,flgC,clsA,flgK,fliM,cheC,cheA,cheR,cheD,serB1_2,por_2,recJ_2,yesS_2,glpF,arcD,arcA_1,group_2020,group_202,group_1826,group_5036,group_1908,btr_2,yhjQ,hemE_3,metH_1,nqrF,mleN_2,hemE_1,group_745,pncB2,dppC_2,appA_1,napF,padI,cooS1,ptsG_3,cshE,adhE_5,adeC,ywnA,malH,malP,yxdL_2,secG,tpiA,pgk,gap1,group_1870,group_3783,pepA,group_551,group_699,yjbM,cdr_2,pagR,cpdA_2,flhB_2,group_355,prfB,secA,recD2_2,tdk,exsA,yabG,group_1869,glgM,mftC,purE_2,recD2_3,group_167,arcA_2,group_2504,corA_2,group_1456,rplGB,rplJ,rplK,vanW_1,rlmB,thyX,mrnC,group_8664,disA,radA,rplB,rpsH,ecfA1_1,rplM,rpsI,phoU_1,zraR_1,nudC,sfsA,ubiB,mcpB_5,dmdA,addB,addA,thiD_2,lysN_3,mngR_1,group_974,group_2056,ecfA1_2,psuG,zupT_2,yxeM_1,tilS_2,yxlF_6,mleN_1,alr1_2,ftsX,ctpA,fhaB,group_3755,dgaR,wcaJ,group_2720,yunB,queA,hisS,group_3356,immR_2,adhE_1,adhE_2,

group_1398,group_2867,group_1654,aaaT_2,valS,asd,tylM1,thrZ,aviRb,iolX,rip2,mcp1,ceo,group_2891,ytrB_1,ypdB_1,group_417,trxB_3,trxB_2,alsT_3,group_2597,group_2642,ydbD,group_200,glyQS,lysS,hpt_2,group
_4044,pth,rimO_2,group_2624,group_2291,group_3062,yycJ,purA,dnaC_2,gdpP,ssbA_2,csd_2,parB,group_1107,spo0A_1,glxR,group_311,greB,yusV_1,group_74,gcvT,group_2181,ycgJ,rpoH,group_1938,adeP,group_15
78,iolI,pyrF,group_6249,fldI_3,pyrK_1,rex,hmpT,helD_2,aroC,yhdE,rluD_2,group_1497,dnaJ_1,prmA,rsmE,ybeY,tsf_2,group_2113,group_2436,cwlJ,dapE_2,helD_1,hyuA,pucD,yfcA,nagE,ycaM,yflN,purB,group_1199,r
ecA,tepA,group_569,dprA,trmD,rnc,plsX,ackA,group_1489,tipA,arnB,desV,tipA_2,group_234,cbiN,asrC_1,walR_4,group_1955,add,recQ,pheT_1,aspS2,yfkM,walR_5,group_1253,pchR_1,ettA_1,vioD,chiA,gltX,group_9
75,gadC,prdA_6,group_245,group_7189,stp,rsmB,priA,coaBC,spoIVA,pstA,pstC_1,pstS1,nrdR,spoIIGA,ftsA_1,spoVD_2,mltG,hprK,ydjZ_2,cofC,arnC,fprA1_2,rihA,yloB,flgG_1,ylxH,flgG_3,fliE,argG,group_4274,group
_1271,fliN,cheW_2,glcT_2,nfo,glpP,gyrA_1,galE,glpK,queC,ydaM,group_1259,lutR,ugpB,metQ,modA,group_3316,rlmL,exoA,pyrR_1,spoVD_1,rluC,crp_2,group_240,larA_1,artP,aspC_2,vnfA,rsxD,group_1790,walK_3
,walR_8,group_1114,group_2509,phoP,ptsG_1,yxdM_1,graS_2,graR_2,smpB,gpmI,cinA,cysK,group_2077,hrp1,group_1473,dnaJ_2,metK,mreB_2,spoIIID,lytB_1,rpiB,ywlE,ywlC,prfA,group_831,rpmE,rho,pyrG,ugtP,ve
g,larC,ccpA,ispF,iap_2,yfiC,tmk,abgB,cobB,group_682,mtrB,sseB,rplL_2,group_2128,metH_2,glcR,hbpA_1,group_8665,dauC,group_2495,hom_1,acoR_2,adhE_4,ttdA,group_916,group_780,sorC,group_3397,group_144
53,nrdB,uvrA_1,group_2185,icd,panB,group_1899,group_2067,betL,cymR_2,group_3539,group_1504,yhdN_1,celA,dtd,ravA,bssD,dapB_2,oppF_1,group_2863,ecsA,infC,rplT,pta_2,ctc,group_1517,group_8647,cheY_1,
mngR,fbp,asnC,adiC_2,srpC_2,mcpA,thiT,metG_1,lolE,rnjA_2,topB_2,mneP,appA_2,sdhB,ydhD,group_316,cysL_2,accA,fabH_2,group_1371,pgcA_2,rpsR,ykuT,group_2290,group_2289,walR_2,glnK,adaA,rhlE,bglF_1,
group_1958,gcvH,group_611,group_1773,rghR,group_46,carD_4,crt,group_7206,rdgB,group_6248,gltD_3,ypdB_3,group_5063,rplU,dacB_1,trmK,glpQ_1,purF_2,gltD_1,ribH,cutS,bcd_1,glcT_1,group_1837,bioB,sodA,gr
oup_2349,cca_1,dnaA_1,arnF,ribF,nusA,rimP,frr,comM,rimM,group_979,group_1863,aguA,lexA_1,cbiF,asrA,group_1510,regX3_2,group_492,cypC,dsbD_2,group_1764,pqqE,metN_1,group_1384,group_4532,group_426
6,rnfC_5,glnG,group_2423,def1,ansA,group_809,walR_7,argF,nadD_2,ldhB,yciV,group_766,def1_1,group_1207,hag_2,yfmS_2,cheW_1,cheB,group_441,glcB,group_1553,cdr_1,group_710,group_361,hxlR,ldh,yhdN_2,to
pB_1,asnA,namA,hemE_2,modB,uraA,aroB,divIVA,group_1825,mraY_2,murE,spoVB_2,yxeP,ftsA_2,yxeO,yxeN_2,dppB_2,padH,group_80,norG,tlyA,recN,hndC,hndD,larA_2,hexR,mcpC,uppP_2,ygjI,ttcA_2,uacT_2,gr
oup_2612,cspL,murR_1,group_2092,phoB,resA_1,group_1039,exoX,sstT_3,rnr,mglA,tmpC,nth,cysE,dagK,rpsA_1,hpf,ttcA_1,atpA,mfpsA,manC1,yocH_1,group_4650,walK_1,walR_3,fusA_2,acoA,bfmBAB,pdhC,lpd,gr
oup_849,srlD,group_3063,group_719,comEC_2,livF,sbcC,hadI_3,group_329,mnmA_3,pdaA_2,lysN_2,group_5551,ligA_2,group_776,group_675,drrA_1,group_7230,group_4317,csoR_2,ndhS,group_3763,group_822,mur
D,yabP,rsxB_6,mcp4_2,group_2623,opuE,baiN_2,group_5597,nupX,phnPP,lsrC,artM_1,group_622,zraR_3,ligA_1,brnQ_3,alkA,group_2049,recD2_1,spoIVFB,trpP,mepA_5,lhgO_1,ptsH,spoVS,rpsO,ftsY,queD,group_14
35,cbiG,crp_1,moeA_3,rbr3B,yfcE,fecE_2,group_1223,ynjB,prdA_5,thiN,rlmN,group_1137,gpsA,group_1138,group_855,group_1732,hemZ_1,group_8619,murG,ywaC,saeR_1,group_1480,alsT_5,rbsK,deoC,fldI_1,ogt,gr
oup_1260,group_4249,rluD_1,sepF,ftsW,group_2826,patB_2,degS,oppD_2,mdtG,rbr1,group_1562,group_565,group_2025,group_2342,group_500,group_590,group_433,group_7184,nusB,megL_1,xerD_2,punA,pdp,gerN
_2,hndA,rluB_2,ydjZ_1,group_1789,pgdA_1,group_1975,group_4039,ypdF_2,puuR_1,kdgK,yvdP_1,dsbD_1,gloB_2,rpoN,trmL,group_7238,aas,fba,prmC,group_4321,group_2507,group_2743,yknX,group_2388,rsmA,yhj
X,group_5079,group_4663,group_58,group_1934,group_1430,group_152,celD,apt,group_8627,pduU,ykuD_2,rapF,csxA,ybhS,group_2277,group_1780,srpC_1,group_1778,ctpE,group_2596,mtnK,grxC,group_415,group_
452,group_3789,map_2,group_4390,acpP_2,merP,pflA,group_1855,group_2048,group_859,group_6250,hydA,tsaE,group_4006,group_370,group_2154,fprA1_1,bioY_2,rplGA,group_262,topA,hemN_2,mraY_1,cobU,glc
A,group_1225,melR,fhuB,irtA,group_43,mdlD,mepA_7,group_678,fmt,phoU_2,resE_6,corA_1,group_3856,swrD,srrA_2,ugpC,group_1188,proX,puuB,group_1258,group_4248,group_2568,group_2127,rsmH_1,group_66
2,bdhA,nfrA2_2,murR_2,group_598,htpG,sphR,chiA1_1,group_1828,nadK,ybiT_3,group_527,group_2830,baiN_1,manA,mcpB_6,adrA_1,yohK,group_1682,xpt_1,glvR,udk_2,group_4035,mepM_2,yknY_3,group_2071,d
td3,speA_2,group_5085,fumB,group_119,gatD,srlA,srlB,group_2516,group_1583,panE,wbpI,group_4287,group_7204,tpx,dapH_1,mazF,group_3048,amj,mtnA,cph2_1,rlmH,group_3786,group_866,licT_1,group_24,nifD,
mccA,group_2603,rbr3A,rluB_1,fadD3,ttcA_3,group_1697,group_507,dfx,group_3354,group_5064,group_4627,group_674,hcrA,ndhF_1,group_6232,gabR_2,sirC,group_289,tlp,group_1354,ylmC,group_715,sigD,yscN,gr

oup_1335,group_3171,yoaB_1,group_1625,group_1478,lspA,mtnN,group_4599,nudL,dacF_2,drrA_2,mqnD,group_1387,cat_1,pyrE_1,dhaD_1,treB,group_1396,lolD_3,mepA_2,moeA_2,group_2575,btuD_2,gadB,truA_1,
rqcH,group_1328,group_5041,spoIIIAE,group_4257,ispA,argR,spoIIM,rsxB_1,cmk,miaB,dinG_2,group_363,rnfE,rnfG,graR_3,cggR,ribU,atpE_2,group_984,group_879,cobS,acoR_3,group_3001,group_2168,ydaF_2,grou
p_3414,asD,yhbE,group_1144,group_4010,group_8614,group_265,barA,adiC_1,hepA,group_1944,mtnB,group_2907,group_3209,group_3770,norR_4,ytfE,group_1957,group_1513,perR_3,group_574,adeQ,gloB_3,dxr,def
_1,group_514,cbiL,cqsS_1,group_2882,metP,group_10734,group_3733,plc,group_2353,resA_2,group_2569,group_1549,aroF,oppF_2,trkG,group_1925,dtpA_1,treR,phoR_2,tupA,tupB,moaA,moaC,ydhV,group_342,group
_479,dxs_2,dacB_2,tqsA_1,draG,group_431,lexA_2,dtpA,rapA_2,group_2238,group_3562,lrgA,uppP_1,malX_2,pagL_2,apbC,group_1672,rpsQ,srlE,ndhI_2,group_222,group_3754,group_457,pdaA_3,copZ,group_22482,
guaD,srlR,group_5094,resE_1,gloB_1,yvdT,rpmA,dnaG,ettA_5,ymdB,tetD_1,sigW_1,group_3166,cysA_2,bdlA,group_2567,yecS,tcyA,pdxK,helY,znuA,group_2565,hmuU_3,group_5541,sleB_3,group_3161,yjdF,gatY,tr
eA,mutS_1,sgcG,tupC,mog,moeB,dapF,xseB,group_4254,dacF_1,group_1557,mdeA,xerD_3,group_1242,group_1447,lysP,group_225,cotI_3,group_830,group_188,group_2760,ywpJ,hutI,rplX,yciB,mcp2_2,nudF,pgtC,pot
A_2,fchA,rpsD_1,kanC,graR_1,spsB,tal_2,ytrF_2,group_199,group_4041,csaA,group_4014,yidA,group_1304,lldR,group_3729,group_1492,malR,rapA_1,cysL_1,yxdL_5,degU,trkA,dhaS,group_2834,lacC_1,ahpD,mcp2_
1,efp,group_4601,group_7185,group_2532,cheX,ypuA,ybbH_2,udg,rpsN1,rpmD,folD,citS,group_2042,dtpT_2,group_4259,yxdL_1,bceB_2,rpmI,rpfG_1,mntP_2,group_1946,ftsB,yddE_2,rng,yhbY,regX3_1,group_4263,fo
lP,yhhW,speG_1,yueB_1,group_1584,group_2464,group_1075,hisK,group_1270,aaeB,hisB,hisD,mcpA_1,hom_2,metA,aroH,group_2780,miaA,group_449,DNPH1,ubiE_1,group_683,thiD_1,nhaX,flr_2,phnV,por_1,graS_
1,group_640,trmB,ytrH,group_2262,group_2055,group_781,group_5599,yfmC_1,group_2888,group_1656,corC,purD,nlhH,phoR_1,ydaF_3,ycsE,mapP,group_8604,hisG,mraZ,group_2230,inlA_2,group_3321,scpB,ytfJ,ch
eY_3,cheV,asrC_2,nhaP,rpmG2,group_14490,norV_2,tqsA_2,ulaE,group_1094,puuR_3,group_2753,group_6274,fdx_2,group_2258,ytrB_2,pgmB,group_1400,group_787,pilT,hisF,adhA,group_3148,znuC,spoIIIAB,spoIV
B,gerN_3,cshC,group_2510,group_112,tmung,rplR,group_815,sdpR_1,group_2497,group_1279,pdxA2,denK,glpR,group_2841,cbiQ,ftsH_1,gap,hisA,nprM_2,group_293,epsE_3,hydA_1,rimI_2,mutS_3,group_2130,group
_10751,ybiV,murJ,neoA,group_2741,group_3378,lgt,gntT,gutB,cooS2,group_5078,group_3375,bioY_1,spxA,pgaC,group_14450,hisI,hisH,scpA,group_1916,rpfG,yhdG,group_2505,mgtE,ynjE,group_3370,group_5069,gro
up_1161,group_10745,ytrA_2,cfr,yfmS_1,group_3516,fbiB,ribU_1,group_788,group_917,ycjP,group_1059,group_1389,group_2456,graR_4,tdcF_2,spoIIIAF,ypfD,lysN_4,mutL,hfq,pstA2,pstS,crr_2,yclM,group_973,grou
p_1662,lytC_2,group_907,nrdG_1,yhbU,group_387,dosC_2,znuB,yxlF_2,resE_10,xseA,yjeM,cvfB,pstB,pstC_2,rsmI,rpsA_2,group_3071,group_190,serS_1,trxA_2,group_2604,group_7200,cdsA,group_734,ftsL_1,group_
4253,soxS_2,group_3737,group_3369,group_2872,group_2143,rbbA,group_2264,group_1321,cspA,group_2239,apbE,malX_1,arsC,rub,group_1927,group_87,perR_2,ssbA_3,group_870,group_4286,group_5598,group_21
61,tetR,hssR,graS_4,saeR_2,yeeO,kanE,ppiB,mrdA,group_1024,group_3331,hssS,glpE,group_2617,agrB_1,kdgA,ilvD,pagL_1,group_3779,group_2893,group_3395,yoaB,flr_1,cynR_2,yknY_1,group_3021,yeiL_2,group_
792,ftnA,group_270,group_2169,group_1760,group_543,acnR,niaX,dbpA,group_2458,lytC_1,group_4016,ktrB,group_2170,group_1285,group_3352,yvdD,yjaB_1,nprB,msrA,fepC,pchR_2,group_3050,group_1649,group_
813,hypB_2,sfrB,group_509,group_3757,group_3031,group_5072,bglH_2,group_463,cbpA,ydcZ,group_3768,yocK,group_2400,vanW_2,group_4298,acpS,tetM,dut,group_3793,group_1395,lytG,dosP,motB_1,csrA,arnT,c
ssS,cssR,group_6287,group_3776,group_1361,group_698,group_1070,group_2026,group_3544,comB,yodJ_1,lnrL_2,group_791,group_1085,group_1671,cbiK,group_3041,group_1952,group_4275,group_2355,btuF_2,gro
up_1238,zupT_1,ispE,sspH_2,walR_1,bchB,group_4631,apeB,pomA_1,group_2842,arcB,group_4000,lytB_2,group_2340,inhA,ykoT,ohrR,acrR,tilS_3,zntR,group_852,yvbK,group_3042,group_962,atpF_1,glcK,group_63
0,group_4597,group_560,rssB,group_690,group_2080,atpB,sigK_1,group_187,thiE,group_1286,group_1439,group_519,group_900,group_2271,group_542,group_100,ntpC,ntpK,malL,group_260,rsxB_2,group_14483,thrC
,plsC,ubiE_2,group_4637,epsE_2,natB_2,plaP,group_820,group_3187,rnpA,group_4313,slt_1,nadD_1,group_1122,group_1071,group_1564,group_4262,yodB,mta,group_2883,atpE_1,fieF_2,metI,nadA,group_2835,nhaP2,
group_5038,graS_3,proS_2,group_563,thrB,cdaR,fsaA,yocH_2,preT,group_6278,thiM_2,nnr,rlmCD_2,hpt_1,patA_1,ywqG,group_1966,group_1109,group_1536,yniC,slcC,mrdB_1,cfa,fldH_1,lysA,group_2414,group_130
6,vfr,ribD,ltaS1,gerN_1,group_2027,azoR,dinG_1,tgl,arlS,arlR,group_2708,group_408,oxdD,yvgN,panM,rsiV,gltR,group_679,omcB,group_1861,maa,group_2468,ilvE,cydD,lon,group_2825,group_3319,splB,atpG,atpF,gr

oup_3055,cphA,cwlD,yccM,group_1080,panD,gloB_4,COQ5_2,murK,epsE_1,ppnP,dinG,icaA,group_1370,yidD,oppA_2,group_2360,group_3030,comEA,group_1929,group_1839,rnhB,mhqN,group_2491,group_1668,gr
oup_2058,group_14467,group_3020,mccF,rhaR_2,fbpC,nadC,amiC_2,group_1452,csd_1,group_3061,pdaA_1,queG_2,group_2508,atpC,atpH,tilS_1,dnaX_1,ykfA,niaR,dinB,thpR,group_2856,mutT4,glpQ_2,bpsA,group_1
943,paeR7IM,galE1,group_4011,group_279,group_1781,group_2892,yknW,xre_1,speG_3,group_2718,recO,group_4625,pucA,group_1411,elaA,group_3337,group_8617,btr_1,group_3547,metQ_1,aroE,group_3730,group
_5557,yqgN,ycgR,ptsG_2,msbA_3,group_429,group_4246,group_2454,fecE_3,group_697,dosC_1,rsmH_2,surE,group_1597,sleB_1,group_4323,ywaD,ypeB,group_3052,group_2176,sleB_2,licH,group_10741,group_2501
,ndhF_2,mshA_7,group_1683,proS_1,coaE,mgtB,ribE,group_3996,uppS_1,yodJ_2,ywqD,yraA,group_2690,group_3155,sphS,murF,ftsL,group_2231,uppS_2,baeS,glnQ_3,nit1,group_892,group_494,group_3207,group_518
2,purF_3,group_1521,ywiB,sigX,group_1006,sodB,group_3762,group_4636,group_12,rimI_1,mepM_1,group_2472,atoD,group_3724,group_1641,group_6228,group_2009,group_447,mhqR,group_3371,yfiH,group_5054,
group_5558,group_906,ywqC,ywtF,group_368,mntR,metN_2,divIB,group_3149,group_7346,pcrA_2,iolW,group_836,birA,speH,group_6244,group_1087,recX,group_1866,group_2382,adrA,xynC,htrB,group_3211,group_
3526,msrB,group_3045,hisZ,mprA,group_2309,mftC_1,group_437,ykoC,cloSI,hit_1,ydaD,pbuO,alr1_1,bcrC,group_1313,cph2,group_2725,bcsB,ethR,msrC,bmrR_4,group_8634,cysT,yjjP_2,group_963,group_1733,group
_246,glf,epsL,group_459,group_1013,group_4598,group_2824,pepD_3,trxA_3,group_950,group_3223,group_4037,group_3387,lacE,group_4318,walK_2,group_1337,group_573,group_6243,group_8631,yicJ,mntA,group
_3722,thiM_1,group_4612,group_1268,def_2,group_5048,zitR,group_3192,group_2630,ycf3,group_3160,yndE_3,gerBA_1,group_2668,cdd_1,fdx_1,metF,panC,group_2502,group_3188,bglK,selO,group_2622,lplJ_1,grou
p_2142,group_1727,group_3548,group_264,rbn,hemN_1,menG,nahD,sigW_2,srtD,group_4012,group_4296,araC,group_3362,crr_1,group_1266,group_123,group_2462,mgrA,group_5053,aroA,nadB,group_1311,tauB_2,g
roup_3323,group_1723,hypE,group_3990,mepA_1,glcB_1,group_1442,group_1593,group_877,group_3550,group_3543,group_1309,group_3790,group_3565,group_2724,group_2359,ltaE,group_2246,group_1824,group_
2124,aroK,cotI_5,group_1368,group_8606,group_5074,resE_11,group_1216,bshA,group_5577,wapA_2,group_3522,steT_2,rffG,rmlA,vioA,epsM,group_5044,dsbD_3,mcp4_1,group_67,group_2191,truA_2,group_1276,pi
mB,group_771,pgcA_1,tesA,yfbR,group_5045,group_3325,group_92,group_1329,yqhS,group_2286,lytC_5,group_812,group_1025,group_1741,mocA,group_1399,ywqE,group_4001,group_338,group_2752,folA,group_35
59,group_8659,tadA,dnaI,fldB_1,group_1563,hopD2,group_2330,sacX,murQ,act,group_1017,lytC_3,group_3558,group_4033,group_4031,yrrB,group_814,group_2157,group_318,fabD,group_1267,phoR_3,group_1483,yn
dE_1,group_2136,pspE_1,agrA,tuaC,group_2474,fosX,legF,group_5055,group_7191,perR_1,mepH,gluP,group_1602,group_2881,group_1215,group_13,cpo,group_5579,group_7216,legI,group_2700,group_3564,ytrA_5,y
ndE_4,group_3018,safA,group_3355,group_4628,group_501,psuK_2,group_3393,group_922,group_2379,ydjZ_4,group_3720,hypA_2,group_2059,group_2057,group_4,msrA1,group_2132,group_5544,inlA_4,group_3765
,group_5071,azoB,group_372,sunS_1,group_1675,phzF,lysN_1,folE,group_2129,group_1222,group_1759,group_1687,epsF,bltD_2,lytC_4,group_3392,thyA,group_3773,queE,group_2997,yloB_2,menH,group_3563,group
_1146,group_1740,group_1865,group_2480,comEC_1,group_2692,group_1056,group_478,hydB,tgt_1,grdB_1,group_4303,xerD_1,group_1110,group_4373,grdA,group_2755,mshD,group_1734,hisE,rocR,group_3383,gro
up_455,group_643,group_1922,feoB_1,group_1428,ylbJ,group_4271,yeiR,patB_1,gltT,tdcF,group_373,group_3167,deoR,group_3003,xpt_2,group_3361,tcrY,amaB,slt_2,rutB,group_3368,group_5578,lytD,accB,group_14
859,group_2722,sigV,fruA_1,group_2574,group_4644,group_1909,group_1479,group_2171,ysnF_2,hypF,group_1878,group_4662,grdA1,ypeA,group_3721,msbA_4,yafP,group_5075,group_2072,group_231,ypdB_2,grou
p_2162,bioD1,group_1446,group_1162,epsG,yxdM_2,yfiR,group_6252,sulD,hypB_1,group_4616,group_14446,group_3024,group_3008,group_5134,group_2276,group_2122,group_3320,yajR,group_1968,ydeA,pncA,gro
up_740,hypC,hypD,group_1973,group_2476,group_2182,hit_2,pspE_2,group_7222,group_3540,group_1754,group_2889,tmcAL,group_2452,group_566,ddc,group_2041,group_6266,yxdM_3,group_2134,group_3727,pgs
A1,group_2728,group_4285,group_3165,group_918,group_319,group_4325,group_3204,cypB,group_2719,group_1171,secE,icaC,group_6285,group_3334,lytC_8,albA,yxlF_1,grdB_2,group_3507,group_954,yycB,group_
10757,yusO,group_4255,oppC_1,dppB_1,hbpA_2,group_1915,group_3039,group_5084,group_3381,csxC,iga,amiC,group_2293,group_5571,yndE_2,group_2594,gsiA,group_3551,group_10744,group_712,soxS_1,group_5
066,group_365,group_73,brnQ_1,group_7211,group_471,rplL_1,group_4613,natA_1,group_4606,group_252,lysO,uvrC_2,group_3992,group_7231,mntP_1,group_2269,group_2832,glpQ_3,group_1588,group_2275,group

_1845,group_1884,group_3080,group_2489,group_2337,yxeM_2,group_2457,group_1570,group_299,hisC,group_1718,comC,yndE_5,group_834,group_2174,group_3059,slyA_1,group_2022,group_2998,group_3520,group_5153,gerAB,gerBA_3,group_2126,gerAC_1,group_7233,gtf1,group_6256,lutA,tetD_2,group_4614,group_2695,group_5037,group_3338,group_3406,group_4646,group_3533,group_1770,group_4641,group_3709,group_448,group_2484,group_845,group_10723,group_2496,group_5042,group_1840,group_3552,ybdM,group_5077,group_1772,arsA,acr3,group_3511,uvrB_1,group_4618,group_2193,group_1607,hsdM,rhdA,group_4025,group_3725,cbiE,yusV_2,group_1525,feuC_2,group_4316,yxeB_2,cat_2,group_835,group_1935,group_3006,group_5575,mntR_1,opuBB,group_10739,group_3379,group_3162,group_4620,group_5563,group_3766,yxdL_4,group_7213,group_4279,chiD_1,nirC,group_513,group_6258,cbh,group_2927,group_3740,ytcD,pezA,group_3367,group_6225,group_2734,group_8608,group_7215,group_4643,bltD_1,walR_9,group_4024,spoVAD_2,group_2691,xylR,group_6283,group_14482,group_5092,group_1598,group_1599,group_1771,ricR,ctpV,menG_1,group_22449,group_4312,group_3391,group_2068,yhcN,ysnE,yofA,thrC_1,phoR_8,smc_4,group_3336,bglC_2,ykuD_1,group_2236,group_1963,group_2877,group_2268,group_6262,group_22481,group_5576,group_2082,lyc_1,group_2463,group_5581,group_5091,group_8230,group_22483,group_2500,group_14449,group_5090,xdhA_1,group_4040,pdaA_4,sodC,uviB,group_4639,group_3203,group_7210,group_8626,group_7209,group_10752,group_3513,group_5580,group_14480,group_8638,lspA_1,mscL,group_7225,group_5553,group_22486,group_6254,group_22500,group_10729,group_7239,group_7214,group_7194,group_14442,group_14472,group_2453,senX3,ctrA,group_14469,group_2563,group_14474,group_14473,group_7217,group_20387,walK_4,group_5076,vanY,group_8629,group_5121,group_10755,group_5548,gerBA_2,group_8633,group_4608,group_22440,group_5586,group_22439,group_3457,group_14462,group_22484,group_6226,group_14454,group_7226,group_5564,group_6275,group_4645,group_5093,group_5582,group_10740,group_4595,group_3004,group_4270,group_3377,group_10718+A2935:BI2935

Table S5 Core genes based on 652 isolates and public C. botulinum isolates
smc_3,mleN_3,mutS2_1,group_1030,murC_2,gyrB,group_105,ccmL,prdA_1,rplN,glsA,selA,fldC_3,fabG_1,kduD,truB,oppD_1,thlA,guaB,bmrR_3,trmR,iscS_1,mnmA_1,mreC,yclQ,btuF_1,feoB_2,group_1243,obg,group_128,cstA_2,mnaA,murAA,phoR_5,ndhI,manR,sph,uacT_1,potB,cobQ_2,smc_2,flgG_2,mepA_4,hrcA,pyrK,gatB,pepV,group_1423,yvcJ,cdaA,feuB_2,rpsE,glmU,panF,nasF,typA,group_1493,hcp,yigZ,pbpA,yjjP_1,pglF,pepD_2,tgt,pyrD,lysC_2,serB1_1,trxB_1,potD,cynR_1,polC,ffh,rasP,udk_1,glyA,thiC,bcd_2,engB,uvrA_2,licC,buk2_2,lhgO_2,dnaK_2,pta_1,group_1737,tauB,group_1751,larA_3,braC,cobD_1,pyrC,rnjA_1,group_1843,purN,group_1849,cbiD,selD,proC,carC_2,rodA,tdcB,glpX,hslO,groL,group_1965,lptB,dnaN,hemC,iap_1,magA,purC,spoVAD,group_205,yhaM_1,spoIIIE,lon1,tagO,hemZ_2,group_2138,cutD,carE_1,grpE,gpr,ruvA,gph,rbsC,pcrA_1,livH,rny_2,group_22507,rplV,hbd,pyrB,prfC,ycdX,kdsD,aspC,group_2314,mreB_1,cobQ_1,msbA_2,ssuC_1,group_2385,group_2392,adhE_3,yrrK,oppB,dxs_1,group_2487,hemB,rny_1,rplD,hadI_1,natB_1,group_2600,group_2607,purR,yehU,cphB,pepIP,tal_1,carD_1,group_271,pyrR_2,carE_3,whiA,murB,ytrA_1,lemA,infB,gloC,pduV,alaS,hadC,csoR_1,purE_1,cshA,dgkA,group_2861,dppC_1,hgdC_1,cysC,ddl,yqeN,yacP,flhB_1,group_3029,nadE,ftsE,arcC1,rplY,tktB,rpoA,fldH,rnfC_4,natA_2,mazG,yebC,spoIIAA,pfkA,spmA,hadI_2,mcpB_7,group_3510,carC_1,minD,minC,clpX,spmB,prs,rpsC,mutS2_2,group_3745,dadA,carD_3,korB,korA,srrA_4,hup,map_1,rplE,artQ_2,yvhJ,lon2_2,tig,iscU,mcpB_1,darA,group_406,mcpB_2,cymR_1,ntpG,group_4283,ktrA,padE,feoA_2,group_4302,rsxA,purL,dgt,polA,minE,ulaB,hadB,ccpN,clpP,yheS,rplC,mcpS,ftsH_2,dnaE,ccmK,pflB,dnaK_1,rpsT,group_5081,yugI,dhaK,cobC,ppdK,pepT,glnQ,pduA_2,yabJ,groS,group_5585,rpoB,adk,rpsS,flhA,mepA_6,prsA1,wbpA,rimO_1,ntpA,ribZ,group_6234,fur_1,feoA_1,upp,selB,group_641,pgdA_2,mtaD,ptsI,metG_2,glnA,spoVB_1,mrdB_2,acpP_1,cobD_2,purM,mtaB,rplF,group_726,feoB_4,norM,oppA_1,ftsH4,pnp,group_765,purH,gatA,feoB_3,group_823,cbiJ,fabH_1,macB_2,ydcP,pduX,group_8651,group_8656,recF_2,megL_2,glmM,group_896,lysC_1,group_910,mfd,group_935,tam,nrnA,dpaL,speA_1,pheS,pgi,hmuU_2,gcvPB,pgpH

Table S6 Basic information of public isolates

ID	Species	Isolate	Toxin gene subtype	type botulism	country	year	ST	aroE	mdh	aceK	oppB	rpoB	recA	hsp	Genome accession	Event	Strain received from
SRR8527709	Clostridium botulinum	SRR8527709	A1B5	Foodborne	Ireland	2006	4	9	10	6	9	8	7	8	SGMI00000000	Event I	Isolated at PHE Colindale, UK
SRR8527710	Clostridium botulinum	SRR8527710	A1B5	Foodborne	Ireland	2006	4	9	10	6	9	8	7	8	SGMJ00000000	Event I	Isolated at PHE Colindale, UK
SRR8527766	Clostridium botulinum	SRR8527766	A1	Foodborne	UK	2011	19	13	10	12	10	9	8	7	SGNO00000000	Event II	Isolated at PHE Colindale, UK
SRR8527763	Clostridium botulinum	SRR8527763	A1	Foodborne	UK	2011	19	13	10	12	10	9	8	7	SGKK00000000	Event II	Isolated at PHE Colindale, UK
SRR8527761	Clostridium botulinum	SRR8527761	A1	Foodborne	UK	2011	19	13	10	12	10	9	8	7	SGKM00000000	Event II	Isolated at PHE Colindale, UK
SRR8527762	Clostridium botulinum	SRR8527762	A1	Foodborne	UK	2011	19	13	10	12	10	9	8	7	SGKN00000000	Event II	Isolated at PHE Colindale, UK
SRR8527653	Clostridium botulinum	SRR8527653	B2	Foodborne	UK	2012	51	13	8	15	19	8	7	8	SGKH00000000	Event III	Isolated at PHE Colindale, UK
SRR8527656	Clostridium botulinum	SRR8527656	B2	Foodborne	UK	2012	51	13	8	15	19	8	7	8	SGKI00000000	Event III	Isolated at PHE Colindale, UK
SRR8527655	Clostridium botulinum	SRR8527655	B2	Foodborne	UK	2012	51	13	8	15	19	8	7	8	SGKJ00000000	Event III	Isolated at PHE Colindale, UK
SRR8981586	Clostridium botulinum	CDC 13280	A1B5	foodborne	USA	1972	4	9	10	6	9	8	7	8	SWXR00000000	Other	A. East, Institute of Food Research, UK (1997)
SRR8981582	Clostridium botulinum	NCTC 2916	A1B5	foodborne	USA	1929	4	9	10	6	9	8	7	8	SWXN00000000	Other	M. Wictome, CAMR, UK (1997)
SRR8981000	Clostridium botulinum	AIP 580-86	B2	foodborne	Spain	1986	57	13	8	32	5	8	6	8	SWWJ00000000	Other	Institut Pasteur, Paris, France (1993) via B.M. Lund
SRR8981317	Clostridium botulinum	NCTC 3806	A1	foodborne	USA	1920s	1	13	6	13	10	9	8	9	SWRB00000000	Other	P. Barrett, Unilever Research, UK (2014)
SRR8981584	Clostridium botulinum	CDC 1690	A1	foodborne	USA	1977	1	13	6	13	10	9	8	9	SWXP00000000	Other	A. East, Institute of Food Research, UK (1997)
SRR8981610	Clostridium botulinum	16037	A1	foodborne	USA	1974	1	13	6	13	10	9	8	9	SWZU00000000	Other	Leatherhead Food RA (1981)
SRR8981002	Clostridium botulinum	ATCC 3502	A1	foodborne	USA	1920s	9	13	6	9	10	9	8	9	SWWL00000000	Other	H. Tranter, CAMR, UK (2000)
SRR8980987	Clostridium botulinum	2045/98	B2	foodborne	UK	1998	67	10	10	12	10	9	8	7	SWSW00000000	Other	Isolated at PHE Colindale, UK
SRR8981518	Clostridium botulinum	B2192	B2	foodborne	unknown	unknown	13	7	8	8	6	7	4	9	SXBH00000000	Other	T. Grenda, NVRI, Pulawy, Poland (2016)
SRR8981625	Clostridium botulinum	CDC 15044	B1	foodborne	USA	1973	34	16	12	20	8	10	12	9	SWZI00000000	Other	C. Hatheway, CDC, USA (1987)
SRR8981424	Clostridium botulinum	Langeland	F1	foodborne	Denmark	1960	88	34	11	20	8	10	10	9	SXFG00000000	Other	P. Barrett, Unilever Research, UK (2014)
SRR8981653	Clostridium botulinum	Langeland (2)	F1	foodborne	Denmark	1960	88	34	11	20	8	10	10	9	SWYH00000000	Other	NCTC (1993)
SRR8981479	Clostridium botulinum	5052	F1	foodborne	Denmark	1960	88	34	11	20	8	10	10	9	SXCR00000000	Other	P. Barrett, Unilever Research, UK (2014)
